# Antifungal Peptides with Unexpected Structure from a Library of Synthetic Analogs of Host-Defense Peptide Rigin

**DOI:** 10.3390/ijms26051900

**Published:** 2025-02-22

**Authors:** Marina Porras, Dácil Hernández, Alicia Boto

**Affiliations:** Instituto de Productos Naturales y Agrobiología del CSIC, Avda. Astrofísico Fco. Sánchez, 3, 38206 La Laguna, Tenerife, Spain; mporras@ipna.csic.es

**Keywords:** antifungals, host-defense peptides, site-selective modification, combinatorial synthesis, peptide libraries, in silico ADME

## Abstract

Rising antifungal resistance prompted the World Health Organization and the Food and Agriculture Organization to bring attention to the consequences of this threat to human, animal, and environmental health, and food security. In addition, there is an alarming cross-species pathogenicity. New antifungal agents are urgently needed, preferably with a low induction of antimicrobial resistance (AMR). Among the most promising novel antimicrobials are the host-defense peptides, which present potent anti-infective properties and elicit low or negligible AMR. The rapid creation of libraries of host-defense peptides is highlighted by the synthesis of analogs of the immunomodulator and antimicrobial peptide rigin. Starting from smaller fragments incorporating hydroxyproline customizable units, which can be selectively cleaved and modified to give different lateral chains and N-substituents, two fragment libraries were built. Then the fragments were combined to give a library of rigin analogs, some of which displayed a potent antifungal activity not observed in the natural peptide. Surprisingly, the most active ones were N-substituted and lateral-chain protected analogs, while the free cationic peptides displayed low direct activity. This work shows that the strategy of combining site-selective peptide modification and a combinatorial approach can provide peptide-diverse libraries, where unexpected drug leads may be identified.

## 1. Introduction

The growing threat of antifungal resistance (AFR) prompted the World Health Organization (WHO) in late 2022 to release for the first time a list of fungal priority pathogens (WHO FPPL), bringing attention to the limited number of treatments available [1]. The Food and Agriculture Organization of the United Nations (FAO) has also advised that fungal diseases in plants and cattle are on a sharp increase, thus threatening food security [2]. Moreover, there is an alarming cross-species (even cross-kingdoms) pathogenicity [3]. For instance, *Fusarium oxysporum*, considered a major plant pathogen causing multimillion losses, is now emerging as a human pathogen causing severe infections, particularly in immunocompromised patients. This pathogen invades the vascular system of plants, while in humans it causes lethal blood infections, and is resistant to standard treatments [3,4].

Among the novel families of antifungal compounds, host-defense peptides (HDPs) are particularly promising, since they display a multiple mode of action that hinders the appearance of resistance [5,6,7]. Many of them act by disrupting the pathogen membrane, but they can also alter the cellular metabolism, target the nucleic acids, or act as immunomodulators. In fact, they have been used by animals and plants for millions of years as a defense against a variety of pathogens, with a negligible induction of resistance. Moreover, they can be synergistic with current antimicrobials.

**Figure 1:**
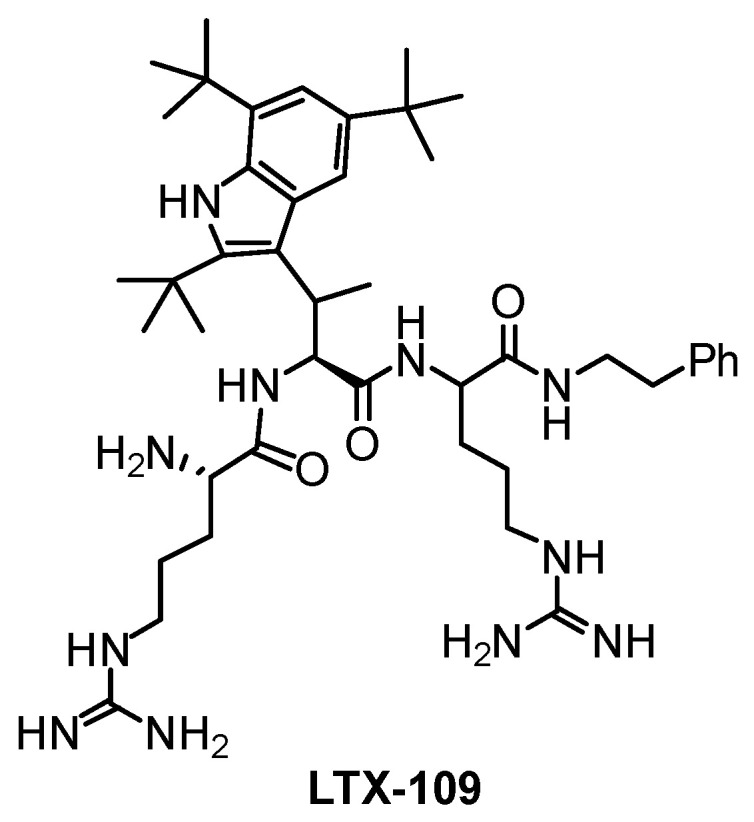
Antifungal HDP analog LTX-109 with an unnatural residue.

However, a problem with natural HDPs is their lability to proteases, resulting in short half-lives, and limiting their clinical use (intravenous administration, topical treatments). Therefore, some groups have prepared analogs with non-proteinogenic units, such as D-amino acids or unnatural L-amino acids, to overcome this problem [5]. Another limitation is their high cost of production, so truncated versions [8] or small synthetic analogs have been developed [5,9]. For instance, Lytixar^®^ (LTX-109, Figure 1) was developed against bacterial topical infections, but also displayed high antifungal activity against *Candida* infections and onychomycosis, superior to the current treatments [10].

Although the HDPs have provided both FDA-approved drugs and drug candidates in advanced clinical stages, few belong to antifungal compounds, and none are approved for systemic fungal infections [5,6]. Therefore, further research in this area is encouraged. Regarding the primary sector, host-defense peptides have started to attract attention as long-lasting antifungals [7].

In order to rapidly create peptide libraries for biological evaluation and to determine structure–activity relationships, we decided to combine the advantages of site-selective peptide modification [11] and combinatorial chemistry [12,13,14]. Moreover, when a reference drug candidate acts through a multiple mechanism of action (such as host-defense peptides), or when its biological target is unclear, second-generation ‘hits’ are preferably discovered by combining selectively modified sections of the original structure [5,6,7,9,12].

Site-selective modification of peptides can be carried out using “customizable units” such as serine, threonine, or hydroxyproline, among others [15,16]. The customizable unit can be modified without affecting the remaining residues. Starting from one or a few “parent peptides” a relatively large library can be prepared quickly, saving time and materials with respect to the traditional ‘bead by bead’ approach. In previous works [15,16,17], we introduced hydroxyproline (Hyp) as a customizable unit, since its domino oxidative radical fragmentation provides an *N*-substituted 4-oxohomoalanine residue, which has retained the configuration of the starting Hyp unit. In addition, both lateral chains can be functionalized independently, to give a variety of compounds, as will be discussed later.

In cases where selective modification is used to generate different fragments of a target peptide rather than the whole peptide, a combinatorial strategy to attach these fragments could increase the diversity of the library in a short time. Herein, we present a proof of concept for this strategy through the synthesis of rigin analogs and the identification of new antifungal lead compounds with an unexpected substitution pattern.

**Figure 2:**
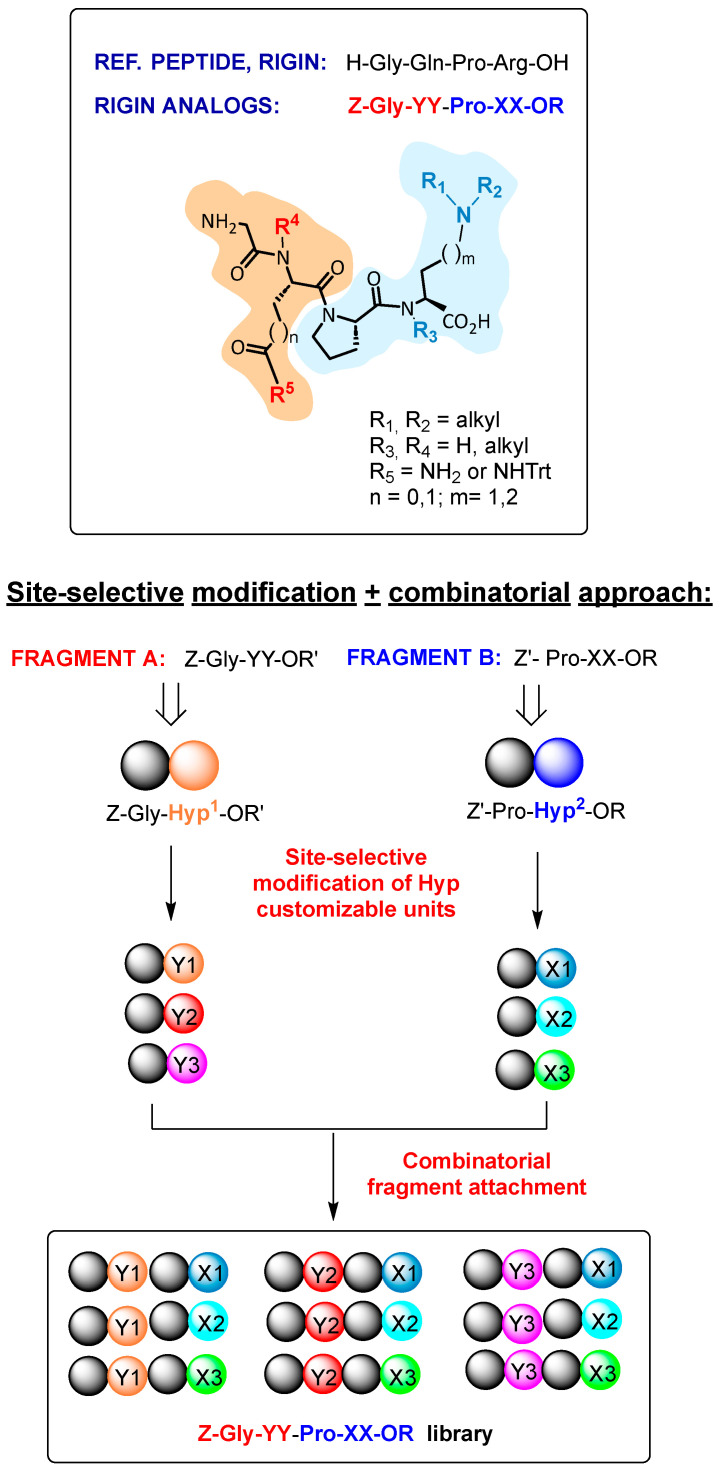
A site-selective peptide modification followed by a combinatorial fragment attachment provides a diverse library for drug discovery. Application to analogs of host-defense peptide rigin.

Rigin (H-Gly-Gln-Pro-Arg-OH) is a hydrolysis product of human Immunoglobulin G, with a potent immunomodulatory action, such as the regulation of antibody production in lymphocytes, the cytotoxic activity of killer T-cells, or the release of lysozymes [18,19,20]. However, rigin is easily degraded in vivo by proteases, making it difficult to use as a drug. The replacement of proteinogenic residues with non-natural ones could afford derivatives that retain their pharmacological activity but present superior stability [5]. Surprisingly, little work has been carried out to study such derivatives [21,22]. We reasoned that a combinatorial approach, in which selectively modified fragments of rigin were attached, could provide a valuable library for determining structure–activity relationships (Figure 2).

In the case of host-defense peptides, it is vital that modifications maintain an appropriate balance between hydrophobic and cationic amino acids, or bioactivity could be drastically reduced [23,24]. Therefore, in the work described herein, the cationic residue Arg was in most cases replaced by an unnatural cationic unit, and Gln was replaced by Asn. Since the degree of protection of the residues can affect the hydrophobic or cationic character of the whole peptide, libraries of differently protected peptides would be useful to draw structure–activity relationships.

Moreover, our methodology for the modification of customizable units allows the preparation of *N*-alkyl derivatives, whose hydrophobicity, volume, and interactions with biological targets through hydrogen bonds differ from the original peptides [16,25]. As commented later, the resultant *N*-alkyl groups are *N*,*O*-acetals that can be readily removed, and thus, a comparison of the *N*-alkylated and non-alkylated HDP analogs is possible.

The site-selective methodology commented herein would also allow the transformation of customizable units either in cationic residues, such as 4-(amino)-homoalanines, or hydrophobic units, such as α-alkylglycines with hydrophobic lateral chains. Therefore, once these different fragments undergo attachment in a combinatorial way, a variety of peptides with different structures and cationic character can be obtained.

Finally, a particularly interesting issue is the development of short peptides that retain antimicrobial activity [24,26]. These peptides not only have lower production costs but can also present improved ADMET properties. The comparison between short precursors and their combination products would provide useful information on the requirements of peptide length for biological activity.

## 2. Results and Discussion

This Section provides a description of the experimental results (Section 2.1, Section 2.2 and Section 2.3) and their interpretation (Section 2.4, Discussion).

### 2.1. Preparation of Libraries

**Figure 3:**
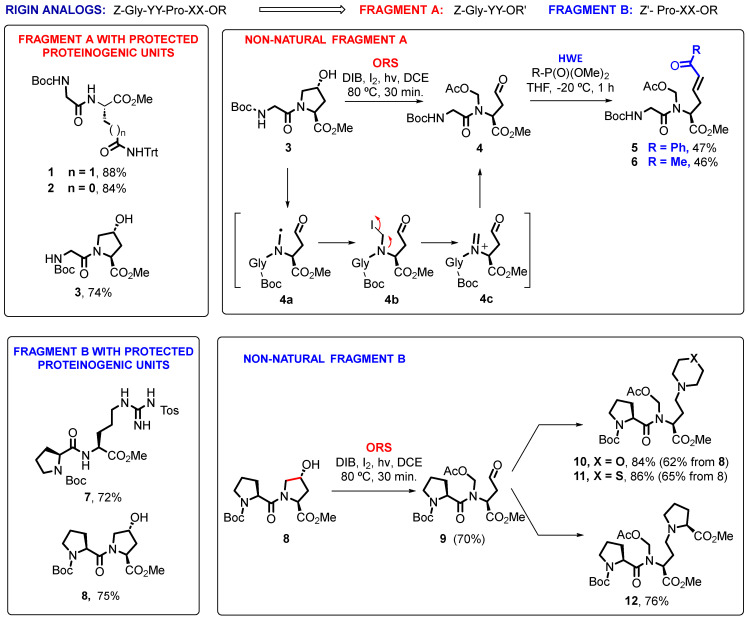
AHL analogs as potential quorum quenchers.

As shown in Figure 3, the production of rigin analogs Z-Gly-YY-Pro-XX-OR involved the production of “Fragment A” dipeptides Z-Gly-YY-OR′ and “Fragment B” dipeptides Z′-Pro-XX-OR.

The “reference” proteinogenic fragments **1**–**3** and **7**–**8** were synthesized using standard peptide chemistry. For peptides with unnatural residues XX and YY, these amino acids were prepared from customizable Hyp units. For instance, the domino oxidative radical scission of Boc-Gly-Hyp-OMe (**3**, Figure 3) afforded a C-radical intermediate **4a**, which was trapped by iodine or other halogenated species [11] to give haloderivatives such as **4b**. Extrusion of iodide promoted by the adjacent nitrogen function afforded an intermediate acyliminium ion **4c**, which could be trapped by acetate ions from the reagent (diacetoxyiodo)benzene, finally affording the intermediate aldehyde **4**. This aldehyde was not purified due to its instability, but immediately underwent a Horner–Wadsworth–Emmons reaction to give *N*-acetoxymethyl α-alkylglycines **5** or **6**. In this case, a cationic residue in the original peptide (Gln) was replaced by hydrophobic ones. In the case where a cationic residue was desired, the strategy shown in the conversion **8** → **10**–**12** was applied. Thus, the oxidative radical scission generated an aldehyde **9**, which was treated under reductive amination conditions to give dipeptides **10**–**12**. These residues present bulky, but removable, *N-*substituents. It should be noticed that the introduction of *N*-substituents in peptides is rather difficult, particularly when they are larger than Me groups. This protocol allows for the ready preparation of such residues, which will influence bioactivity, as will be discussed later.

**Figure 4:**
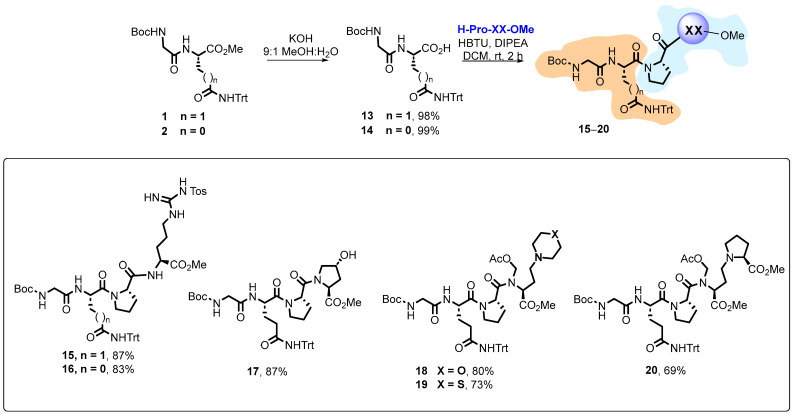
Synthesis of rigin tetrapeptide analogs with different protecting groups and terminal *N*-alkyl residues.

The dipeptides were saponified to provide “Fragment A” free acid derivatives (Figure 4). While the saponification of esters **1**–**2** proceeded in excellent yields, the saponification of compounds **5**–**6** gave complex mixtures of products, not only because of simultaneous cleavage of the acetoxymethyl group but also from different addition reactions to the conjugated ketones. Saturated derivatives and other α-alkylglycines with different hydrophobic and cationic lateral chains will be tried in the future.

In any case, peptide coupling was carried out with free acids **13** and **14**. On the contrary, the quantitative deprotection of “Fragment B” compounds **7**–**8** and **10**–**12** in acid media proceeded as expected, and the resulting amines were coupled to the free acids in good yields. In this first library, compounds **18**–**20** retained the *N*,*O*-acetal in the C-terminal position, which conditioned their physicochemical and biological properties.

**Figure 5:**
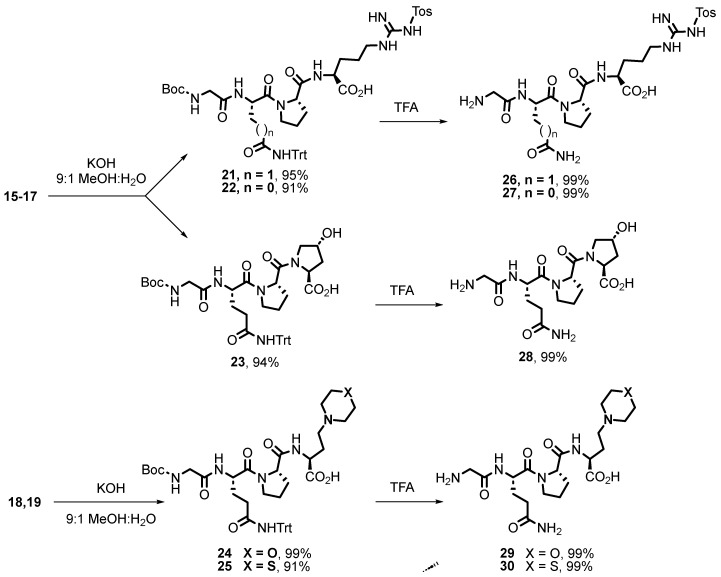
Synthesis of rigin tetrapeptide analogs prior to antimicrobial evaluation.

Then, the *N*,*O*-acetals and the *C*-terminal ester were hydrolyzed in basic media to give products **21**–**25** (Figure 5) in excellent yield. In the next step, acid treatment with TFA removed the Boc and trityl protecting groups to give the final peptides **26**–**30**, with several free cationic groups.

### 2.2. Evaluation of Antifungal Activity

The antifungal activities of these rigin analogs were then evaluated against three fungal pathogens that cause multimillion losses in the primary sector: *Alternaria alternata*, *Botrytis cinerea*, and *Fusarium oxysporum* [1,2,3,4]. The latter can also be a dangerous human pathogen, and cause severe blood infections as mentioned before [3,4]. The results are shown in Table 1.

Surprisingly, the most promising antifungal peptide was the fully protected tetrapeptide **18**, presenting a lateral chain with a morpholino group [27] and a *C*-terminal *N*-acetoxyacetal. This compound displayed a 79% radial growth inhibition of grapevine pathogen *Botrytis cinerea* at 500 µM, a dose usual in agriculture. It was also active against *A. alternata* (42%). In contrast, the deprotected analog **29**, which presents several free cationic groups, showed little activity (9%). In a similar way, the thiomorpholino derivative **25** displayed 43% inhibition against the same pathogen at the same dose, but its deprotected analog **30** presented little activity (15.6%). Interestingly, most peptides were inactive against *F. oxysporum*, and only dipeptide **5** displayed moderate activity (42.7% at 500 µM), likely due to the reaction of the conjugated ketone with biological nucleophiles.

### 2.3. In Silico ADME Study of Rigin Analogs

The predicted ADME/T properties of selected compounds are shown in Table 2: the most active dipeptide **5**, the fully protected tetrapeptides **18** and **19**, and for comparison, the semi- or deprotected thiomorpholine derivatives **25** and **30**. The in silico study was carried out with the SwissADME tool (www.swissadme.ch, accessed 18 February 2025) [28,29]. It must be said that many predictions are accurate for usual-size drug-like molecules but should be taken as orientative for biomolecules such as peptides with relatively high MW (>500).

Dipeptide **5** deserves to be discussed separately since its predicted physicochemical properties and mechanism of action are different from those of the tetrapeptides. The ratio of donor/acceptor bonds is the lowest of the five compounds. In addition, its Topological Polar Surface (TPSA) was half the value for the tetrapeptides, which in turn influences the properties of distribution, absorption, brain access, etc. [30].

The calculated partition coefficient between water and *n*-octanol log P_o/w_ was positive for dipeptide **5**, supporting its lipophilic nature [31,32]. However, its solubility should still be acceptable (LogS = −3.36; ESOL method; Insoluble < −10 < Poorly < −6 < Moderately < −4) [28,32]. Its predicted gastrointestinal absorption was high, but it could not cross the BBB [28].

Drug interactions with different biological targets were studied next. The in silico study suggested that compound **5** was a substrate of the permeability glycoprotein (P-gp, an ABC transporter) and thus crossing through different barriers (GI wall, lumen, etc.) was facilitated [33]. Fortunately, it was not an inhibitor of most cytochrome isoforms, and therefore the risk of drug accumulation and side effects was low [34].

The oral bioavailability can be determined using different filters, but Lipinsky (Pfizer) is the best known. Its rules indicate that for good bioavailability, the compound should present MW < 500, MLOGP < 415, N or O < 10, and NH or OH < 5. Therefore, dipeptide **5** met Lipinsky’s criteria [32].

In addition, the Abbot Bioavailability Score predicts whether a compound could achieve at least 10% oral bioavailability in rat or Caco-2 permeability. The calculated value for compound **5** (0.55) also met this criterion [35].

Finally, the PAINS (pan assay interference compounds) alerts point out compounds that may interact with many protein targets, giving false positives in assays, which was not the case for compound **5 [36]**. However, the Brenk alarm (for toxicity or unstability risks) indicated that the compound could be a Michael acceptor, which supports the proposed (positive) mechanism of antimicrobial action [37].

In the tetrapeptides, although TPSA values were similar, the ratio of donor/acceptor bonds increased as deprotection advanced. Accordingly, compounds **18** and **19** were predicted to be poorly soluble, while the acid **25** should be moderately soluble and the deprotected product **30** should be highly soluble. However, GI absorption for all compounds would be low, and no BBB permeation was expected. The protected tetrapeptides **18** and **19** and the acid **25** were likely substrates for protein P-gp, while the soluble product **30** was not. None of the tetrapeptides was a potential CYP inhibitor. However, the three tetrapeptides **18**, **19**, and **25** did not meet two Lipinsky criteria (MW > 500, Number O > 10), while the deprotected compound **30** only violated one rule (number O > 10). The Abbot bioavailability score was also appropriate for compound **30**, but was too low for the protected analogs **18**, **19**, and **25**. This, however, does not exclude these compounds for further pharmaceutical development, rather indicates that a suitable conjugation or drug formulation should be developed. None of the compounds gave PAINS alerts. The Brenk alarm pointed out more than two ester groups (unstability), but this result is because the stable carbamate groups are considered an ester.

**Figure 6:**
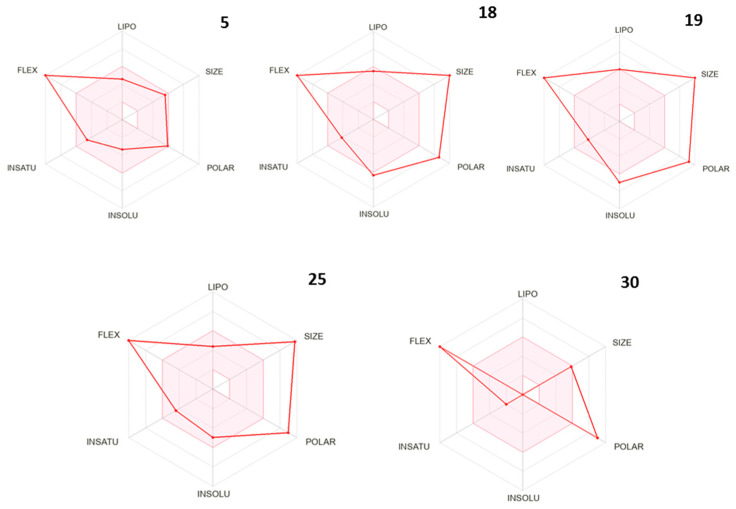
Representations of oral bioavailability of selected compounds **5**, **18**, **19**, **25**, and **30**. From the top and clockwise, the web points read: LIPO (liposolubility), SIZE, POLAR, INSOLU (insolubility), INSATU (insaturation degree), FLEX (flexibility).

Figure 6 depicts the bioavailability radar of the selected compounds. The radar is a representation of their oral bioavailability, based on their molecular and physicochemical properties. For small-size molecules (MW < 500), the best values would fall in the pink area, but for larger biomolecules, the results are orientative.

In our case, only compounds **5** and **30** present MW between 150 and 500 g/mol (SIZE in the pink area). Nevertheless, the radar predicts that dipeptide **5** would present satisfactory oral bioavailability, while the promising tetrapeptides **18**, **19**, and **25** would not. However, as for many peptidic drugs, this shortcoming can be solved with a suitable formulation. We will address this point in future works.

### 2.4. Discussion of Results

The applications of site-selective peptide modification to accelerate drug discovery have already been commented on by our group and others [11,15,16,17,25], and the use of combinatorial chemistry for a similar purpose has often been reported [12,13,14]. However, combining both approaches, as discussed in this Article, can speed up the process even further. The synthesis of a library of analogs of the host-defense peptide rigin was used as a proof of concept for this strategy. Host-defense peptides have a balance of cationic and hydrophobic residues that are necessary to preserve activity [5,6,7,8,9,10]. Svenson [23,24,26] and others [5,6,7,8,9,10,11] have shown that very small synthetic peptides can display high antimicrobial activity if this balance is achieved, and besides, the synthetic units increase the resistance of the peptide to degradation in vivo. Therefore, we considered that the most potent antimicrobial compounds would likely be the unprotected analogs, which are the closest rigin analogs.

Using our versatile site-selective modification with “customizable units” [15,16,17], to transform natural hydroxyproline residues into cationic or hydrophobic residues as needed, a library of dipeptides with unnatural amino acid units was prepared, generally in good yields. This approach provides peptides that are more resistant to degradation by proteases. In addition, this methodology can generate moieties with bulky *N*-substituents, which are very difficult to introduce in a peptide using other methodologies [25]. However, these *N*-substituents were easily removable, so a comparison between protected and unprotected rigin analogs was possible. These synthetic dipeptides and other dipeptides with proteinogenic units were classified as “Fragment A” or “Fragment B”, and then a combinatorial approach was used to attach the units of “Fragment A” to those of “Fragment B”. In this way, the resulting tetrapeptide library could be prepared faster than with a traditional approach. Gradual deprotection steps afforded partially protected and nearly unprotected derivatives with high efficiency.

When the antifungal assays were carried out, surprisingly the most active compounds were not the unprotected analogs, but fully protected tetrapeptides **18** and **19** and dipeptide **5**. The activity of the dipeptide could be explained by its conjugated ketone moiety, which can interact with many nucleophilic (amino, thiol, etc.) groups in biological receptors. However, the superior activity of analogs **18** and **19** was unexpected, proving the value of comparing analog libraries to discover new types of drug candidates.

A possible explanation for these results is that rigin is an immunomodulatory peptide, and therefore rigin and the unprotected analogs may be more potent in vivo than in vitro. This should be the subject of future studies. However, increasing hydrophobicity may also enhance the activity. Thus, Dutta et al. [21] studied the immunomodulatory activity of *N*-palmitoyl- and *N*-cholestanyl-amino-ethyl-rigin amides, and compared it with those of related tetrapeptide tuftsin derivatives, which also have immunomodulatory properties. The new rigin analogs, whose *N*-substitution made them more hydrophobic, displayed superior humoral and cell-mediated immune responses in mice, through the activation of lymphocytes rather than macrophages. In vivo studies with mice infected with *Plasmodium berghei* showed a considerable reduction in parasitemia and mortality rate. The authors postulated an increased interaction with membranes, particularly for the *N*-cholestanyl derivative. In any case, *N*-derivatization with a large hydrophobic group considerably increased, rather than decreased, antimicrobial activity for these rigin analogs.

The comparison of our results with those by Dutta et al. should be made with caution, since the effect of rigin derivatives on a protozoan pathogen may be different from the effect on a fungal pathogen. Moreover, in our case, a direct effect is observed, not one mediated by an immune system. In the case of derivatives **5**, **18**, and **19**, the reactive *N*,*O*-acetal may also contribute to the direct effect, but this should be clarified in future studies, where in vitro and in vivo activities should be compared.

Finally, in silico ADME/T studies of the most promising antifungal compounds **5**, **18**, and **19**, and their unprotected derivatives **25** and **30**, predicted low toxicity (no CYP inhibitor, no PAINS alerts, etc.). No BBB permeability was predicted, but as P-gp substrates, other types of permeability (GI wall, lumen) should be expected, as desired. As for a good oral bioavailability, compounds should have MW between 150 and 500 g/mol, no more than 9(10) rotatable bonds for optimal flexibility, TPSA between 20 and 130 Å^2^, and appropriate lipophilicity (XLOGP3 in the −0.7/+5.0 range) and solubility (logS < 6). In summary, for best results, in the radar plot, all/most peaks should fall in the pink area. Only dipeptide **5** meets these criteria, whereas the tetrapeptides do not. However, these results should be taken with caution, as the larger the biomolecule (MW > 500), the less accurate the bioavailability predictions made by the in silico models. Still, these orientative predictions are useful, and warn that the larger peptide drug candidates may need an appropriate formulation (e.g., liposomes) to avoid bioavailability issues, as needed for other peptide drugs [5,6,9].

## 3. Materials and Methods

### 3.1. Synthetic Procedures and Characterization Data

**General Methods**. Commercially available reagents and solvents were analytical grade or were purified by standard procedures prior to use. All reactions involving air- or moisture-sensitive materials were carried out under a nitrogen atmosphere. Melting points were determined with a hot-stage apparatus and were uncorrected. Optical rotations were measured at the sodium line at ambient temperature (26 °C) in CHCl_3_ solutions. NMR spectra were determined at 500 or 400 MHz for ^1^H and 125.7 or 100.6 MHz for ^13^C, at 25 °C or 70 °C, as stated for each case. Sometimes, due to slower rotamer interconversion at 26 °C, two (or more) sets of signals are visible at room temperature, while only one set of signals (rotamer average) is seen at 70 °C, due to faster rotamer interconversion. For some compounds, the ^1^H NMR spectra show some signals as **broad bands** (br b) due to equilibria between rotamers.

^1^H NMR spectra are reported as follows (s = singlet, d = doublet, t = triplet, dd = doublet of doublets, ddd = doublet of doublet of doublets, q = quartet, m = multiplet, br = broad, br b = broad band, br s = broad singlet; coupling constant(s) in Hz). Mass spectra were carried out using electrospray ionization techniques (ESI). Merck silica gel 60 PF_254_ and 60 (0.063–0.2 mm) were used for preparative thin layer chromatography and column chromatography, respectively. The reagent for TLC analysis was KMnO_4_ in NaOH/K_2_CO_3_ aqueous solution and the TLC was heated until the development of color.

**Fragment Boc-Gly-Gln[Trt]-OMe (1) and its precursors**

***N^α^*-Fluorenylmethyloxycarbonyl-*N^δ^*-trityl-L-glutamine (31):** A solution of the commercial amino acid *N^α^*-fluorenylmethyloxycarbonyl-*N^δ^*-trityl-L-glutamine (6.10 g, 10.0 mmol) in dry methanol (50 mL) was cooled to 0 °C. Thionyl chloride (SOCl_2_, 1.43 g, 0.88 mL, 12.0 mmol, 1.2 equiv.) was then added dropwise and the solution was stirred for 16 h. Subsequently, the solvent was evaporated and the residue was purified by silica gel chromatography (*n*-hexane:EtOAc, 60:40), affording the amino acid **31** (5.69 g, 9.10 mmol, 91%) as an amorphous solid. The product has been previously reported [38].

***N^δ^*-Trityl-L-glutamine methyl ester (32):** A solution of the amino acid **31** (5.62 g, 9 mmol) in dichloromethane (60 mL) at 0 °C was treated with diethylamine (Et_2_NH, 24.85 g, 35 mL), and allowed to reach room temperature (5 h). Then, it was poured onto water, extracted with ethyl acetate, and washed several times with a saturated NaHCO_3_ aqueous solution. Next, the organic phase was dried over anhydrous sodium sulfate, filtered, and concentrated under vacuum, and the residue was purified by silica gel column chromatography (EtOAc), yielding the amino acid **32** (2.98 g, 7.41 mmol, 82%) as an amorphous solid. ^1^H RMN (400 MHz, CDCl_3_, 26 °C) δ_H_ 7.34–7.14 (m, 15H, Trt), 7.01 (s, 1H, NH), 3.70 (s, 3H, OMe), 3.42 (dd, *J* = 8.6, 5.1 Hz, 1H, 2-H), 2.51–2.34 (m, 2H, 4-H_2_), 2.14–2.04 (m, 1H, 3-H_a_), 1.80 (td, *J* = 13.9, 7.5 Hz, 1H, 3-H_b_). ^13^C RMN (100.6 MHz, CDCl_3_, 26 °C) δ_C_ 176.2 (C, CO), 171.2 (C, CO), 144.9 (3 × C, Trt), 128.8 (6 × CH, Trt), 128.0 (6 × CH, Trt), 127.1 (3 × CH, Trt), 70.6 (C, C-N, Trt), 53.8 (CH, 2-C), 52.2 (CH_3_, OMe), 33.9 (CH_2_, 4-C), 30.1 (CH_2_, 3-C). HRMS (ESI) calculated for C_25_H_26_N_2_O_3_Na [M + Na]^+^ 425.1841 found 425.1844.

***N^α^*-(*N*-tert-Butoxycarbonyl-L-glycyl)-*N^δ^*-trityl-L-glutamine methyl ester (1).** To a solution at 0 °C of *N*-tert-butoxycarbonyl-L-glycine (1.30 g, 7.40 mmol) and *N^δ^*-trityl-L-glutamine methyl ester **32** (2.98 g, 7.40 mmol) in dichloromethane (30 mL) were added *N*,*N*-diisopropylethylamine (DIPEA, 1.91 g, 2.6 mL, 14.80 mmol), 1-hydroxybenzotriazole hydrate (HOBt·H_2_O, 1.25 g, 8.14 mmol), and *N*-(3-dimethylaminopropyl)-*N*′-ethylcarbodiimide hydrochloride (EDAC, 1.56 g, 8.14 mmol). The reaction mixture was stirred at 0 °C for one hour and then allowed to reach room temperature over 16 h. The reaction mixture was washed first with saturated NaHCO_3_ solution and then with 5% aqueous HCl, and extracted with dichloromethane. The organic phase was dried over anhydrous sodium sulfate, filtered, and concentrated under vacuum. The reaction crude was purified by silica gel column chromatography (n-hexane: EtOAc, 50:50), obtaining the dipeptide **1** (3.62 g, 6.47 mmol, 88%) as a white foam. ^1^H RMN (500 MHz, CDCl_3_, 26 °C) δ_H_ 7.32–7.16 (m, 15H, Trt), 7.04 (d, *J* = 7.3 Hz, 1H, NH), 6.96 (s, 1H, NH), 4.98–4.90 (br s, 1H, NH), 4.52 (td, *J* = 8.7, 4.0 Hz, 1H, 2-H), 3.75–3.62 (m, 2H, 2′-H_2_), 3.71 (s, 3H, OMe), 2.46–2.32 (m, 2H, 4-H_2_), 2.25–2.16 (m, 1H, 3-H_a_), 1.94 (td, *J* = 15.1, 6.4 Hz, 1H, 3-H_b_), 1.41 (s, 9H, Boc). ^13^C RMN (125.7 MHz, CDCl_3_, 26 °C) δ_C_ 172.2 (C, CO), 171.2 (C, CO), 169.8 (C, CO), 156.0 (C, CO), 144.7 (3 × C, Trt), 128.8 (6 × CH, Trt), 128.1 (6 × CH, Trt), 127.2 (3 × CH, Trt), 80.3 (C, C-O, Boc), 70.8 (C, C-N, Trt), 52.7 (CH_3_, OMe), 52.1 (CH, 2-C), 44.3 (CH_2_, 2′-C), 33.4 (CH_2_, 4-C), 28.4 (3 × CH_3_, Boc), 27.7 (CH_2_, 3-C). HRMS (ESI) calculated for C_32_H_37_N_3_O_6_Na [M + Na]^+^ 582.2580 found 582.2573.

**Fragment Boc-Gly-Asn[Trt]-OMe (2) and its precursors**

***N^α^*-(Fluorenylmethyloxycarbonyl)-*N^γ^*-(trityl)-L-asparagine methyl ester (33).** To a solution at 0 °C of commercial *N^α^*-fluorenylmethyloxycarbonyl-*N^γ^*-trityl-L-asparagine (5.96 g, 10 mmol) in dry methanol (60 mL) was added dropwise SOCl_2_ (1.43 g, 0.87 mL, 12 mmol) and left stirring for 16 h. Subsequently, the solvent was evaporated and the residue was purified by silica gel chromatography (n-hexanes:EtOAc, 70:30), giving the amino acid **33** (5.43 g, 8.90 mmol, 89%) as an amorphous solid. This compound has been previously described [38,39].

***N^γ^*-Trityl-L-asparagine methyl ester (34).** A solution of substrate **33** (5.39 g, 8.83 mmol) in dichloromethane (65 mL) at 0 °C was treated with Et_2_NH (23 g, 32.5 mL) and stirred for 5 h. Then, it was poured into water and extracted with ethyl acetate. The organic layer was washed with an aqueous saturated NaHCO_3_ solution, then dried over sodium sulfate, filtered, and evaporated under vacuum. The residue was purified by column chromatography on silica gel (*n*-hexane:EtOAc, 10:90), yielding amino acid **34** (3.08 g, 7.94 mmol, 90%) as an amorphous solid. The RMN data for this product in CDCl_3_ have been reported [40], and are similar to those described herein. ^1^H RMN (500 MHz, CD_3_OD, 26 °C) δ_H_ 7.29–7.19 (m, 15H, Trt), 3.72 (t, *J* = 5.8 Hz, 1H, 2-H), 3.68 (s, 3H, OMe), 2.74 (d, *J* = 5.8 Hz, 2H, 3-H_2_). ^13^C RMN (125.7 MHz, CD_3_OD, 26 °C) δ_C_ 176.0 (C, CO), 172.2 (C, CO), 146.0 (3 × C, Trt), 130.0 (6 × CH, Trt), 128.7 (6 × CH, Trt), 127.8 (3 × CH, Trt), 71.7 (C, C-N, Trt), 52.7 (CH_3_, OMe), 52.2 (CH, 2-C), 40.9 (CH_2_, 3-C).

***N^α^*-(*N*-tert-Butoxycarbonyl-L-glycyl)-*N^γ^*-trityl-L-asparagine (2).** A solution of *N*-tert-butoxycarbonyl-L-glycine (1.37 g, 7.80 mmol) and **34** (3.03 g, 7.80 mmol) in dichloromethane (30 mL) at 0 °C was treated with EDC (1.64 g, 8.58 mmol) and HOBt·H_2_O (1.32 g, 8.58 mmol), followed by dropwise addition of DIPEA (2.02 g, 2.7 mL, 15.60 mmol). The reaction was stirred at 0 °C for one hour and then allowed to reach room temperature over 16 h. The reaction mixture was washed with saturated aqueous NaHCO_3_ solution, then with 5% aqueous HCl, and finally was extracted with dichloromethane. The organic phase was dried and concentrated as usual, and the residue was purified by column chromatography on silica gel (n-hexane:EtOAc, 50:50), affording the dipeptide **2** (3.57 g, 6.55 mmol, 84%) as an amorphous solid. ^1^H RMN (400 MHz, CDCl_3_, 26 °C) δ_H_ 7.33–7.21 (m, 9H, Trt), 7.15 (d, *J* = 8.4 Hz, 6H, Trt), 7.10 (d, *J* = 8.3 Hz, 1H, NH), 6.76 (s, 1H, NH), 5.07–5.01 (br.s, 1H, NH), 4.80 (td, *J* = 8.5, 4.2 Hz, 1H, 2-H), 3.85–3.66 (m, 2H, 2′-H_2_), 3.66 (s, 3H, OMe), 3.07 (dd, *J* = 16.0, 4.4 Hz, 1H, 3-H_a_), 2.79 (dd, *J* = 16.0, 4.3 Hz, 1H, 3-H_b_), 1.43 (s, 9H, Boc). ^13^C RMN (100.6 MHz, CDCl_3_, 26 °C) δ_C_ 171.2 (C, CO), 169.5 (C, CO), 169.3 (C, CO), 155.9 (C, CO), 144.4 (3 × C, Trt), 128.7 (6 × CH, Trt), 128.1 (6 × CH, Trt), 127.3 (3 × CH, Trt), 80.2 (C, C-O, Boc), 71.0 (C, C-N, Trt), 52.9 (CH_3_, OMe), 49.1 (CH, 2-C), 44.0 (CH_2_, 2′-C), 38.3 (CH_2_, 3-C), 28.4 (3 × CH_3_, Boc). HRMS (ESI) calculated for C_31_H_35_N_3_O_6_Na [M + Na]^+^ 568.2424 found 568.2421.

**Boc-Gly-Hyp-OMe (3) and its products from scission and Horner–Wadsworth–Emmons modification of the lateral chain.**

***N*-(*N*-tert-Butoxycarbonyl-L-glycyl)-4*R*-hydroxy-L-proline (3).** A solution of *N*-tert-butoxycarbonyl-L-glycine (876.0 mg, 5 mmol) and 4*R*-hydroxy-L-proline methyl ester (725.0 mg, 5 mmol) in dichloromethane (17 mL) at 0 °C was treated with EDC (1.06 g, 5.5 mmol), HOBt·H_2_O (842 mg, 5.5 mmol), followed by dropwise addition of DIPEA (1.29 g, 1.73 mL, 10 mmol). The reaction mixture was stirred at 0 °C for one hour and then allowed to reach room temperature over 15 h. The reaction mixture was concentrated under vacuum and the residue was purified by column chromatography on silica gel (n-hexane:EtOAc, 30:70), affording the dipeptide **3** (1.12 g, 3.70 mmol, 74%) as a white foam. The product was reported as a synthetic intermediate, but it was not characterized [41]. ^1^H RMN (500 MHz, CD_3_CN, 70 °C) δ_H_ 5.41–5.28 (br.s, 1H, NH), 4.51–4.42 (m, 2H, 4-H, 2-H), 3.88 (dd, *J* = 17.1, 5.5 Hz, 1H, 2′-H_a_), 3.78 (dd, *J* = 17.0, 5.5 Hz, 1H, 2′-H_b_), 3.70–3.63 (m, 1H, 5-H_a_), 3.67 (s, 3H, OMe), 3.43 (br.d, *J* = 13.3 Hz, 1H, 5-H_b_), 2.22–2.15 (m, 1H, 3-H_a_), 2.07–1.98 (m, 1H, 3-H_b_), 1.44 (s, 9H, Boc). ^13^C RMN (125.7 MHz, CD_3_CN, 70 °C) δ_C_ 173.7 (C, CO), 169.2 (C, CO), 157.1 (C, CO), 80.2 (C, C-O, Boc), 71.0 (CH, 4-C), 59.1 (CH, 2-C), 55.1 (CH_2_, 5-C), 52.8 (CH_3_, OMe), 44.1 (CH_2_, 2′-C), 38.5 (CH_2_, 3-C), 28.9 (3 × CH_3_, Boc). HRMS (ESI) calculated for C_13_H_22_N_2_O_6_Na [M + Na]^+^ 325.1376 found 325.1365.

***N*-acetoxymethyl-*N*-(*N*-tert-butoxycarbonyl-L-glycyl)-6-oxo-6-phenylhex-4-enoate methyl ester (5).** A solution of dipeptide **3** (90.7 mg, 0.3 mmol) in dry dichloroethane (6 mL) was treated with (diacetoxyiodo)benzene (DIB, 193.2 mg, 0.6 mmol) and iodine (38.0 mg, 0.15 mmol). The reaction mixture was stirred at 80 °C for 30 min, under irradiation with visible light. Then, it was poured into a 10% aqueous Na_2_S_2_O_3_ solution and extracted with dichloromethane. The organic layer was dried and evaporated as usual, yielding *N*-acetoxymethyl-*N*-(*N*-tert-butoxycarbonyl-L-glicyl)-4-oxo-L-homoalanine methyl ester. This aldehyde was used without additional purification in the HWE reaction.

Then, the HWE was prepared, by dropwise addition at –20 °C of diethyl (2-oxo-2-phenylethyl) phosphonate (84.7 mg, 72 µL, 0.33 mmol) to a suspension of NaH (60% in mineral oil, 13.5 mg, 0.33 mmol) in dry tetrahydrofuran (1.2 mL). The reaction mixture was stirred for 1 h, and then a solution of the aldehyde in dry THF (1.7 mL) was added dropwise. The stirring continued for 1 h, and then the reaction mixture was poured into water and extracted with diethyl ether. The organic layer was dried and concentrated as usual, and the residue was purified by column chromatography on silica gel (*n*-hexane:EtOAc, 60:40) affording the dipeptide **5** (64.8 mg, 0.14 mmol, 47%) as a yellowish oil. ^1^H RMN (500 MHz, CDCl_3_, 26 °C) δ_H_ 7.92 (d, *J* = 7.0 Hz, 2H, Ph), 7.57 (t, *J* = 7.4 Hz, 1H, Ph), 7.47 (t, *J* = 7.6 Hz, 2H, Ph), 7.00–6.87 (m, 2H, 4-H, 5-H), 5.36 (d, *J* = 12.4 Hz, 1H, OCH_a_N), 5.32–5.27 (br.s, 1H, NH), 5.31 (d, *J* = 12.5 Hz, 1H, OCH_b_N), 4.69 (dd, *J* = 10.1, 5.1 Hz, 1H, 2-H), 4.18 (t, *J* = 5.0 Hz, 2H, 2′-H_2_), 3.73 (s, 3H, OMe), 3.12–3.04 (m, 1H, 3-H_a_), 3.03–2.93 (m, 1H, 3-H_b_), 2.06 (s, 3H, Ac), 1.44 (s, 9H, Boc). ^13^C RMN (125.7 MHz, CDCl_3_, 26 °C) δ_C_ 190.0 (C, CO), 170.7 (C, CO), 170.6 (C, CO), 170.0 (C, CO), 155.8 (C, CO), 143.4 (CH, 4-C), 137.5 (C, Ph), 133.2 (CH, Ph), 128.8 (2 × CH, Ph), 128.7 (2 × CH, Ph), 128.6 (CH, 5-C), 80.1 (C, C-O, Boc), 70.8 (CH_2_, OCH_2_N), 59.5 (CH, 2-C), 52.9 (CH_3_, OMe), 42.6 (CH_2_, 2′-C), 32.4 (CH_2_, 3-C), 28.4 (3 × CH_3_, Boc), 20.8 (CH_3_, Ac). HRMS (ESI) calculated for C_23_H_30_N_2_O_8_Na [M + Na]^+^ 485.1900 found 485.1898.

***N*-Acetoxymethyl-*N*-(*N*-tert-butoxycarbonyl-L-glycyl)-6-oxohept-4-enoate methyl ester (6).** The same procedure as described for product **5**, but using dimethyl (2-oxoprop-1-yl) phosphonate (55.0 mg, 46 µL, 0.33 mmol) as HWE reagent. After work-up and solvent removal, the residue was purified by column chromatography on silica gel (*n*-hexane:EtOAc, 50:50) affording dipeptide **6** (56.0 mg, 0.14 mmol, 46%) as a yellow oil. ^1^H RMN (400 MHz, CDCl_3_, 26 °C) δ_H_ 6.68 (dt, *J* = 15.6, 6.3 Hz, 1H, 4-H), 6.11 (d, *J* = 15.9 Hz, 1H, 5-H), 5.42–5.23 (m, 3H, OCH_2_N, NH), 4.69 (dd, *J* = 9.3, 5.5 Hz, 1H, 2-H), 4.20–4.12 (m, 2H, 2′-H_2_), 3.71 (s, 3H, OMe), 2.99 (dt, *J* = 15.0, 5.5 Hz, 1H, 3-H_a_), 2.82 (dt, *J* = 15.4, 8.7 Hz, 1H, 3-H_b_), 2.23 (s, 3H, Me), 2.08 (s, 3H, Ac), 1.44 (s, 9H, Boc). ^13^C RMN (100.6 MHz, CDCl_3_, 26 °C) δ_C_ 198.0 (C, CO), 170.8 (C, CO), 170.5 (C, CO), 170.0 (C, CO), 155.8 (C, CO), 142.2 (CH, 4-C), 133.6 (CH, 5-C), 80.1 (C, C-O, Boc), 70.7 (CH_2_, OCH_2_N), 59.2 (CH, 2-C), 52.9 (CH_3_, OMe), 42.5 (CH_2_, 2′-C), 32.4 (CH_2_, 3-C), 28.4 (3 x CH_3_, Boc), 27.6 (CH_3_, Me), 20.8 (CH_3_, Ac). HRMS (ESI) calculated for C_18_H_28_N_2_O_8_Na [M + Na]^+^ 423.1743 found 423.1736.

**Preparation of Fragment 2 protected derivatives**

***N^α^*-(*N*-tert-Butoxycarbonyl-L-prolyl)-*N^ω^*-tosyl-L-arginine methyl ester (7).** A solution of *N^ω^*-tosyl-L-arginine hydrochloride (2.27 g, 6 mmol) in dry methanol (20 mL) was cooled to 0 °C, and SOCl_2_ (857.0 mg, 0.52 mL, 7.2 mmol) was added dropwise. The reaction mixture was left to react overnight (18 h), and then was concentrated under vacuum, affording *N^ω^*-tosyl-L-arginine methyl ester hydrochloride as a white foam (2.60 g). Next, a portion of this product (355.0 mg, 0.9 mmol) was dissolved in dichloromethane (5 mL), treated with *N*-tert-butoxycarbonyl-L-proline (194.0 mg, 0.9 mmol) and cooled to 0 °C, followed by addition of *O*-(benzotriazol-1-yl)-*N*,*N*,*N*′,*N*′- tetramethyluronium hexafluorophosphonate (HBTU, 375 mg, 0.99 mmol) and dropwise injection of *N*,*N*-diisopropylethylamine (DIPEA, 349.0 mg, 0.47 mL, 2.7 mmol). The mixture was stirred at 0 °C for 30 min and then allowed to reach room temperature for 1.5 h; afterward, it was poured into a saturated aqueous NaHCO_3_ solution, and the organic layer was then washed with 5% aqueous HCl, and extracted with dichloromethane. The organic phase was dried and concentrated as usual, and the residue was purified by column chromatography on silica gel (n-hexane:EtOAc, 20:80), yielding the dipeptide **7** (350.5 mg, 0.65 mmol, 72%) as a white foam. ^1^H RMN (400 MHz, CD_3_CN, 70 °C) δ_H_ 7.71 (d, *J* = 8.3 Hz, 2H, Ar), 7.30 (d, *J* = 8.3 Hz, 2H, Ar), 6.95–6.79 (br.s., 1H, NH), 6.19–6.03 (br.s, 2H, 2 × NH), 5.92–5.77 (br.s, 1H, NH), 4.39 (td, *J* = 8.4, 5.1 Hz, 1H, 2-H), 4.17 (dd, *J* = 8.5, 3.7 Hz, 1H, 2′-H), 3.68 (s, 3H, OMe), 3.46–3.34 (m, 2H, 5′-H_2_), 3.17 (q, *J* = 6.5 Hz, 2H, 5-H_2_), 2.40 (s, 3H, Me-Ar), 2.18–2.05 (m, 1H, 3′-H_a_), 2.01–1.96 (m, 1H, 3′-H_b_), 1.90–1.75 (m, 3H, 4′-H_2_, 3-H_a_), 1.71–1.60 (m, 1H, 3-H_b_), 1.59–1.47 (m, 2H, 4-H_2_), 1.43 (s, 9H, Boc). ^13^C NMR (100.6 MHz, CD_3_CN, 70 °C) δ_C_ 174.1 (C, CO), 173.5 (C, CO), 158.5 (C, CO), 143.4 (C, Ar), 143.2 (C, Ar), 130.4 (2 × CH, Ar), 127.0 (2 × CH, Ar), 80.8 (C, C-O, Boc), 61.7 (CH, 2′-C), 53.1 (CH, 2-C), 52.9 (CH_3_, OMe), 48.2 (CH_2_, 5′-C), 41.8 (CH_2_, 5-C), 30.3 (2 × CH_2_, 3-C, 3′-C), 29.0 (3 × CH_3_, Boc), 26.5 (CH_2_, 4-C), 25.0 (CH_2_, 4′-C), 21.6 (CH_3_, Me). One of the CO groups was not detected. HRMS (ESI) calculated for C_24_H_37_N_5_O_7_SNa [M + Na]^+^ 562.2311 found 562.2316.

***N*-(*N*-tert-butoxycarbonyl-L-prolyl)-4*R*-hydroxy-L-proline methyl ester (8).** A solution of *N*-tert-butoxycarbonyl-L-proline (322 mg, 1.5 mmol) and 4*R*-hydroxy-L-proline methyl ester (218 mg, 1.5 mmol) in dichloromethane (4 mL) at 0 °C was treated with HBTU (625.9 mg, 1.65 mmol), and then DIPEA (581.5 mg, 0.78 mL, 4.5 mmol) was injected dropwise. The reaction was stirred at 0 °C for 30 min. and then allowed to reach room temperature for 1.5 h. The reaction mixture was washed with saturated aqueous NaHCO_3_ solution and then with a 5% aqueous HCl solution, and extracted with dichloromethane. The organic phase was dried and concentrated as usual, and the crude was purified by silica gel column chromatography (DCM:MeOH, 98:2), affording the dipeptide **8** (386.5 mg, 1.13 mmol, 75%) as a white foam. This product has been previously reported [42].

**Products from the oxidative radical scission of substrate Boc-Pro-Hyp-OMe (8).**

***N*-Acetoxymethyl-*N*-(*N*-tert-butoxycarbonyl-L-prolyl)-4-oxo-L-homoalanine methyl ester (9).** A solution of dipeptide **8** (171.0 mg, 0.5 mmol) in dry dichloroethane (10 mL) was treated with DIB (322.0 mg, 1 mmol) and iodine (63.5 mg, 0.25 mmol). The mixture was stirred at 80 °C for 45 min, under visible light irradiation. After this time, it was poured onto a 10% aqueous solution of Na_2_S_2_O_3_ and extracted with dichloromethane. The organic layer was dried and concentrated as usual, and the residue was purified by silica gel column chromatography (n-hexane:EtOAc, 50:50), giving the aldehyde **9** (140.1 mg, 0.35 mmol, 70%) as a yellow oil. ^1^H RMN (400 MHz, CD_3_CN, 70 °C) δ_H_ 9.69 (s, 1H, CHO), 5.60–5.39 (m, 2H, OCH_2_N), 4.96 (t, *J* = 6.7 Hz, 1H, 2-H), 4.73 (d, *J* = 6.3 Hz, 1H, 2′-H), 3.67 (s, 3H, OMe), 3.41 (t, *J* = 6.8 Hz, 2H, 5′-H_2_), 3.24 (dd, *J* = 17.9, 6.5 Hz, 1H, 3-H_a_), 2.83 (dd, *J* = 17.9, 7.2 Hz, 1H, 3-H_b_), 2.34–2.15 (m, 1H, 3′-H_a_), 2.04 (s, 3H, Ac), 1.92–1.76 (m, 3H, 3′-H_b_, 4′-H_2_), 1.42 (s, 9H). ^13^C RMN (100.6 MHz, CD_3_CN, 70 °C) δ_C_ 200.4 (CH, CHO), 175.6 (C, CO), 171.5 (2 × C, CO), 155.0 (C, CO), 80.4 (C, C-O, Boc), 73.2 (CH_2_, OCH_2_N), 58.5 (CH, 2′-C), 56.3 (CH, 2-C), 53.3 (CH_3_, OMe), 47.8 (CH_2_, 5′-C), 44.7 (CH_2_, 3-C), 32.0 (CH_2_, 3′-C), 29.0 (3 × CH_3_, Boc), 24.1 (CH_2_, 4′-C), 21.2 (CH_3_, Ac). HRMS (ESI) calculated for C_19_H_32_N_2_O_9_Na [M + MeOH + Na]^+^ 455.2005 found 455.2005.

**Reductive amination products of the aldehyde (9).**

**General procedure for the reductive amination with aldehydes:** A solution of aldehyde **9** (80.0 mg, 0.2 mmol) in dry dichloroethane (3 mL) was treated with a secondary amine (0.26 mmol) and triethylamine (Et_3_N, 28.5 mg, 39 µL, 0.28 mmol). The reaction mixture was allowed to react for 1 h at room temperature. After this time, sodium triacetoxyborohydride (NaBH(OAc)_3_, 67.8 mg, 0.32 mmol) was added and the solution was stirred overnight. Then, it was poured onto a saturated aqueous NaHCO_3_ solution and extracted with dichloromethane. The organic layer was dried and concentrated as usual, and the residue was purified by silica gel column chromatography (n-hexane:EtOAc mixtures), giving the desired triamine or tetraamine derivatives.

***N*-Acetoxymethyl-*N*-(*N*-tert-butoxycarbonyl-L-prolyl)-4-morpholino-L-homoalanine methyl ester (10).** Obtained following the general procedure for reductive amination, using the secondary amine morpholine (22.7 mg, 23 µL, 0.26 mmol). The reaction crude was purified by rotary chromatography (n-hexanes:EtOAc, 10:90), affording the product **10** (80.0 mg, 0.17 mmol, 84%) as a yellow oil.

This product was also obtained from the hydroxyproline derivative **8** (171 mg, 0.5 mmol), following the procedure for the oxidative radical cleavage of the pyrrolidine C_4_-C_5_ bond, followed by reductive amination of the reaction crude containing the aldehyde intermediate. For reductive amination, the amounts of the general procedure were extrapolated, using morpholine (56.8 mg, 0.65 mmol), triethylamine (70.8 mg, 98 µL, 0.7 mmol), and NaBH(AcO)_3_ (67.8 mg, 0.32 mmol) dissolved in dry dichloroethane (7.5 mL). In this way, the dipeptide **10** (146.0 mg, 0.31 mmol, 62%) was obtained. ^1^H RMN (400 MHz, CD_3_CN, 70 °C) δ_H_ 5.64–5.41 (m, 2H, OCH_2_N), 4.96–4.65 (m, 2H, 2-H, 2′-H), 3.66 (s, 3H, OMe), 3.62 (t, *J* = 4.7 Hz, 4H, CH_2_OCH_2_), 3.48–3.35 (m, 2H, 5′-H_2_), 2.48–2.30 (m, 6H, 4-H_2_, CH_2_NCH_2_), 2.25–2.14 (m, 1H, 3′-H_a_), 2.04 (s, 3H, Ac), 2.03–1.99 (m, 2H, 3-H_2_), 1.94–1.81 (m, 3H, 3′-H_b_, 4′-H_2_), 1.42 (s, 9H, Boc). ^1^H RMN (400 MHz, CDCl_3_, 26 °C) 1:1 rotamer mixture. The numbers in italics correspond to one rotamer; the others correspond to the whole set of rotamers δ_H_ [5.61 (d, *J* = 12.3 Hz, *1H*, OCH_a_N, Rot.1), 5.53 (d, *J* = 12.3 Hz, *1H*, OCH_b_N, Rot.1)/5.48 (s, *2H*, OCH_2_N, Rot.2], [5.09 (dd, *J* = 9.8, 5.0 Hz, *1H*, 2-H, Rot.1)/4.84 (dd, *J* = 9.2, 6.2 Hz, *1H*, 2-H, Rot.2)], 4.68 (dd, *J* = 8.5, 3.2 Hz, 1H, 2′-H, Rot.1/2), 3.73–3.64 (m, 7H, OMe, CH_2_OCH_2_), 3.64–3.36 (m, 2H, 5′-H_2_), 2.51–2.33 (m, 6H, 4-H_2_, CH_2_NCH_2_), 2.31–2.21 (m, 1H, 3′-H_b_), 2.08 (s, 3H, OAc), 2.00–1.78 (m, 5H, 3′-H_b_, 3-H_2_, 4′-H_2_), 1.42/1.41 (s/s, 9H, Boc). ^13^C RMN (100.6 MHz, CDCl_3_, 26 °C) rotamer mixture δ_C_ 175.1/174.3 (C, CO), 171.9/171.5 (C, CO), 170.5/170.3 (C, CO), 154.4/153.8 (C, CO), 79.8/79.6 (C, C-O, Boc), 71.2/70.4 (CH_2_, OCH_2_N), 67.2/67.1 (2 × CH_2_, CH_2_OCH_2_), 57.4/56.7 (CH, 2′-C), 57.2/56.0 (CH, 2-C), 55.2/54.8 (CH_2_, 4-C), 53.7 (2 × CH_2_, CH_2_NCH_2_), 52.5/52.3 (CH_3_, OMe), 47.0/46.7 (CH_2_, 5′-C), 31.3/30.3 (CH_2_, 3′-C), 28.52/28.50 (3 × CH_3_, Boc), 26.6/26.3 (CH_2_, 3-C), 24.4/23.2 (CH_2_, 4′-C), 21.1/21.0 (CH_3_, OAc). HRMS (ESI) calculated for C_22_H_37_N_3_O_8_Na [M + Na]^+^ 494.2478 found 494.2482.

***N*-Acetoxymethyl-*N*-(*N*-tert-butoxycarbonyl-L-prolyl)-4-thiomorpholine-L-homoalanine methyl ester (11).** Obtained following the general procedure for reductive amination, using the secondary amine thiomorpholine (27.0 mg, 26 µL, 0.26 mmol). The reaction crude was purified by rotatory chromatography (*n*-hexane:EtOAc, 50:50), giving product **11** (83.8 mg, 0.17 mmol, 86%) as a yellow oil. This product was also obtained from hydroxyproline derivative **8** (171 mg, 0.5 mmol), according to the procedure for the oxidative radical fragmentation of the hydroxyproline C_4_-C_5_ bond, followed by the reductive amination of the resultant scission crude. For reductive amination, the amounts of the general procedure were extrapolated, using thiomorpholine (67.1 mg, 0.65 mmol), triethylamine (70.8 mg, 98 µL, 0.7 mmol), and NaBH(OAc)_3_ (67.8 mg, 0.32 mmol) dissolved in dry dichloroethane (7.5 mL). After work-up and chromatographic purification, the dipeptide **11** (158.9 mg, 0.33 mmol, 65%) was isolated. ^1^H RMN (400 MHz, CD_3_CN, 70 °C) δ_H_ 5.60–5.43 (m, 2H, OCH_2_N), 4.97–4.61 (m, 2H, 2′-H, 2-H), 3.66 (s, 3H, OMe), 3.48–3.36 (m, 2H, 5′-H_2_), 2.76–2.56 (m, 8H, CH_2_NCH_2_, CH_2_SCH_2_), 2.47–2.35 (m, 2H, 4-H_2_), 2.33–2.12 (m, 2H, 3′-H_a_, 3-H_a_), 2.02 (s, 3H, Ac), 1.92–1.80 (m, 4H, 3′-H_b_, 3-H_b_, 4′-H_2_), 1.42 (s, 9H, Boc). ^1^H RMN (400 MHz, CDCl_3_, 26 °C) 1:1 rotamer mixture. The proton numbers in italics refer only to one rotamer; the non-italics numbers refer to the whole set of rotamers. δ_H_ [5.59 (d, *J* = 12.3 Hz, *1H*, OCH_a_N, Rot.1), 5.52 (d, *J* = 12.4 Hz, *1H*, OCH_b_N, Rot.1)/5.47 (s, *2H*, OCH_2_N, Rot.2], [5.09 (dd, *J* = 9.8, 5.0 Hz, *1H*, 2-H, Rot.1)/4.81 (dd, *J* = 9.4, 5.1 Hz, *1H*, 2-H, Rot.2)], 4.68 (dd, *J* = 8.7, 3.2 Hz, 1H, 2′-H, Rot.1/2), 3.69/3.66 (s/s, 3H, OMe), 3.64–3.34 (m, 2H, 5′-H_2_), 2.78–2.55 (m, 8H, CH_2_NCH_2_, CH_2_SCH_2,_), 2.48–2.30 (m, 2H, 4-H_2_), 2.30–2.10 (m, 2H, 3′-H_a_, 3-H_a_), 2.07 (s, 3H, Ac), 2.00–1.78 (m, 5H, 3′-H_b_, 3-H_2_, 4′-H_2_), 1.42/1.41 (s/s, 9H, Boc). ^13^C RMN (100.6 MHz, CDCl_3_, 26 °C) δ_c_ 174.9/174.3 (C, CO), 172.0/171.5 (C, CO), 170.5/170.3 (C, CO), 154.4/153.8 (C, CO), 79.8/79.6 (C, C-O, Boc), 71.2/70.4 (CH_2_, OCH_2_N), 57.3/56.7 (CH, 2′-C), 57.2/55.8 (CH, 2-C), 55.5/54.9 (CH_2_, 4-C), 55.2/55.1 (2 × CH_2_, CH_2_NCH_2_), 52.5/52.3 (CH_3_, OMe), 47.0/46.7 (CH_2_, 5′-C), 31.3/30.3 (CH_2_, 3′-C), 28.54/28.50 (3 × CH_3_, Boc), 28.2/28.1 (2 × CH_2_, CH_2_SCH_2_), 26.8/26.5 (CH_2_, 3-C), 24.4/23.2 (CH_2_, 4′-C), 21.1/21.0 (CH_3_, Ac). HRMS (ESI) calculated for C_22_H_37_N_3_O_7_SNa [M + Na]^+^ 510.2250 found 510.2249.

***N*-Acetoxymethyl-*N*-(*N*-tert-butoxycarbonyl-L-prolyl)-4-(2*S*-methyloxycarbonyl-1-pyrrolidinyl)-L-homoalanine methyl ester (12).** Obtained following the general procedure for reductive amination, using the secondary amine proline methyl ester (34.0 mg, 0.26 mmol). The reaction crude was purified by rotary chromatography (*n*-hexanes:EtOAc, 40:60), obtaining the derivative **12** (78.0 mg, 0.15 mmol, 76%) as a yellow oil. ^1^H RMN (500 MHz, CDCl_3_, 55 °C) 1:1 rotamer mixture. The proton numbers in italics refer only to one rotamer; the non-italics numbers refer to the whole set of rotamers: δ_H_ [5.71 (d, *J* = 12.9 Hz, *1H*, OCH_a_N, Rot.1), 5.59 (d, *J* = 12.5 Hz, *1H*, OCH_b_N, Rot.1)/5.54–5.43 (br.s, *2H*, OCH_2_N, Rot.2], 4.81–4.57 (m, 2H, 2′-H, 2-H), 3.69 (s, 6H, 2 × OMe), 3.66–3.33 (m, 2H, 5′-H_2_), 3.26–3.15 (m, 1H, 2″-H), 3.15–3.08 (m, 1H, 5″-H_a_), 2.75 (dt, *J* = 12.4, 8.1 Hz, 1H, 4-H_a_), 2.54–2.41 (m, 1H, 4-H_b_), 2.41–2.31 (m, 1H, 5″-H_b_), 2.30–2.20 (m, 2H, 3′-H_a_, 3-H_a_), 2.12–1.97 (m, 2H, 3″-H_a_, 3-H_b_), 2.08 (s, 3H, Ac), 1.97–1.75 (m, 6H, 3′-H_b_, 3″-H_b_, 4″-H_2_, 4′-H_2_), 1.44 (s, 9H, Boc). ^13^C RMN (125.7 MHz, CDCl_3_, 55 °C) rotamer mixture δ_C_ 175.1/174.5 (C, CO), 174.6/174.1 (C, CO), 171.7/171.4 (C, CO), 170.5/170.4 (C, CO), 154.4/154.0 (C, CO), 79.8/79.6 (C, C-O, Boc), 72.0/71.3 (CH_2_, OCH_2_N), 66.0/65.8 (CH, 2″-C), 58.3/57.4 (CH, 2-C), 57.6/56.9 (CH, 2′-C), 53.1/52.9 (CH_2_, 5″-C), 52.3 (CH_2_, 4-C), 51.7 (CH_3_, OMe), 51.1 (CH_3_, OMe), 47.1/46.7 (CH_2_, 5′-C), 31.3/30.3 (CH_2_, 3″-C), 29.5 (CH_2_, 3′-C), 28.6 (3 × CH_3_, Boc), 28.2 (CH_2_, 3-C), 24.4/23.3 (CH_2_, 4′-C or 4″-C), 23.3/23.2 (CH_2_, 4′-C or 4″-C), 21.0 (CH_3_, Ac). HRMS (ESI) calculated for C_24_H_39_N_3_O_9_Na [M + Na]^+^ 536.2584 found 536.2579.

**Preparation of the free acids corresponding to Fragment 1.**

***N^α^*-(*N*-tert-butoxycarbonyl-L-glycyl)-*N^δ^*-trityl-L-glutamine (13).** A solution of product **1** (447.5 mg, 0.8 mmol) in tetrahydrofuran (4 mL) at 0 °C was treated dropwise with a solution of 1M KOH (9:1 MeOH:H_2_O) (1.6 mL, 1.6 mmol) and allowed to react for 2 h. Afterward, it was acidified with a 5% HCl solution, poured onto water, and extracted with ethyl acetate. The organic layer was dried and concentrated as usual. Finally, the residue was purified by silica gel column chromatography (dichloromethane: methanol, 9:1), giving the dipeptide **13** (427.5 mg, 0.78 mmol, 98%) as an amorphous solid. ^1^H RMN (400 MHz, CD_3_OD, 26 °C) δ_H_ 7.32–7.14 (m, 15H, Trt), 4.43 (dd, *J* = 9.4, 4.6 Hz, 1H, 2-H), 3.74 (d, *J* = 17.0 Hz, 1H, 2′-H_a_), 3.68 (d, *J* = 16.8 Hz, 1H, 2′-H_b_), 2.54–2.33 (m, 2H, 4-H_2_), 2.24–2.09 (m, 1H, 3-H_a_), 1.94–1.81 (m, 1H, 3-H_b_), 1.43 (s, 9H, Boc). ^13^C RMN (100.6 MHz, CD_3_OD, 26 °C) δ_C_ 174.7 (C, CO), 174.2 (C, CO), 172.5 (C, CO), 158.4 (C, CO), 146.0 (3 × C, Trt), 130.0 (6 × CH, Trt), 128.7 (6 × CH, Trt), 127.8 (3 × CH, Trt), 80.8 (C, C-O, Boc), 71.6 (C, C-N, Trt), 53.0 (CH, 2-C), 44.6 (CH_2_, 2′-C), 33.9 (CH_2_, 4-C), 28.8 (3 x CH_3_, Boc), 28.7(CH_2_, 3-C). HRMS (ESI) calculated for C_31_H_35_N_3_O_6_ [M]^+^ 545.2526 found 545.2535.

***N^α^*-(*N*-tert-butoxycarbonyl-L-glycyl)-*N^γ^*-trityl-L-asparagine (14).** A solution of dipeptide **2** (932.5 mg, 1.71 mmol) in tetrahydrofuran (6 mL) at 0 °C was treated dropwise with a 1M KOH solution (9:1 MeOH:H_2_O) (3.5 mL, 3.42 mmol) and allowed to react for 2 h. It was then acidified with a 5% HCl solution, poured into water, and extracted with ethyl acetate. The organic phase was dried with anhydrous sodium sulfate, filtered, and concentrated. Finally, it was purified by silica gel column chromatography (dichloromethane: methanol, 9:1), affording product **14** (898.9 mg, 1.69 mmol, 99%) as an amorphous solid. ^1^H RMN (400 MHz, CD_3_OD, 26 °C) δ_H_ 7.31–7.15 (m, 15H, Trt), 4.76–4.66 (m, 1H, 2-H), 3.79–3.64 (m, 2H, 2′-H_2_), 2.94 (dd, *J* = 15.7, 6.2 Hz, 1H, 3-H_a_), 2.84 (dd, *J* = 15.7, 5.2 Hz, 1H, 3-H_b_), 1.42 (s, 9H, Boc). ^13^C RMN (100.6 MHz, CD_3_OD, 26 °C) δ_C_ 174.2 (C, CO), 171.9 (C, CO), 171.7 (C, CO), 158.3 (C, CO), 145.8 (3 × C, Trt), 130.0 (6 × CH, Trt), 128.7 (6 × CH, Trt), 127.8 (3 × CH, Trt), 80.8 (C, C-O, Boc), 71.7 (C, C-N, Trt), 50.6 (CH, 2-C), 44.6 (CH_2_, 2′-C), 39.1 (CH_2_, 3-C), 28.7 (3 × CH_3_, Boc). HRMS (ESI) calculated for C_30_H_33_N_3_O_6_Na [M + Na]^+^ 554.2267 found 554.2250.

**Preparation of the first tetrapeptide library: fully protected derivatives.**

***N*-terminal deprotection of (F2) fragments Boc-Pro-XX-OMe**. A solution of the Boc-Pro-XX-OMe derivative (0.4 mmol) in dichloromethane (1.5 mL) at 0 °C was treated dropwise with trifluoroacetic acid (TFA, 0.5 mL). The reaction mixture was allowed to react for 2 h, and then was concentrated under vacuum. To ensure the elimination of traces of volatiles, dichloromethane was added again, and the solvent was removed under reduced pressure. After a couple of washing-concentration cycles, the *N*-deprotected derivative was obtained with high purity.

**General procedure for coupling of dipeptides Boc-Gly-YY(Trt)-OH and H-Pro-XX-OMe [6]**. A Fragment-1 derivative with a deprotected carboxylic acid (0.4 mmol) and a Fragment-2 derivative with a deprotected terminal amino group (0.4 mmol) were used as starting materials. Both substrates were dissolved in dichloromethane (6 mL), and after cooling to 0 °C, HBTU (166.9 mg, 0.44 mmol) and DIPEA (0.14–0.27 mL, 0.8–1.6 mmol) were added dropwise until a basic pH was achieved. The reaction was stirred at 0 °C for 30 min. and then allowed to reach room temperature for 1.5 h. The reaction mixture was washed with saturated aqueous NaHCO_3_ solution and subsequently with 5% aqueous HCl, and extracted with dichloromethane. The organic phase was dried and concentrated as usual, and the crude was purified by silica gel column chromatography, using as eluent either ethyl acetate or dichloromethane:methanol mixtures. Thus, the rigin tetrapeptide derivatives with all their functional groups protected were isolated.

***N*^α^-(*N*-[*N*^α^-(*N*-tert-butoxycarbonyl-L-glycyl)-*N^δ^*-trityl-L-glutaminyl]-L-prolyl)-*N^ω^*-tosyl-L-arginine methyl ester (15).** Obtained from the derivative **13** (218.2 mg, 0.4 mmol) and the dipeptide **7** (215.7 mg, 0.4 mmol), using the general procedure for peptide coupling. The reaction crude was purified by silica gel column chromatography (EtOAc), yielding the tetrapeptide **15** (336.6 mg, 0.35 mmol, 87%) as a white foam. ^1^H RMN (500 MHz, CD_3_OD, 50 °C) rotamer mixture; when the signal is split, the minor one is indicated in italics δ_H_ *7.72*/7.71 ([*d*, *J = 8.1 Hz*/d, *J =* 8.2 Hz], 2H, Tos), 7.29–7.16 (m, 17H, Tos, Trt), 4.60–4.56 (m, 1H, 2″-H), 4.46–4.38 (m, 1H, 2′-H), 4.38–4.31 (m, 1H, 2-H), 3.75–3.66 (m, 3H, 2‴-H_2_), 3.68 (s, 3H, OMe), 3.66–3.58 (m, 1H, 5′-H_a_), 3.48–3.39 (m, 1H, 5′-H_b_), *3.22*–*3.14*/3.09 ([*m*/t, *J* = 7.2 Hz], 2H, 5-H_2_), 2.54 (dt, *J* = 14.8, 7.4 Hz, 1H, 4″-H_a_), 2.46–2.35 (m, 1H, 4″-H_b_), 2.37 (s, 3H, Me-Ar), 2.17–1.74 (m, 7H, 3″-H_2_, 3′-H_2_, 4′-H_2_, 3-H_a_), 1.63–1.53 (m, 1H, 3-H_b_), 1.54–1.46 (m, 2H, 4-H_2_), 1.42 (s, 9H, Boc). ^1^H RMN (400 MHz, CD_3_CN, 70°C) δ_H_ 7.68 (d, *J* = 8.3 Hz, 2H, Tos), 7.56 (br.s, 1H, NH), 7.37–7.19 (m, 17H, Tos, Trt), 7.10 (d, *J* = 7.8 Hz, 1H, NH), 6.95 (d, *J* = 7.0 Hz, 1H, NH), 6.12–5.95 (br.s, 2H, 2 × NH), 5.63–5.46 (br.s, 1H, NH), 4.62 (q, *J* = 7.4 Hz, 1H, 2″-H), 4.44–4.30 (m, 2H, 2′-H, 2-H), 3.70–3.62 (m, 2H, 2‴-H_2_), 3.67 (s, 3H, OMe), 3.62–3.50 (m, 1H, 5′-H_a_), 3.50–3.39 (m, 1H, 5′-H_b_), *3.17*–*3.08*/3.05 ([*m*/td, *J* = 7.9, 6.5 Hz, 2H, 5-H_2_), 2.52–2.41 (m, 1H, 4″-H_a_), 2.42–2.32 (m, 1H, 4″-H_b_), 2.39 (s, 3H, Me-Ar), 2.08–1.85 (m, 5H, 3″-H_a_, 3′-H_2_, 4′-H_2_), 1.83–1.71 (m, 2H, 3″-H_b_, 3-H_a_), 1.62–1.49 (m, 1H, 3-H_b_), 1.47–1.37 (m, 2H, 4-H_2_), 1.42 (s, 3H, Boc). ^13^C RMN (125.7 MHz, CD_3_OD, 50 °C) rotamer mixture δ_C_ 174.20/174.16 (C, CO), 174.1/174.0 (C, CO), 173.64/173.60 (C, CO), 172.5/172.3 (C, CO), 172.2 (C, CO), 158.6 (C, C=N), 158.3 (C, CO), 146.1/146.0 (3 × C, Trt), 143.5 (C, Tos), 142.4 (C, Tos), 130.3 (2 × CH, Tos), 130.04/130.00 (6 × CH, Trt), 128.73/128.70 (6 × CH, Trt), 127.80/127.77 (3 × CH, Trt), 127.1 (2 × CH, Tos), 80.9 (C, C-O, Boc), 71.72/71.68 (C, C-N, Trt), 61.8/61.6 (CH, 2′-C), 53.4/53.1 (CH, 2-C), 52.7 (CH_3_, OMe), 52.1/51.7 (CH, 2″-C), 49.0 (CH_2_, 2‴-C), 44.7 (CH_2_, 5′-C), 41.5 (CH_2_, 5-C), 33.4 (CH_2_, 4″-C), 30.3/30.2 (CH_2_, 3′-C), 29.7/29.6 (CH_2_, 3-C), 28.9 (CH_2_, 3″-C), 28.8/28.7 (3 × CH_3_, Boc), 26.5 (CH_2_, 4-C), 25.9/25.5 (CH_2_, 4′-C), 21.4 (CH_3_, OMe). ^13^C RMN (100.6 MHz, CD_3_CN, 70°C) δ_C_ 173.5 (C, CO), 173.4 (C, CO), 172.7 (C, CO), 172.3 (C, CO), 170.9 (C, CO), 158.5 (C, C=N), 157.3 (C, CO), 146.2 (3 × C, Trt), 143.3 (C, Tos), 143.2 (C, Tos), 130.4 (2 × CH, Tos), 130.0 (6 × CH, Trt), 129.0 (6 × CH, Trt), 128.0 (3 × CH, Trt), 127.0 (2 × CH, Tos), 80.5 (C, C-O, Boc), 71.5 (C, C-N, Trt), 61.4 (CH, 2′-C), 53.01 (CH, 2-C), 53.0 (CH_3_, OMe), 51.5 (CH, 2″-C), 48.6 (CH_2_, 5′-C), 45.3 (CH_2_, 2‴-C), 41.7 (CH_2_, 5-C), 33.8 (CH_2_, 4″-C), 30.1 (CH_2_, 3-C), 29.7 (CH_2_, 3′-C), 29.3 (CH_2_, 3″-C), 29.0 (3 × CH_3_, Boc), 26.2 (CH_2_, 4-C), 26.0 (CH_2_, 4′-C), 21.6 (CH_3_, Me). HRMS (ESI) calculated for C_50_H_63_N_8_O_10_S [M + H]^+^ 967.4388 found 967.4404.

***N^α^*-(*N*-[*N^α^*-(*N*-tert-butoxycarbonyl-L-glycyl)-*N^γ^*-trityl-L-asparaginyl]-L-prolyl)-*N^ω^*-tosyl-L-arginine methyl ester (16).** Obtained from the derivative **14** (212.5 mg, 0.4 mmol) and the dipeptide **7** (215.7 mg, 0.4 mmol), using the general procedure for coupling dipeptides. The reaction crude was purified by silica gel column chromatography (dichloromethane:MeOH, 97:3 (d1) and dichloromethane:MeOH, 96:4 (d2)), providing the tetrapeptide **16** (317.0 mg, 83%). **Product 16**. White foam ^1^H RMN (400 MHz, CD_3_CN, 70 °C) rotamer mixture: δ_H_ 7.70 (d, *J* = 8.4 Hz, 2H, Tos), 7.70–7.65 (br.s, 1H, NH), 7.41 (d, *J* = 7.7 Hz, 1H, NH), 7.32–7.18 (m, 17H, Tos, Trt), 7.07 (d, *J* = 7.5 Hz, 1H, NH), 6.24–6.09 (br.s, 2H, 2 × NH), 6.04–5.90 (br.s, 1H, NH), 5.51–5.40 (br.s, 1H, NH), 4.85–4.72 (m, 1H, 2″-H), 4.42–4.32 (m, 1H, 2′-H), 4.17–4.07 (m, 1H, 2-H), 3.69–3.60 (m, 2H, 2‴-H_2_), 3.65 (s, 3H, OMe), 3.60–3.45 (m, 1H, 5′-H_a_), 3.31–3.21 (br.s, 1H, 5′-H_b_), 3.05–2.95 (m, 2H, 5-H_2_), 2.83–2.64 (m, 2H, 3″-H_2_), 2.38 (s, 3H, Me-Ar), 2.19–1.92 (m, 2H, 3′-H_2_), 1.88–1.75 (m, 2H, 4′-H_2_), 1.72–1.58 (m, 1H, 3-H_a_), 1.52–1.29 (m, 3H, 3-H_b_, 4-H_2_), 1.42 (s, 9H, Boc). ^13^C RMN (100.6 MHz, CD_3_CN, 70 °C) δ_C_ 173.3 (C, CO), 172.8 (C, CO), 171.7 (C, CO), 170.6 (C, CO), 170.5 (C, CO), 158.5 (C, CO), 157.2 (C, C=N), 146.0 (3 × C, Trt), 143.3 (C, Tos), 143.2 (C, Tos), 130.4 (2 × CH, Tos), 130.1 (6 × CH, Trt), 129.0 (6 × CH, Trt), 128.0 (3 × CH, Trt), 127.0 (2 × CH, Tos), 80.5 (C, C-O, Boc), 71.8 (C, C-N, Trt), 61.8 (CH, 2′-C), 53.2 (CH_3_, OMe), 52.8 (CH, 2-C), 49.6 (CH, 2″-C), 48.4 (CH_2_, 5′-C), 45.2 (CH_2_, 2‴-C), 41.7 (CH_2_, 5-C), 40.7 (CH_2_, 3″-C), 30.0 (CH_2_, 3-C), 29.3 (CH_2_, 3′-C), 28.9 (3 × CH_3_, Boc), 26.2 (CH_2_, 4-C), 25.7 (CH_2_, 4′-C), 21.6 (CH_3_, Me). HRMS (ESI) calculated for C_49_H_60_N_8_O_10_SNa [M + Na]^+^ 975.4051 found 975.4059.

***N*-(*N*-[*N^α^*-(*N*-tert-butoxycarbonyl-L-glycyl)-*N^δ^*-trityl-L-glutaminyl]-L-prolyl)-4-hydroxy-L-proline methyl ester (17).** Obtained from the F1 dipeptide **13** (218.0 mg, 0.4 mmol) and the F2 dipeptide **8** (136.8 mg, 0.4 mmol), using the general procedure for F2 deprotection and peptide coupling. The reaction crude was purified by silica gel column chromatography (dichloromethane:methanol, 97:3), affording the tetrapeptide **17** (267.6 mg, 0.35 mmol, 87%) as a white foam. ^1^H NMR (500 MHz, CD_3_OD, 50 °C) rotamer mixture, the minor rotamer signals are barely visible or overlap; therefore, only the signals of the major rotamer are described δ_H_ 7.29–7.16 (m, 15H, Trt), 4.67 (dd, *J* = 8.8, 4.4 Hz, 1H, 2-H), 4.61 (t, *J* = 6.4 Hz, 2″-H), 4.53 (t, *J* = 7.9 Hz, 1H, 2′-H), 4.47 (dq, *J* = 3.9, 3.8 Hz, 1H, 4-H), 3.74 (d, *J* = 4.3 Hz, 5-H_2_), 3.72–3.66 (m, 3H, 5′-H_a_, 2‴-H_2_), 3.65 (s, 3H, OMe), 3.58–3.44 (m, 1H, 5′-H_b_), 2.50 (dt, *J* = 15.0, 7.5 Hz, 1H, 4″-H_a_), 2.40 (dt, *J* = 15.0, 6.8 Hz, 1H, 4″-H_b_), 2.27–2.19 (m, 2H, 3-H_a_, 3′-H_a_), 2.13–1.80 (m, 6H, 3-H_b_, 3′-H_b_, 3″-H_2_, 4′-H_2_), 1.43 (s, 9H, Boc). ^1^H RMN (500 MHz, CD_3_CN, 70 °C) rotamer mixture; the minor rotamer signals are barely visible or overlap; therefore, only the signals of the major rotamer are described δ_H_ 7.47 (s, 1H, NH), 7.36–7.19 (m, 15H, Trt), 6.89 (d, *J* = 7.9 Hz, 1H, NH), 5.54–5.40 (br.s, 1H, NH), 4.67–4.60 (m, 2H, 2-H, 2″-H), 4.48–4.41 (m, 2H, 2′-H, 4-H), 3.72–3.61 (m, 4H, 5-H_2_, 2‴-H_2_), 3.59 (s, 3H, OMe), 3.57–3.50 (m, 1H, 5′-H_a_), 3.48–3.40 (m, 1H, 5′-H_b_), 3.24 (d, *J* = 4.3 Hz, 1H, OH), 2.40 (dt, *J* = 15.1, 7.6 Hz, 1H, 4″-H_a_), 2.31 (dt, *J* = 14.8, 6.7 Hz, 1H, 4″-H_b_), 2.21–2.13 (m, 2H, 3-H_a_, 3′-H_a_), 2.08–1.68 (m, 6H, 3-H_b_, 3′-H_b_ 3″-H_2_, 4′-H_2_), 1.43 (s, 9H, Boc). ^13^C RMN (125.7 MHz, CD_3_OD, 50 °C) rotamer mixture δ_C_ 174.08/174.04 (C, CO), 174.06/174.00 (C, CO), 172.70/172.65 (C, CO), 172.4/172.1 (C, CO), 172.0/171.9 (C, CO), 158.3 (C, CO), 146.1/146.0 (3 × C, Trt), 130.1 (6 × CH, Trt), 128.7 (6 × CH, Trt), 127.8/127.7 (3 × CH, Trt), 80.9 (C, C-O, Boc), 71.70/71.66 (C, C-N, Trt), 71.09/71.07 (CH, 4-C), 59.82/59.77 (CH, 2′-C), 59.4/59.3 (CH, 2-C), 55.8/55.5 (CH_2_, 5-C), 52.7 (CH_3_, OMe), 51.7/51.5 (CH, 2″-C), 49.0 (CH_2_, 5′-C), 44.6 (CH_2_, 2‴-C), 38.2/38.1 (CH_2_, 3-C), 33.2 (CH_2_, 4″-C), 29.20/29.15 (CH_2_, 3′-C), 28.73/28.72 (3 × CH_3_, Boc), 28.6 (CH_2_, 3″-C), 25.8/25.5 (CH_2_, 4′-C). ^13^C RMN (125.7 MHz, CD_3_CN, 70 °C) rotamer mixture δ_C_ 173.8/173.7 (C, CO), 172.9/172.7 (C, CO), 172.1/171.9 (C, CO), 171.2/170.9 (C, CO), 170.8/170.7 (C, CO), 157.3 (C, CO), 146.55/146.50 (3 × C, Trt), 130.1/130.0 (6 × CH, Trt), 128.90/128.86 (6 × CH, Trt), 128.0/127.9 (3 × CH, Trt), 80.5 (C, C-O, Boc), 71.3 (C, C-N, Trt), 71.2 (CH, 4-C), 59.4/59.3 (CH, 2′-C), 59.05/59.01 (CH, 2-C), 56.0/55.9 (CH_2_, 5-C), 52.70/52.68 (CH_3_, OMe), 51.4/51.1 (CH, 2″-C), 48.4/48.3 (CH_2_, 5′-C), 45.3 (CH_2_, 2‴-C), 38.5/38.4 (CH_2_, 3-C), 33.6 (CH_2_, 4″-C), 29.7 (CH_2_, 3′-C), 29.3/29.2 (CH_2_, 3″-C), 28.9 (3 × CH_3_, Boc), 25.8/25.5 (CH_2_, 4′-C). HRMS (ESI) calculated for C_42_H_51_N_5_O_9_Na [M + Na]^+^ 792.3584 found 792.3612.

***N-*Acetoxymethyl*-N*-(*N*-[*N^α^*-(*N*-tert-butoxycarbonyl-L-glycyl)-*N^δ^*-trityl-L-glutaminyl]-L-prolyl)-4-(morpholinyl)-L-homoalanine methyl ester (18).** Obtained from the F1 dipeptide **13** (218.0 mg, 0.4 mmol) and the F2 dipeptide **10** (188.5 mg, 0.4 mmol), using the general procedure for peptide coupling. The reaction crude was purified by silica gel column chromatography (dichloromethane:MeOH, 97:3), giving the tetrapeptide **18** (287.5 mg, 0.33 mmol, 80%) as a white foam. ^1^H RMN (500 MHz, CD_3_OD, 50 °C) rotamer mixture; when the signal of the minor rotamer is clearly differentiated from the bulk, its description is displayed in italics δ_H_ 7.32–7.16 (m, 15H, Trt), 5.61 (d, *J* = 12.5 Hz, 1H, OCH_a_N), 5.56 (d, *J* = 12.5 Hz, 1H, OCH_b_N), 4.93 (dd, *J* = 9.3, 5.3 Hz, 1H, 2-H), 4.88 (dd, *J* = 8.5, 5.4 Hz, 1H, 2′-H), 4.63–4.50 (m, 1H, 2″-H), *3.85*–*3.79*/3.76–3.72 (*m*/m, 4H, CH_2_OCH_2_), 3.73–3.65 (m, 3H, 2‴-H_2_, 5′-H_a_), 3.68 (s, 3H, OMe), 3.59–3.47 (m, 1H, 5′-H_b_), 3.05–2.84 (m, 4H, CH_2_NCH_2_), 2.80 (t, *J* = 7.8 Hz, 2H, 4-H_2_), 2.55–2.23 (m, 4H, 4″-H_2_, 3-H_a_, 3′-H_a_), 2.12–1.75 (m, 6H, 3-H_b_, 3′-H_b_, 4′-H_2_, 3″-H_2_), 2.09 (s, 3H, Ac), *1.45*/1.43 (*s*/s, 9H, Boc). ^1^H RMN (500 MHz, CD_3_CN, 70 °C) δ_H_ 7.50 (s, 1H, NH), 7.35–7.18 (m, 15H, Trt), 6.83 (d, *J* = 7.9 Hz, 1H, NH), 5.60–5.42 (m, 3H, NH, OCH_2_N), 4.93–4.85 (m, 1H, 2-H), 4.81 (dd, *J* = 8.5, 5.6 Hz, 1H, 2′-H), 4.63 (dd, *J* = 12.6, 8.0 Hz, 1H, 2″-H), 3.81–3.57 (m, 7H, 2‴-H_2_, CH_2_OCH_2_, 5′-H_a_), 3.67 (s, 3H, OMe), 3.54–3.46 (m, 1H, 5′-H_b_), 3.03–2.70 (m, 6H, CH_2_SCH_2_, 4-H_2_), 2.36–2.23 (m, 4H, 3-H_a_, 3′-H_a_, 4″-H_2_), 2.11–1.90 (m, 4H, 3-H_b_, 4′-H_2_, 3″-H_a_), 2.05 (s, 3H, Ac), 1.88–1.79 (m, 1H, 3′-H_b_), 1.76–1.64 (m, 1H, 3″-H_b_), 1.43 (s, 9H, Boc). ^13^C RMN (125.7 MHz, CD_3_OD, 50 °C) rotamer mixture δ_C_ 176.3 (C, CO), 174.0 (C, CO), 172.2 (C, CO), 172.1 (C, CO), 171.9 (C, CO), 171.7 (C, CO), 158.4 (C, CO), 146.1/146.0 (C, Trt), 130.03/129.97 (6 × CH, Trt), 128.72/128.69 (6 × CH, Trt), 127.82/127.77 (3 × CH, Trt), 80.9 (C, C-O, Boc), 71.8 (C, C-N, Trt), 71.7 (CH_2_, OCH_2_N), 66.3 (2 × CH_2_, CH_2_OCH_2_), 58.8 (CH, 2′-C), 57.7 (CH, 2-C), 55.8 (CH_2_, 4-C), 54.0 (2 × CH_2_, CH_2_NCH_2_), 53.1 (CH_3_, OMe), 51.7 (CH, 2″-C), 49.0 (CH_2_, 5′-C), 44.7 (CH_2_, 2‴-C), 33.4 (CH_2_, 4″-C), 30.44/30.41 (CH_2_, 3′-C), 28.8 (CH_2_, 3″-C), 28.7 (3 × CH_3_, Boc), 26.1 (CH_2_, 3-C), 26.0 (CH_2_, 4′-C), 20.8 (CH_3_, Ac). ^13^C RMN (125.7 MHz, CD_3_CN, 70 °C) δ_C_ 176.2 (C, CO), 173.1 (C, CO), 172.0 (C, CO), 171.3 (C, CO), 170.7 (C, CO), 157.3 (C, CO), 146.4 (3 × C, Trt), 130.0 (6 × CH, Trt), 128.9 (6 × CH, Trt), 128.0 (3 × CH, Trt), 80.6 (C, C-O, Boc), 71.9 (C, C-N, Trt), 71.4 (CH_2_, OCH_2_N), 66.3 (2 × CH_2_, CH_2_OCH_2_), 58.5 (CH, 2′-C), 57.3 (CH, 2-C), 55.5 (CH_2_, 4-C), 54.1 (2 × CH_2_, CH_2_NCH_2_), 53.3 (CH_3_, OMe), 51.4 (CH, 2″-C), 48.6 (CH_2_, 5′-C), 45.4 (CH_2_, 2‴-C), 33.7 (CH_2_, 4″-C), 30.4 (CH_2_, 3′-C), 29.6 (CH_2_, 3″-C), 28.9 (3 × CH_3_, Boc), 26.2 (CH_2_, 3-C), 26.0 (CH_2_, 4′-C), 21.3 (CH_3_, Ac). A (C) signal corresponding to a carbonyl group was not clearly observed. HRMS (ESI) calculated for C_48_H_63_N_6_O_11_ [M + H]^+^ 899.4555 found 899.4534.

***N-*Acetoxymethyl*-N*-(*N*-[*N^α^*-(*N*-tert-butoxycarbonyl-L-glycyl)-*N^δ^*-trityl-L-glutaminyl]-L-prolyl)-4-(thiomorpholinyl)-L-homoalanine methyl ester (19).** Obtained from the dipeptide **13** (218.0 mg, 0.4 mmol) and the dipeptide **11** (195.0 mg, 0.4 mmol), using the general procedure for peptide coupling. The reaction crude was purified by silica gel column chromatography (dichloromethane:MeOH, 97:3), giving the tetrapeptide **19** (267.0 mg, 0.29 mmol, 73%) as a white foam. ^1^H RMN (500 MHz, CD_3_OD, 50 °C) rotamer mixture δ_H_ 7.31–7.17 (m, 15H, Trt), 5.60 (d, *J* = 12.5 Hz, 1H, OCH_a_N), 5.56 (d, *J* = 12.5 Hz, 1H, OCH_a_N), 4.94–4.86 (m, 2H, 2-H, 2′-H), 4.56 (t, *J* = 6.7 Hz, 1H, 2″-H), 3.77–3.59 (m, 3H, 2‴-H_2_, 5′-H_a_), 3.65 (s, 3H, OMe), 3.52–3.43 (m, 1H, 5′-H_b_), 2.80–2.57 (m, 8H, CH_2_NCH_2_, CH_2_SCH_2_), 2.52–2.17 (m, 6H, 4-H_2_, 4″-H_2_, 3′-H_a_, 3-H_a_), 2.11–1.74 (m, 6H, 3-H_b_, 3′-H_b_, 4′-H_2_, 3″-H_2_), 2.08 (s, 3H, Ac), 1.43 (s, 9H, Boc). ^1^H RMN (500 MHz, CD_3_CN, 70 °C) δ_H_ 7.51 (s, 1H, NH), 7.33–7.17 (m, 15H, Trt), 6.92–6.80 (m, 1H, NH), 5.55 (d, *J* = 15.2 Hz, 1H, OCH_a_N), 5.48 (d, *J* = 15.2 Hz, 1H, OCH_b_N), 4.93–4.86 (m, 1H, 2-H), 4.84 (dd, *J* = 8.5, 5.4 Hz, 1H, 2′-H), 4.68–4.58 (m, 1H, 2″-H), 3.72–3.54 (m, 3H, 2‴-H_2_, 5′-H_a_), 3.63 (br.s, 3H, OMe), 3.51–3.44 (m, 1H, 5′-H_b_), 2.80–2.56 (m, 8H, CH_2_NCH_2_, CH_2_SCH_2_), 2.50–1.69 (m, 8H, 3-H_2_, 3′-H_2_, 3″-H_2_, 4-H_2_, 4′-H_2_, 4″-H_2_), 2.04 (s, 3H, Ac), 1.43 (s, 9H, Boc). ^13^C RMN (125.7 MHz, CD_3_OD, 50 °C) rotamer mixture δ_C_ 176.0/175.9 (C, CO), 174.2/174.0 (C, CO), 172.9/172.6 (C, CO), 172.2/172.1 (C, CO), 172.0/171.9 (C, CO), 171.7/171.5 (C, CO), 158.3 (C, CO), 146.11/146.09 (3 × C, Trt), 130.09/130.05 (6 × CH, Trt), 128.72/128.68 (6 × CH, Trt), 127.80/127.78 (3 × CH, Trt), 80.9 (C, C-O, Boc), 74.0 (C, C-N, Trt), 71.70/71.66 (CH_2_, OCH_2_N), 61.7/61.5 (CH, 2′-C), 58.9/58.7 (CH, 2-C), 56.24/56.20 (2 × CH_2_, CH_2_NCH_2_), 56.2/56.1 (CH_2_, 4-C), 52.8/52.6 (CH_3_, OMe), 52.2/51.6 (CH, 2″-C), 49.0 (CH_2_, 5′-C), 44.7 (CH_2_, 2‴-C), 33.3/33.2 (CH_2_, 4″-C), 30.44/30.38 (CH_2_, 3′-C), 28.8 (CH_2_, 3″-C), 28.7 (3 × CH_3_, Boc), 28.6/28.5 (2 × CH_2_, CH_2_SCH_2_), 27.2 (CH_2_, 3-C), 26.1/26.0 (CH_2_, 4′-C), 20.8/20.7 (CH_3_, Ac). ^13^C NMR (125.7 MHz, CD_3_CN, 70 °C) δ_C_ 175.1 (C, CO), 172.8 (C, CO), 172.3 (C, CO), 171.2 (C, CO), 170.3 (C, CO), 170.2 (C, CO), 157.1 (C, CO), 146.0 (3 × C, Trt), 129.7 (6 × CH, Trt), 128.6 (6 × CH, Trt), 127.6 (3 × CH, Trt), 80.1 (C, C-O, Boc), 71.6 (C, C-N, Trt), 70.7 (CH_2_, OCH_2_N), 58.1 (CH, 2′-C), 57.0 (CH, 2-C), 55.7 (2 × CH_2_, CH_2_NCH_2_), 55.2 (CH_2_, 4-C), 52.8 (CH_3_, OMe), 50.9 (CH, 2″-C), 48.2 (CH_2_, 5′-C), 44.7 (CH_2_, 2‴-C), 32.9 (CH_2_, 4″-C), 30.1 (CH_2_, 3′-C), 29.1 (CH_2_, 3″-C), 28.6 (3 × CH_3_, Boc), 28.3 (2 × CH_2_, CH_2_SCH_2_), 26.6 (CH_2_, 3-C), 25.9 (CH_2_, 4′-C), 21.1 (CH_3_, Ac). HRMS (ESI) calculated for C_48_H_63_N_6_O_10_S [M + H]^+^ 915.4326 found 915.4348.

***N-*Acetoxymethyl*-N*-(*N*-[*N^α^*-(*N*-tert-butoxycarbonyl-L-glycyl)-*N^δ^*-trityl-L-glutaminyl]-L-prolyl)-4-(2*S*-methyloxycarbonyl-1-pyrrolidinyl)-L-homoalanine methyl ester 20).** Obtained from the dipeptide **13** (218.0 mg, 0.4 mmol) and the dipeptide **12** (205.3 mg, 0.4 mmol), using the general procedure for peptide coupling. The reaction crude was purified by silica gel column chromatography (dichloromethane:MeOH, 99:1), affording the tetrapeptide **20** (263.5 mg, 0.28 mmol, 69%) as a white foam. ^1^H RMN (500 MHz, CD_3_OD, 50 °C) rotamer mixture, minor rotamer indicated in italics δ_H_ 7.31–7.15 (m, 15H, Trt), 5.68 (br.d., *J* = 12.8 Hz, 1H, OCH_a_N), 5.53 (d, *J* = 12.9 Hz, 1H, OCH_b_N), 4.94/*4.91*–*4.86* ([dd, *J* = 8.5, 5.0 Hz/m], 1H, 2-H), 4.74–4.67 (m, 1H, 2′-H), 4.66–4.56 (m, 1H, 2″-H), 3.75–3.64 (m, 2H, 2‴-H_2_), 3.67 (s, 3H, OMe), 3.63 (br.s, 3H, OMe), 3.58–3.46 (m, 2H, 5′-H_2_), 3.25–3.16 (m, 1H, 2″″-H), 3.16–3.06 (m, 1H, 5″″-H_a_), 2.77–2.66 (m, 1H, 4-H_a_), 2.53–1.77 (m, 16H, 4-H_b_, 5″″-H_b_, 3-H_2_, 3′-H_2_, 3″-H_2_, 3″″-H_2_, 4′-H_2_, 4″-H_2_, 4″″-H_2_), 2.08 (s, 3H, Ac), *1.44*/1.43 (*s*/s, 9H, Boc). ^13^C RMN (125.7 MHz, CD_3_OD, 50°C) rotamer mixture δ_C_ 176.1/176.0 (C, CO), 175.7/175.6 (C, CO), 174.11/174.06 (C, CO), 172.8/172.7 (C, CO), 172.1 (C, CO), 172.09/172.07 (C, CO), 171.5/171.2 (C, CO), 158.3 (C, CO), 146.2/146.1 (3 × C, Trt), 130.0 (6 × CH, Trt), 128.70/128.68 (6 × CH, Trt), 127.79/127.75 (3 × CH, Trt), 80.9 (C, C-O, Boc), 73.8/72.5 (C, C-N, Trt), 72.4/71.7 (CH_2_, OCH_2_N), 67.02/66.95 (CH, 2″″-C), 60.2/59.0 (CH, 2′-C), 59.2/58.8 (CH, 2-C), 54.1/53.8 (CH_2_, 5″″-C), 53.0/52.8 (CH_2_, 4-C), 52.3/52.1 (2 × CH_3_, 2 × OMe), 51.63/51.55 (CH, 2″-C), 48.7 (CH_2_, 5′-C), 44.7 (CH_2_, 2‴-C), 33.6/33.2 (CH_2_, 4″-C), 30.4 (CH_2_, 3′-C), 30.2 (CH_2_, 3″″-C), 28.9/28.6 (CH_2_, 3″-C), 28.75/28.72 (3 × CH_3_, Boc), 25.9/25.6 (CH_2_, 4″″-C), 24.1/24.0 (CH_2_, 3-C), 23.9 (CH_2_, 4′-C), 20.9/20.8 (CH_3_, Ac). HRMS (ESI) calculated for C_50_H_64_N_6_O_12_Na [M + Na]^+^ 963.4480 found 963.4481.

**Preparation of the second tetrapeptide library: derivatives with a free C-terminal acid. General procedure for the saponification of tetrapeptides**. A solution of the starting tetrapeptide (0.2 mmol) in tetrahydrofuran (4 mL) at 0 °C was treated dropwise with a 1M KOH solution (9:1 MeOH:H_2_O; 0.4 mL, 0.4 mmol) and was stirred for 90 min. It was then acidified with a 5% HCl aqueous solution, poured into water, and extracted with ethyl acetate. The organic phase was dried and concentrated as usual, and the residue was purified by silica gel column chromatography using dichloromethane:methanol mixtures as eluent, providing the tetrapeptide with the deprotected carboxylic acid.

***N^α^-(N-[N^α^-(N-tert-*Butoxycarbonyl-L-glycyl)-*N^γ^-*trityl-L-glutaminyl]-L-prolyl-N^ω^-tosyl-L-arginine (21).** Obtained from the tetrapeptide **15** (194.0 mg, 0.20 mmol) using the general procedure for the saponification of tetrapeptides. The reaction crude was purified by silica gel column chromatography (dichloromethane:methanol, 96:4), giving the derivative **21** (180.9 mg, 0.19 mmol, 95%) as a white foam. ^1^H RMN (500 MHz, CD_3_OD, 50 °C) rotamer mixture; when clearly observed, the minor rotamer signal is indicated in italics δ_H_ *7.72*/7.71 (*d*, *J = 7.5 Hz*/d, *J* = 7.7 Hz, 2H, Tos), 7.29–7.16 (m, 17H, Tos, Trt), 4.63–4.53 (m, 1H, 2″-H), 4.47–4.40/*4.40*–*4.36* (m/*m*, 1H, 2′-H), 4.35–4.26 (m, 1H, 2-H), 3.76–3.57 (m, 3H, 2‴-H_2_, 5′-H_a_), 3.49–3.40 (m, 1H, 5′-H_b_), 3.22–3.04 (m, 2H, 5-H_2_), 2.53 (dt, *J* = 15.1, 7.6 Hz, 1H, 4″-H_a_), 2.47–2.39 (m, 1H, 4″-H_b_), 2.37 (s, 3H, Me-Ar), 2.18–1.78 (m, 8H, 3′-H_2_, 3″-H_2_, 4′-H_2_, 3-H_a_), 1.65–1.47 (m, 3H, 3-H_b_, 4-H_2_), 1.42 (s, 9H, Boc). ^13^C RMN (125.7 MHz, CD_3_OD, 50 °C) rotamer mixture δ_C_ 174.2 (C, CO), 174.0 (C, CO), 173.8 (C, CO), 172.4 (C, CO), 172.2 (C, CO), 158.6 (C, CN), 158.3 (C, CO), 146.02/146.00 (3 × C, Trt), 143.48/143.46 (C, Tos), 142.3 (C, Tos), 130.3 (2 × CH, Tos), 130.0 (6 × CH, Trt), 128.7 (6 × CH, Trt), 127.8 (3 × CH, Trt), 127.1 (2 × CH, Tos), 80.9 (C, C-O, Boc), 71.8/71.7 (C, C-N, Trt), 61.9/61.7 (CH, 2′-C), 53.7 (CH, 2-C), 52.0/51.8 (CH, 2″-C), 48.8 (CH_2_, 5′-C), 44.7 (CH_2_, 2‴-C), 41.7 (CH_2_, 5-C), 33.4 (CH_2_, 4″-C), 30.3 (CH_2_, 3′-C), 30.1 (CH_2_, 3-C), 28.9 (CH_2_, 3″-C), 28.8/28.7 (3 × CH_3_, Boc), 26.5 (CH_2_, 4-C), 25.9/25.5 (CH_2_, 4′-C), 21.4 (CH_3_, Me). HRMS (ESI) calculated for C_49_H_61_N_8_O_10_S [M + H]^+^ 953.4231 found 953.4252.

***N^α^*-(*N*-[*N^α^*-(*N*-tert-butoxycarbonyl-L-glycyl)-*N^γ^*-trityl-L-asparaginyl]-L-prolyl-*N^ω^*-tosyl-L-arginine (22).** Obtained from the tetrapeptide **16** (190.6 mg, 0.20 mmol) using the general procedure for the saponification of tetrapeptides. The reaction crude was purified by silica gel column chromatography (dichloromethane:methanol, 90:10), affording product **22** (170.9 mg, 0.18 mmol, 91%) as a white foam. ^1^H RMN (500 MHz, CD_3_OD, 50 °C) rotamer mixture; when clearly observed, the minor rotamer signal is indicated in italics δ_H_ 7.71 (d, *J* = 7.8 Hz, 2H, Tos), 7.30–7.15 (m, 17H, Trt, Tos), *4.85*/4.79 ([*t*, *J = 7.1 Hz*/t, *J* = 6.9 Hz], 1H, 2″-H), 4.38 (dd, *J* = 8.5, 4.0 Hz, 1H, 2′-H), *4.25*–*4.18*/4.16–4.08 (*m*/m, 1H, 2-H), 3.69/*3.65* (s/*s*, 2H, 2‴-H_2_), 3.58/*3.50*–*3.41* ([q, *J* = 7.6 Hz/*m*] 1H, 5′-H_a_), 3.22–3.10 (m, 1H, 5′-H_b_), 2.98/*2.92*–*2.88* ([t, *J* = 7.1 Hz/*m*], 2H, 5-H_2_), 2.84/*2.76*–*2.79* ([td, *J* = 16.3, 6.7 Hz/*m*], 2H, 3″-H_2_), *2.38*/2.36 (*s*/s, 3H, Me-Ar), 2.13–2.02 (m, 1H, 3′-H_a_), 2.00–1.89 (m, 1H, 3′-H_b_), 1.88–1.74 (m, 2H, 4′-H_2_), 1.73–1.64 (m, 1H, 3-H_a_), 1.48–1.34 (m, 3H, 3-H_b_, 4-H_2_), 1.42 (s, 9H, Boc). ^13^C RMN (125.7 MHz, CD_3_OD, 50 °C) rotamer mixture δ_C_ 173.8 (C, CO), 172.2 (2 × C, CO), 172.0 (C, CO), 171.1 (C, CO), 158.6 (C, CO), 158.2 (C, C=N), 145.84/145.80 (3 × C, Trt), 143.4 (C, Tos), 142.5 (C, Tos), 130.3 (2 × CH, Tos), 130.1/130.0 (6 × CH, Trt), 128.8/128.7 (6 × CH, Trt), 127.8 (3 × CH, Trt), 127.1 (2 × CH, Tos), 80.9 (C, C-O, Boc), 71.9 (C, C-N, Trt), 62.2/61.7 (CH, 2′-C), 54.6 (CH, 2-C), 49.8 (CH_2_, 2″-C), 48.6 (CH, 5′-C), 44.6 (CH_2_, 2‴-C), 41.7/41.2 (CH_2_, 5-C), 39.7 (CH_2_, 3″-C), 30.4 (CH_2_, 3′-C), 29.7 (CH_2_, 3-C), 28.7 (3 × CH_3_, Boc), 26.6 (CH_2_, 4-C), 25.7 (CH_2_, 4′-C), 21.4 (CH_3_, Me). HRMS (ESI) calculated for C_48_H_58_N_8_O_10_S [M]^+^ 938.3997 found 938.3996.

***N*-(*N*-[*N^α^*-(*N*-tert-Butoxycarbonyl-L-glycyl)-*N^δ^*-trityl-L-glutaminyl]-L-prolyl-*N*-4-hydroxy-L-proline (23).** Obtained from the tetrapeptide **17** (153.8 mg, 0.2 mmol) using the general procedure for the saponification of tetrapeptides. The reaction crude was purified by silica gel column chromatography (dichloromethane:methanol, 96:4), affording product **23** (142.0 mg, 0.19 mmol, 94%) as a white foam. ^1^H RMN (500 MHz, CD_3_OD, 50 °C) rotamer mixture, the minor rotamer signals are barely visible or overlap; therefore, only the signals of the major rotamer are described δ_H_ 7.29–7.16 (m, 15H, Trt), 4.66 (dd, *J* = 8.5, 4.4 Hz, 2-H), 4.64–4.57 (m, 1H, 2″-H), 4.54–4.44 (m, 2H, 2′-H, 4-H), 3.79–3.46 (m, 6H, 5-H_2_, 2‴-H_2_, 5′-H_2_), 2.50 (dt, *J* = 15.0, 7.5 Hz, 1H, 4″-H_a_), 2.40 (dt, *J* = 14.5, 6.9 Hz, 1H, 4″-H_b_), 2.33–2.16 (m, 2H, 3-H_a_, 3′-H_a_), 2.14–1.82 (m, 6H, 3-H_b_, 3′-H_b_, 3″-H_2_, 4′-H_2_), 1.43 (s, 9H, Boc). ^13^C RMN (125.7 MHz, CD_3_OD, 50 °C) δ_C_ 174.0 (2 × C, CO), 172.4 (C, CO), 172.1 (C, CO), 171.9 (C, CO), 158.3 (C, CO), 146.1 (3 × C, Trt), 130.1 (6 × CH, Trt), 128.7 (6 × CH, Trt), 127.7 (3 × CH, Trt), 80.9 (C, C-O, Boc), 71.7 (C, C-N, Trt), 71.1 (CH, 4-C), 60.3 (CH, 2′-C), 59.8 (CH, 2-C), 55.7 (CH_2_, 5-C), 51.7/51.5 (CH, 2″-C), 49.0 (CH_2_, 5′-C), 44.6 (CH_2_, 2‴-C), 38.6 (CH_2_, 3-C), 33.2 (CH_2_, 4″-C), 29.2/29.1 (CH_2_, 3′-C), 28.8/28.7 (3 × CH_3_, Boc), 28.6 (CH_2_, 3″-C), 25.8 (CH_2_, 4′-C). HRMS (ESI) calculated for C_41_H_49_N_5_O_9_Na [M + Na]^+^ 778.3428 found 778.3248.

***N*-(*N*-[*N^α^*-(*N*-tert-butoxycarbonyl-L-glycyl)-*N^δ^*-trityl-L-glutaminyl]-L-prolyl-4-morpholinyl-L-homoalanine (24).** Obtained from the tetrapeptide **18** (179.7 mg, 0.20 mmol) using the general procedure for the saponification of tetrapeptides. The reaction crude was purified by silica gel column chromatography (dichloromethane:methanol, 96:4), affording product **24** (160.9 mg, 0.19 mmol, 99%) as a white foam. ^1^H RMN (500 MHz, CD_3_OD, 50 °C) rotamer mixture; when clearly observed, the minor rotamer signal is indicated in italics δ_H_ 7.32–7.15 (m, 15H, Trt), 4.63–4.55 (m, 1H, 2″-H), 4.39 (dd, *J* = 8.3, 5.2 Hz, 1H, 2′-H), 4.27 (t, *J* = 6.3 Hz, 1H, 2-H), *3.91*–*3.84*/3.80–3.75 (*m*/m, 4H, CH_2_OCH_2_), 3.72 (d, *J* = 16.7 Hz, 1H, 2‴-H_a_), 3.70–3.65 (m, 1H, 5′-H_a_), 3.67 (d, *J* = 16.9 Hz, 2‴-H_b_), 3.54–3.46 (m, 1H, 5′-H_b_), 3.20–3.01 (m, 6H, CH_2_NCH_2_, 4-H_2_), 2.47 (t, *J* = 7.8 Hz, 2H, 4″-H_2_), 2.30–2.13 (m, 2H, 3-H_a_, 3′-H_a_), 2.11–1.78 (m, 6H, 3-H_b_, 3′-H_b_, 3″-H_2_, 4′-H_2_), *1.45*/1.43 (*s*/s, 9H, Boc). ^13^C RMN (125.7 MHz, CD_3_OD, 50 °C) rotamer mixture δ_C_ 174.22 (C, CO), 174.19 (C, CO), 172.3 (C, CO), 172.2 (C, CO), 158.4 (C, CO), 146.0 (3 × C, Trt), 130.1/130.0 (6 × CH, Trt), 128.74/128.69 (6 × CH, Trt), 127.84/127.77 (3 × CH, Trt), 80.9 (C, C-O, Boc), 71.7 (C, C-N, Trt), 65.6/65.4 (2 × CH_2_, CH_2_OCH_2_), 61.9 (CH, 2′-C), 56.0/55.9 (CH_2_, 2-C), 53.5 (CH_2_, 4-C), 53.4/53.3 (2 × CH_2_, CH_2_NCH_2_), 51.8 (CH, 2″-C), 49.0 (CH_2_, 5′-C), 44.7 (CH_2_, 2‴-C), 33.4/33.1 (CH_2_, 4″-C), 30.6/30.4 (CH_2_, 3′-C), 29.1 (CH_2_, 3″-C), 28.8/28.7 (3 × CH_3_, Boc), 28.0 (CH_2_, 3-C), 26.0/25.7 (CH_2_, 4′-C). A (C) signal corresponding to a carbonyl group was not clearly observed. HRMS (ESI) calculated for C_44_H_57_N_6_O_9_^+^ [M + H]^+^ 813.4187 found 813.4222.

***N*-(*N*-[*N^α^*-(*N*-tert-butoxycarbonyl-L-glycyl)-*N^δ^*-trityl-L-glutaminyl]-L-prolyl-4-thiomorpholinyl-L-homoalanine (25).** Obtained from the tetrapeptide **19** (183.0 mg, 0.20 mmol) using the general procedure for the saponification of tetrapeptides. The reaction crude was purified by silica gel column chromatography (dichloromethane:methanol, 94:6), giving product **25** (150.9 mg, 0.18 mmol, 91%) as a white foam. ^1^H RMN (500 MHz, CD_3_OD, 50 °C) rotamer mixture; when clearly observed, the minor rotamer signal is indicated in italics δ_H_ 7.31–7.18 (m, 15H, Trt), 4.63–4.58 (m, 1H, 2″-H), 4.38/4.37–4.33 ([dd, *J* = 8.4, 5.1 Hz]/*m*, 1H, 2′-H), 4.20 (t, *J* = 6.2 Hz, 1H, 2-H), 3.72 (d, *J* = 17.5 Hz, 2‴-H_a_), 3.67 (d, *J* = 16.8 Hz, 2‴-H_b_), 3.64–3.44 (m, 2H, 5′-H_2_), 3.30–3.17 (m, 4H, CH_2_NCH_2_), 3.11–2.96 (m, 2H, 4-H_2_), *2.89*/2.54–2.32 ([*t*, *J = 5.3 Hz*]/m, 4H, CH_2_SCH_2_), 2.46 (dd, *J* = 6.7 Hz, 2H, 4″-H_2_), 2.27–2.11 (m, 2H, 3-H_a_, 3′-H_a_), 2.11–1.79 (m, 7H, 3-H_b_, 3′-H_b_, 3″-H_2_, 4′-H_2_), 1.45/1.43 (s/s, 9H, Boc). ^13^C RMN (125.7 MHz, CD_3_OD, 50 °C) δ_C_ 176.2 (C, CO), 174.2 (C, CO), 174.0 (C, CO), 172.3 (C, CO), 172.2 (C, CO), 158.3 (C, CO), 146.0 (C, Trt), 130.1 (6 × CH, Trt), 128.7 (6 × CH, Trt), 127.8 (3 × CH, Trt), 80.9 (C, C-O, Boc), 71.7 (C, C-N, Trt), 62.1/62.0 (CH, 2′-C), 56.5 (CH_2_, 4-C), 55.5/55.3 (2 × CH_2_, CH_2_NCH_2_), 55.3/54.2 (CH, 2-C), 51.9/51.8 (CH, 2″-C), 49.0 (CH_2_, 5′-C), 44.7 (CH_2_, 2‴-C), 33.4 (CH_2_, 4″-C), 30.4 (CH_2_, 3′-C), 29.1 (CH_2_, 3″-C), 28.8/28.7 (3 × CH_3_, Boc), 28.6/28.5 (CH_2_, 3-C), 26.6/26.5 (2 × CH_2_, CH_2_SCH_2_), 26.0/25.7 (CH_2_, 4′-C). HRMS (ESI) calculated for C_44_H_57_N_6_O_8_S^+^ [M + H]^+^ 829.3959 found 829.3956.

**Preparation of the third tetrapeptide library: unprotected derivatives.**

**General acid-promoted deprotection procedure:** Trifluoroacetic acid (TFA, 0.33 mL) was added dropwise to a solution of the tetrapeptide (0.04 mmol) in dichloromethane (1 mL) cooled to 0 °C. The reaction mixture was allowed to reach room temperature (2 h) and then the solvent was removed under vacuum. Water was added and impurities were removed by extraction with dichloromethane. The aqueous layer was concentrated, affording the unprotected tetrapeptides.

***N^α^*-(*N*-[*N^α^*-(L-glycyl)-L-glutaminyl]-L-prolyl)-*N^ω^*-tosyl-L-arginine (26).** Obtained from the tetrapeptide precursor **21** (38 mg, 0.04 mmol) using the general acid-promoted deprotection. The product was isolated as a white foam (28 mg, 99%). ^1^H RMN (500 MHz, D_2_O, 80 °C) δ_H_ 8.30 (d, *J* = 8.2 Hz, 2H, Tos), 7.95 (d, *J* = 8.4 Hz, 2H, Tos), 5.27 (dd, *J* = 8.7, 5.2 Hz, 1H, 2″-H), 4.99 (dd, *J* = 8.7, 5.4 Hz, 1H, 2′-H), 4.85 (dd, *J* = 8.7, 5.2 Hz, 1H, 2-H), 4.44 (d, *J* = 16.2 Hz, 1H, 2‴-H_a_), 4.41 (d, *J* = 16.5 Hz, 1H, 2‴-H_b_), 4.37–4.30 (m, 1H, 5′-H_a_), 4.27–4.19 (m, 1H, 5′-H_b_), 3.75 (t, *J* = 6.9 Hz, 2H, 5-H_2_), 2.94 (s, 3H, Me-Ar), 2.95–2.76 (m, 3H, 4″-H_2_, 3′-H_a_), 2.73–2.44 (m, 5H, 3″-H_2_, 3′-H_b_, 4′-H_2_), 2.39–2.27 (m, 1H, 3-H_a_), 2.25–2.04 (m, 3H, 3-H_b_, 4-H_2_). ^13^C RMN (125.7 MHz, D_2_O, 80 °C) δ_C_ 178.2 (C, CO), 175.3 (C, CO), 174.3 (C, CO), 171.7 (C, CO), 167.3 (C, CO), 157.7 (C, C=N), 144.6 (C, Tos), 139.3 (C, Tos), 130.3 (2 × CH, Tos), 126.4 (2 × CH, Tos), 61.2 (CH, 2′-C), 52.9 (CH, 2-C), 51.7 (CH, 2″-C), 48.5 (CH_2_, 5′-C), 41.2 (CH_2_, 2‴-C), 41.1 (CH_2_, 5-C), 31.3 (CH_2_, 4″-C), 29.7 (CH_2_, 3′-C), 28.4 (CH_2_, 3-C), 27.1 (CH_2_, 3″-C), 25.3 (CH_2_, 4-C), 25.1 (CH_2_, 4′-C), 21.1 (CH_3_, Me). HRMS (ESI) calculated for C_25_H_39_N_8_O_8_S [M + H]^+^ 611.2612 found 611.2614.

***N^α^*-(*N*-[*N^α^*-(L-glycyl)-L-asparaginyl]-L-prolyl)-*N^ω^*-tosyl-L-arginine (27).** Obtained from the tetrapeptide precursor **22** (37.6 mg, 0.04 mmol) using the general acid-promoted deprotection. The product was isolated as a white foam (28 mg, 99%). ^1^H RMN (500 MHz, D_2_O, 70 °C) δ_H_ 8.20 (d, *J* = 6.6 Hz, 2H, Tos), 7.84 (d, *J* = 7.3 Hz, 2H, Tos), 5.52–5.45 (m, 1H, 2″-H), 4.93–4.86 (m, 1H, 2′-H), 4.75–4.67 (m, 1H, 2-H), 4.34–4.26 (m, 2H, 2‴-H_2_), 4.27–4.21 (m, 1H, 5′-H_a_), 4.21–4.14 (m, 1H, 5′-H_b_), 3.65 (t, *J* = 6.9 Hz, 2H, 5-H_2_), 3.29 (dd, *J* = 15.9, 6.2 Hz, 1H, 3″-H_a_), 3.11 (dd, *J* = 15.5, 7.5 Hz, 1H, 3″-H_b_), 2.84 (s, 3H, Me-Ar), 2.76–2.65 (m, 1H, 3′-H_a_), 2.51–2.35 (m, 3H, 3′-H_b_, 4′-H_2_), 2.30–2.17 (m, 1H, 3-H_a_), 2.15–2.04 (m, 1H, 3-H_b_), 2.04–1.92 (m, 2H, 4-H_2_). ^13^C RMN (125.7 MHz, D_2_O, 80 °C) δ_C_ 175.1 (C, CO), 174.4 (C, CO), 174.2 (C, CO), 171.0 (C, CO), 167.1 (C, CO), 157.4 (C, C=N), 144.6 (C, Tos), 138.9 (C, Tos), 130.2 (2 x CH, Tos), 126.3 (2 x CH, Tos), 61.2 (CH, 2′-C), 52.7 (CH, 2-C), 49.2 (CH, 2″-C), 48.4 (CH_2_, 5′-C), 41.00 (CH_2_, 2‴-C), 40.97 (CH_2_, 5-C), 36.7 (CH_2_, 3″-C), 29.7 (CH_2_, 3′-C), 28.1 (CH_2_, 3-C), 25.3 (CH_2_, 4-C), 24.9 (CH_2_, 4′-C), 21.0 (CH_3_, Me). HRMS (ESI) calculated for C_24_H_36_N_8_O_8_S (M)^+^ 596.2377 found 596.2359.

***N^α^*-(*N*-[*N^α^*-(L-glycyl)-L-glutaminyl]-L-prolyl)-4-hydroxy-L-proline (28).** Obtained from the tetrapeptide precursor **23** (30.4 mg, 0.04 mmol) using the general acid-promoted deprotection. The product was isolated as a white foam (21 mg, 99%). ^1^H RMN (400 MHz, D_2_O, 80 °C) δ_H_ 4.70–4.65 (m, 1H, 2′-H), 4.62 (dd, *J* = 8.9, 5.1 Hz, 1H, 2″-H), 4.58–4.52 (m, 1H, 4-H), 4.46 (t, *J* = 8.4 Hz, 1H, 2-H), 3.83–3.69 (m, 5H, 5′-H_a_, 5-H_2_, 2‴-H_2_), 3.63–3.54 (m, 1H, 5′-H_b_), 2.41–2.21 (m, 4H, 4″-H_2_, 3′-H_a_, 3-H_a_), 2.14–1.93 (m, 4H, 3-H_b_, 4′-H_2_, 3″-H_a_), 1.92–1.77 (m, 2H, 3′-H_b_, 3″-H_b_). ^13^C RMN (100.6 MHz, D_2_O, 80 °C) δ_C_ 177.8 (C, CO), 175.4 (C, CO), 172.3 (C, CO), 171.0 (C, CO), 166.9 (C, CO), 69.9 (CH, 4-C), 58.9 (CH, 2′-C), 58.2 (CH, 2-C), 54.7 (CH_2_, 5-C), 51.0 (CH, 2″-C), 47.9 (CH_2_, 5′-C), 40.2 (CH_2_, 2‴-C), 36.5 (CH_2_, 3-C), 30.5 (CH_2_, 4″-C), 27.7 (CH_2_, 3′-C), 26.3 (CH_2_, 3″-C), 24.6 (CH_2_, 4′-C). HRMS (ESI) calculated for C_17_H_26_N_5_O_7_ (M − H)^+^ 412.1832 found 412.1829.

***N*-(*N*-[*N^α^*-(L-glycyl)-L-glutaminyl]-L-prolyl)-4-morpholinyl-L-homoalanine (29).** Obtained from the tetrapeptide precursor **24** (32.5 mg, 0.04 mmol) using the general acid-promoted deprotection. The product was isolated as a white foam (23 mg, 99%). ^1^H RMN (500 MHz, D_2_O, 80 °C) δ_H_ 5.28 (dd, *J* = 9.2, 5.3 Hz, 1H, 2″-H), 5.01 (dd, *J* = 8.3, 5.4 Hz, 1H, 2′-H), 4.93 (t, *J* = 6.8 Hz, 1H, 2-H), 4.64–4.47 (m, 4H, CH_2_OCH_2_), 4.46 (d, *J* = 16.0 Hz, 1H, 2‴-H_a_), 4.42 (d, *J* = 17.0 Hz, 1H, 2‴-H_b_), 4.41–4.34 (m, 1H, 5′-H_a_), 4.30–4.23 (m, 1H, 5′-H_b_), 4.04–3.75 (m, 6H, CH_2_NCH_2_, 4-H_2_), 3.00–2.83 (m, 4H, 4″-H_2_, 3-H_a_, 3′-H_a_), 2.75–2.48 (m, 6H, 3-H_b_, 3′-H_b_, 4′-H_2_, 3″-H_2_). ^13^C RMN (125.7 MHz, D_2_O, 80 °C) rotamer mixture δ 178.2 (C, CO), 175.6 (C, CO), 174.2 (C, CO), 171.8 (C, CO), 167.4 (C, CO), 64.4/64.3 (2 x CH_2_, CH_2_OCH_2_), 61.6/61.5 (CH, 2′-C), 55.0 (2 x CH_2_, CH_2_NCH_2_), 52.5 (CH_2_ + CH, 4-C, 2-C), 51.8/51.7 (CH, 2″-C), 48.6/48.5 (CH_2_, 5′-C), 41.3/41.1 (CH_2_, 2‴-C), 32.0/31.4 (CH_2_, 4″-C), 30.0/29.8 (CH_2_, 3′-C), 27.7/27.2 (CH_2_, 3-C), 26.8 (CH_2_, 3″-C), 25.2 (CH_2_, 4′-C). HRMS (ESI) for C_20_H_33_N_6_O_7_ (M − H)^+^ 469.2411 found 469.2426.

***N*-(*N*-[*N^α^*-(L-glycyl)-L-glutaminyl]-L-prolyl)-4-thiomorpholinyl-L-homoalanine (30).** Obtained from the tetrapeptide precursor **25** (33.0 mg, 0.04 mmol) using the general acid-promoted deprotection. The product was isolated as a colorless oil (24 mg, 99%). ^1^H RMN (400 MHz, D_2_O, 80 °C) δ_H_ 4.69 (dd, *J* = 9.2, 4.9 Hz, 1H, 2″-H), 4.53 (dd, *J* = 9.4, 4.9 Hz, 1H, 2-H), 4.43 (dd, *J* = 8.4, 5.9 Hz, 1H, 2′-H), 3.90–3.75 (m, 5H, CH_a_NCH_a_, 5′-H_a_, 2‴-H_2_), 3.75–3.64 (m, 1H, 5′-H_b_), 3.35–3.21 (m, 4H, CH_b_NCH_b_, 4-H_2_), 3.07 (t, *J* = 14.8 Hz, 2H, CH_a_SCH_a_), 2.89 (br.d, *J* = 15.4 Hz, 2H, CH_b_SCH_b_), 2.50–2.26 (m, 4H, 4″-H_2_, 3′-H_a_, 3-H_a_), 2.23–1.88 (m, 6H, 3-H_b_, 3′-H_b_, 4′-H_2_, 3″-H_2_). ^13^C RMN (100.6 MHz, D_2_O, 80 °C) rotamer mixture δ_c_ 177.7 (C, CO), 174.2 (C, CO), 173.4 (C, CO), 171.3/171.1 (C, CO), 166.9/166.7 (C, CO), 60.7/60.6 (CH_2_, 2′-C), 54.2 (2 × CH_2_, CH_2_NCH_2_), 53.8/53.7 (CH_2_, 4-C), 51.0/50.8 (CH, 2″-C), 50.5/49.9 (CH, 2-C), 48.0/47.9 (CH_2_, 5′-C), 40.3/40.2 (CH_2_, 2‴-C), 30.6 (CH_2_, 4″-C), 29.3/29.2 (CH_2_, 3′-C), 26.3/26.2 (CH_2_, 3″-C), 25.3/25.1 (CH_2_, 3-C), 24.7 (2 × CH_2_, CH_2_SCH_2_), 24.4 (CH_2_, 4′-C). HRMS (ESI) calculated for C_20_H_33_N_6_O_6_S (M − H)^+^ 485.2182 found 485.2195.

### 3.2. Biological Screenings

***Antifungal activity test: Radial growth in Potato-Dextrose-Agar (PDA) assay*** [43,44]. An initial 20.5 mM solution of each compound in EtOH or in EtOH:DMSO mixtures was prepared, and then an aliquot was taken (0.125 mL, 2.56 µmol) and homogenized with sterile PDA medium (5 mL), so that the final compound concentration was 500 µM (2.56 µmol in 5.125 mL of total media). The solutions were poured onto Petri dishes, and the medium was allowed to solidify.

For the most active compounds, a final concentration of 100 µM was also tested. In that case, a smaller aliquot of the 20.5 mM solution was taken (0.025 mL) and mixed with the same amount of PDA culture (5 mL) before pouring the mixture into the Petri plates.

Once the medium solidified, 4.5 mm diameter discs of the fungus were placed on the top. The assay was performed using 8 replicates on the same plate, which were then incubated at 23 ± 2 °C for 24–72 h.

On the other hand, two untreated control dishes (vehicle only, 5 mL of PDA culture medium) were prepared, and taken as a reference for 100% fungal growth. After the incubation period, the plates were scanned and fungal growth was measured with the Image J 1.53k program. The percentage of inhibition (% I) was calculated as % I = (C − T/C) × 100, where C is the diameter of the control colonies and T is the diameter of the test colonies.

The fungal pathogens were *Alternaria alternate* (strain Aa 100), *Botrytis cinerea* (strain B05.10), and *Fusarium oxysporum f. sp. lycopersici* (CECT 2715). The two first strains were provided by Universidad de La Laguna, Tenerife (www.ull.es), accessed 18 February 2025, and were isolated from *Vitis vinifera* and *Lycopersicon esculentum*, respectively. The *F. oxysporum* strain was supplied by the Spanish Collection of Microorganisms/Colección Española de Cultivos Tipo (CECT, www.uv.es/uvweb/coleccion-espanola-cultivos-tipo/es/cect/catalogo-cepas/medios-cultivo/buscador-cepas-1285892802374.html), accessed 18 February 2025.

### 3.3. In Silico ADME Studies

The in silico ADME studies were performed with the SwissADME tool, developed by the Swiss Institute of Bioinformatics (doi: 10.1038/srep42717) as mentioned in the text and the references.

## 4. Conclusions

This work showed that libraries of host-defense peptides containing both proteinogenic and unnatural residues with unusual *N*-substituents and α-lateral chains can be readily prepared using both our selective peptide modification with “customizable units” and a combinatorial approach. The tetrapeptide rigin was used as a proof of concept of this strategy. Smaller peptide fragments A and B were modified through the scission of hydroxyproline units followed by modification of the resultant α-chains, e.g., HWE or reductive amination. Then, the different fragments were combined to give a pool of tetrapeptides with a variety of substituents. Surprisingly, the evaluation of the antifungal activity of rigin analogs showed that a fully protected tetrapeptide and not the deprotected rigin analogs gave the best results. Thus, compound **18**, containing a *C*-terminal *N*-alkyl substituent and a morpholino group, was the most potent against *Botrytis cinerea* and also showed activity against other fungi. The *N*-alkyl group was removable, allowing comparison with deprotected derivatives, which displayed low activity. Thus, in a short time, valuable structure–activity relationships could be determined.

In addition, in silico ADME studies were performed, which predicted the low risk of undesirable interactions with key biological targets. However, the oral bioavailability was predicted to be low, as happens for other peptide drug candidates, and should be addressed with appropriate formulations.

In summary, an efficient synthesis of structurally diverse peptide analogs of antimicrobial rigin was developed, and several small analogs of host-defense peptides were identified as antifungal candidates. Unexpectedly, the *N*-substituted peptides were more potent than the unprotected ones, which shows the utility of these libraries in identifying new leads.

## Figures and Tables

**Table 1:** Evaluation of antifungal activities against *A. alternata*, *B. cinerea*, and *F. oxysporum*.

Compound	C (µM)	Radial Growth Inhibition (%)		
		*A. alternata (Fr.) * Keissl.	*B. cinerea * Pers.	*F. oxysporum* Schltdl.
		µ ± DE	µ ± DE	µ ± DE
**2**	500	8.63 ± 6.81	38.80 ± 4.00	13.48 ± 2.98
**3**	500	14.98 ± 4.77	30.67 ± 4.61	21.09 ± 6.28
**5**	500	21.15 ± 3.89	25.04 ± 3.47	**42.66 ± 2.87**
**6**	500	NI	31.22 ± 2.94	5.07 ± 3.01
**7**	500	NI	17.45 ± 6.64	3.47 ± 2.27
**8**	500	NI	20.20 ± 4.64	4.67 ± 2.62
**10**	500	NI	7.10 ± 5.08	7.61 ± 5.87
**11**	500	NI	32.14 ± 6.52	
**12**	500	NI	19.93 ± 3.02	6.15 ± 2.18
**13**	500	12.37 ± 4.04	17.11 ± 2.08	11.42 ± 2.77
**14**	500	NI	24.08 ± 3.71	12.33 ± 4.25
**18**	500	**41.29 ± 7.65**	**79.51 ± 4.06**	24.68 ± 4.57
	100	-	11.89 ± 5.62	
**21**	500	NI	NI	8.86 ± 1.84
**22**	500	NI	-	-
**23**	500	NI	10.29 ± 4.74	4.84 ± 4.30
**25**	500	9.45 ± 6.18	**43.00 ± 4.63**	11.59 ± 4.51
**26**	500	3.20 ± 1.77	12.65 ± 4.78	20.19 ± 3.09
**27**	500	NI	15.85 ± 2.34	8.75 ± 3.23
**28**	500	NI	10.60 ± 3.12	14.94 ± 2.99
**29**	500	NI	8.97 ± 6.42	NI
**30**	500	10.45 ± 6.00	15.64 ± 1.59	21.94 ± 4.38

Most relevants results are in bold, and for inhibition > 50% is highlighted in red. NI: no inhibition.

**Table 2:** Predicted properties for dipeptide **5**, protected tetrapeptides **18** and **19**, acid **25**, and deprotected tetrapeptide **30**.

Properties	**5**	**18**	**19**	**25**	**30**
MW (g/mol)	462.49	899.04	915,11	829,02	486,59
Nº rotatable bonds	16	28	28	24	15
Nº H-bond acceptors	8	12	11	9	8
Nº H-bond donors	1	3	3	5	5
TPSA (Å^2^) Topological Polar Surface	128.31	202.22	218.29	211.78	213.46
LogP_o/w_	2.28	3.45	3.96	3.14	−2.33
LogS (ESOL)	−3.36 Soluble	−6.37 Poorly soluble	−6.98 Poorly soluble	−4.98 Moderately soluble	1.96 Highly soluble
GI absorp	High	Low	Low	Low	Low
BBB permeant	No	No	No	No	No
P-gp substrate	Yes	Yes	Yes	Yes	No
CYP inhibitor	No, except isoforms CYP2C19 and CYP2D6	No	No	No	No
Druglikeness (Lipinski)	Yes, 0 violation	No; 2 violations: MW > 500, Number O > 10	No; 2 violations: MW > 500, Number O > 10	No; 2 violations: MW > 500, Number O > 10	Yes; 1 violation: Number O > 10
Abbot Bioavailability score	0.55	0.17	0.17	0.17	0.55
PAINS Alerts	0 alerts	0 alerts	0 alerts	0 alerts	0 alerts
Brenk Alerts	Michael acceptor	+2 esters	+2 esters	0 alerts	0 alerts

## Data Availability

The original contributions presented in this study are included in the article/Supplementary Material. Further inquiries can be directed to the corresponding author.

## Supplementary Materials

The following supporting information can be downloaded at: https://www.mdpi.com/article/10.3390/ijms26051900/s1.

## References

[1] WHO (2022). WHO Fungal Priority Pathogens List to Guide Research, Development and Public Health Action.

[2] FAO Tackling Antimicrobial Resistance in Food and Agriculture. For Free Download. https://openknowledge.fao.org/handle/20.500.14283/cc9185en.

[3] Sáenz V., Alvarez-Moreno C., Le Pape P., Restrepo S., Guarro J., Ramírez A.M.C. (2020). A One Health perspective to recognize *Fusarium* as important in clinical practice. J. Fungi.

[4] Gerardon-Batista B., Antunes de Chaves M., Reginatto P., Saraiva O.J., Meneghello-Fuentefria A. (2020). Human fusariosis: An emerging infection that is difficult to treat. J. Braz. Soc. Trop. Med..

[5] Boto A., Pérez de la Lastra J.M., González C.C. (2018). The Road from Host-Defense Peptides to a New Generation of Antimicrobial Drugs. Molecules.

[6] Mazurkiewicz-Pisarek A., Baran J., Ciach T. (2023). Antimicrobial Peptides: Challenging Journey to the Pharmaceutical, Biomedical, and Cosmeceutical Use. Int. J. Mol. Sci..

[7] Lobo F., Boto A. (2022). Host-Defense Peptides as New Generation Phytosanitaries: Low Toxicity and Low Induction of Antimicrobial Resistance. Agronomy.

[8] Rothstein D.M., Spacciapoli P., Tran L.T., Xu T., Roberts F.D., Dalla Serra M., Buxton D.K., Oppenheim F.G., Friden P. (2001). Anticandida Activity Is Retained in P-113, a 12-Amino-Acid Fragment of Histatin 5. Antimicrob. Agents Chemother..

[9] Seo M.D., Won H.S., Kim J.H., Mishig-Ochir T., Lee B.J. (2012). Antimicrobial Peptides for Therapeutic Applications: A Review. Molecules.

[10] Bojsen R., Torbersen R., Larsen C.E., Folkesson A., Regenberg B. (2013). The synthetic amphipathic peptidomimetic LTX109 is a potent fungicide that disturbs plasma membrane integrity in a sphingolipid dependent manner. PLoS ONE.

[11] Boto A., González C.C., Hernández D., Romero-Estudillo I., Saavedra C.J. (2021). Site-selective modification of peptide backbones. Org. Chem. Front..

[12] Liu R., Li X., Lam K.S. (2017). Combinatorial chemistry in drug discovery. Curr. Opin. Chem. Biol..

[13] Yoshida M. (2019). Combinatorial Synthesis and Biological Evaluation of Destruxins. Chem. Pharm. Bull..

[14] Gerry C.J., Schreiber S.L. (2020). Recent achievements and current trajectories of diversity-oriented synthesis. Curr. Opin. Chem. Biol..

[15] Hernandez D., Porras M., Boto A. (2023). Conversion of Hydroxyproline “Doubly Customizable Units” to Hexahydropyrimidines: Access to Conformationally Constrained Peptides. J. Org. Chem..

[16] Hernandez D., Carro C., Boto A. (2022). Structural Diversity by Using Amino Acid “Customizable Units:” Conversion of Hydroxyproline (Hyp) into Nitrogen Heterocycles. Amino Acids.

[17] Romero-Estudillo I., Boto A. (2015). Domino Process Achieves Site-Selective Peptide Modification with High Optical Purity. Applications to Chain Diversification and Peptide Ligation. J. Org. Chem..

[18] Kumar N., Kishore R. (2010). Determination of an Unusual Secondary Structural Element in the Immuno-Stimulating Tetrapeptide Rigin in Aqueous Environments: Insights via MD Simulations, 1HNMR and CD Studies. J. Pept. Sci..

[19] Veretennikova N.I., Chipens G.I., Nikiforovich G.V., Betinsh Y.R. (1981). Rigin, Another Phagocytosis-Stimulating Tetrapeptide Isolated from Human IgG: Confirmations of a Hypothesis. Int. J. Pept. Protein Res..

[20] Chipen G. (1988). New Biologically Active Fragments of Immunoglobulins. Adv. Drug Deliv. Rev..

[21] Dutta R.C., Puri A., Anand N. (2001). Immunomodulatory Potential of Hydrophobic Analogs of Rigin and Their Role in Providing Protection against Plasmodium berghei Infection in Mice. Int. Immunopharmacol..

[22] Rocchi R., Biondi L., Cavaggion F., Filira F., Gobbo M., Dagan S., Fridkin M. (1987). Synthesis and Biological Activity of Tuftsin and Rigin Derivatives Containing Monosaccharides or Monosaccharide Derivatives. Int. J. Pept. Protein Res..

[23] Labrière C., Kondori N., Caous J.S., Boomgaren M., Sandholm K., Ekdahl K.N., Hansen J.H., Svenson J. (2018). Development and evaluation of cationic amphiphilic antimicrobial 2,5-diketopiperazines. J. Pep. Sci..

[24] Svendsen J.S.M., Grant T.M., Rennison D., Brimble M.A., Svenson J. (2019). Very Short and Stable Lactoferricin-Derived Antimicrobial Peptides: Design Principles and Potential Uses. Acc. Chem. Res..

[25] Saavedra C.J., Carro C., Hernández D., Boto A. (2019). Conversion of “Customizable Units” into *N*-Alkyl Amino Acids and Generation of *N*-Alkyl Peptides. J. Org. Chem..

[26] Svenson J., Molchanova N., Schroeder C.I. (2022). Antimicrobial Peptide Mimics for Clinical Use: Does Size Matter?. Front. Immunol..

[27] Foti C., Piperno A., Scala A., Giuffrè O. (2021). Oxazolidinone Antibiotics: Chemical, Biological and Analytical Aspects. Molecules.

[28] Daina A., Michielin O., Zoete V. (2017). SwissADME: A free web tool to evaluate pharmacokinetics, druglikeness and medicinal chemistry friendliness of small molecules. Sci. Rep..

[29] Daina A., Zoete V.A. (2016). BOILED-Egg to predict Gastrointestinal Absorption and Brain Penetration of Small Molecules. ChemMedChem.

[30] Ertl P., Rohde B., Selzer P. (2000). Fast calculation of molecular polar surface area as a sum of fragment-based contributions and its application to the prediction of drug transport properties. J. Med. Chem..

[31] Daina A., Michielin O., Zoete V. (2014). iLOGP: A Simple, Robust, and Efficient Description of n-Octanol/Water Partition Coefficient for Drug Design Using the GB/SA Approach. J. Chem. Inf. Model..

[32] Lipinski C.A., Lombardo F., Dominy B.W., Feeney P.J. (2001). Experimental and computational approaches to estimate solubility and permeability in drug discovery and development settings. Adv Drug Deliv Rev.

[33] P-Glycoprotein Inhibitors, Drug Bank. https://go.drugbank.com/categories/DBCAT002667.

[34] Cytochrome P-450 Enzyme Inhibitors, Drug Bank. https://go.drugbank.com/categories/DBCAT000394.

[35] Martin Y.C. (2005). A bioavailability score. J. Med. Chem..

[36] Baell J.B., Holloway G.A. (2010). New substructure filters for removal of pan assay interference compounds (PAINS) from screening libraries and for their exclusion in bioassays. J. Med. Chem..

[37] Brenk R., Schipani A., James D., Krasowski A., Gilbert I.H., Frearson J., Wyatt P.G. (2008). Lessons learnt from assembling screening libraries for drug discovery for neglected diseases. ChemMedChem.

[38] Luo D., Chen Q.Y., Luesch H. (2016). Total Synthesis of the Potent Marine-Derived Elastase Inhibitor Lyngbyastatin 7 and in Vitro Biological Evaluation in Model Systems for Pulmonary Diseases. J. Org. Chem..

[39] Huang Z., Zhang M., Burton S.D., Katsakhyan L.N., Ji H. (2014). Targeting the Tcf4 G13ANDE17 Binding Site to Selectively Disrupt β-Catenin/T-Cell Factor Protein–Protein Interactions. ACS Chem. Biol..

[40] Petermichl M., Loscher S., Schobert R. (2016). Total Synthesis of Aurantoside G, an *N*-β-Glycosylated 3-Oligoenoyltetramic Acid from Theonella Swinhoei. Angew. Chem. Int. Ed. Engl..

[41] Kolyasnikova K.N., Vichuzhanin M.V., Konstantinopol’skii M.A., Trofimov S.S., Gudasheva T.A. (2012). Synthesis and pharmacological activity of analogs of the endogenous neuropeptide cycloprolylglycine. Pharm Chem J.

[42] Rump E.T., Rijkers D.T.S., Hilbers H.W., de Groot P.G., Liskamp R.M.J. (2002). Cyclotriveratrylene (CTV) as a New Chiral Triacid Scaffold Capable of Inducing Triple Helix Formation of Collagen Peptides Containing Either a Native Sequence or Pro-Hyp-Gly Repeats. Chem. Eur. J..

[43] PDA method: Li X., Ji M., Qi Z., Li X., Shen Y., Gu Z., Zhang Y., Wei S., Wang Y., Wang D. (2011). Synthesis of 2-amino-6- oxocyclohexenyl- Sulfonamides and Their Activity against Botrytis cinerea. Pest Manag. Sci..

[44] Reyes C.P., Rodríguez-Sabina S., López-Cabeza R., Montelongo C.G., Giménez C., Jiménez I.A., Cabrera R., Bazzocchi I.L. (2022). Antifungal Potential of Canarian Plant Extracts against High-Risk Phytopathogens. Plants.

