# Supplementary material for: Antifungal Peptides with Unexpected Structure from a Library of Synthetic Analogs of Host-Defense Peptide Rigin
Source: Int J Mol Sci. 2025 Feb 22;26(5):1900. doi: 10.3390/ijms26051900 (PMC11900302; doi:10.3390/ijms26051900)
Supplement: Supplementary file 1 [file ijms-26-01900-s001.zip › ijms-3454459-supplementary.pdf]

Supporting Information

Antifungal Peptides with Unexpected Structure from a Library of Synthetic Analogs of Host-Defense Peptide Rigin

Marina Porras, Dácil Hernández* and Alicia Boto*

Instituto de Productos Naturales y Agrobiología del CSIC, Avda. Astrofísico Fco. Sánchez, 3; 38206-La Laguna, Tenerife, Spain; mporras@ipna.csic.es

* Correspondence: dacil@ipna.csic.es (D.H.); alicia@ipna.csic.es (A.B.)

Table of contents

NMR data for fragments A and B new dipeptides **1-3**, **5-7**, **9-14**.	Page 02
NMR data for fully protected tetrapeptides **15-20** and their acids **21-25**	Page 16
NMR for deprotected rigin analogs **26-30**	Page 40

Reproductions of NMR Spectra

Compound **1**, ^{1}H and ^{13}C NMR at 26°C in $CDCl_3$

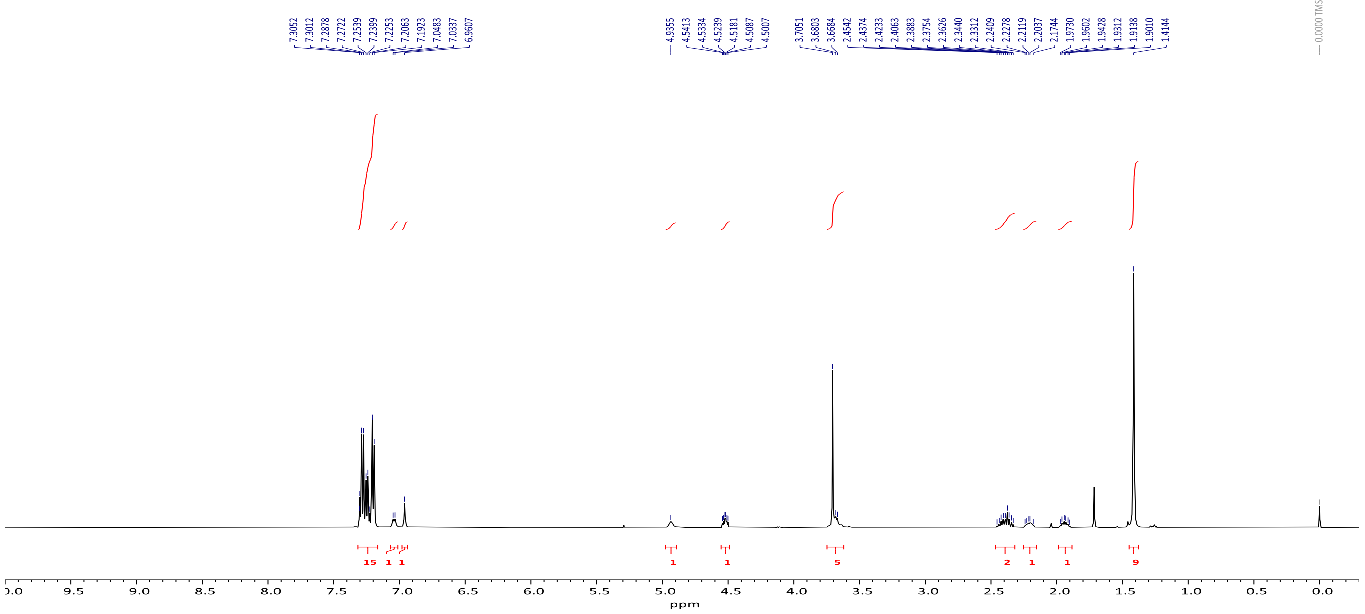

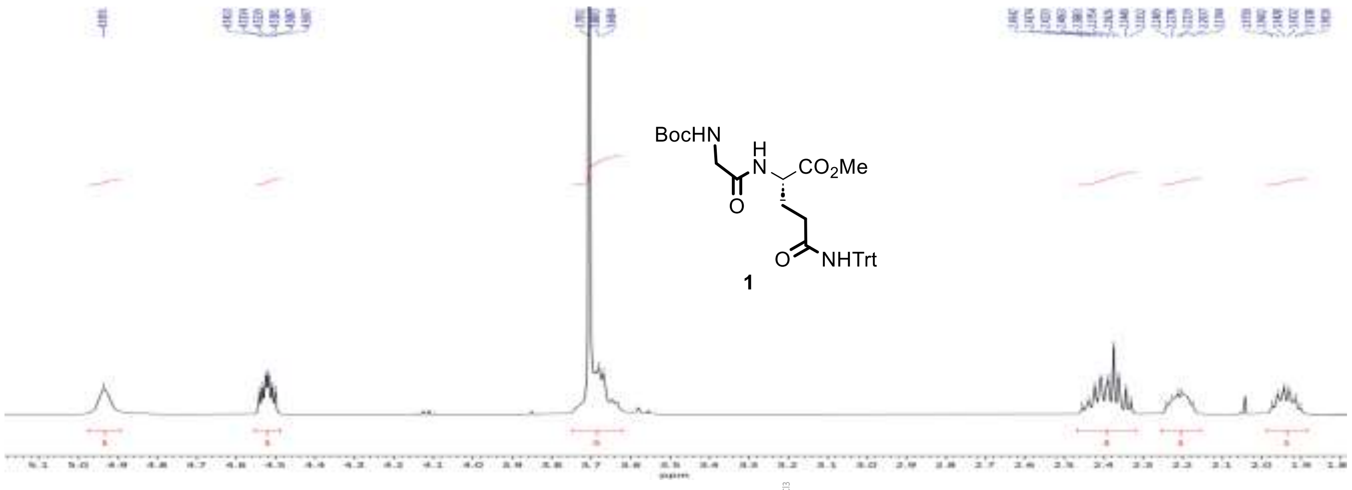

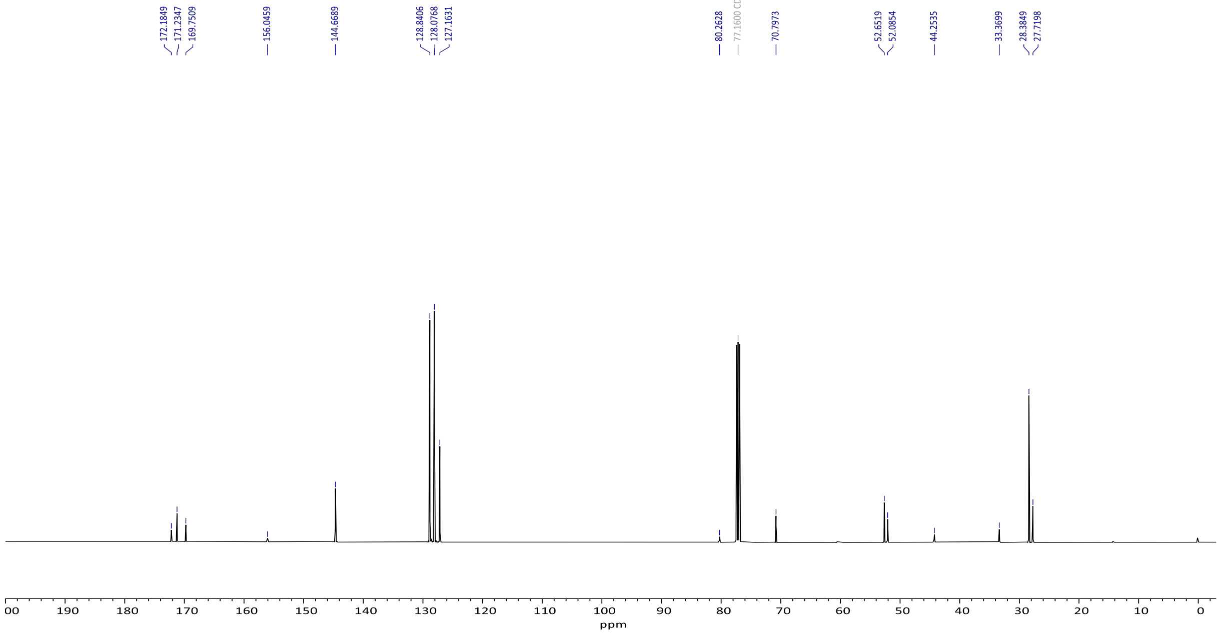

Compound **2**, ^{1}H and ^{13}C NMR at 26°C in $CDCl_3$

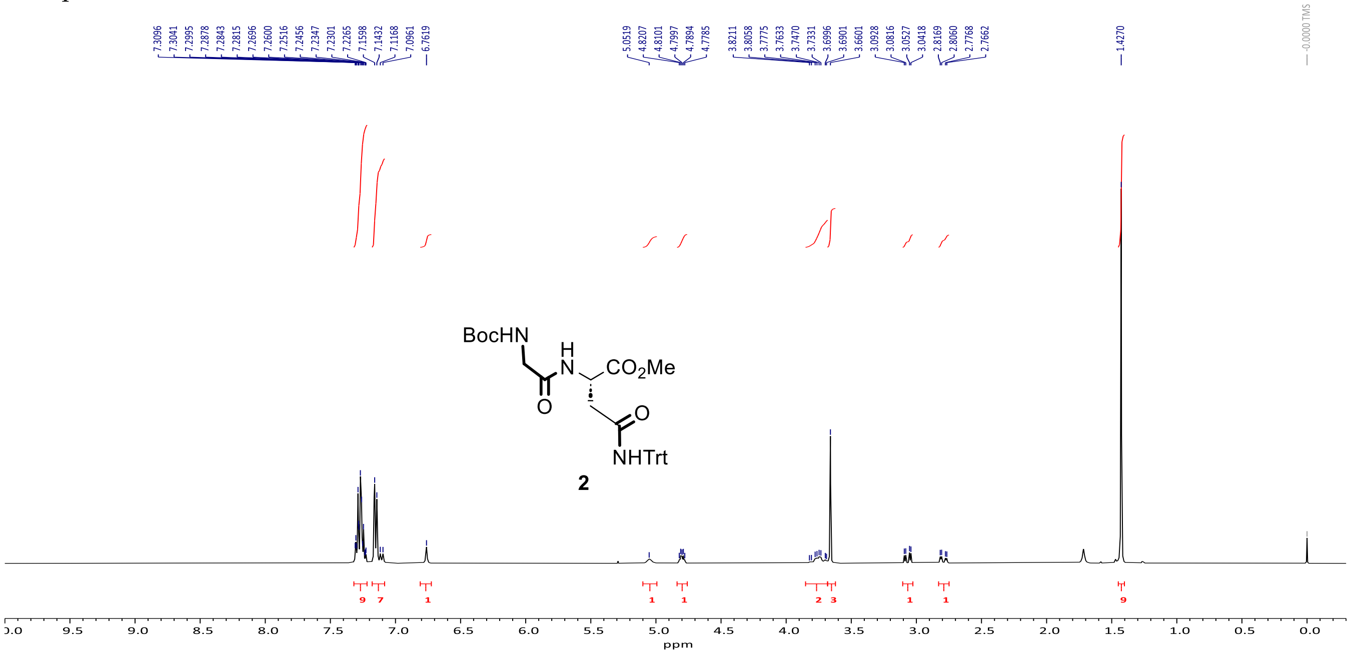

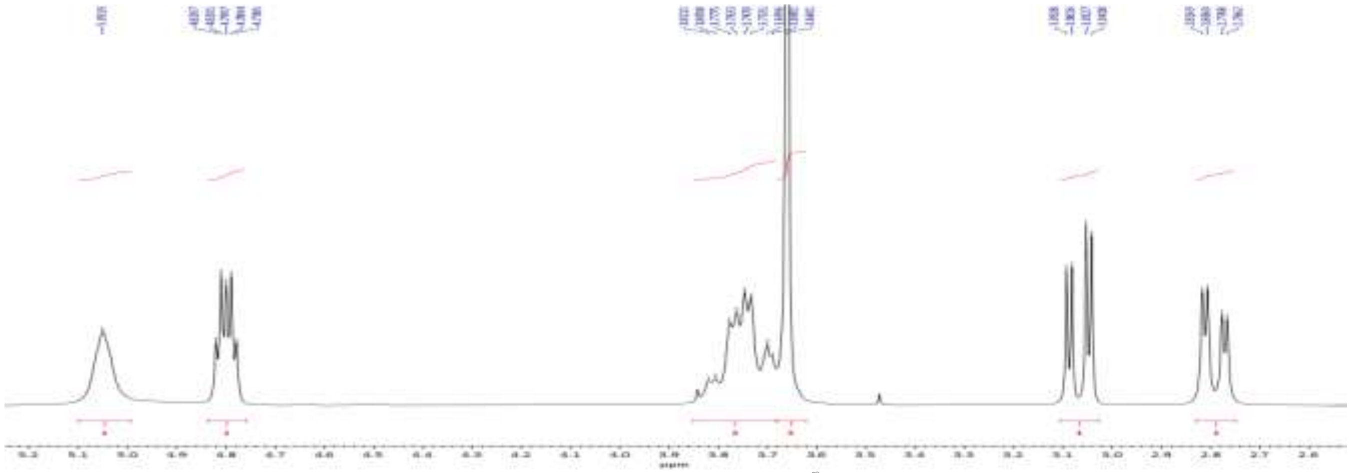

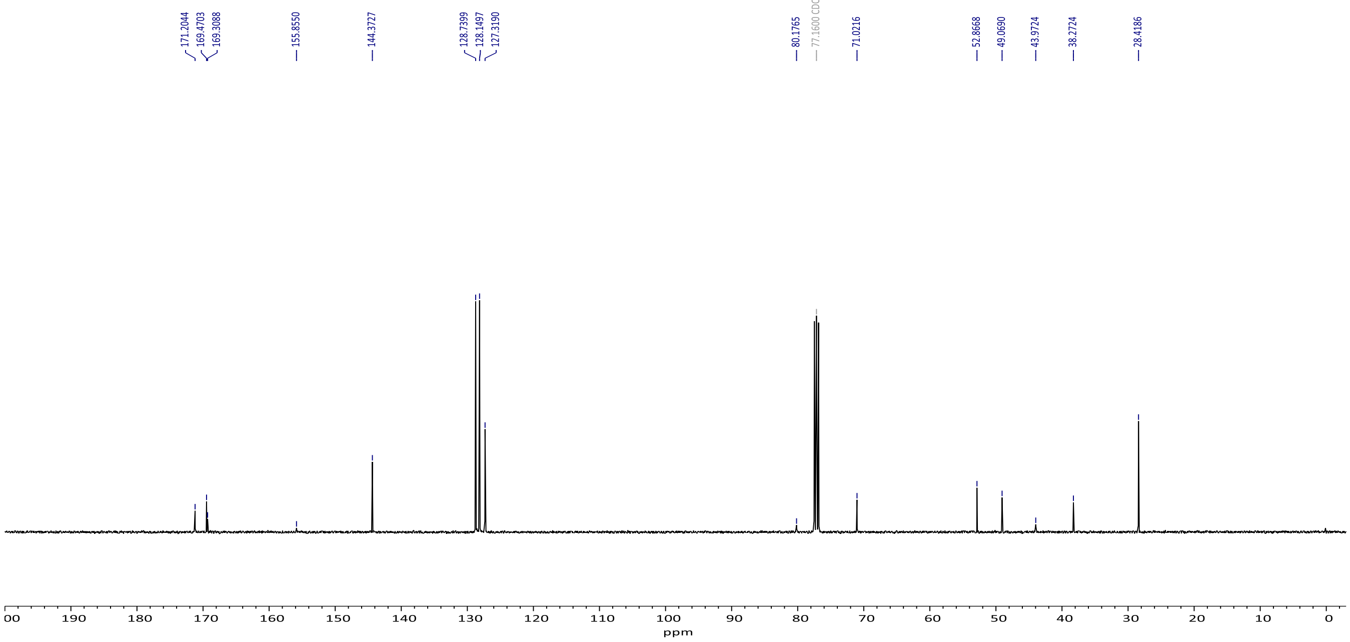

Compound **3**, ^{1}H and ^{13}C NMR at 70°C in CD_3CN

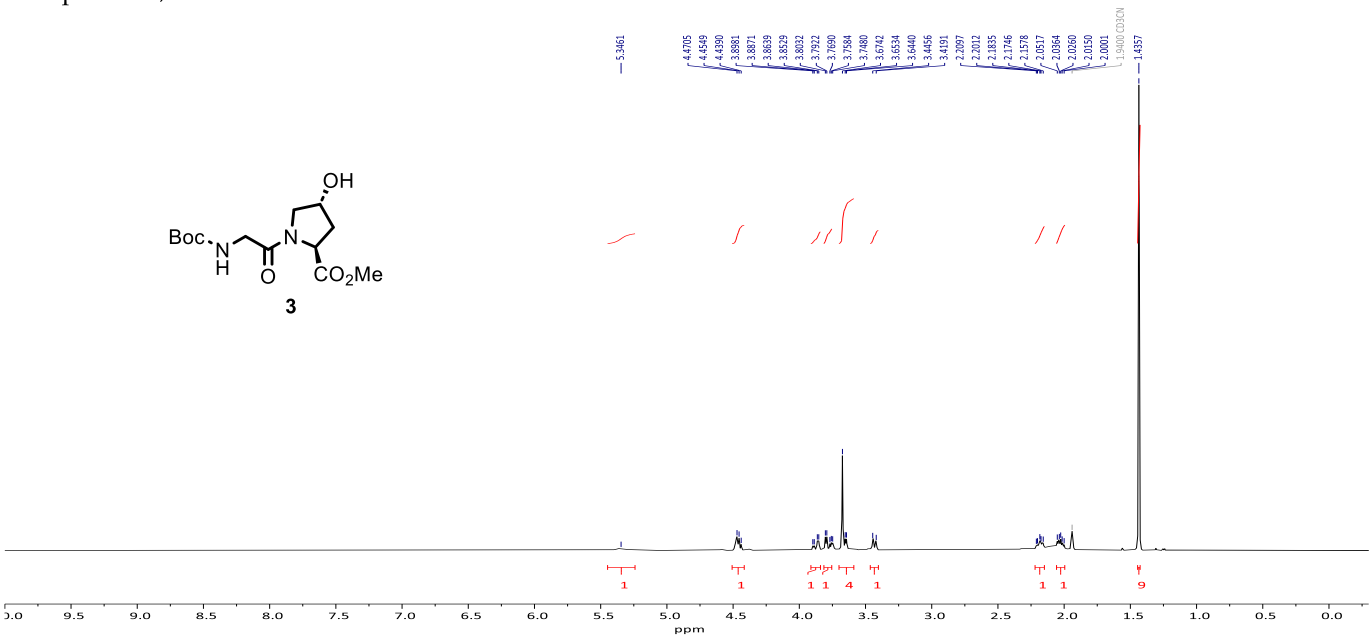

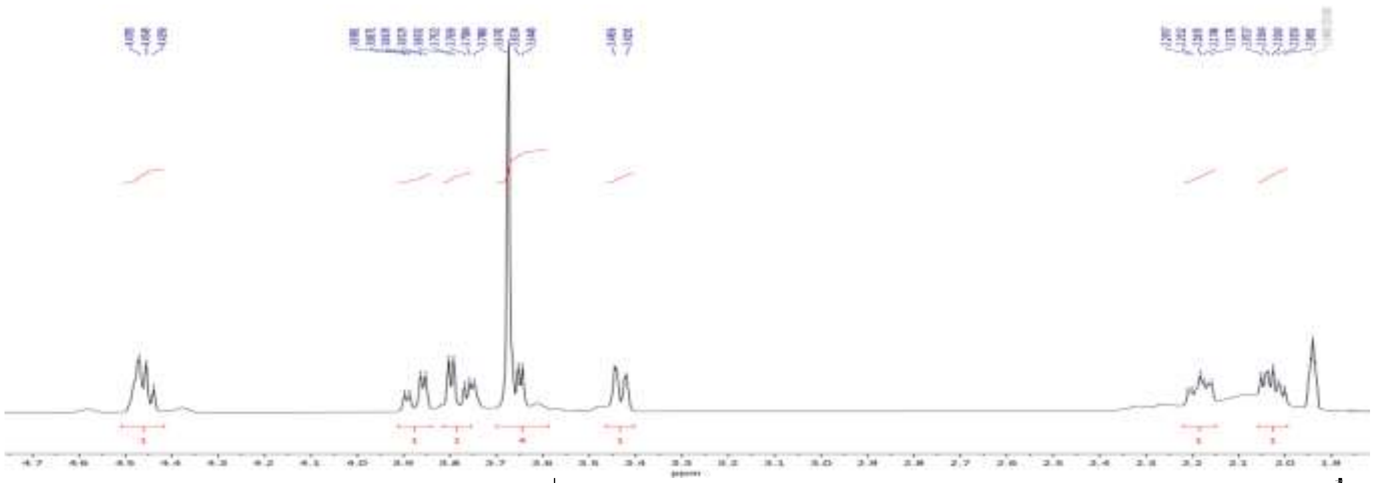

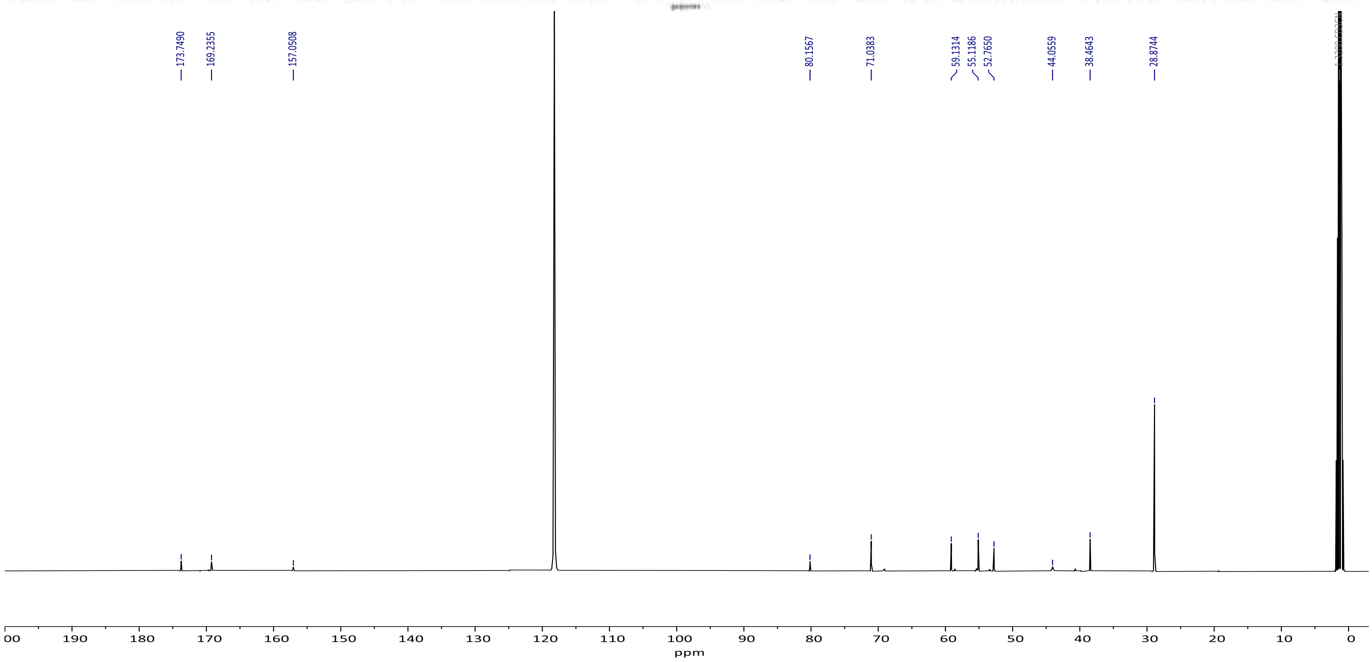

Compound **5**, ^{1}H and ^{13}C NMR at 26°C in $CDCl_3$

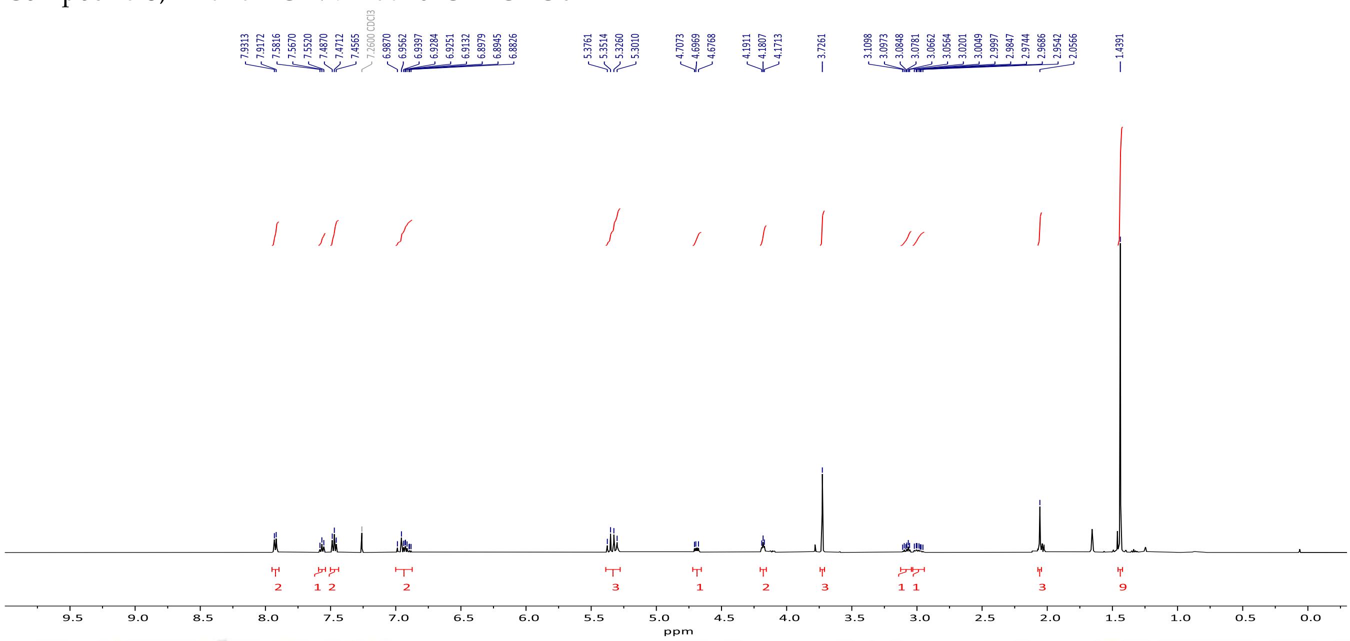

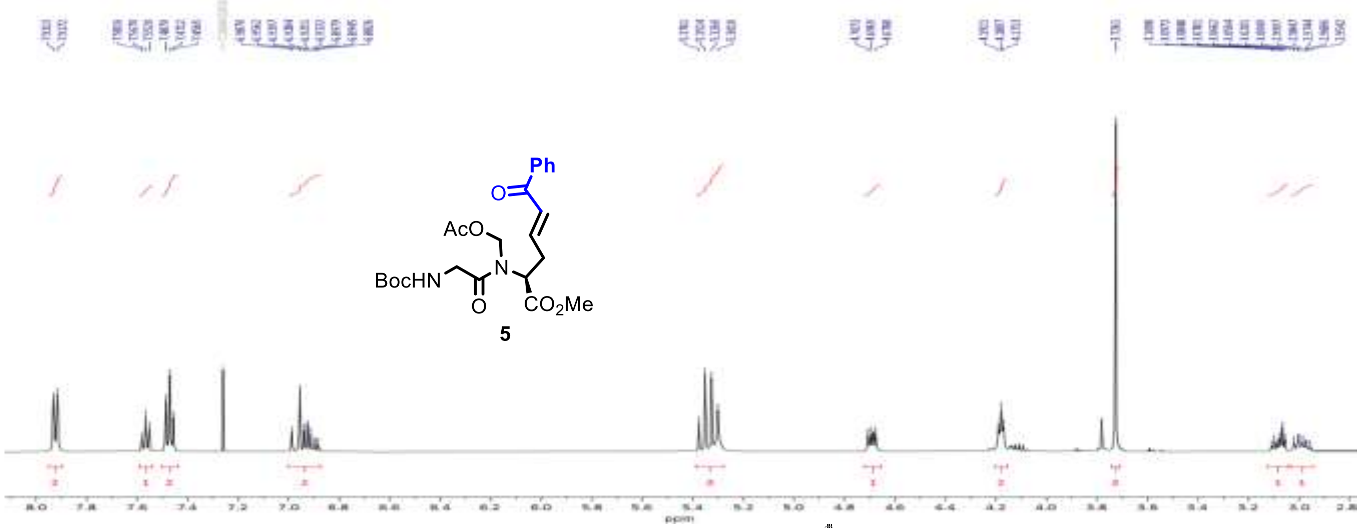

5

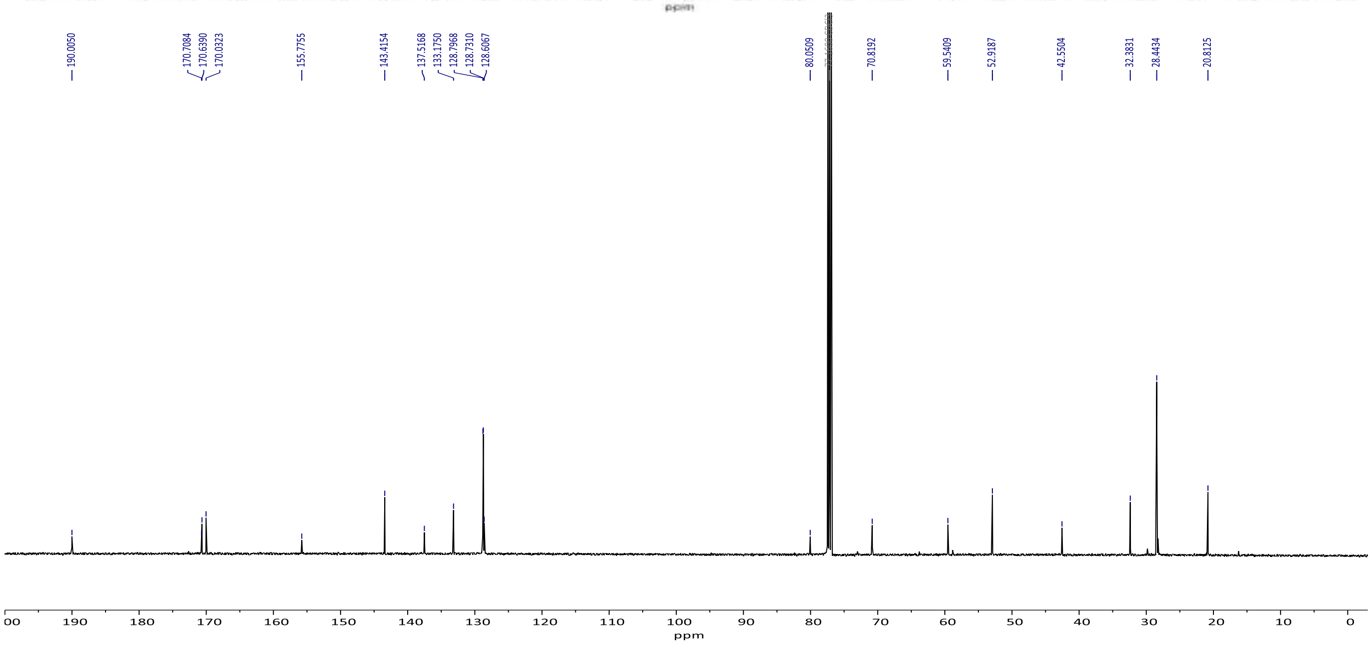

Compound **6**, ^{1}H and ^{13}C NMR and NOESY at 26°C in $CDCl_3$

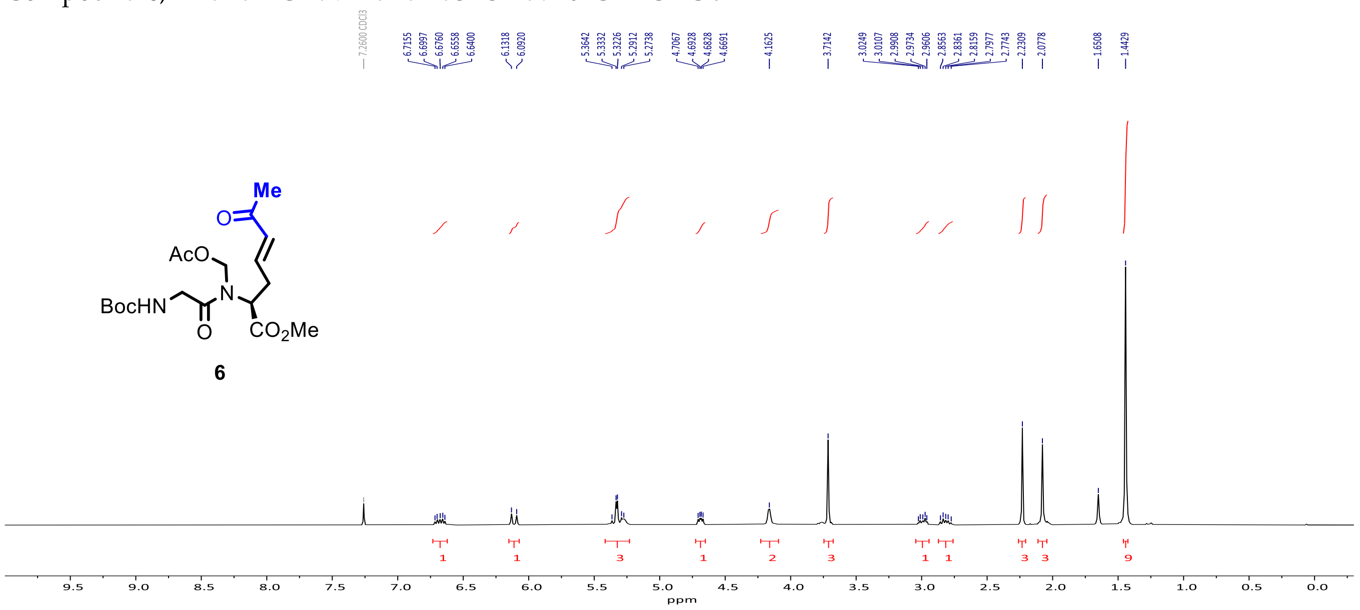

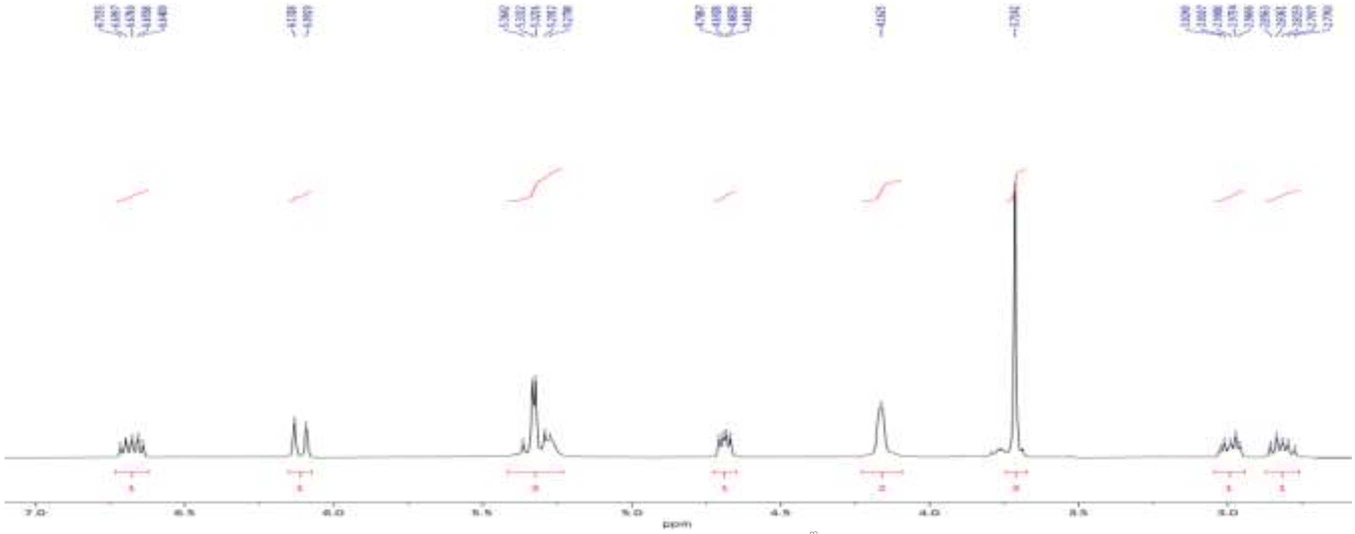

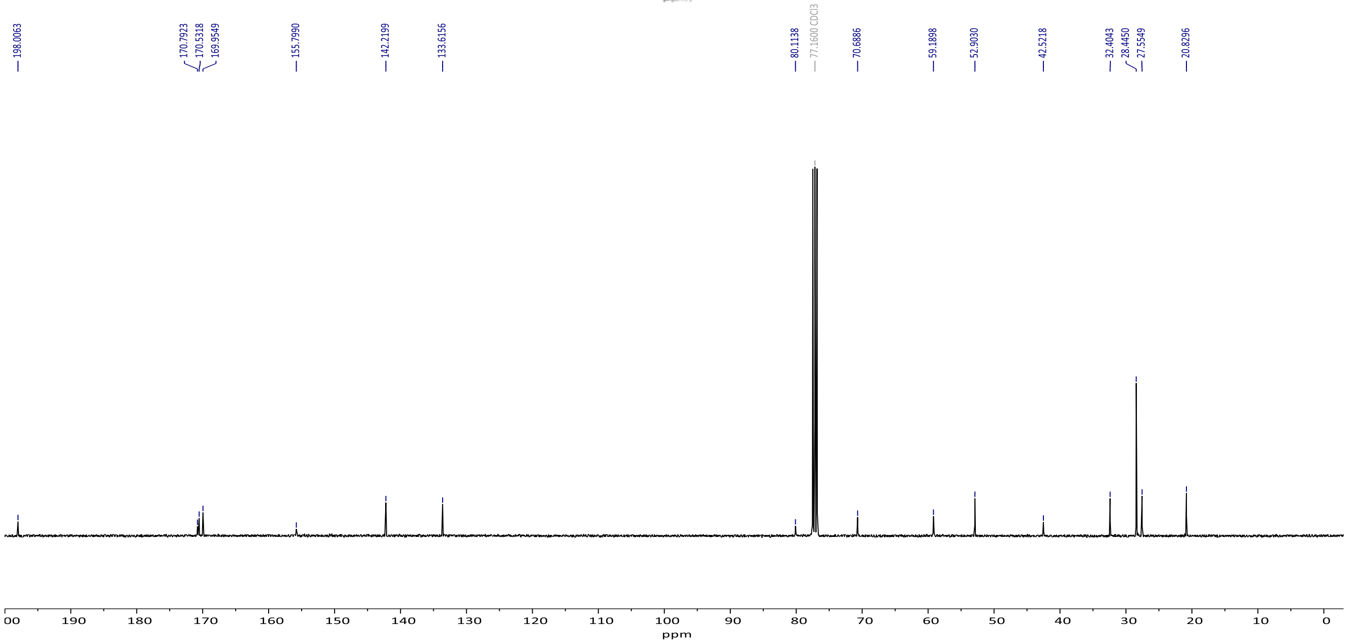

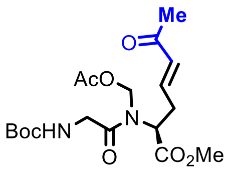

6

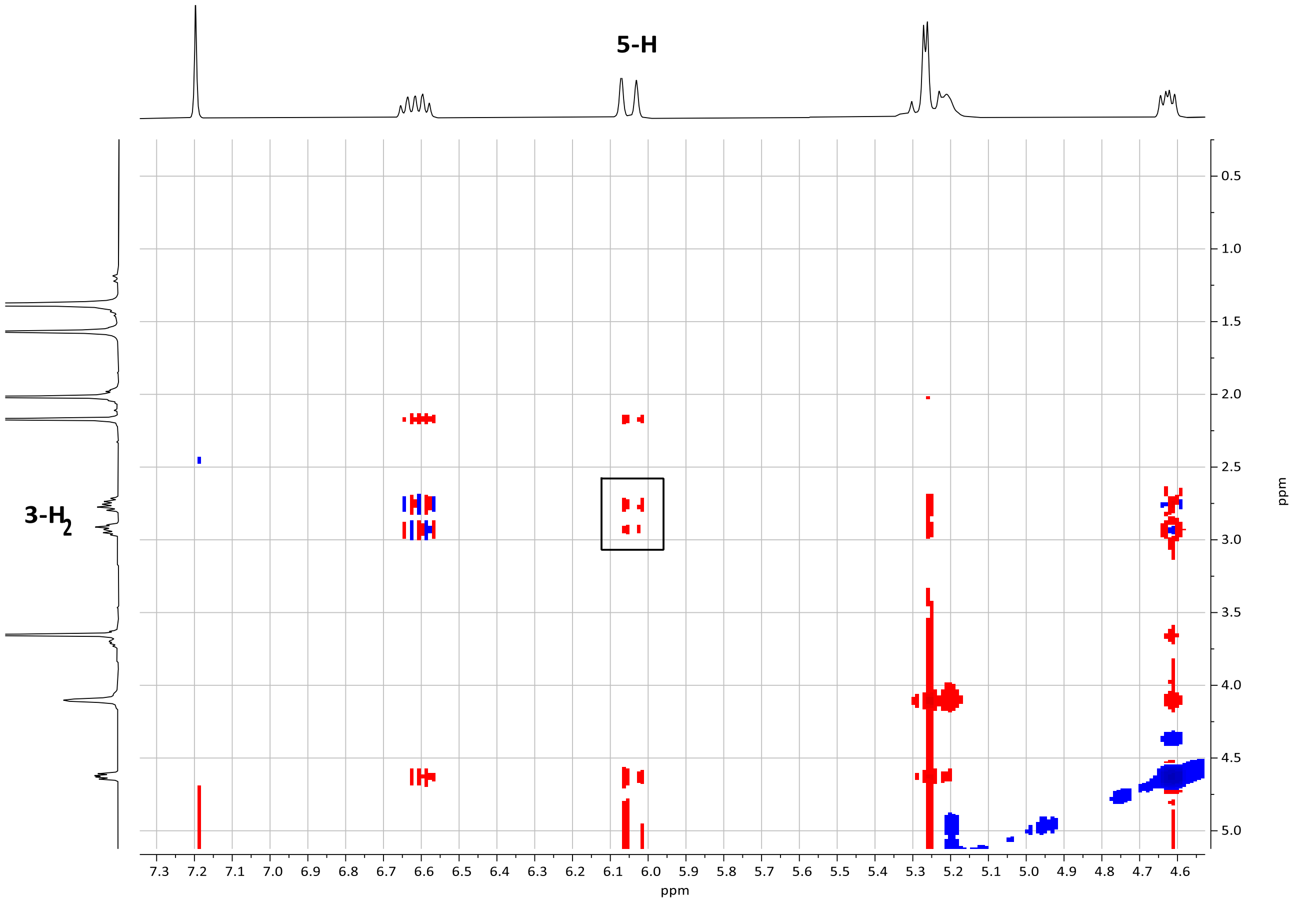

Compound **7**, ^{1}H and ^{13}C NMR at 70°C in CD_3CN

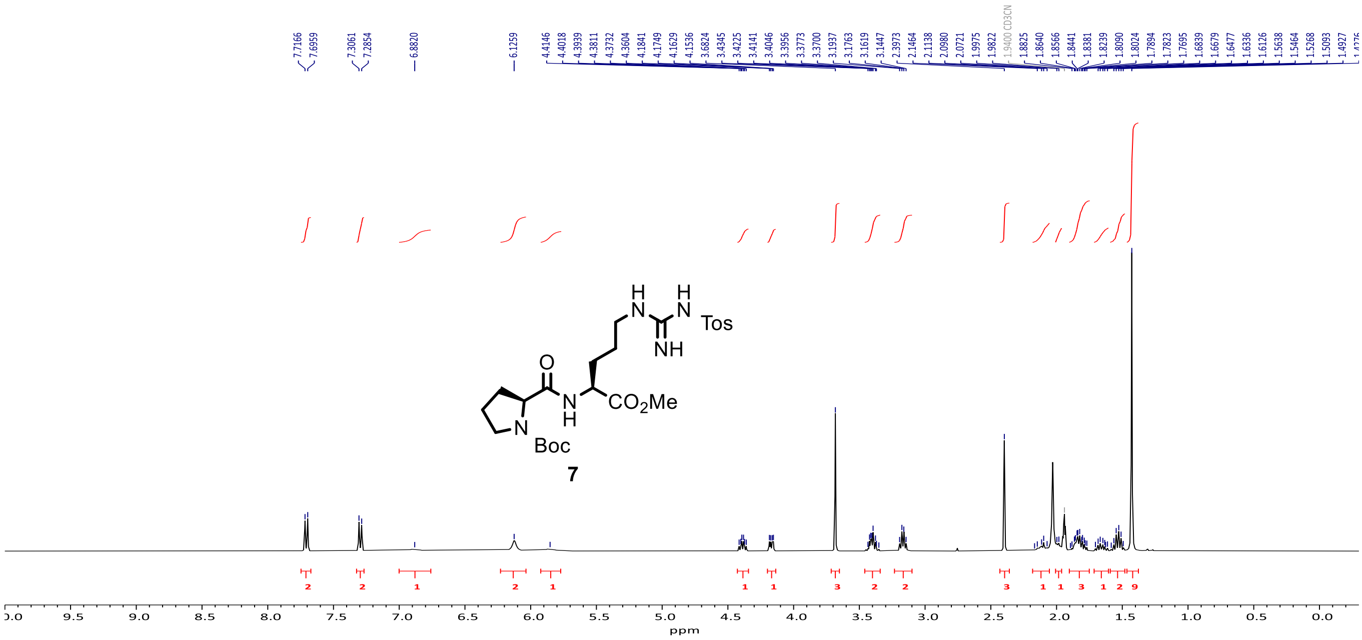

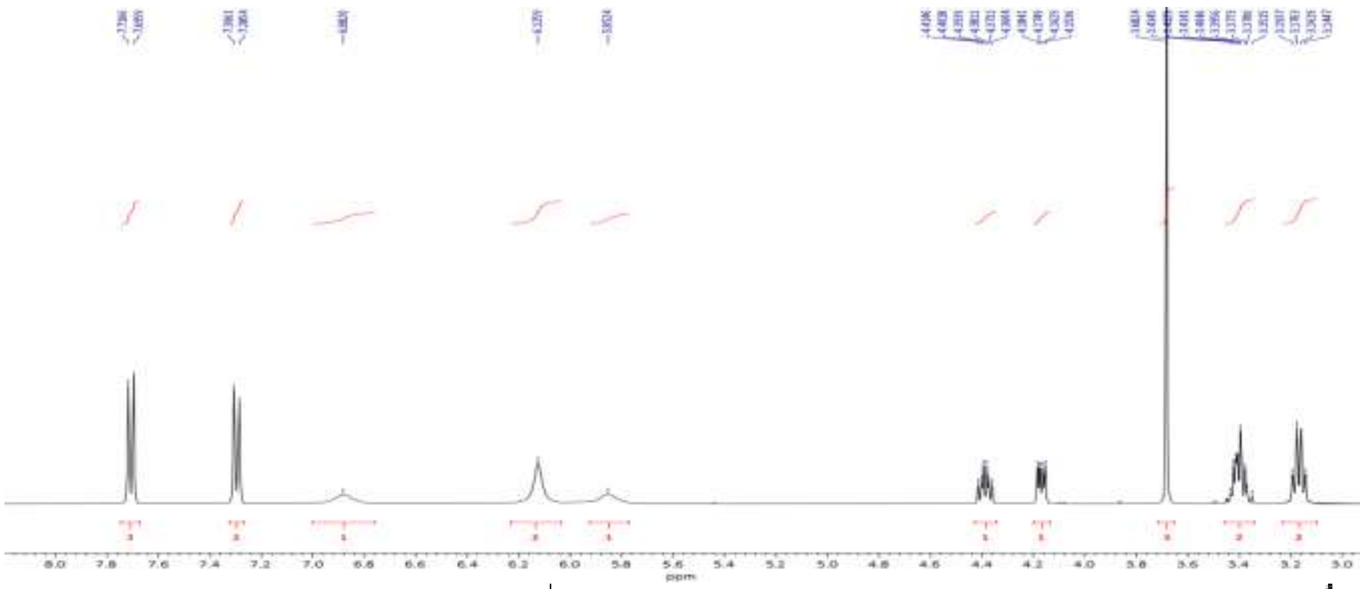

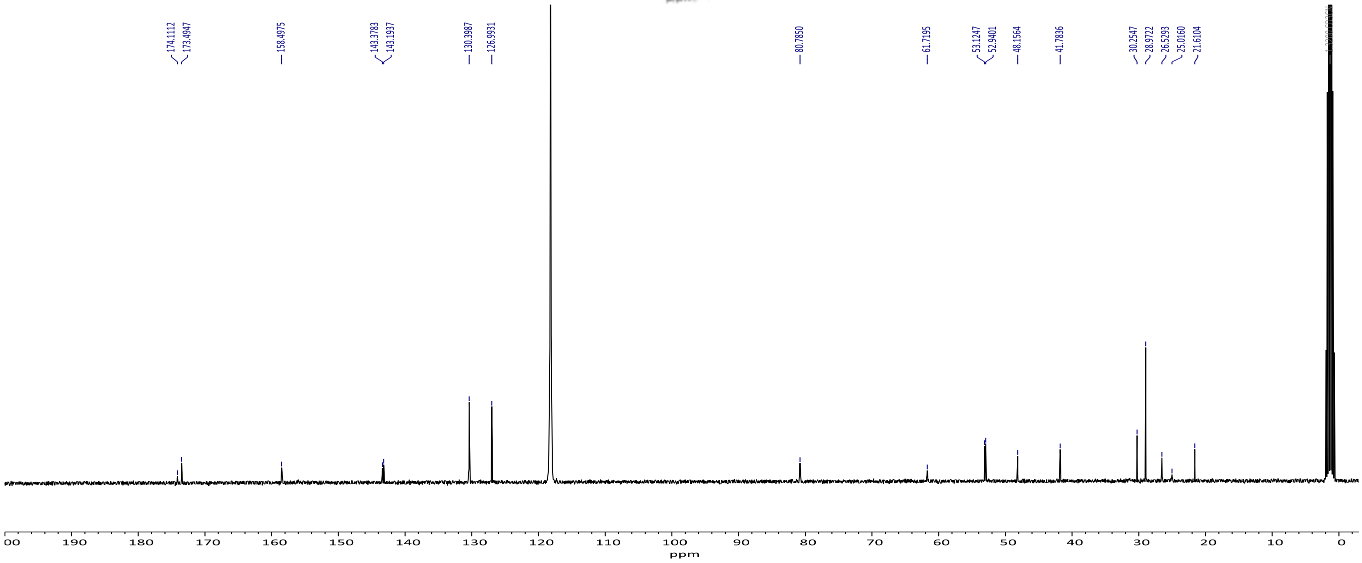

Compound **9**, ^{1}H and ^{13}C NMR at 70°C in CD_3CN

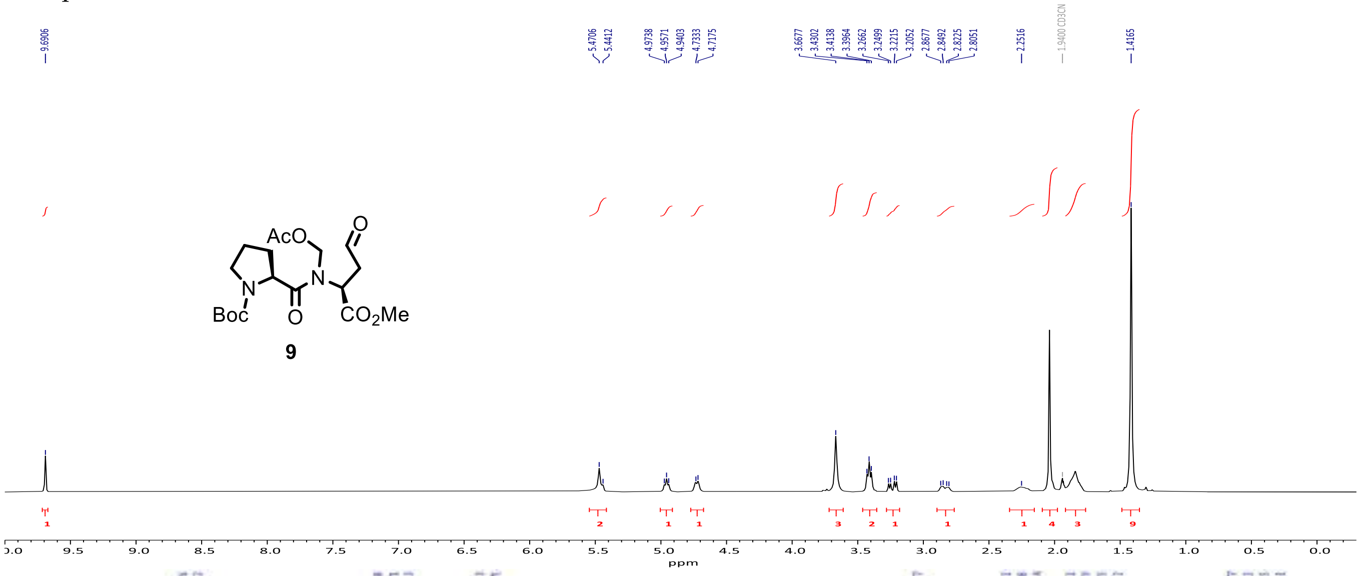

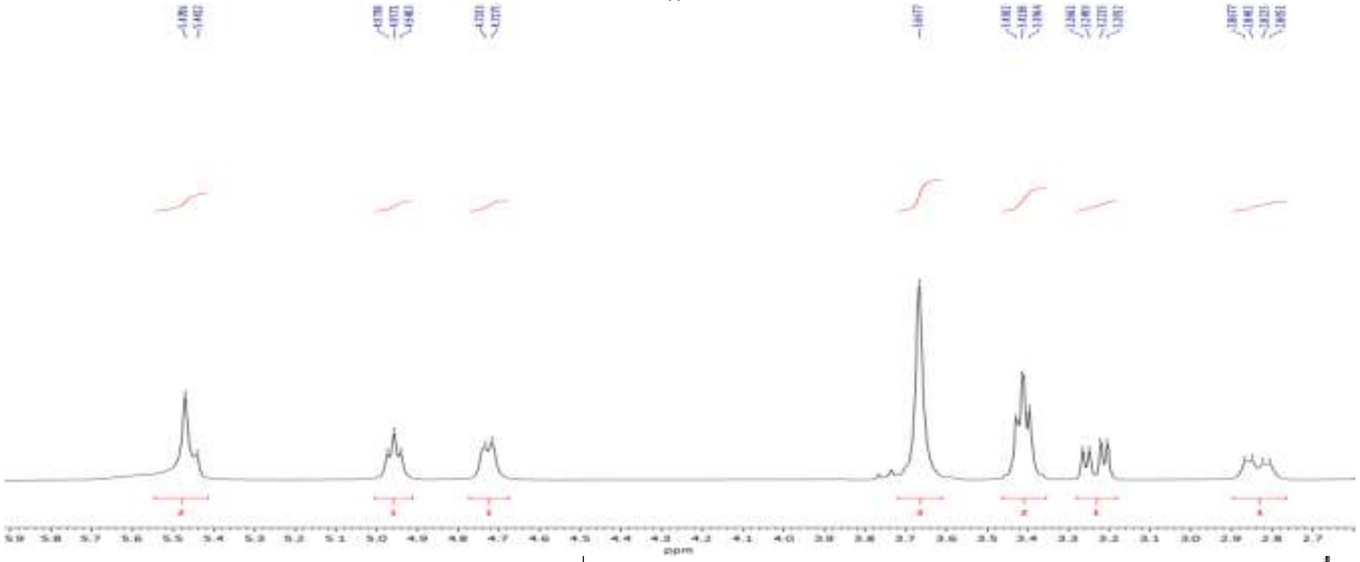

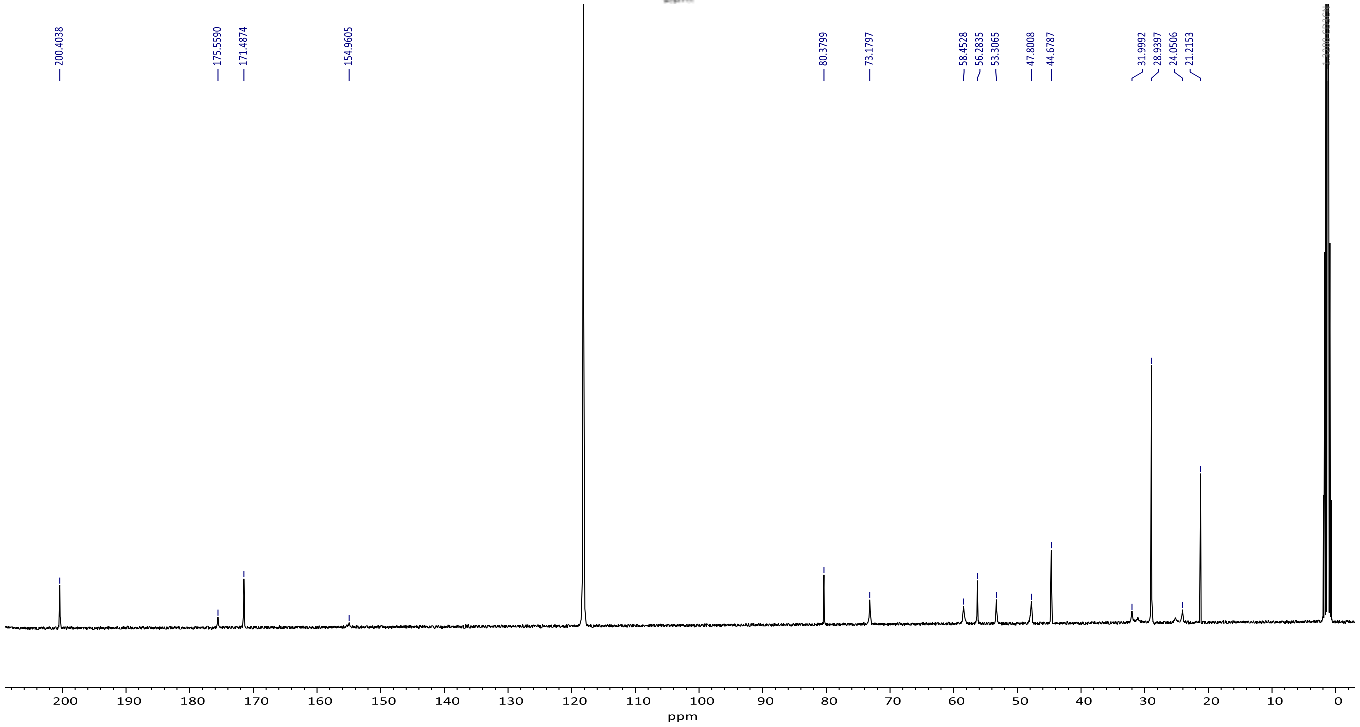

Compound **10**, ^{1}H NMR at 70°C in CD_3CN

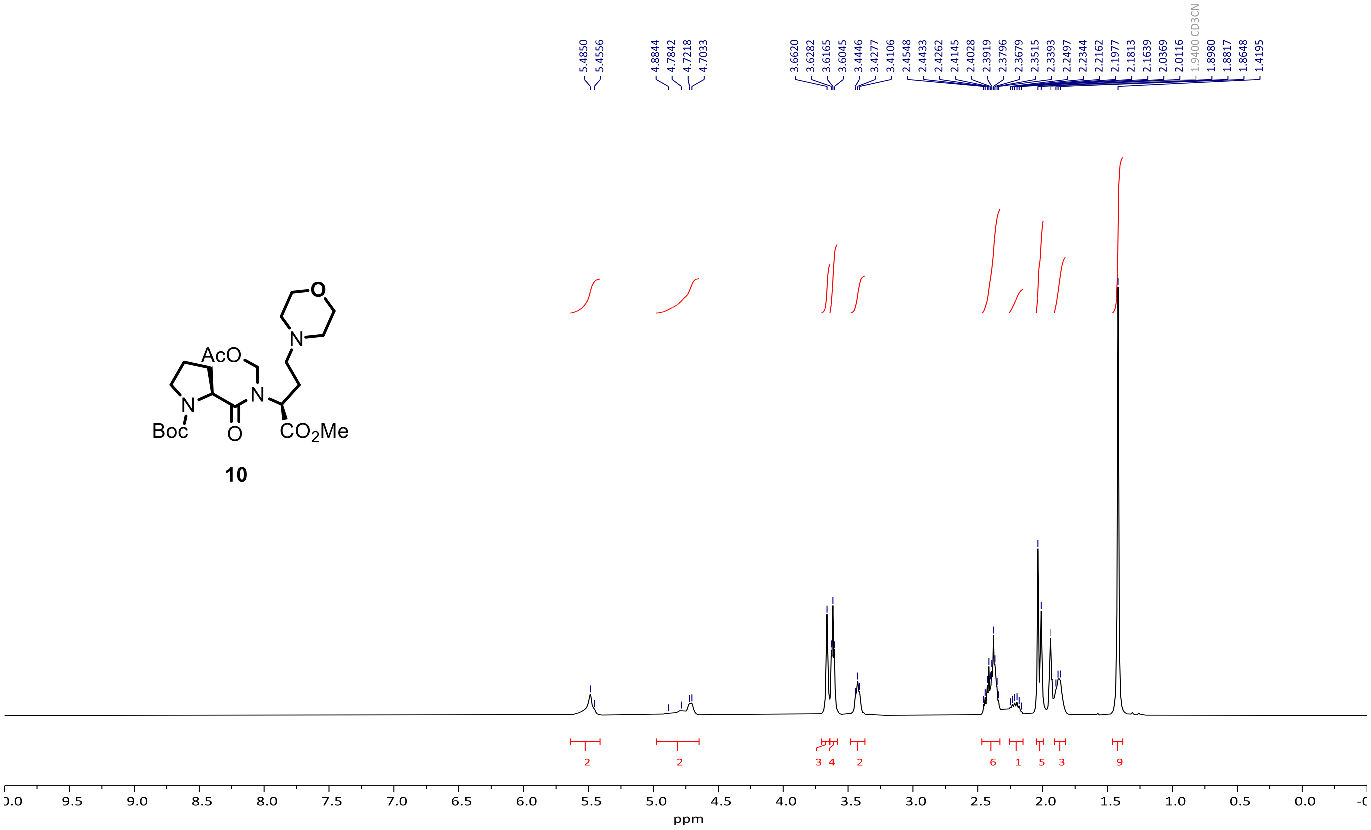

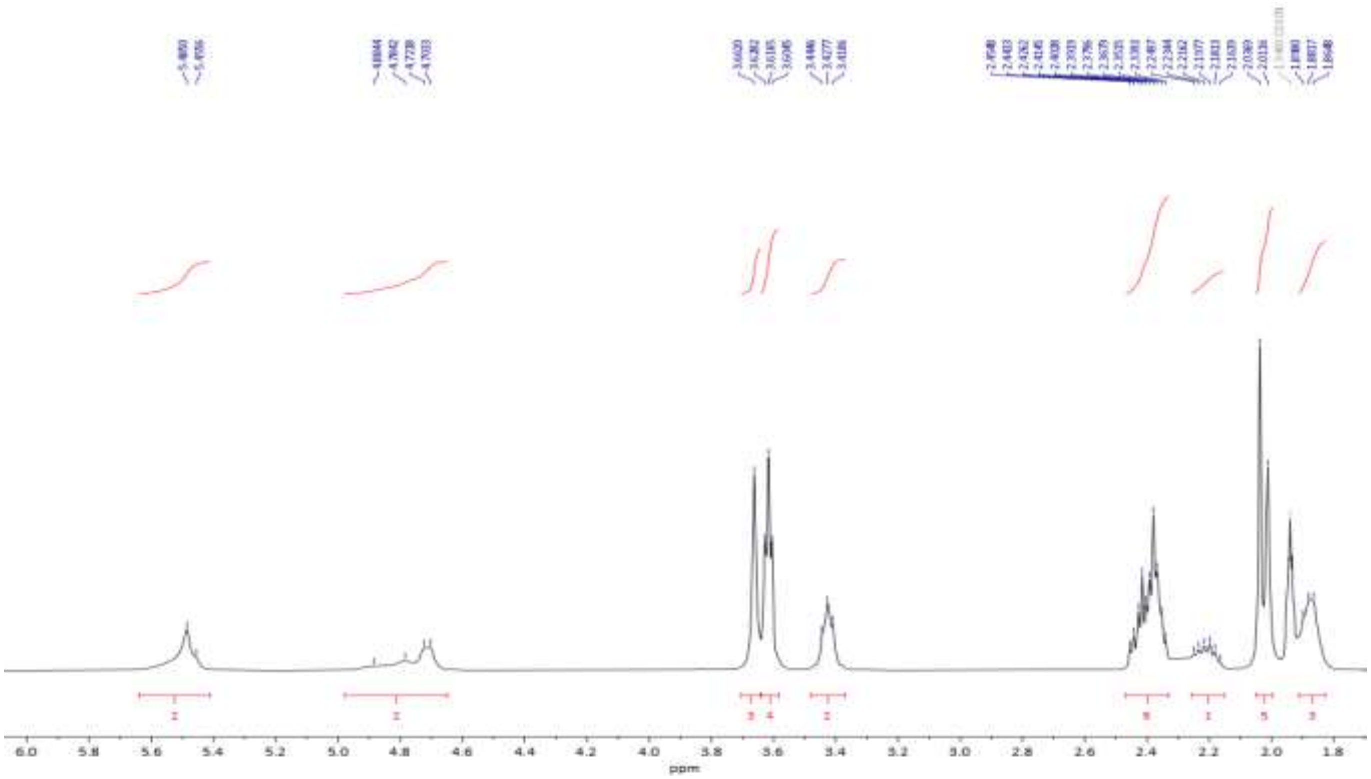

Compound **10**, ^{1}H and ^{13}C NMR at 26°C in $CDCl_3$

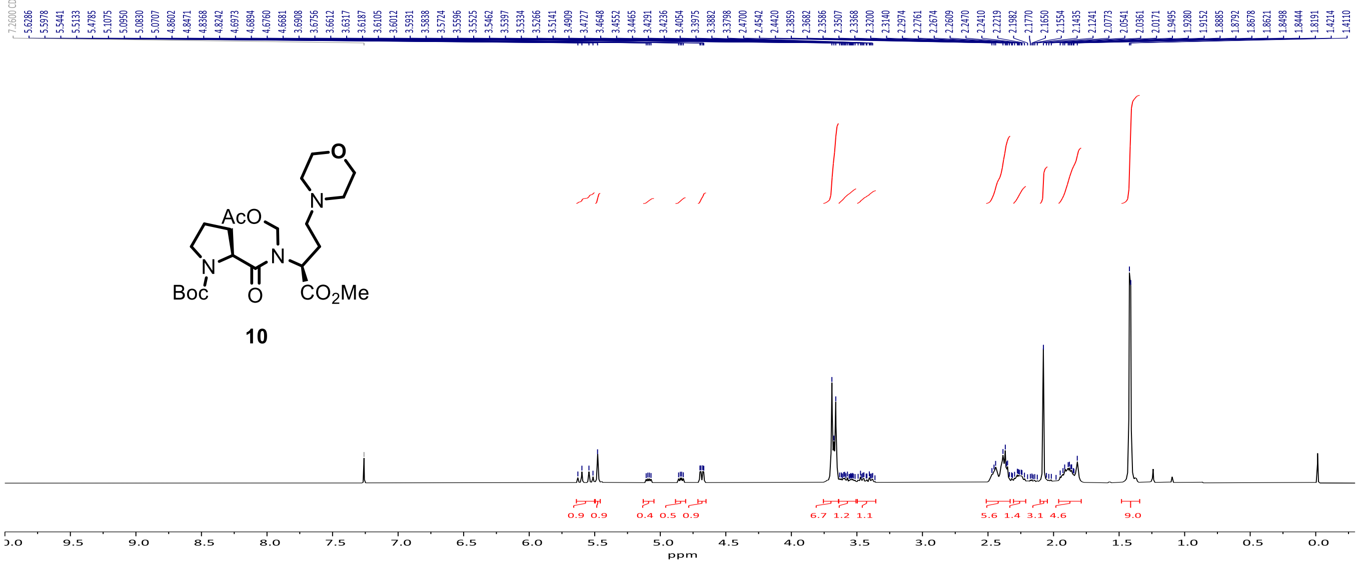

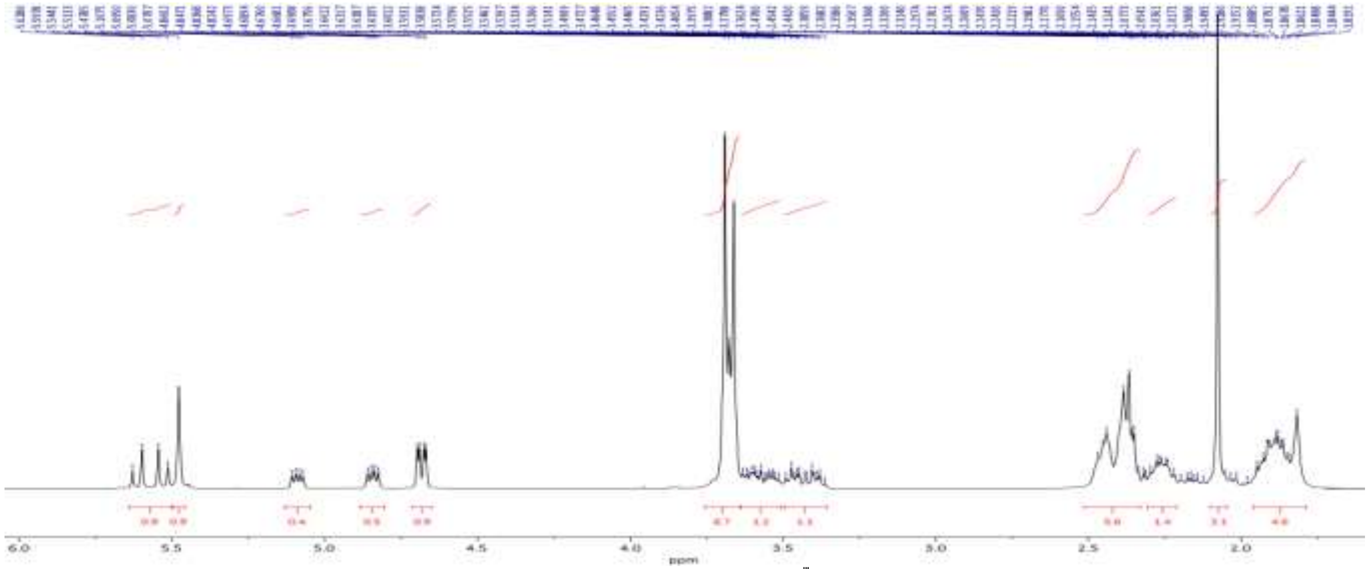

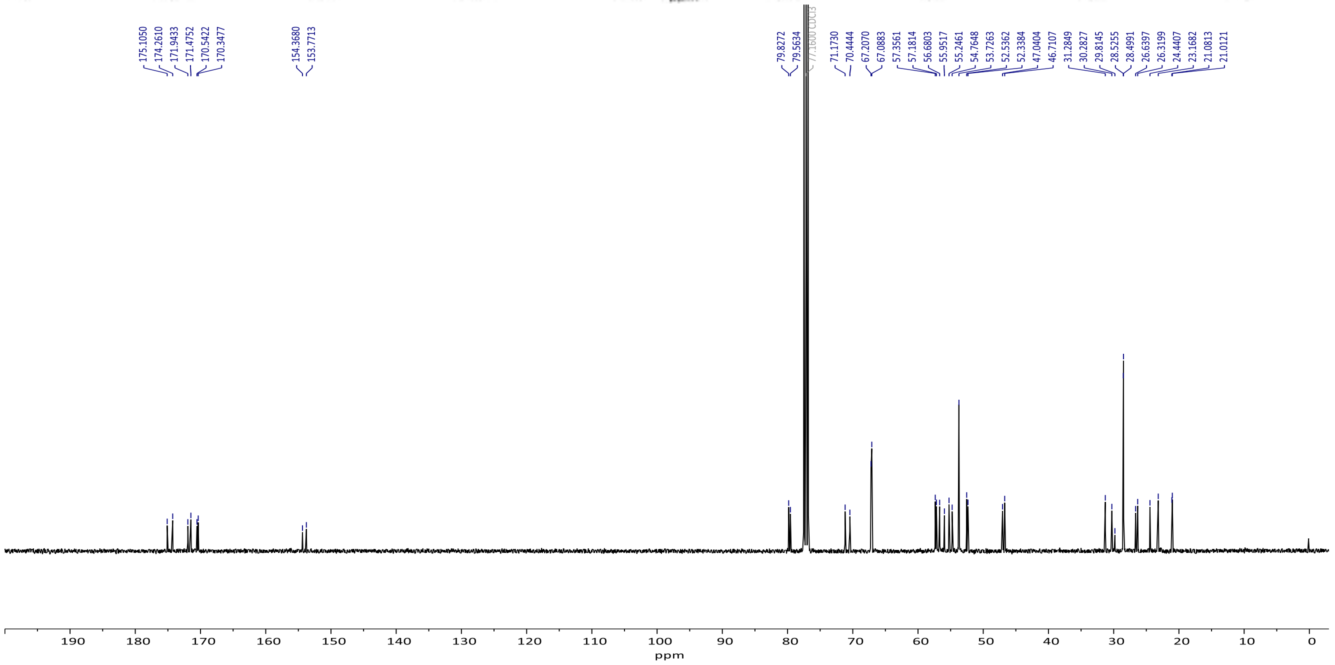

Compound **11**, ^{1}H NMR at 70°C in CD_3CN and ^{13}C NMR at 26°C in $CDCl_3$

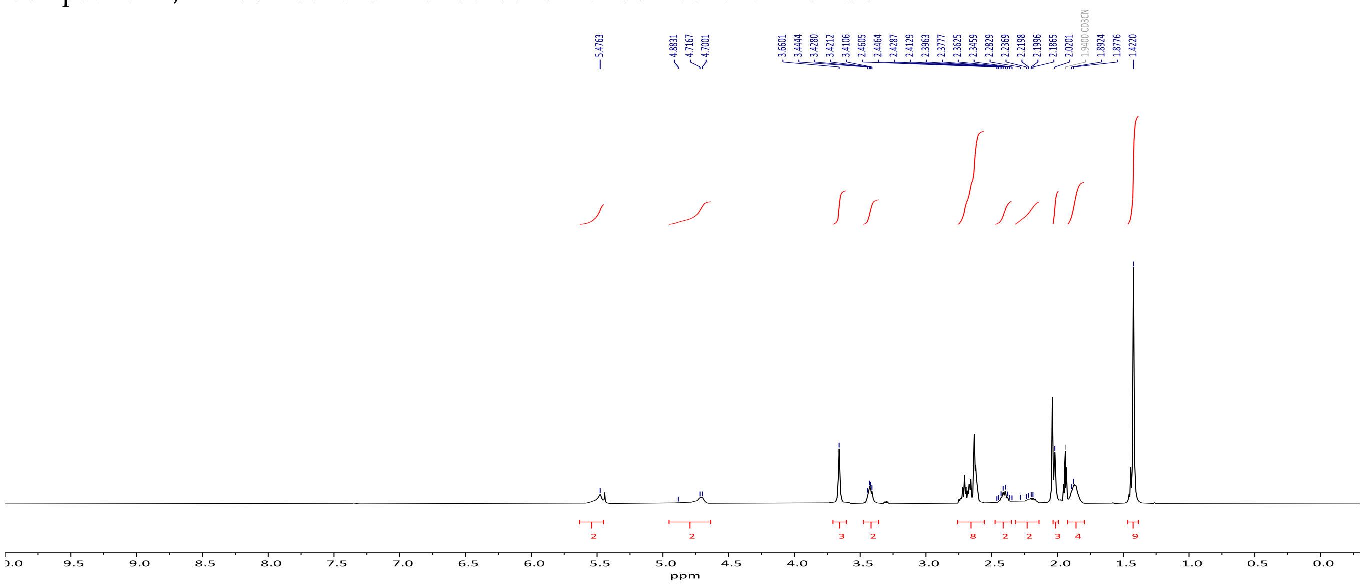

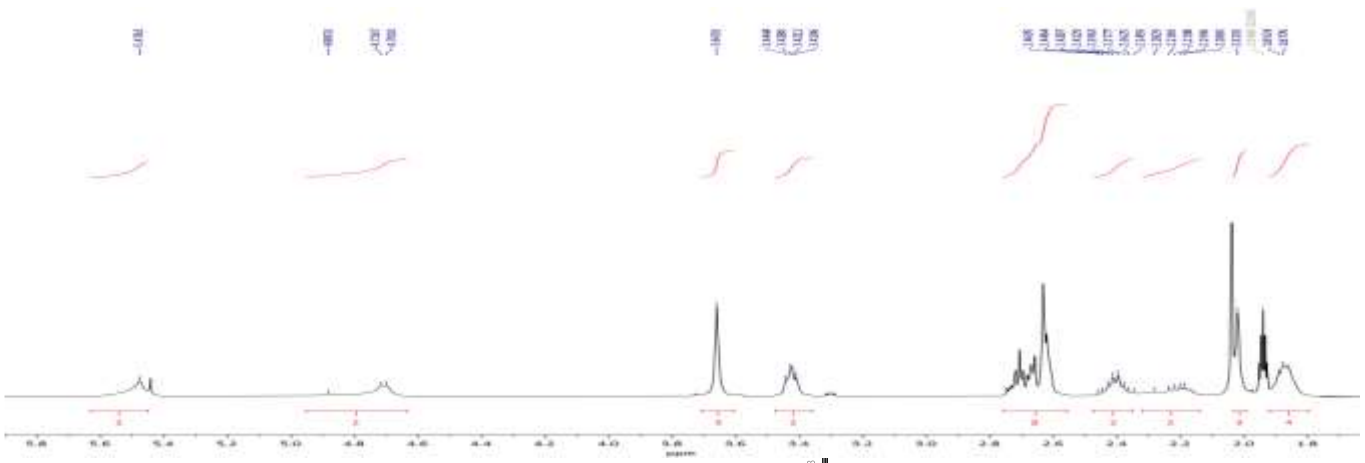

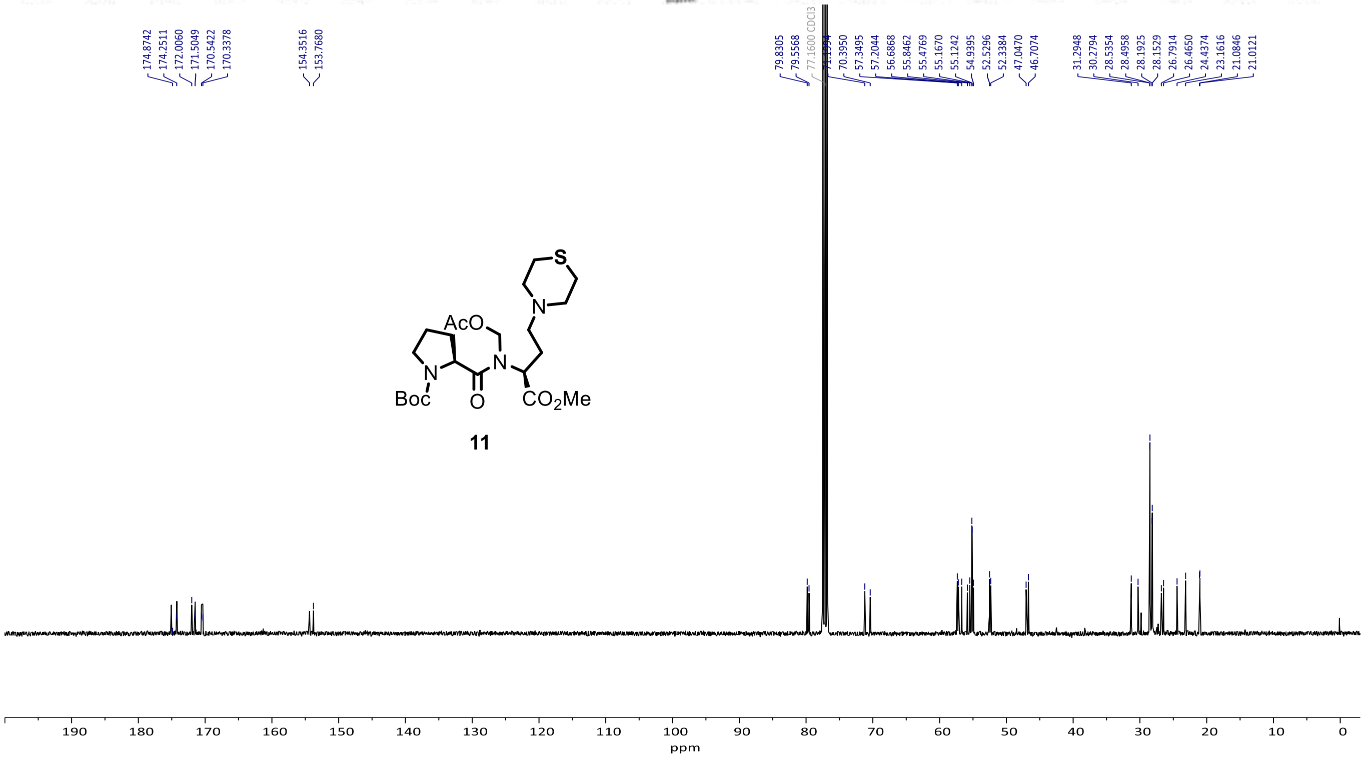

11

Compound **12**, ^{1}H and ^{13}C NMR at 55°C in CDCl$_3$

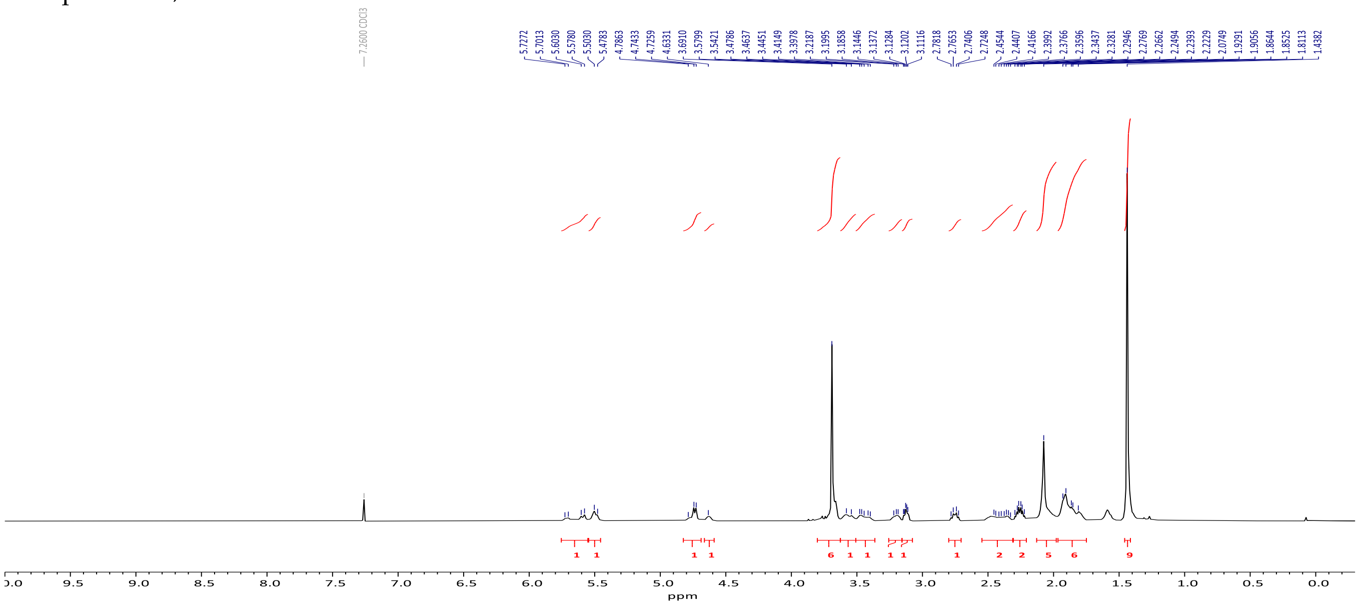

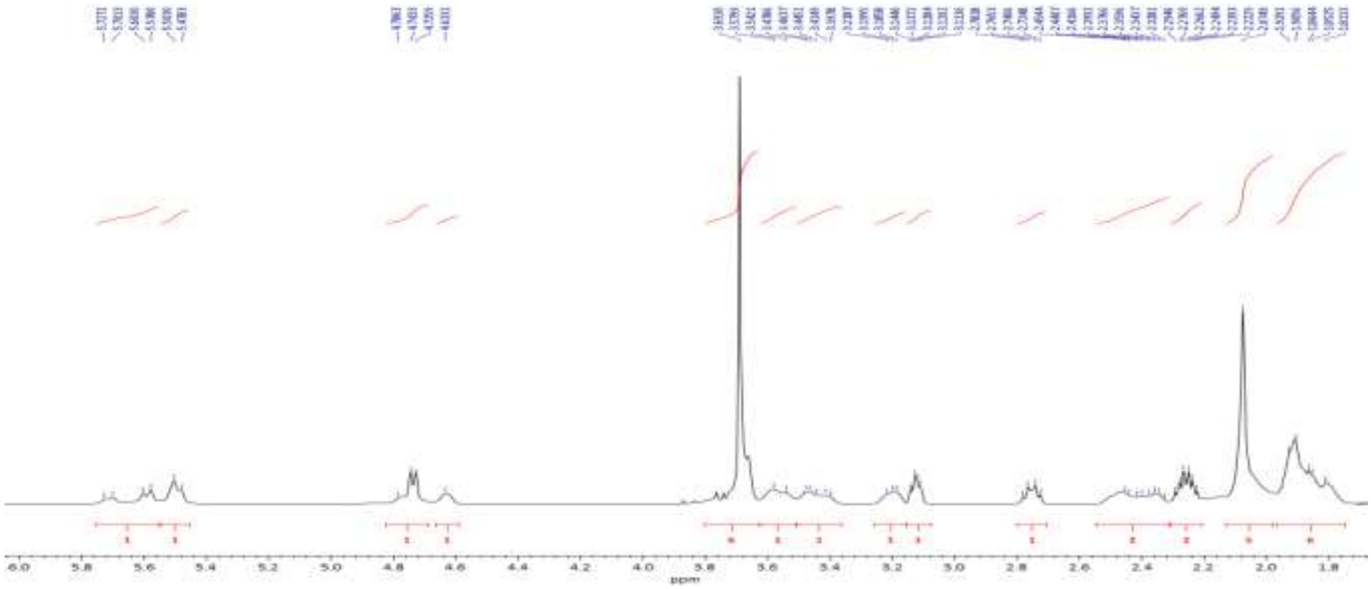

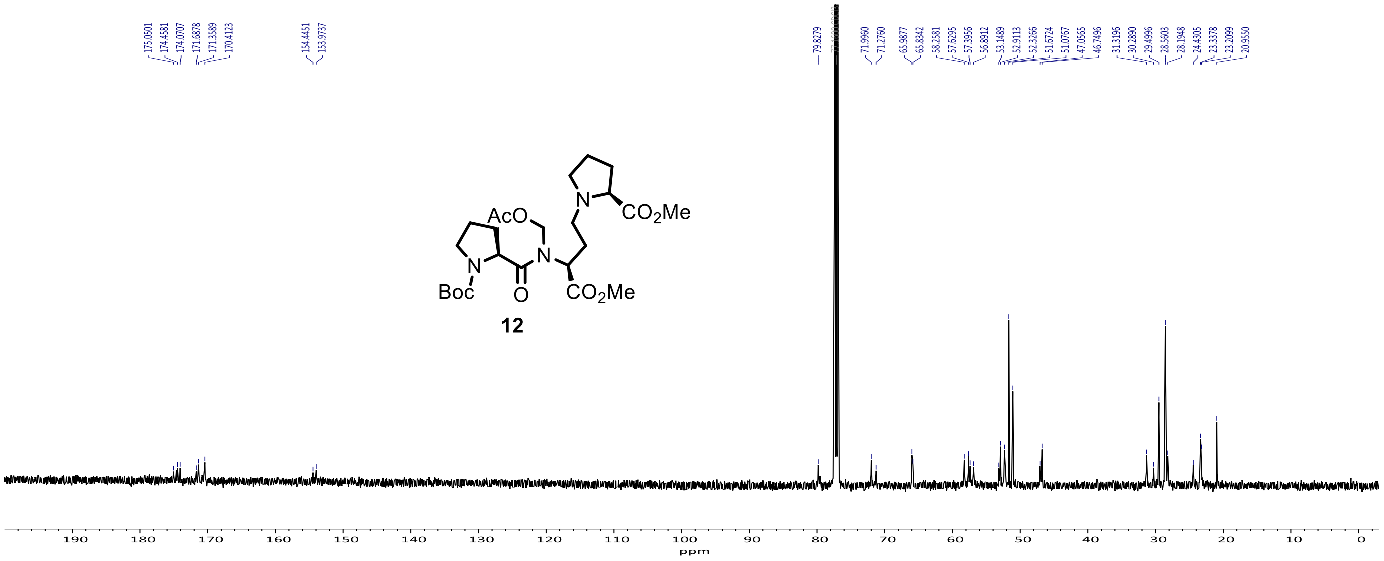

12

Compound **13**, ^{1}H and ^{13}C NMR at 26°C in CD_3OD

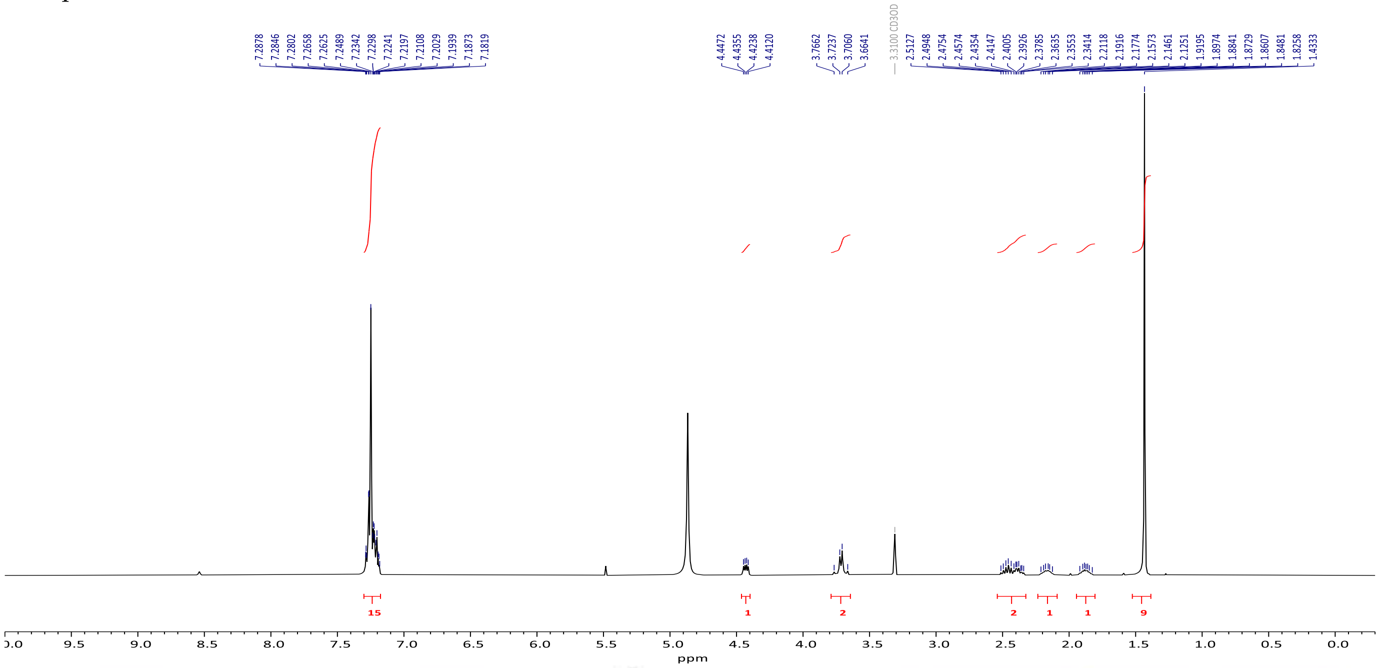

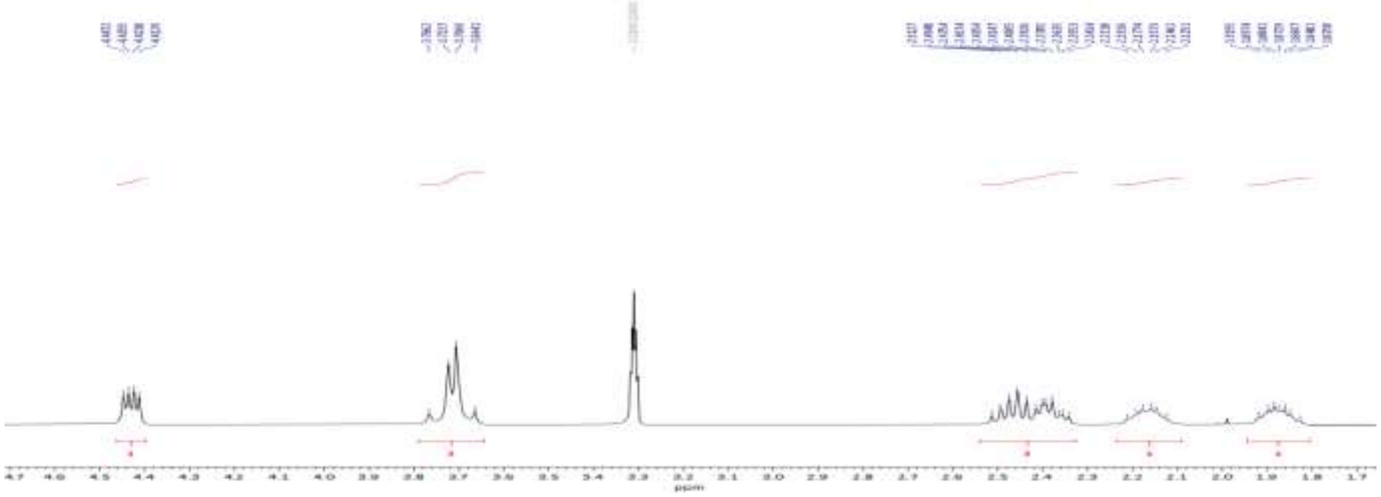

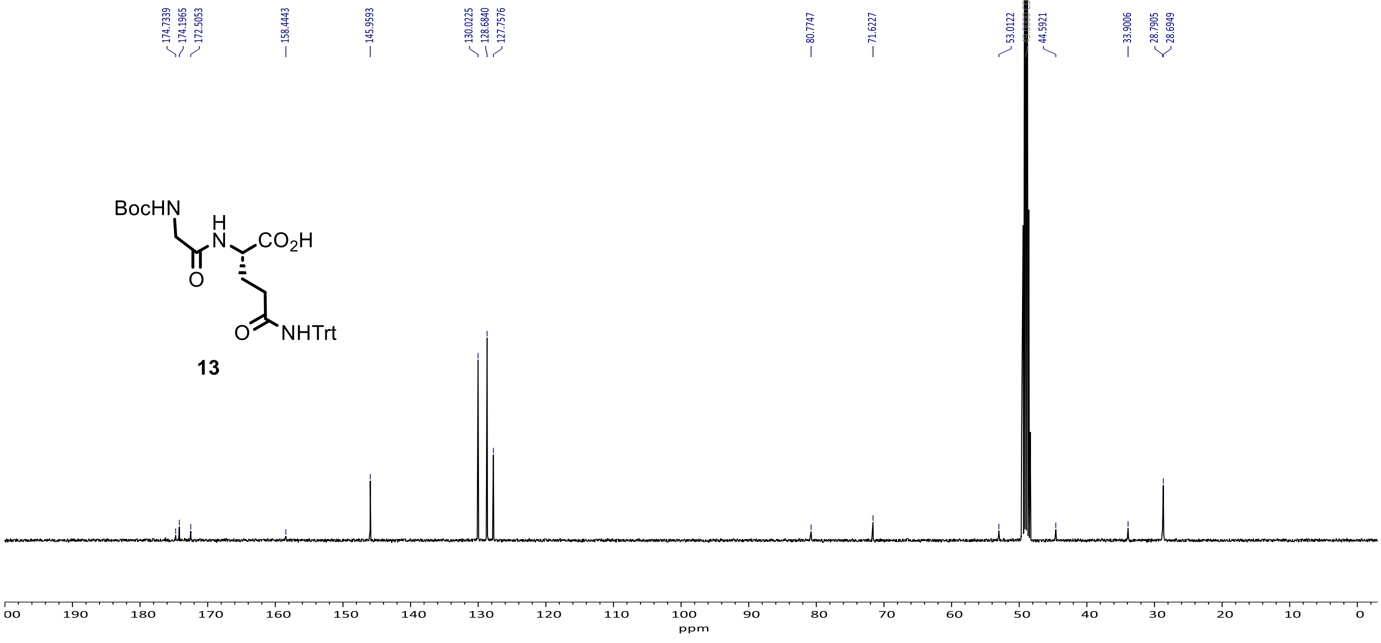

13

Compound **14**, ^{1}H and ^{13}C NMR at 26°C in CD_3OD

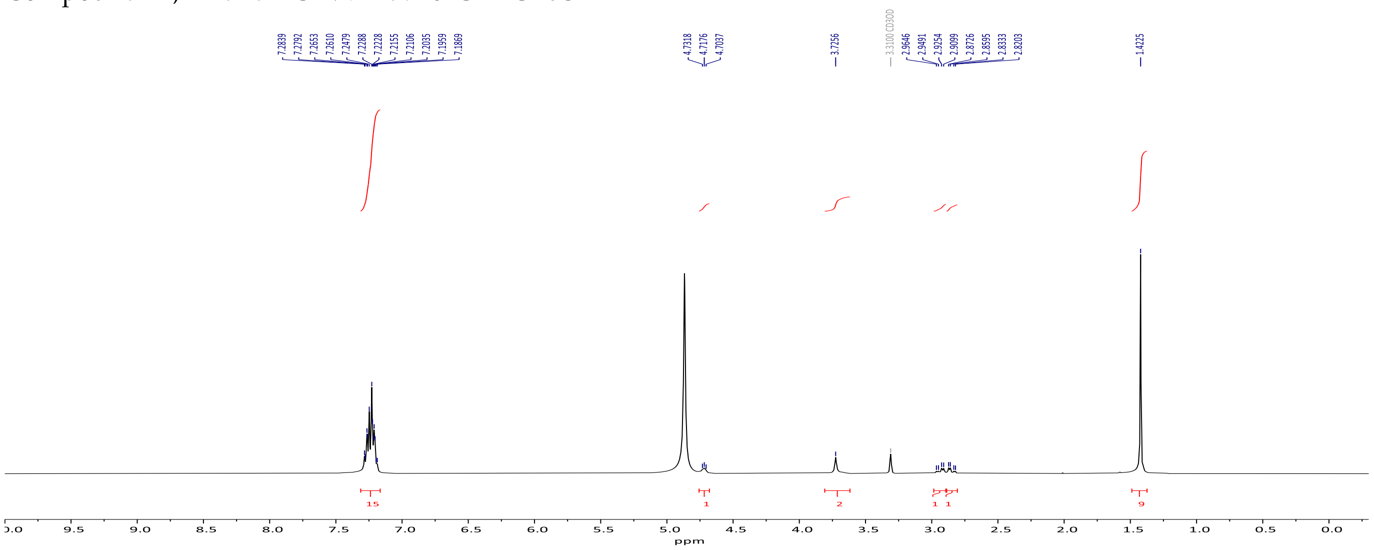

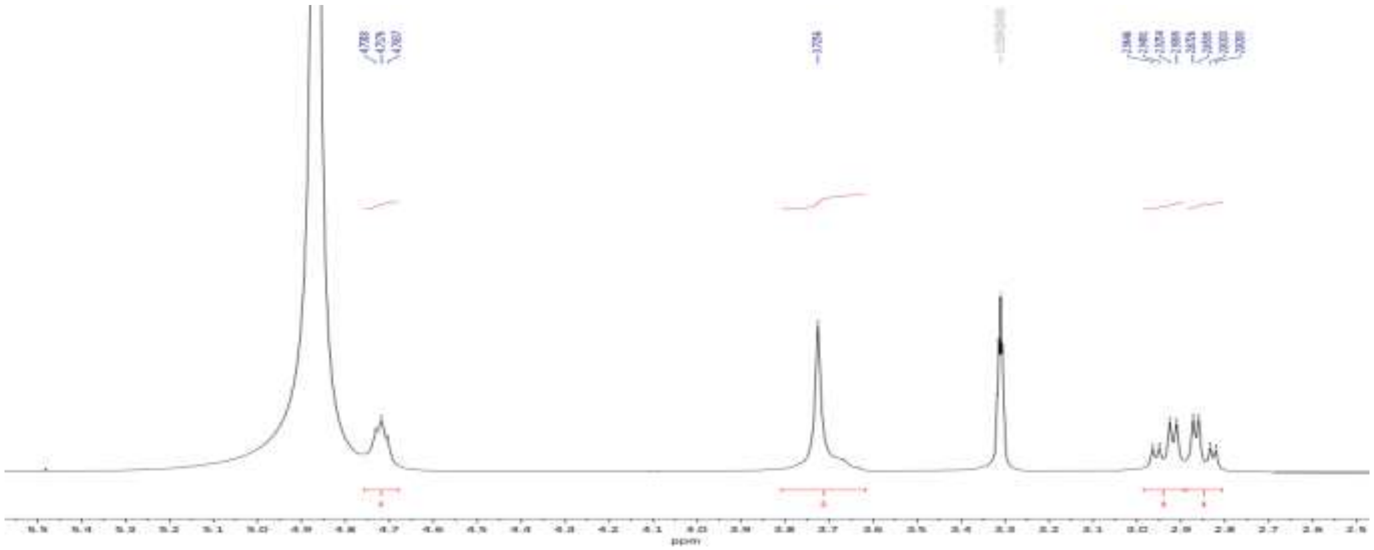

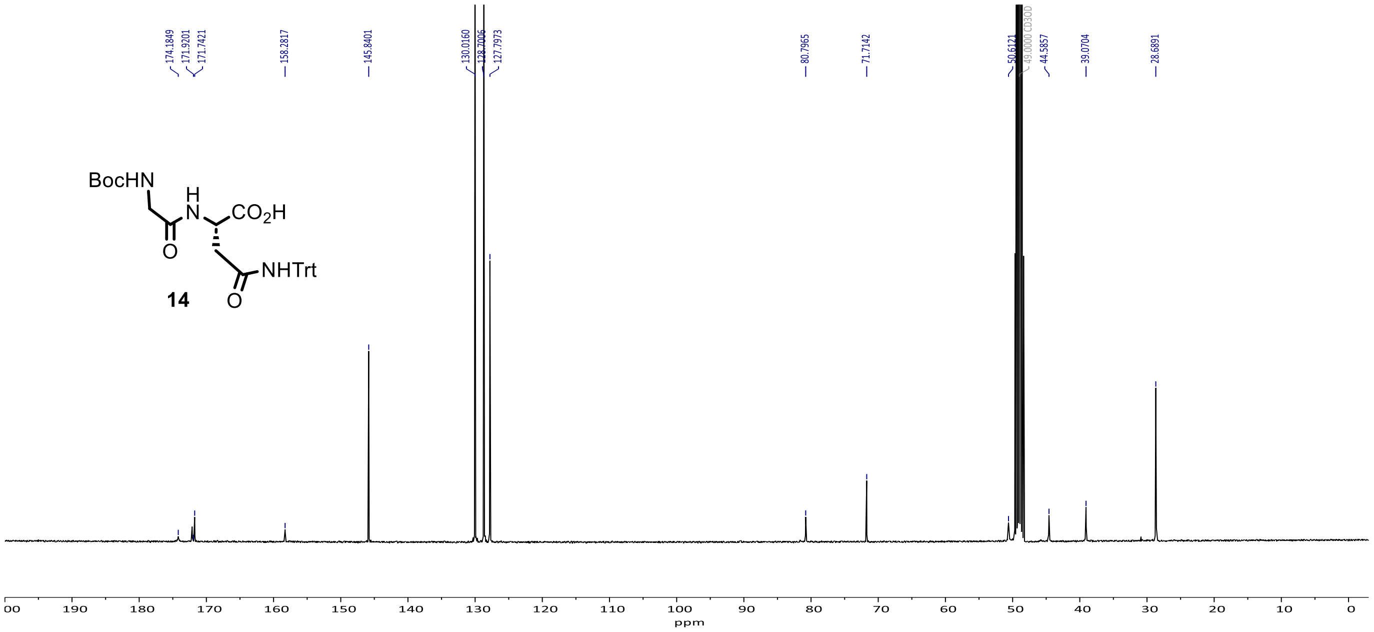

14

Compound **15**, ^1H and ^{13}C NMR at 70°C in CD_3CN

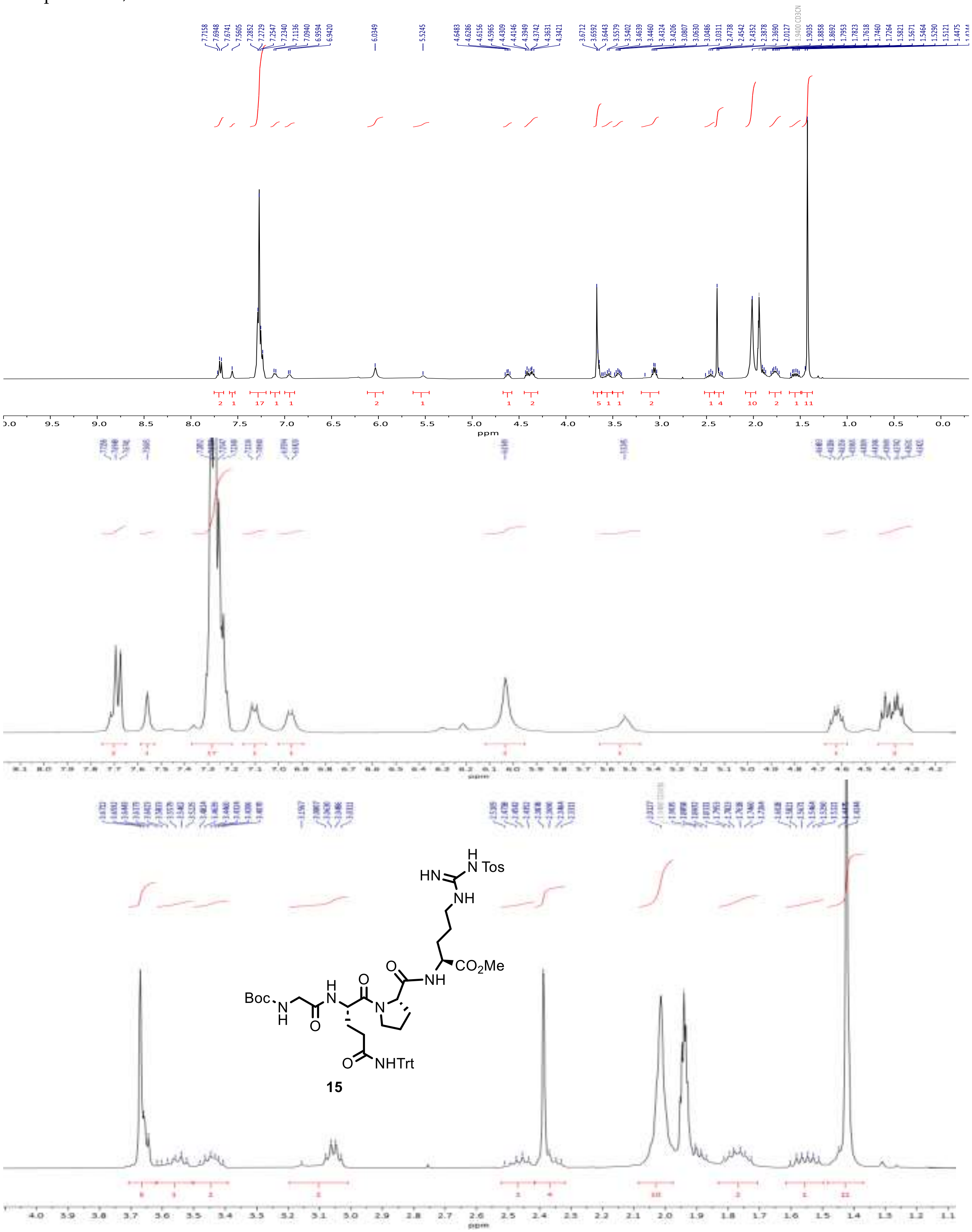

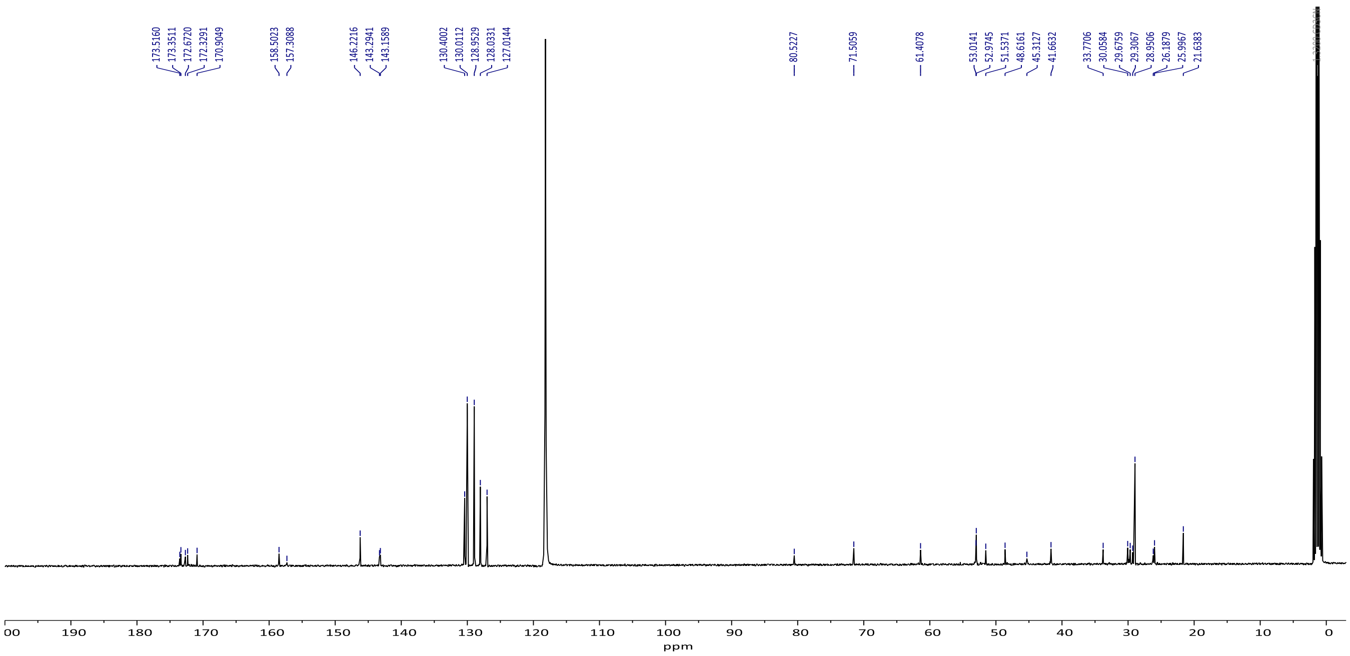
173.5160
173.3511
172.6720
172.3291
170.9049
158.5023
157.3088
146.2216
143.2941
143.1589
130.4002
130.0112
128.9529
128.0331
127.0144
80.5227
71.5059
61.4078
53.0141
52.9745
51.5371
48.6161
45.3127
41.6632
33.7706
30.0584
29.6759
29.3067
28.9506
26.1879
25.9967
21.6383
ppm

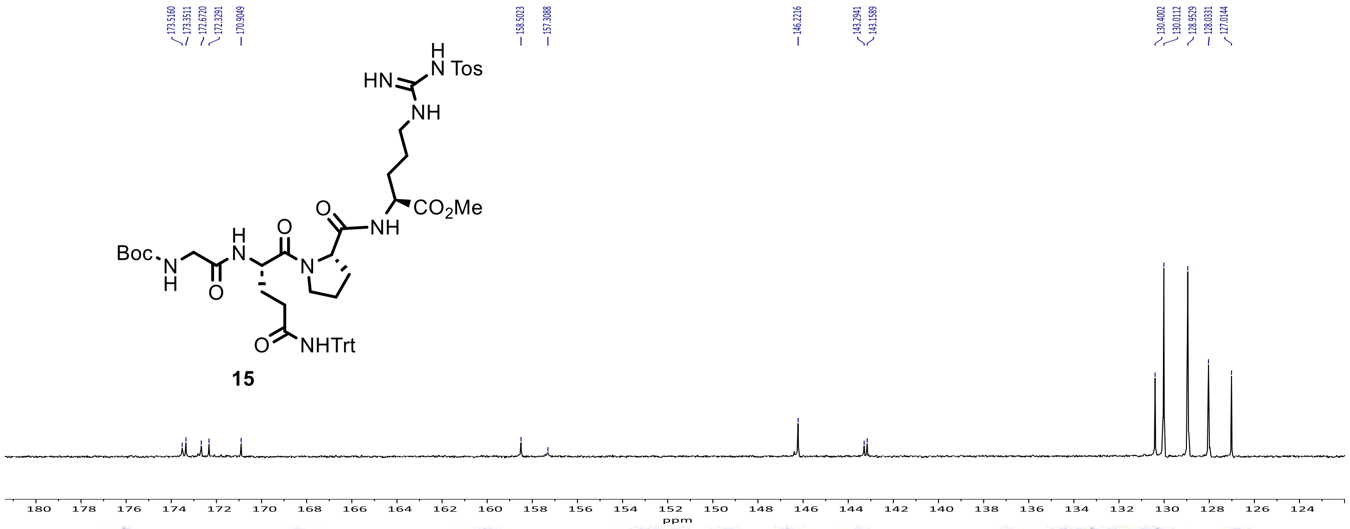
H
N Tos
HN
NH
O
CO2Me
NH
Boc
N
H
O
N
O
N
O
NHTrt
15
ppm

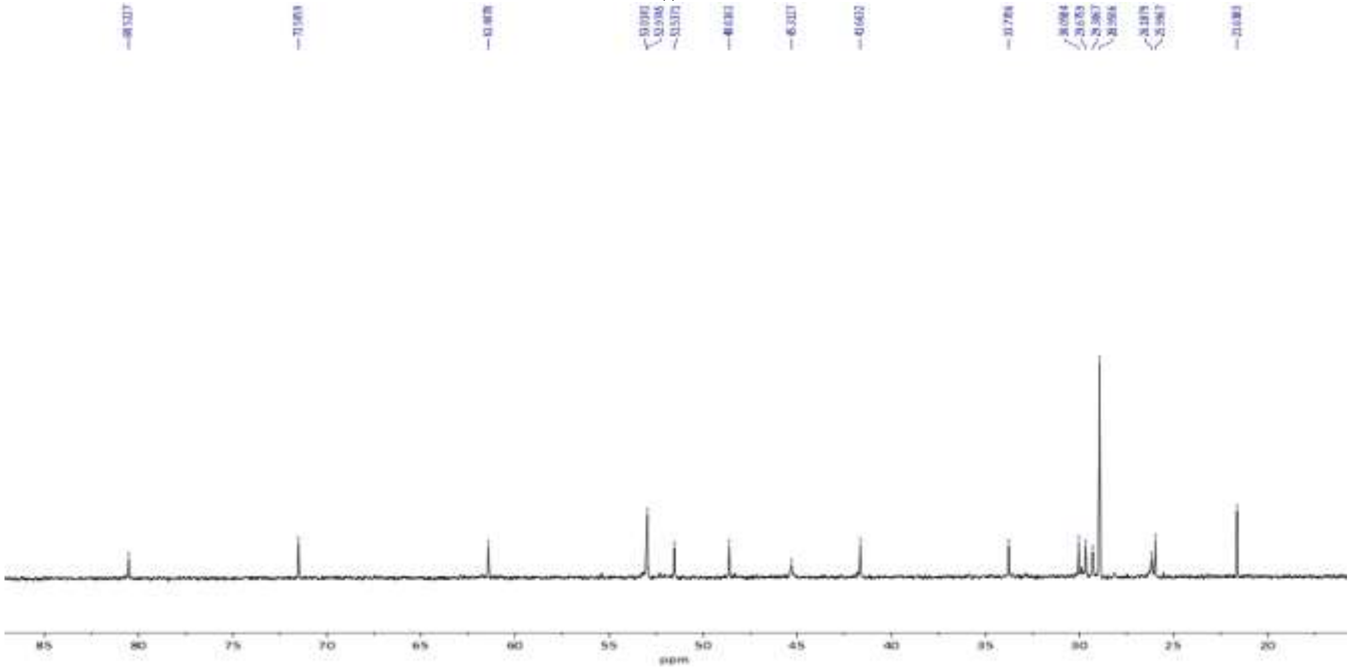
ppm

Compound **16**, ^{1}H and ^{13}C NMR at 70°C in CD_3CN

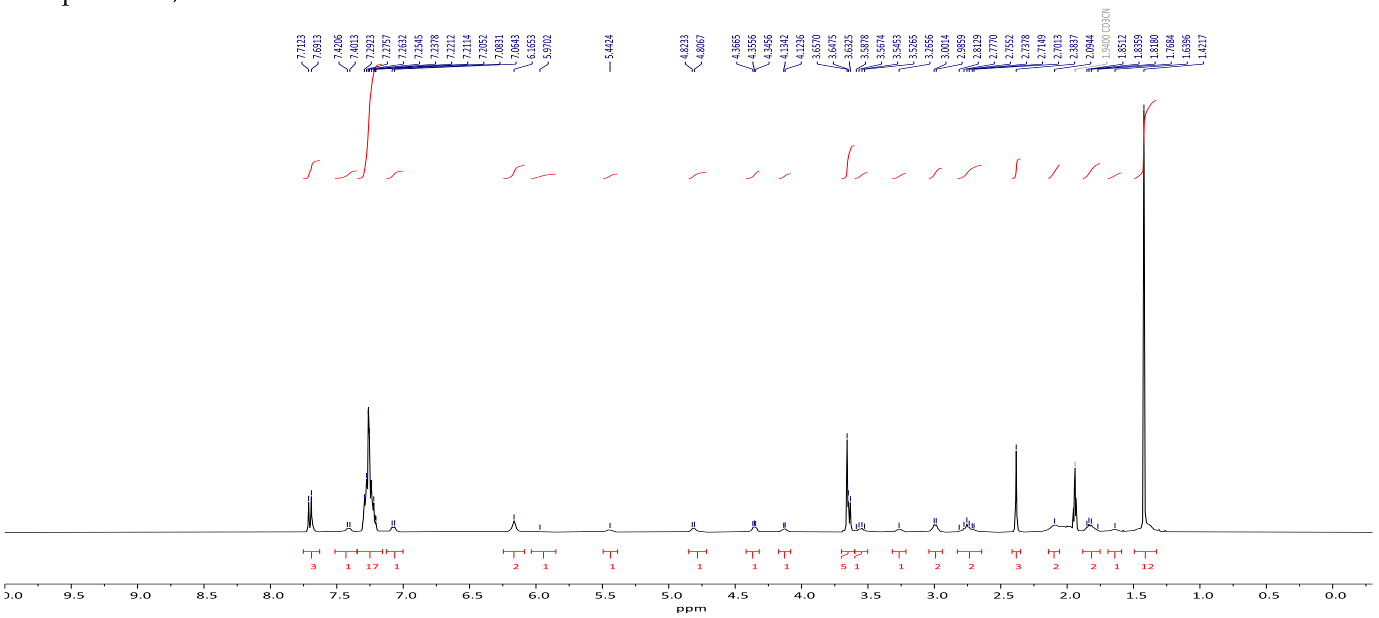

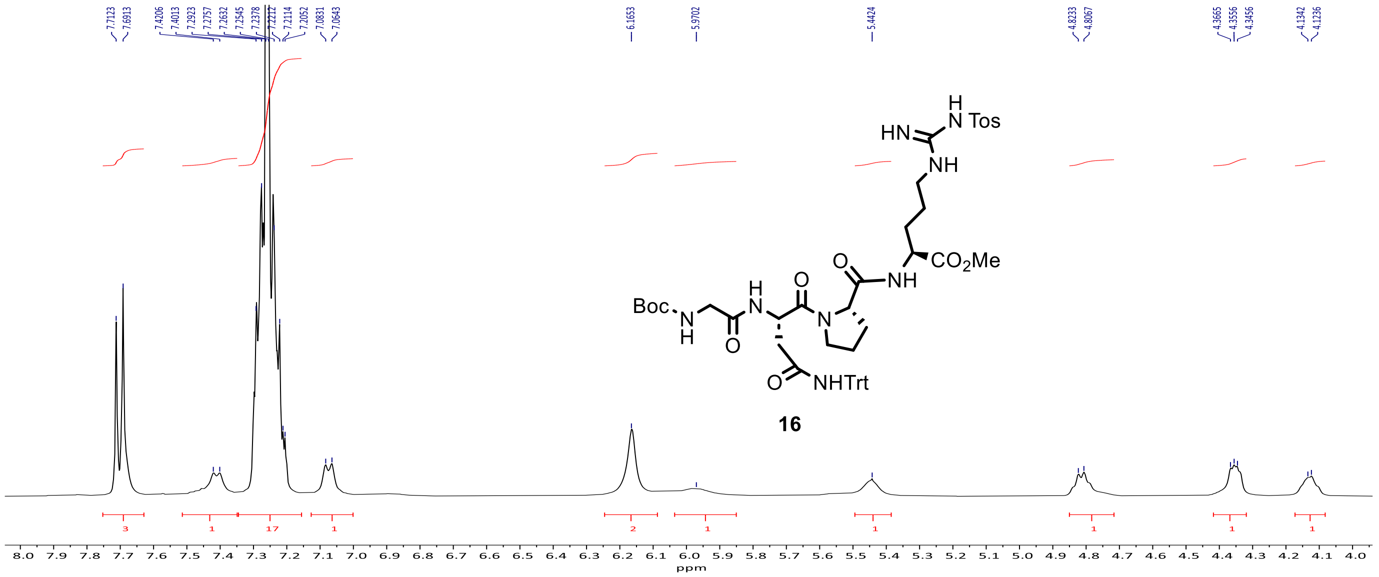

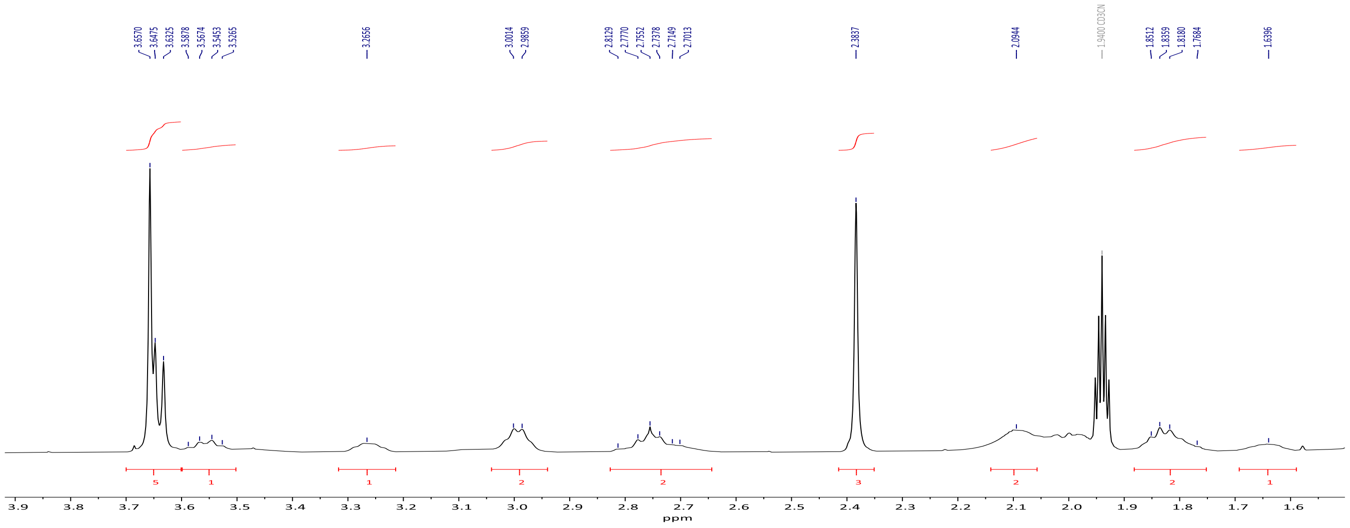

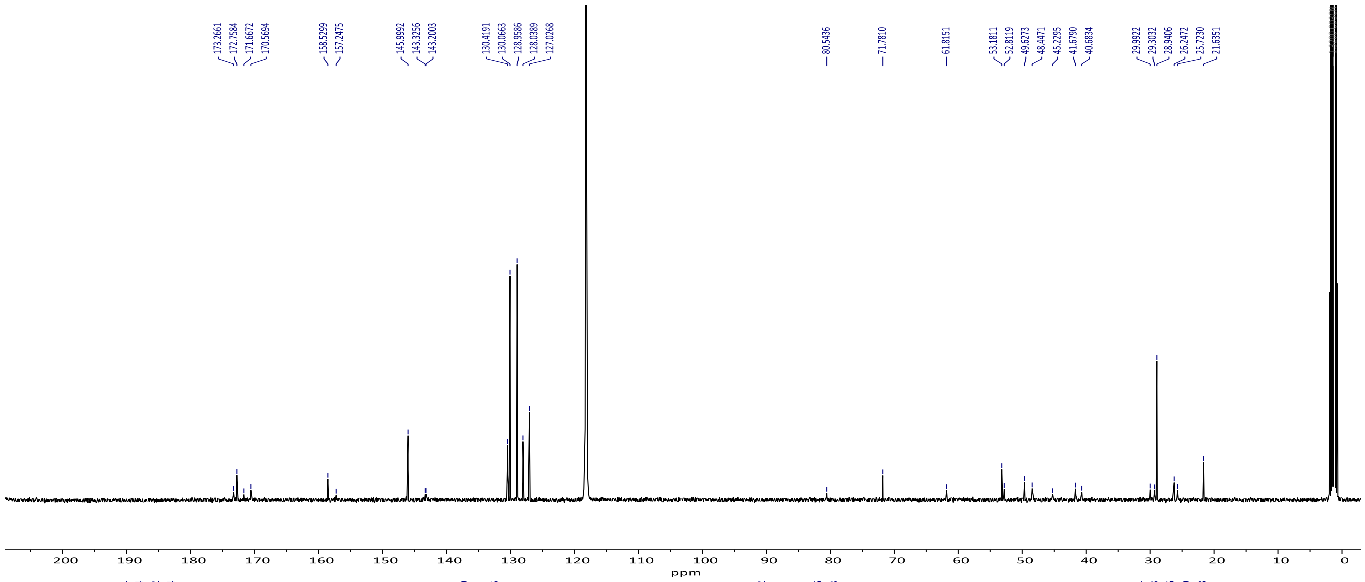

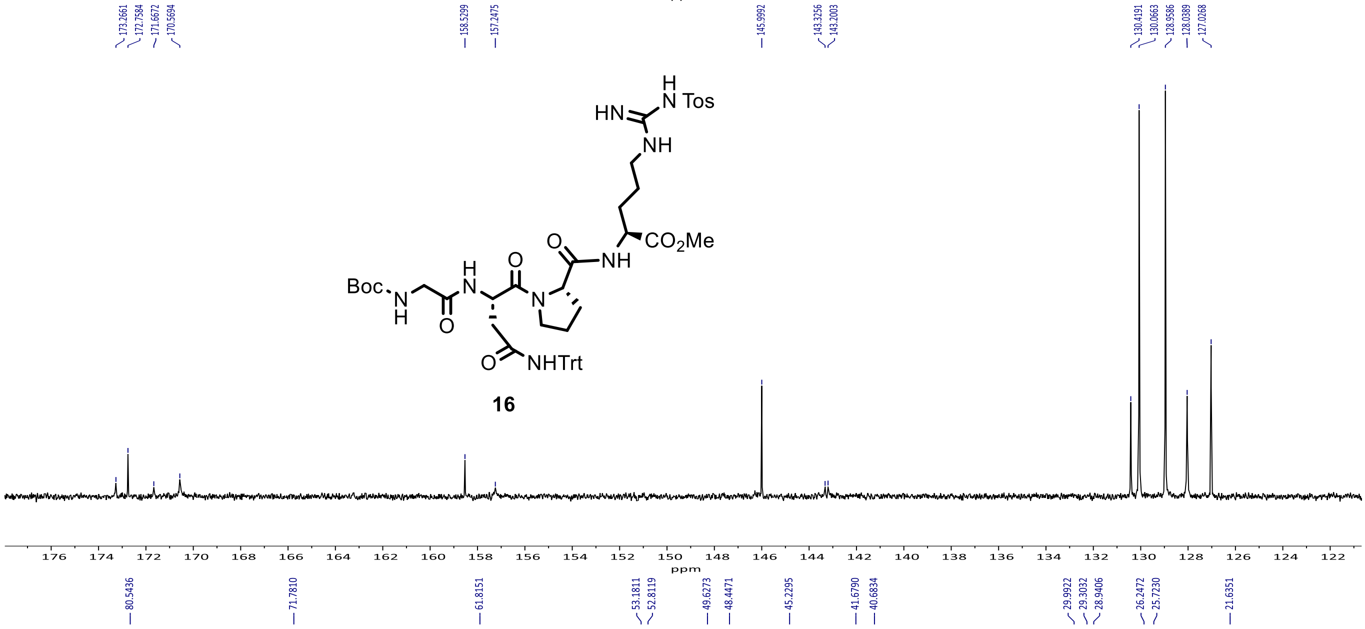

16

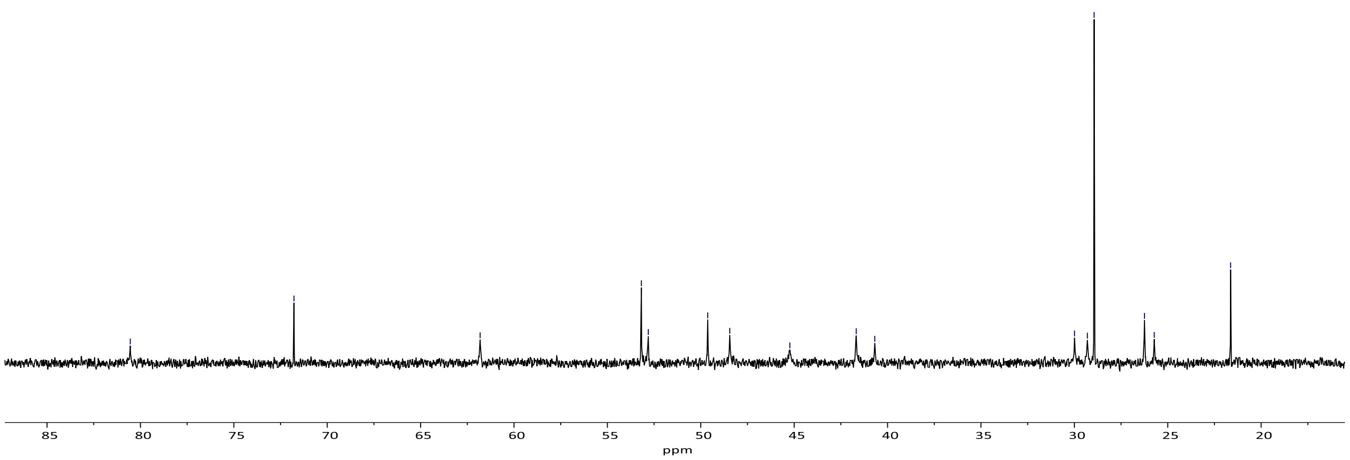

Compound **17**, ^{1}H and ^{13}C NMR at 50°C in CD_3OD

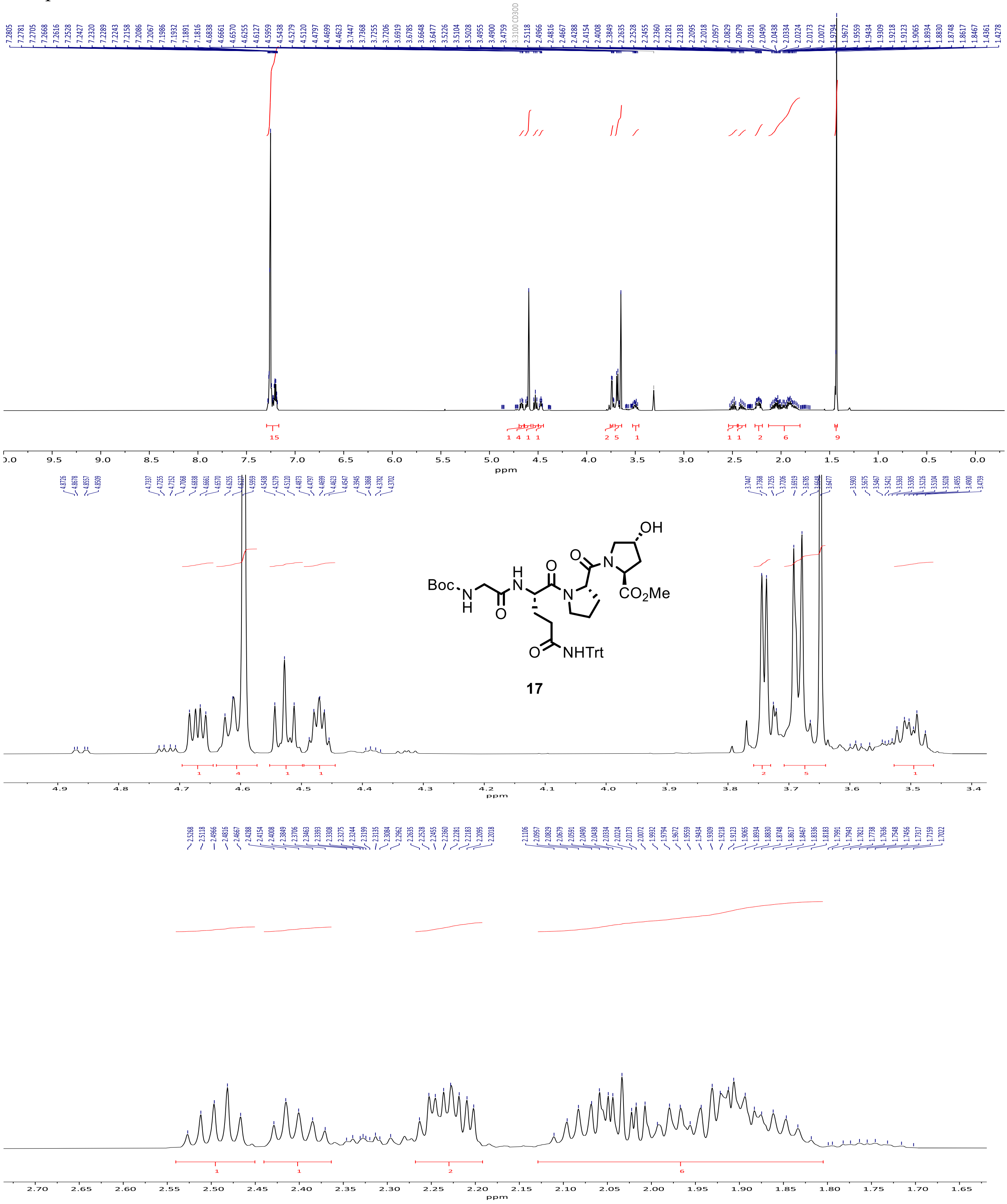

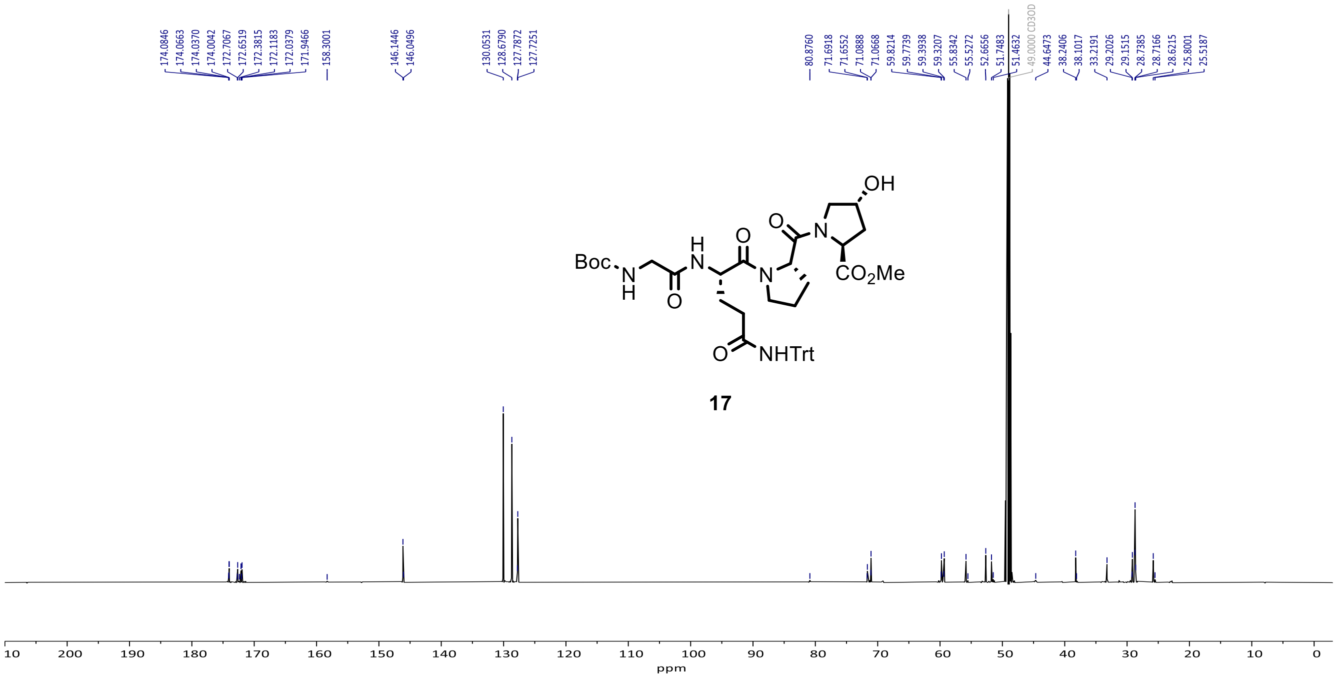
174.0846
174.0663
174.0370
174.0042
172.7067
172.6519
172.3815
172.1183
172.0379
171.9466
158.3001
146.1446
146.0496
130.0531
128.6790
127.7872
127.7251
80.8760
71.6918
71.6552
71.0888
71.0668
59.8214
59.7739
59.3938
59.3207
55.8342
55.5272
52.6656
51.7483
51.4632
49.0000 CD3OD
44.6473
38.2406
38.1017
33.2191
29.2026
29.1515
28.7385
28.7166
28.6215
25.8001
25.5187
OH
O
N
Boc
N
H
H
N
O
O
N
CO2Me
O
NHTrt
ppm

17

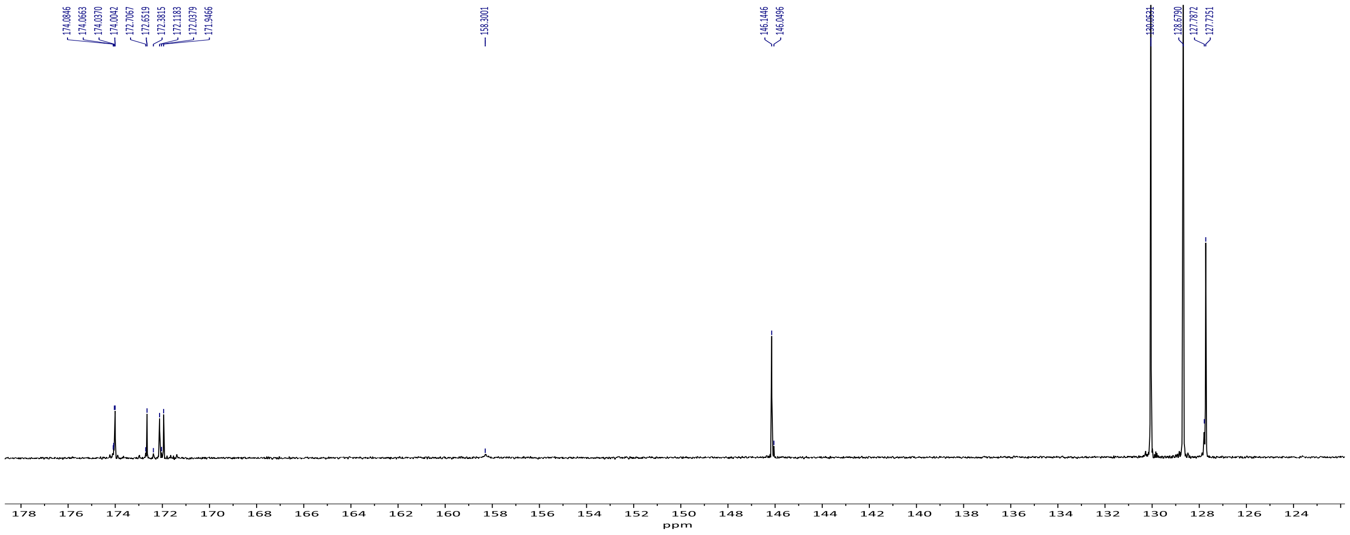
ppm

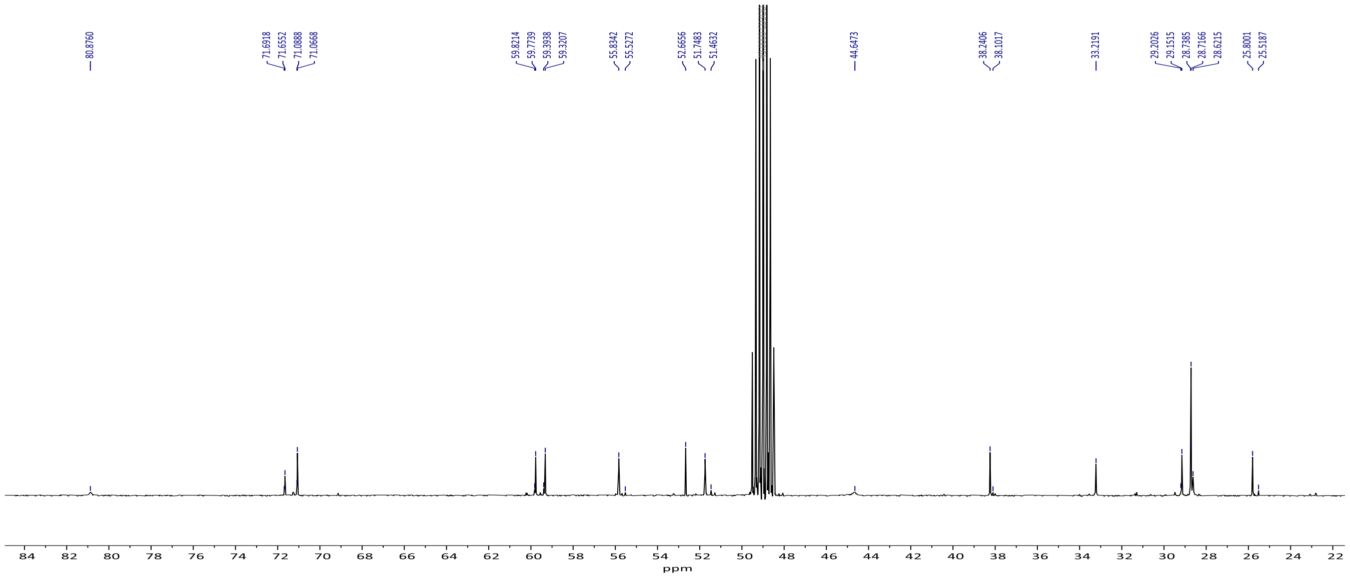
ppm

Compound **18**, ^{1}H and ^{13}C NMR at 70°C in CD_3CN

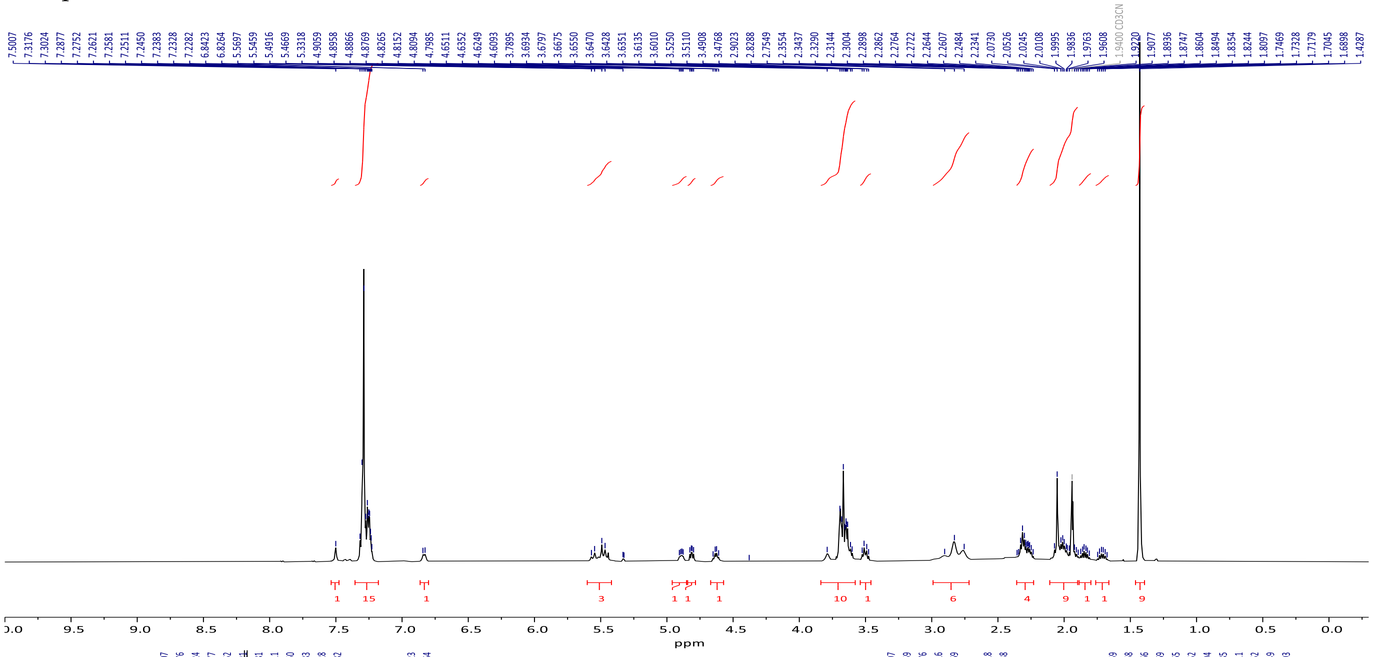

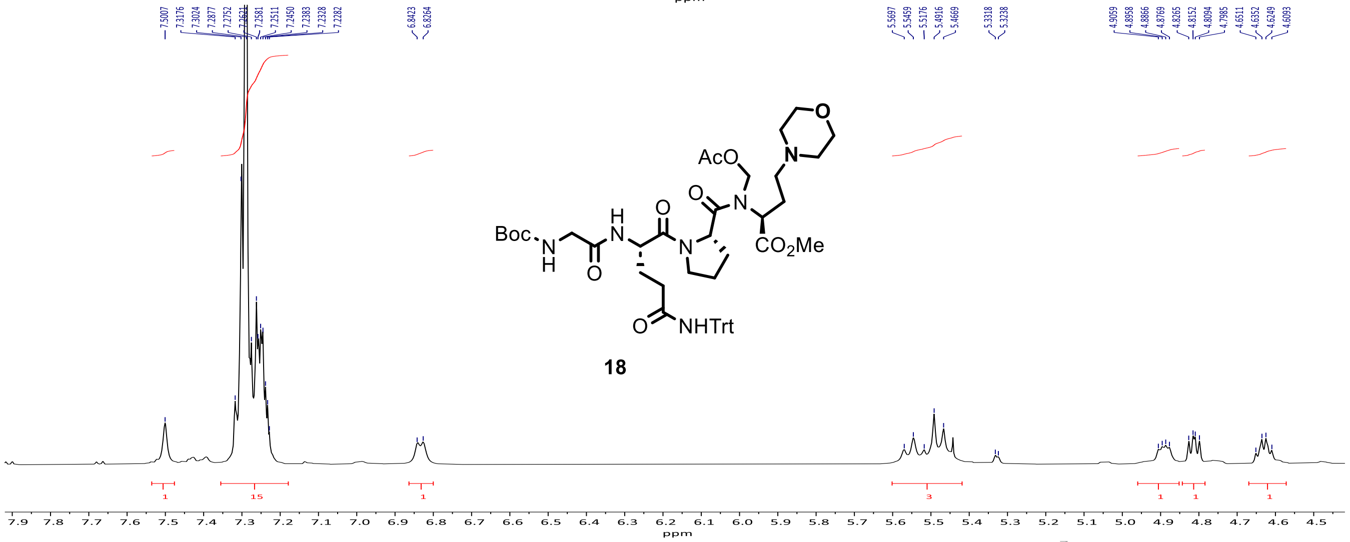

18

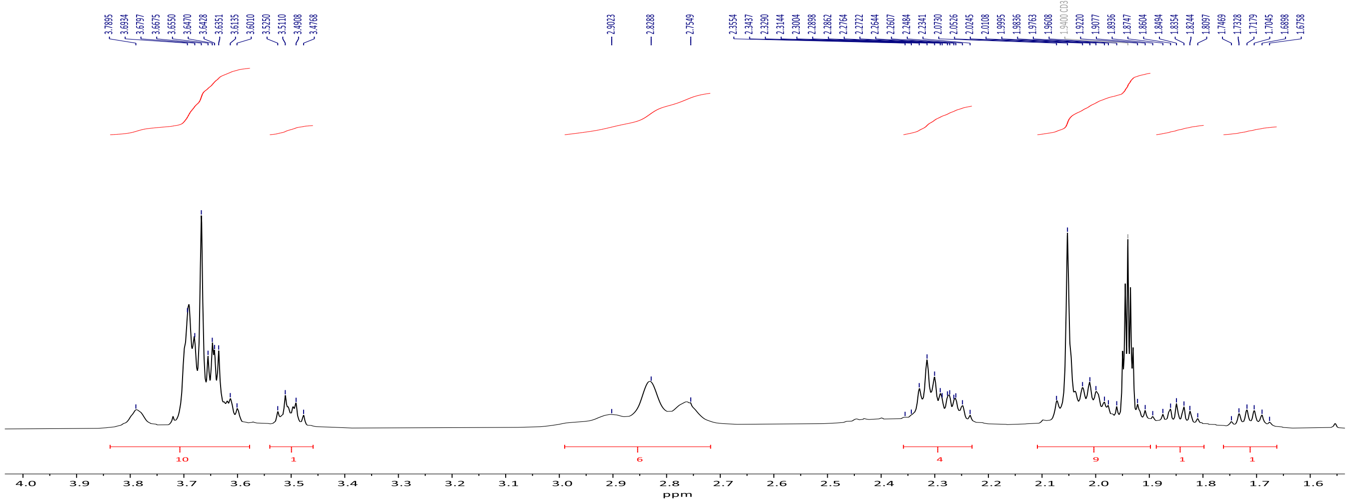

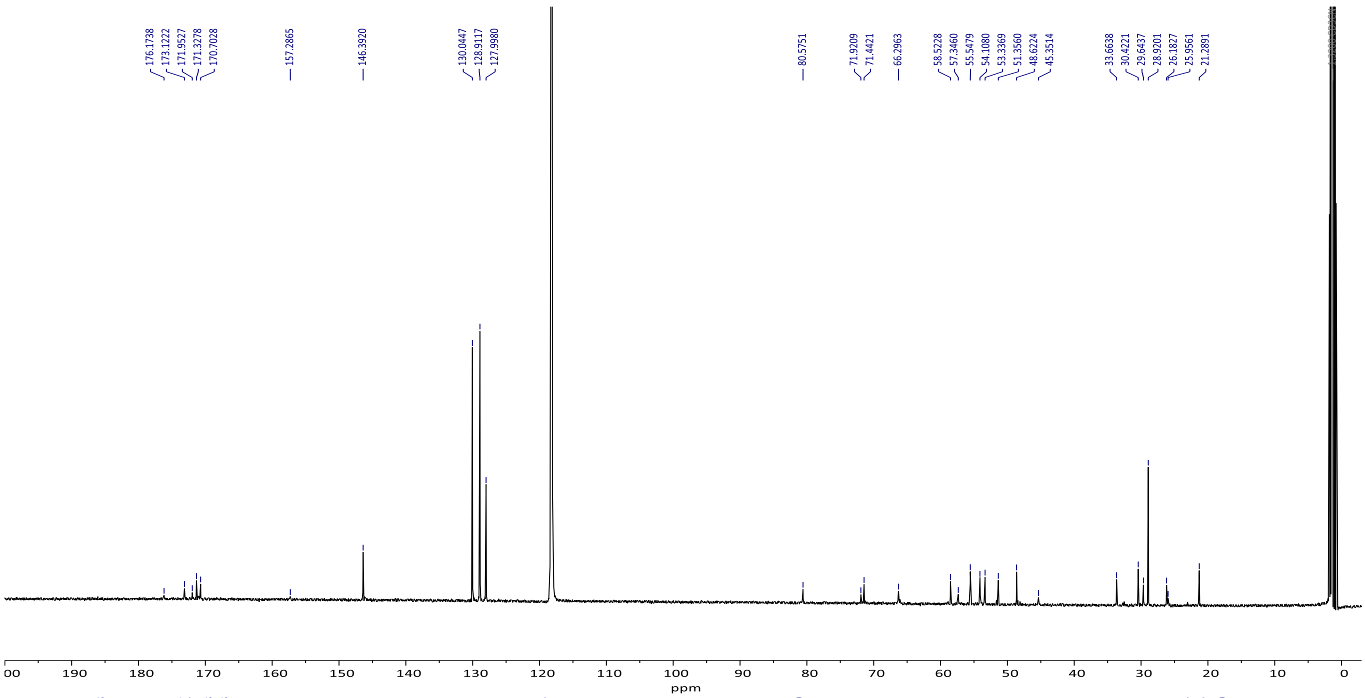
176.1738
173.1222
171.9527
171.3278
170.7028
157.2865
146.3920
130.0447
128.9117
127.9980
80.5751
71.9209
71.4421
66.2963
58.5228
57.3460
55.5479
54.1080
53.3369
51.3560
48.6224
45.3514
33.6638
30.4221
29.6437
28.9201
26.1827
25.9561
21.2891
ppm

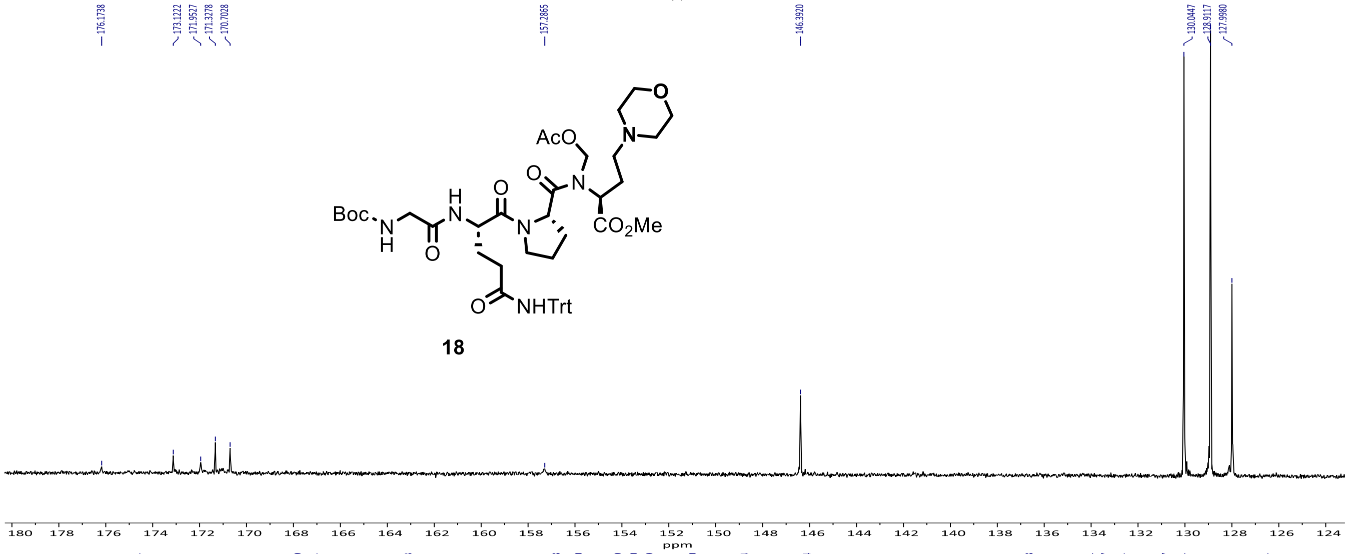
176.1738
173.1222
171.9527
171.3278
170.7028
157.2865
146.3920
130.0447
128.9117
127.9980
ppm
Boc
N
H
O
AcO
CO2Me
NHTrt

18

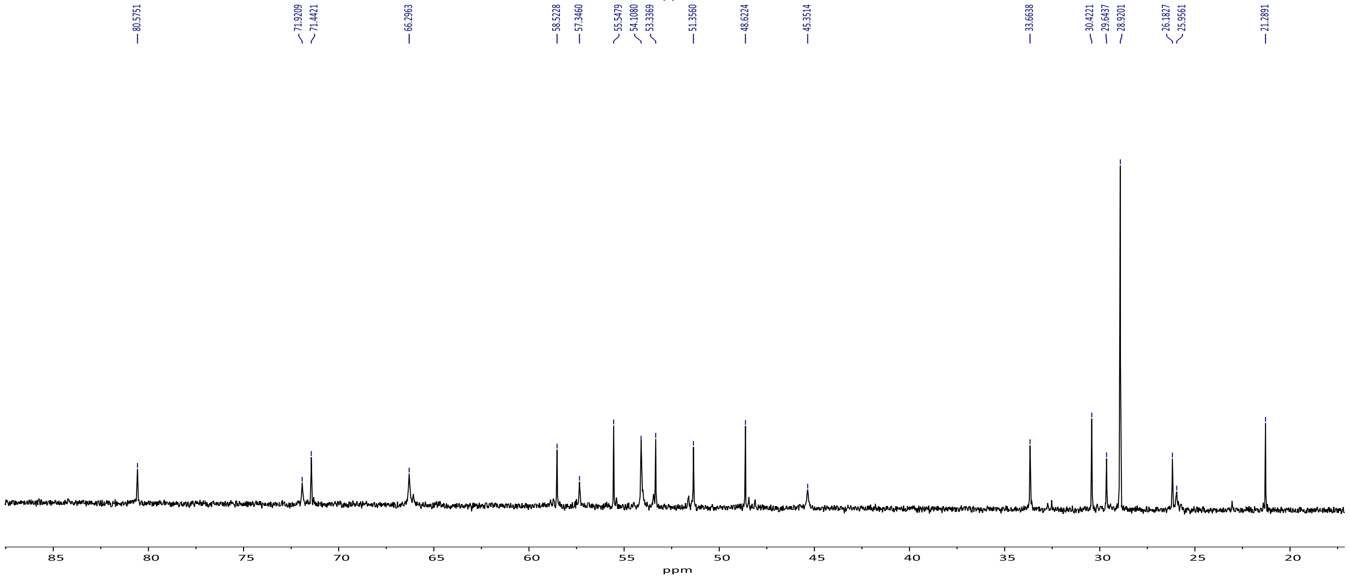
80.5751
71.9209
71.4421
66.2963
58.5228
57.3460
55.5479
54.1080
53.3369
51.3560
48.6224
45.3514
33.6638
30.4221
29.6437
28.9201
26.1827
25.9561
21.2891
ppm

Compound **18**, HSQC and COSY at 70°C in CD_3CN

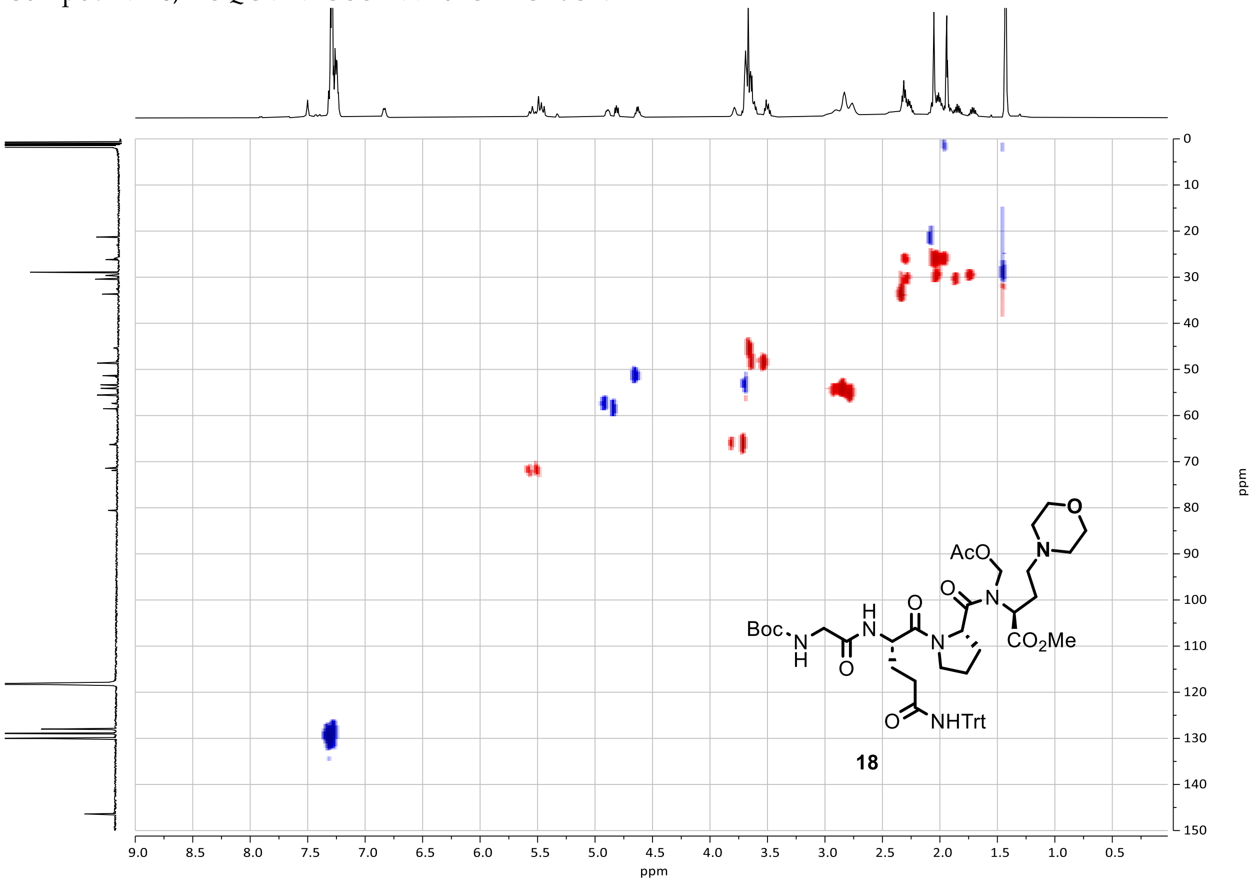

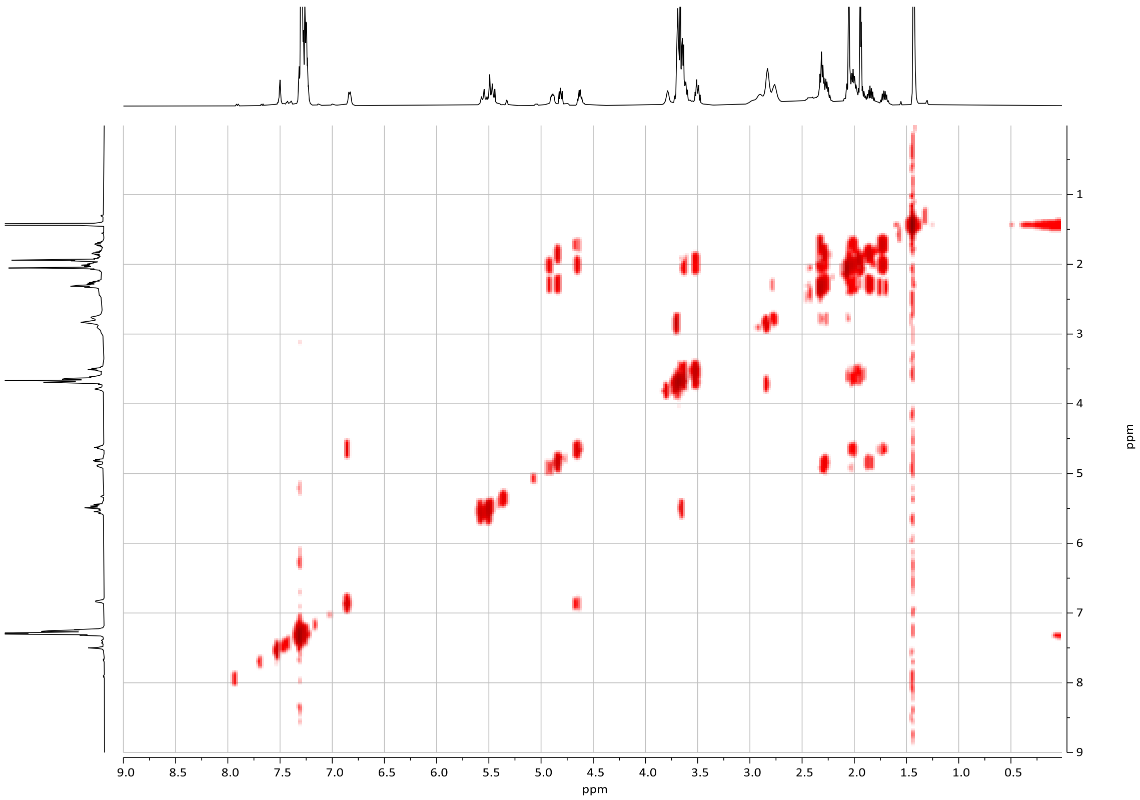

Compound **19**, ^{1}H and ^{13}C NMR at 70°C in CD_3CN

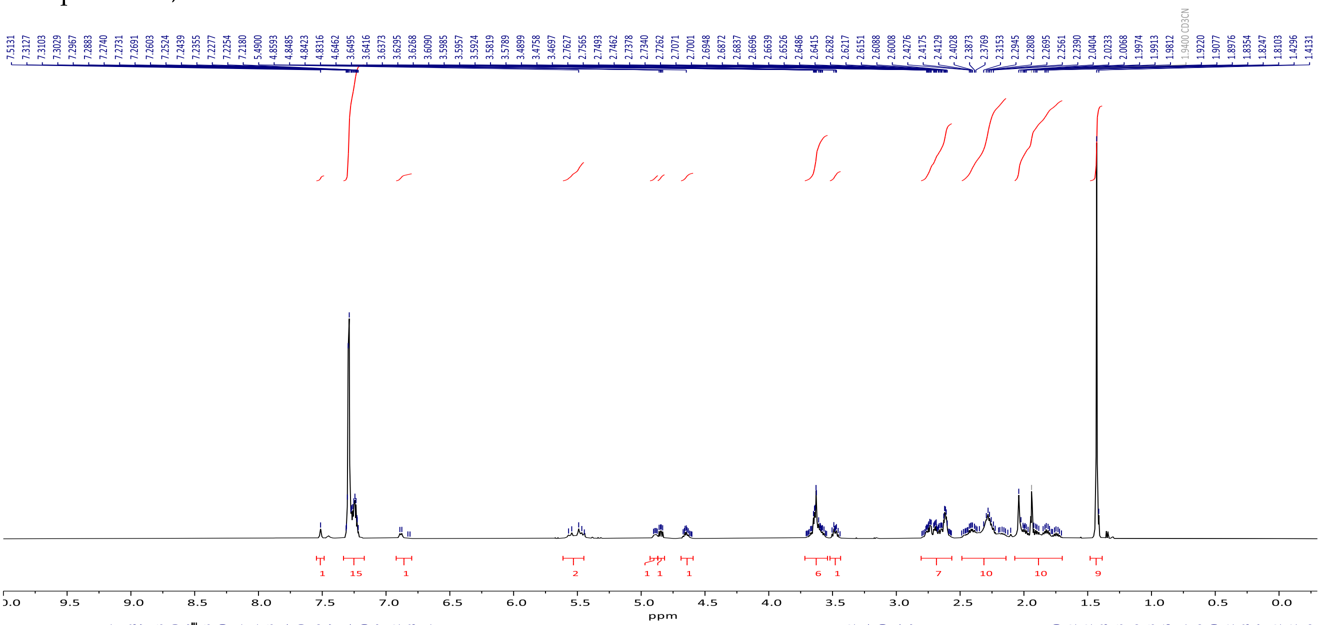

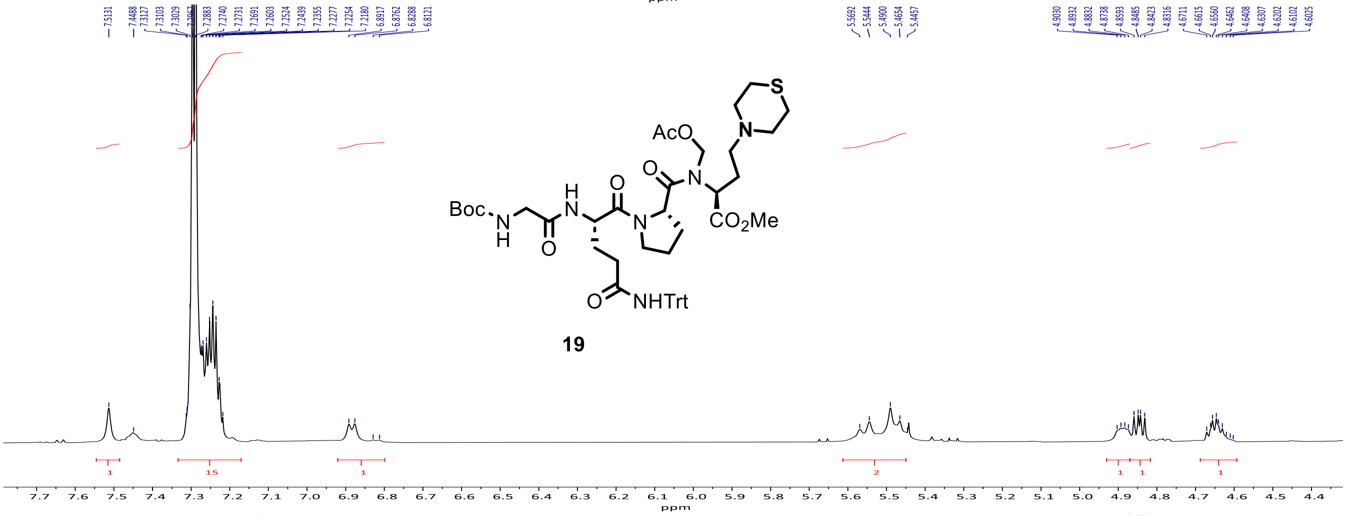

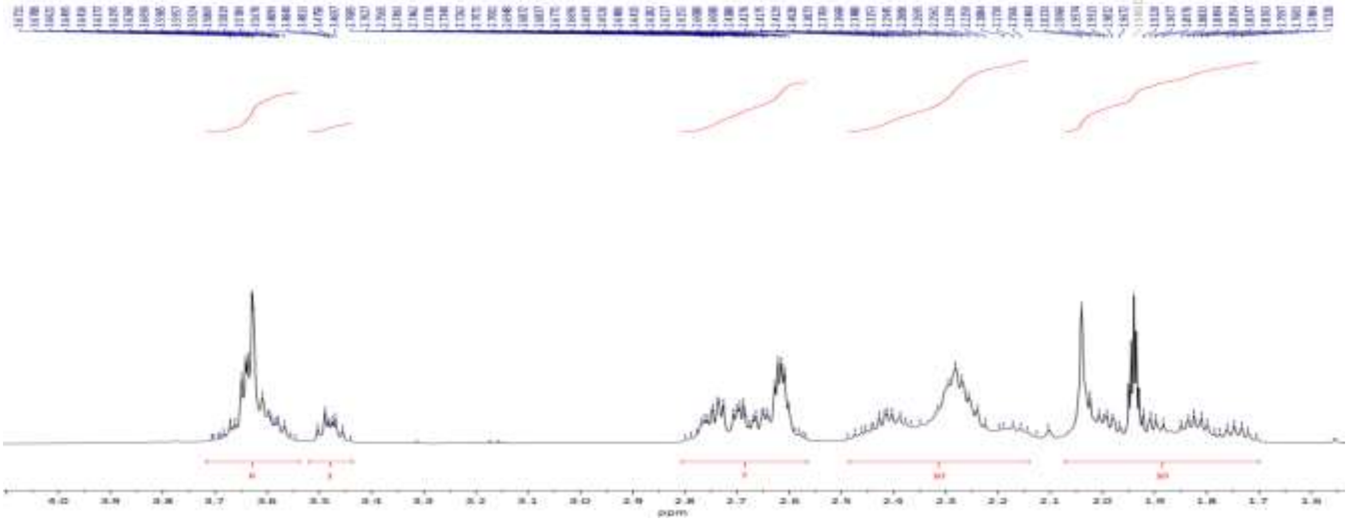

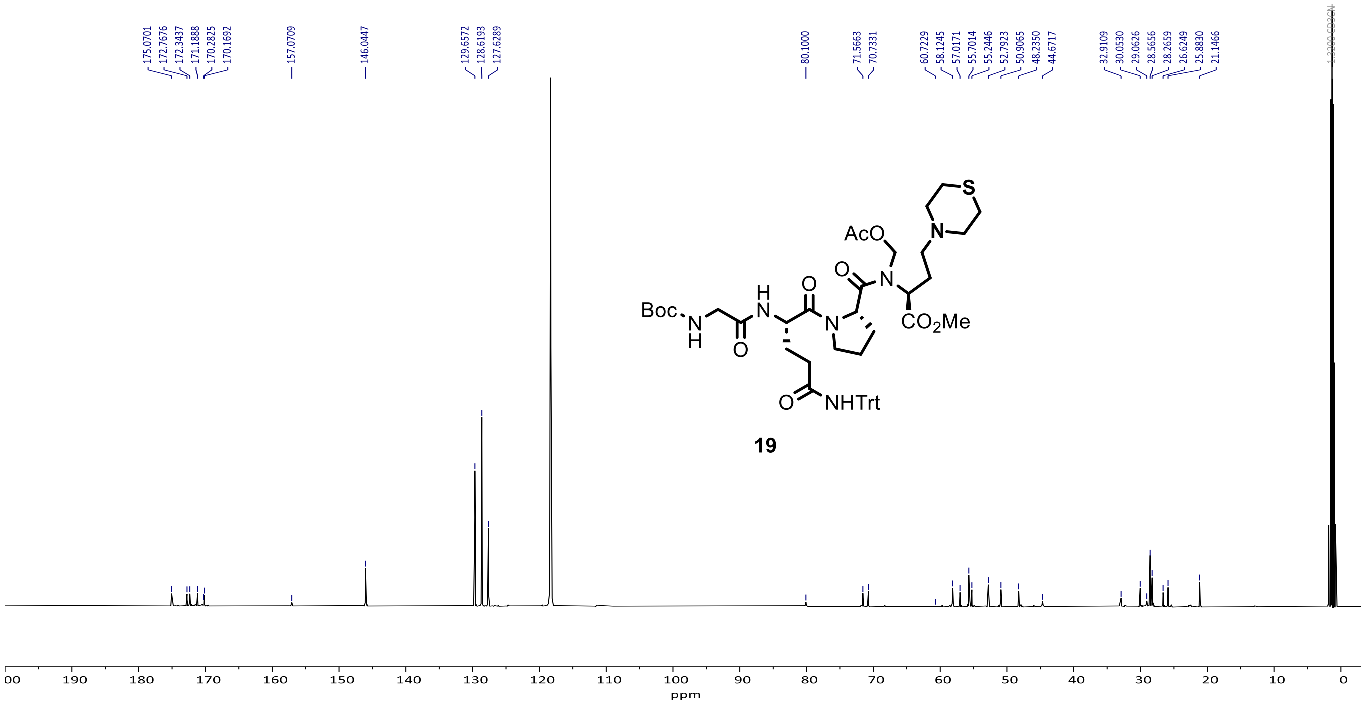
175.0701
172.7676
172.3437
171.1888
170.2825
170.1692
157.0709
146.0447
129.6572
128.6193
127.6289
80.1000
71.5663
70.7331
60.7229
58.1245
57.0171
55.7014
55.2446
52.7923
50.9065
48.2350
44.6717
32.9109
30.0530
29.0626
28.5656
28.2659
26.6249
25.8830
21.1466
Boc
N
H
H
N
O
O
N
O
N
AcO
N
S
CO2Me
O
NHTrt
19
ppm

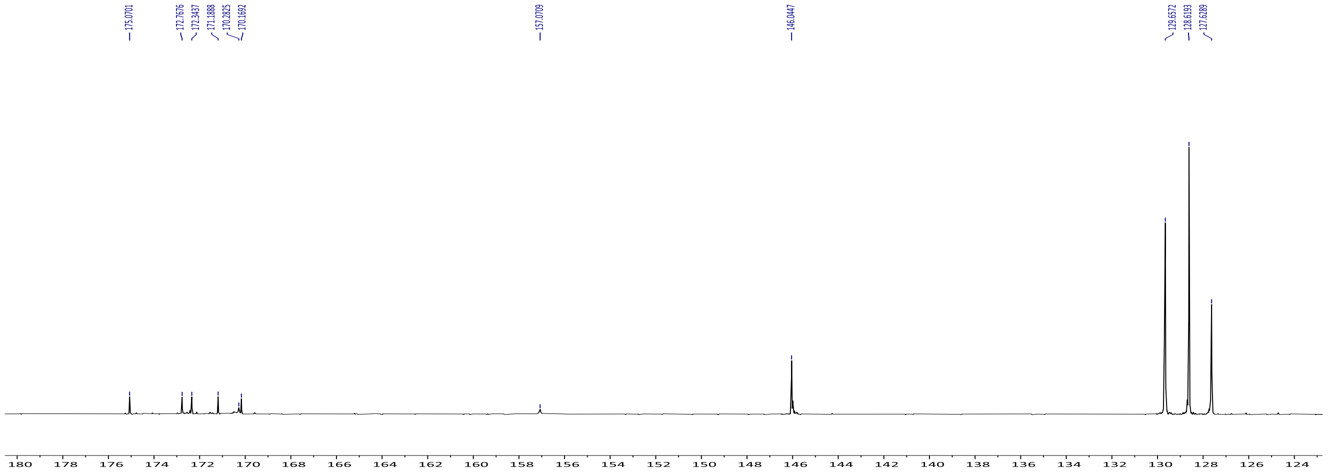
175.0701
172.7676
172.3437
171.1888
170.2825
170.1692
157.0709
146.0447
129.6572
128.6193
127.6289
ppm

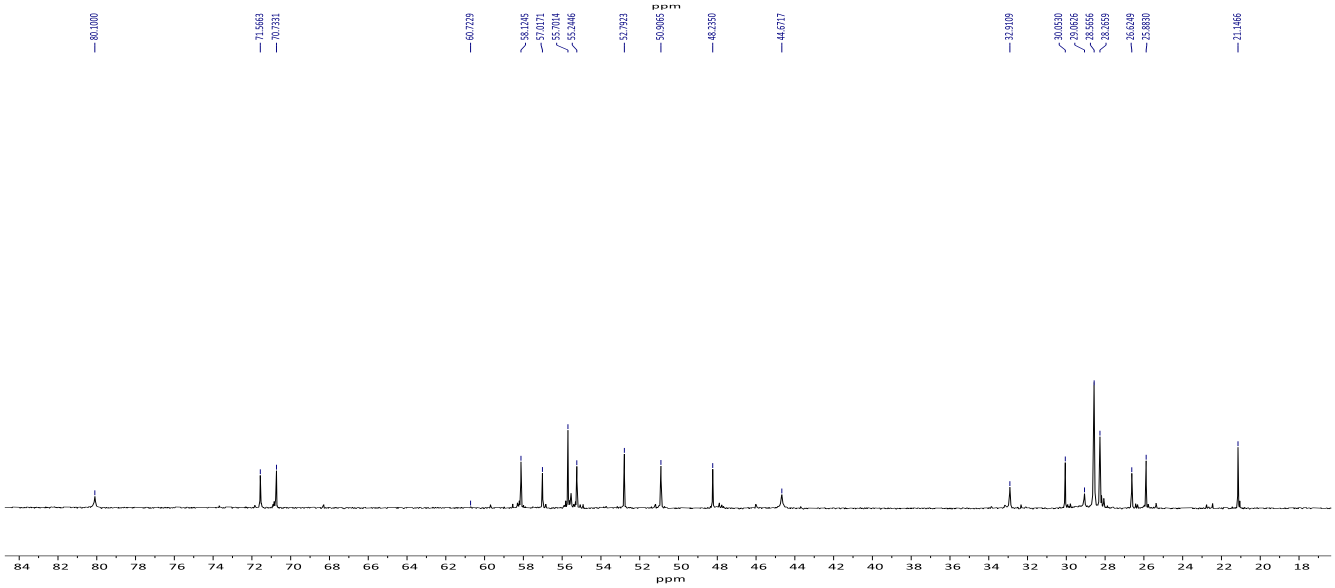
80.1000
71.5663
70.7331
60.7229
58.1245
57.0171
55.7014
55.2446
52.7923
50.9065
48.2350
44.6717
32.9109
30.0530
29.0626
28.5656
28.2659
26.6249
25.8830
21.1466
ppm

Compound **20**, ^{1}H and ^{13}C NMR at 50°C in CD_3OD

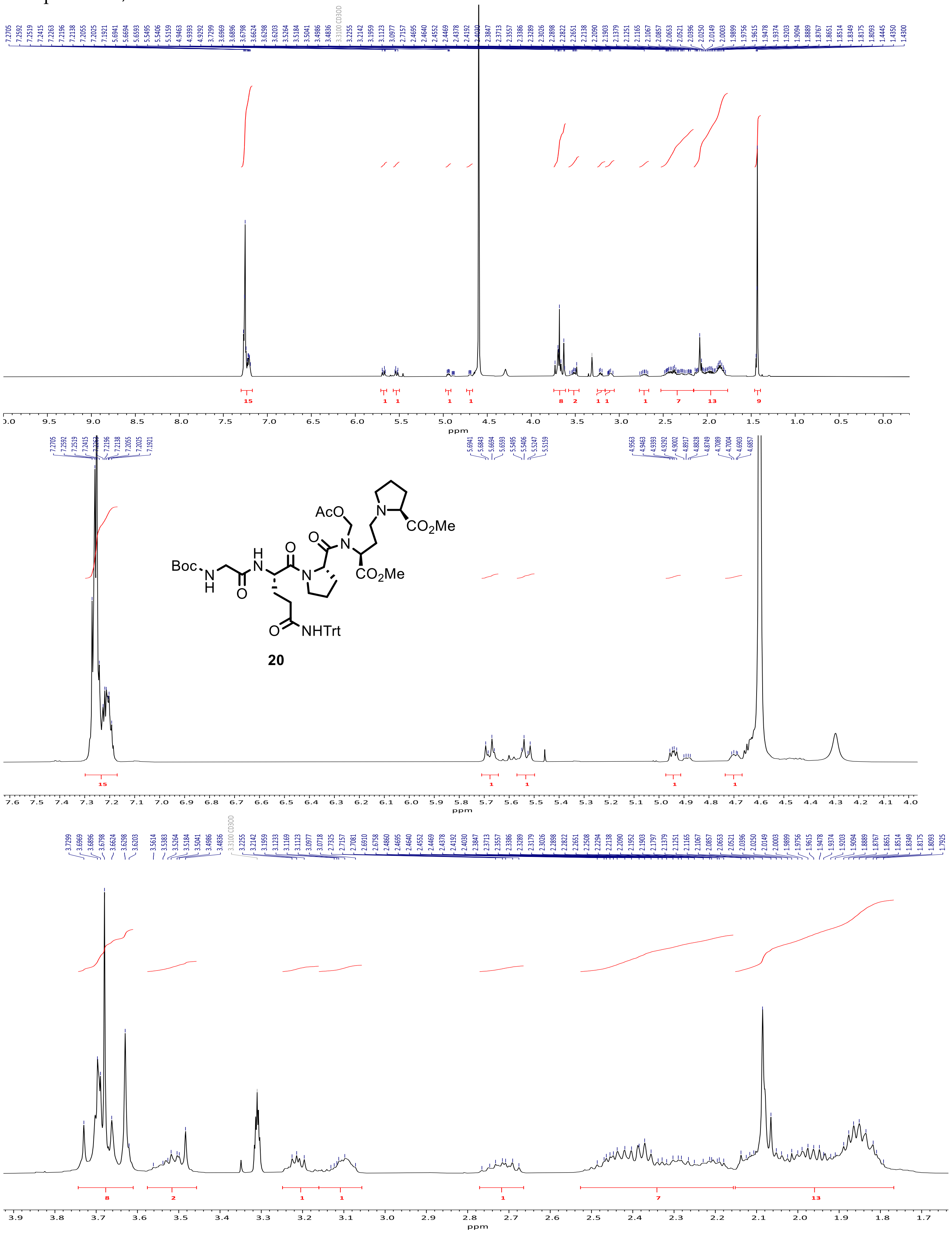

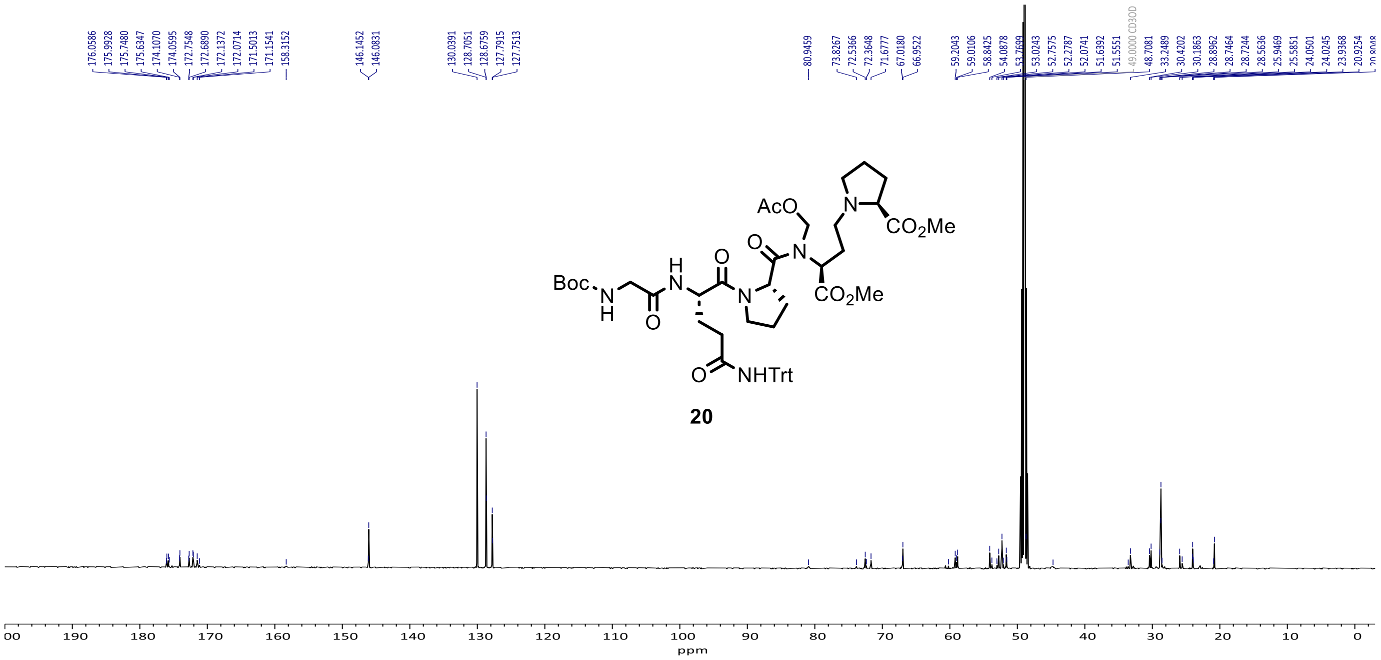
AcO
N
CO2Me
O
N
Boc
N
H
H
N
O
O
N
CO2Me
O
NHTrt
20
ppm

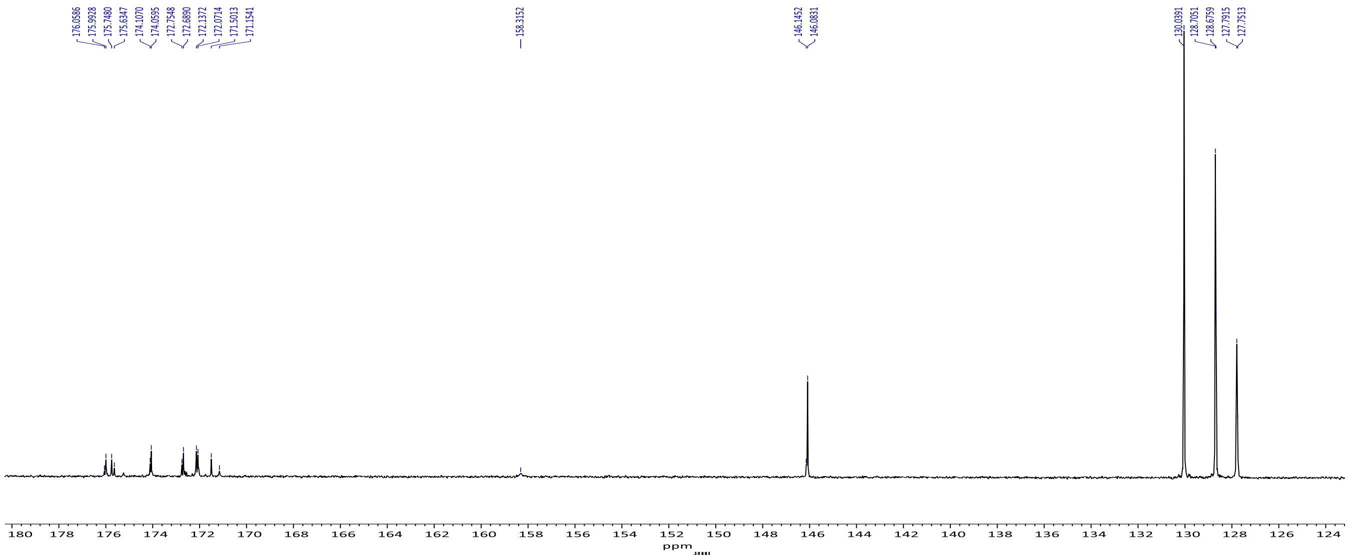
ppm

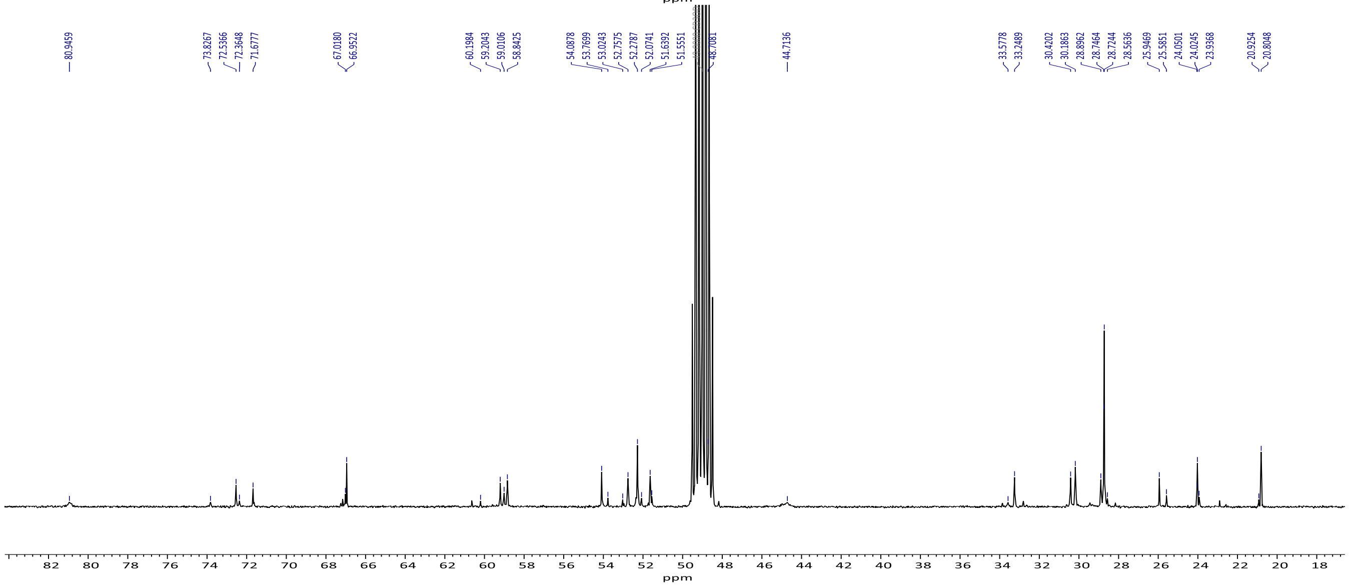
ppm

Compound **21**, ^{1}H and ^{13}C NMR at 50°C in CD_3OD

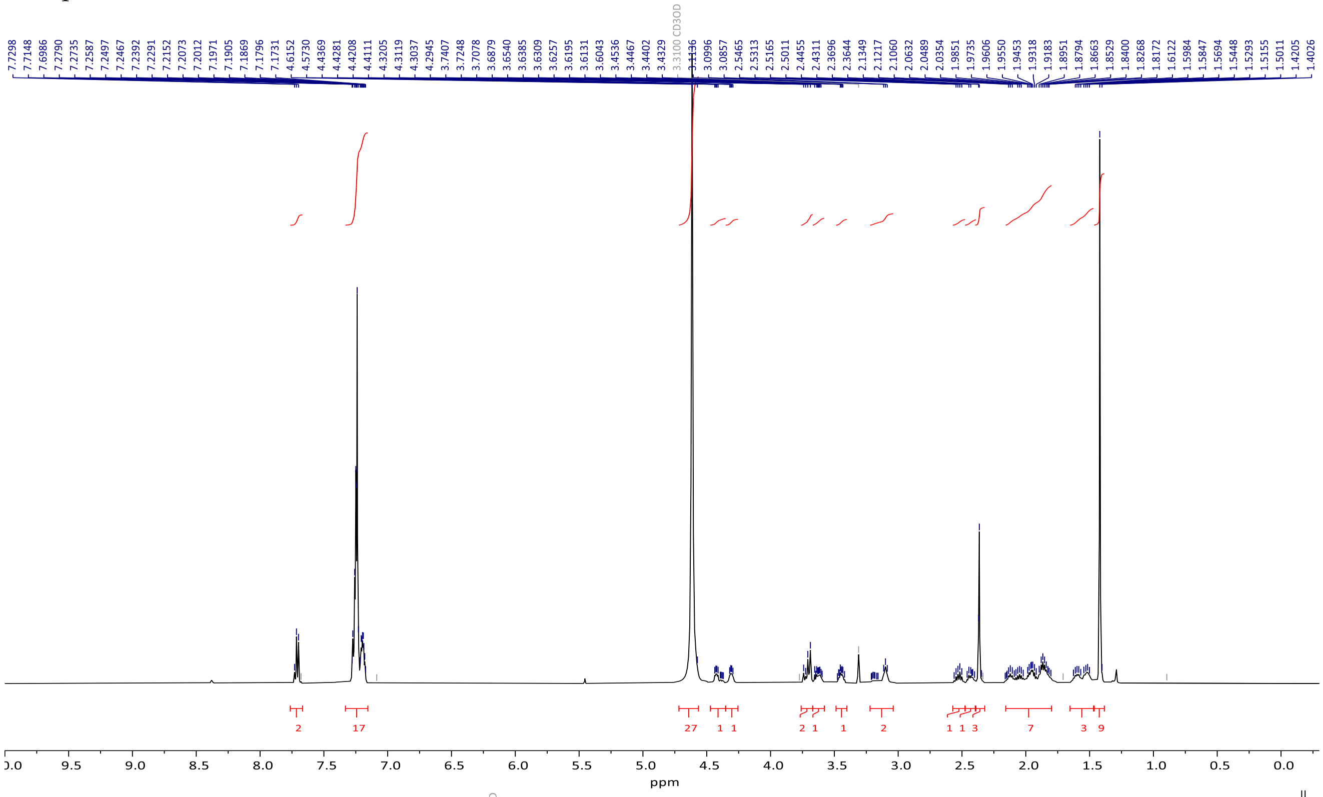

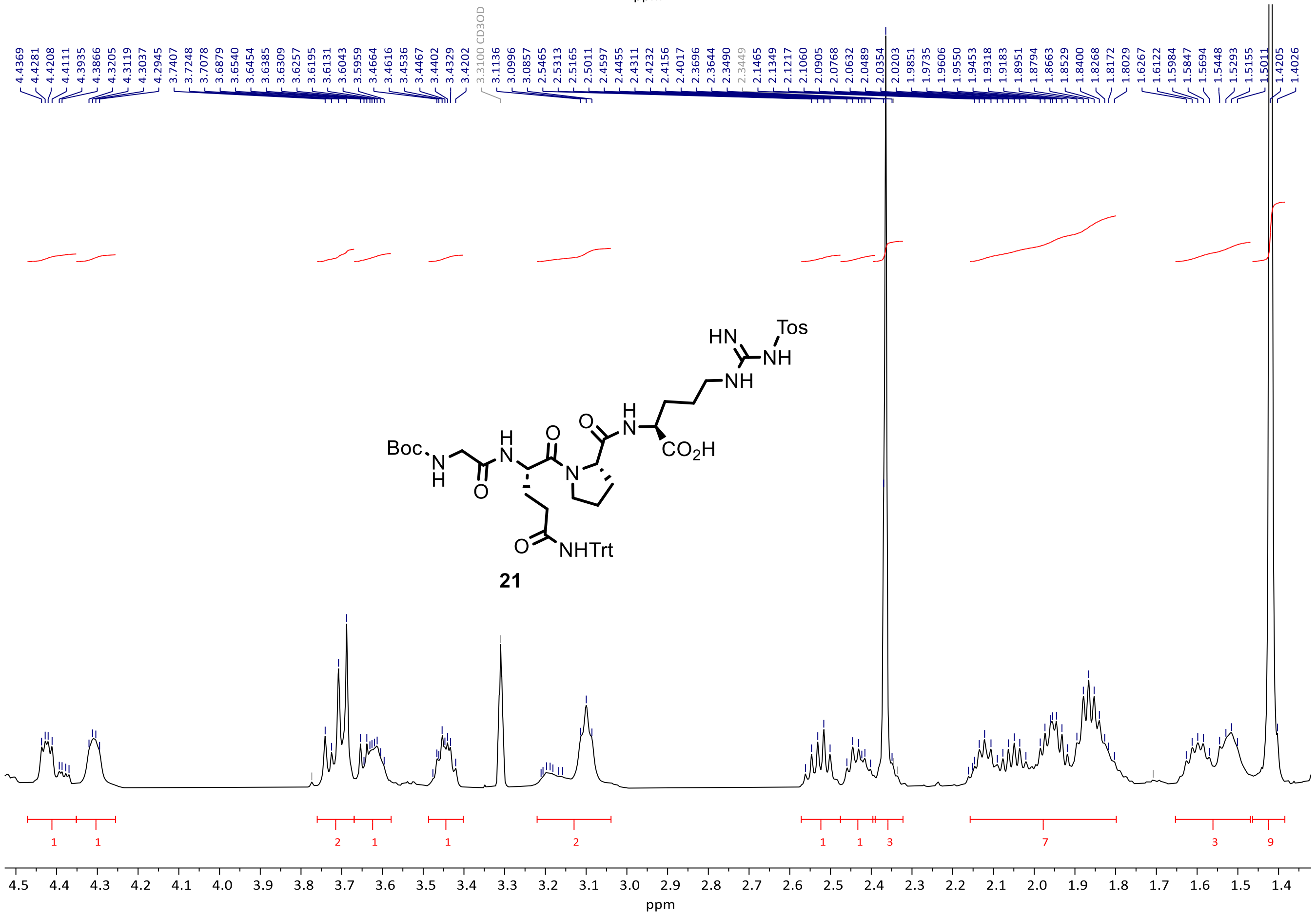

21

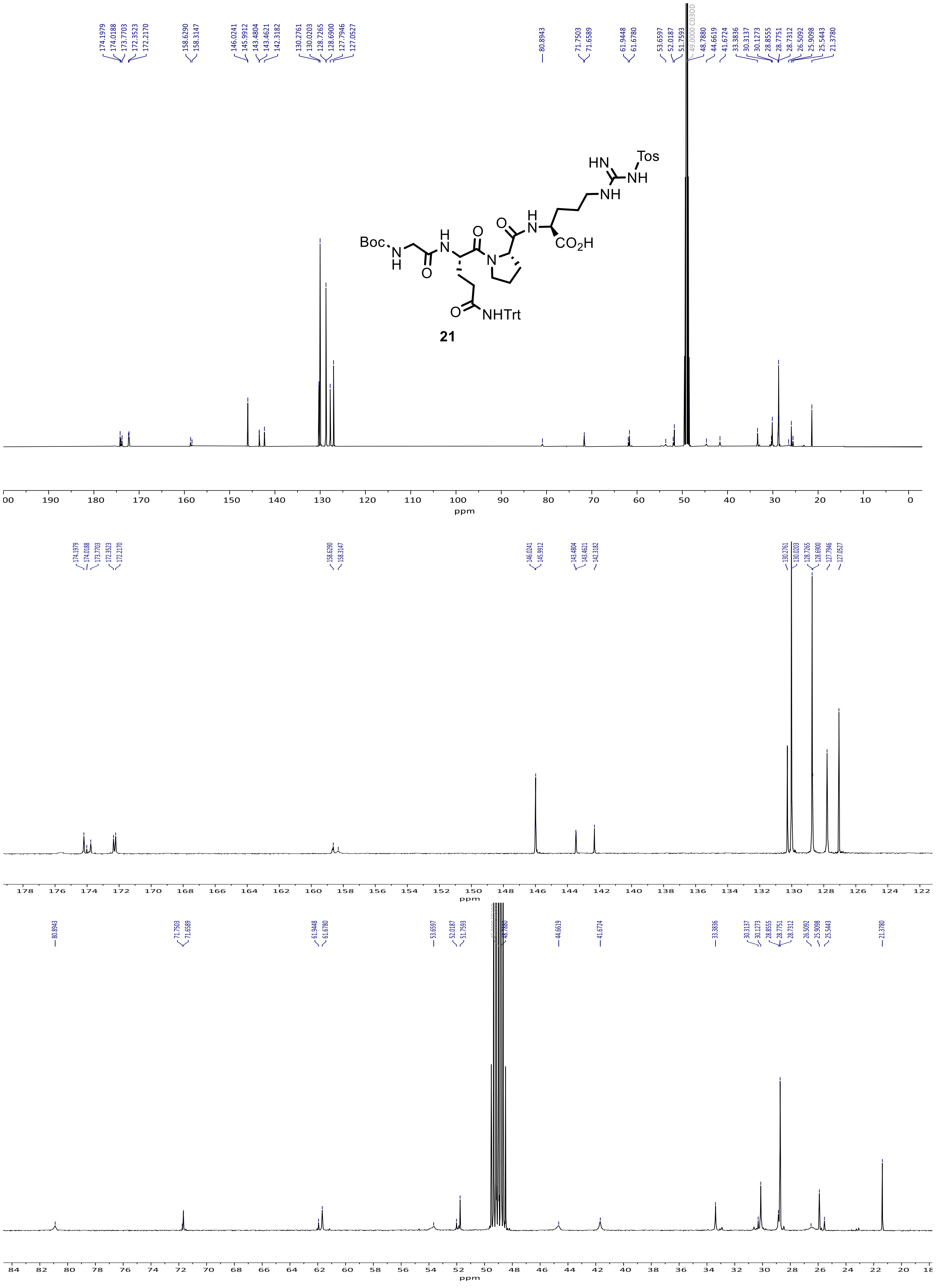
174.1979
174.0188
173.7703
172.3523
172.2170
158.6290
158.3147
146.0241
145.9912
143.4804
143.4621
142.3182
130.2761
130.0203
128.7265
128.6900
127.7946
127.0527
80.8943
71.7503
71.6589
61.9448
61.6780
53.6597
52.0187
51.7593
49.0000 CD3OD
48.7880
44.6619
41.6724
33.3836
30.3137
30.1273
28.8555
28.7751
28.7312
26.5092
25.9098
25.5443
21.3780
Tos
HN
NH
NH
H
O
N
H
N
CO2H
Boc
N
H
O
N
O
O
NHTrt
21
ppm

Compound **22**, ^{1}H and ^{13}C NMR at 50°C in CD_3OD

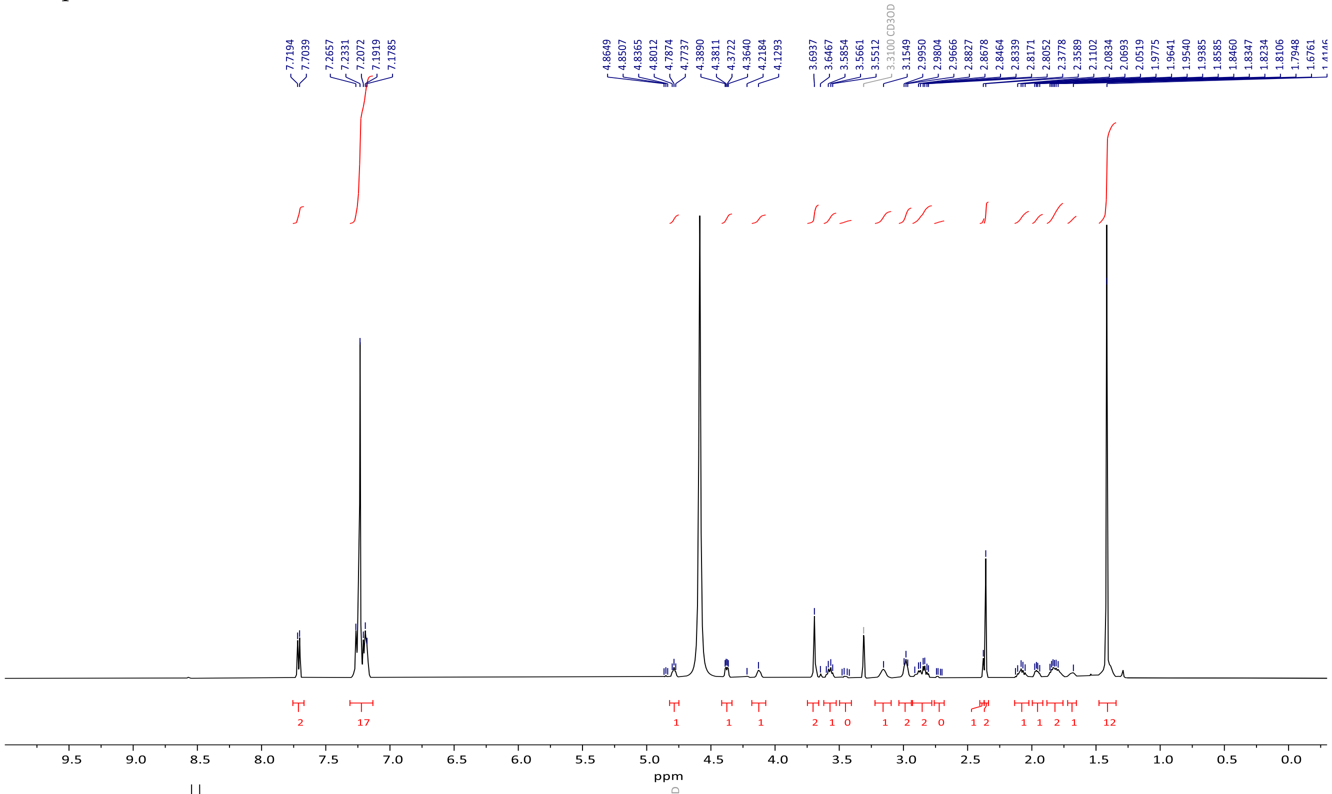

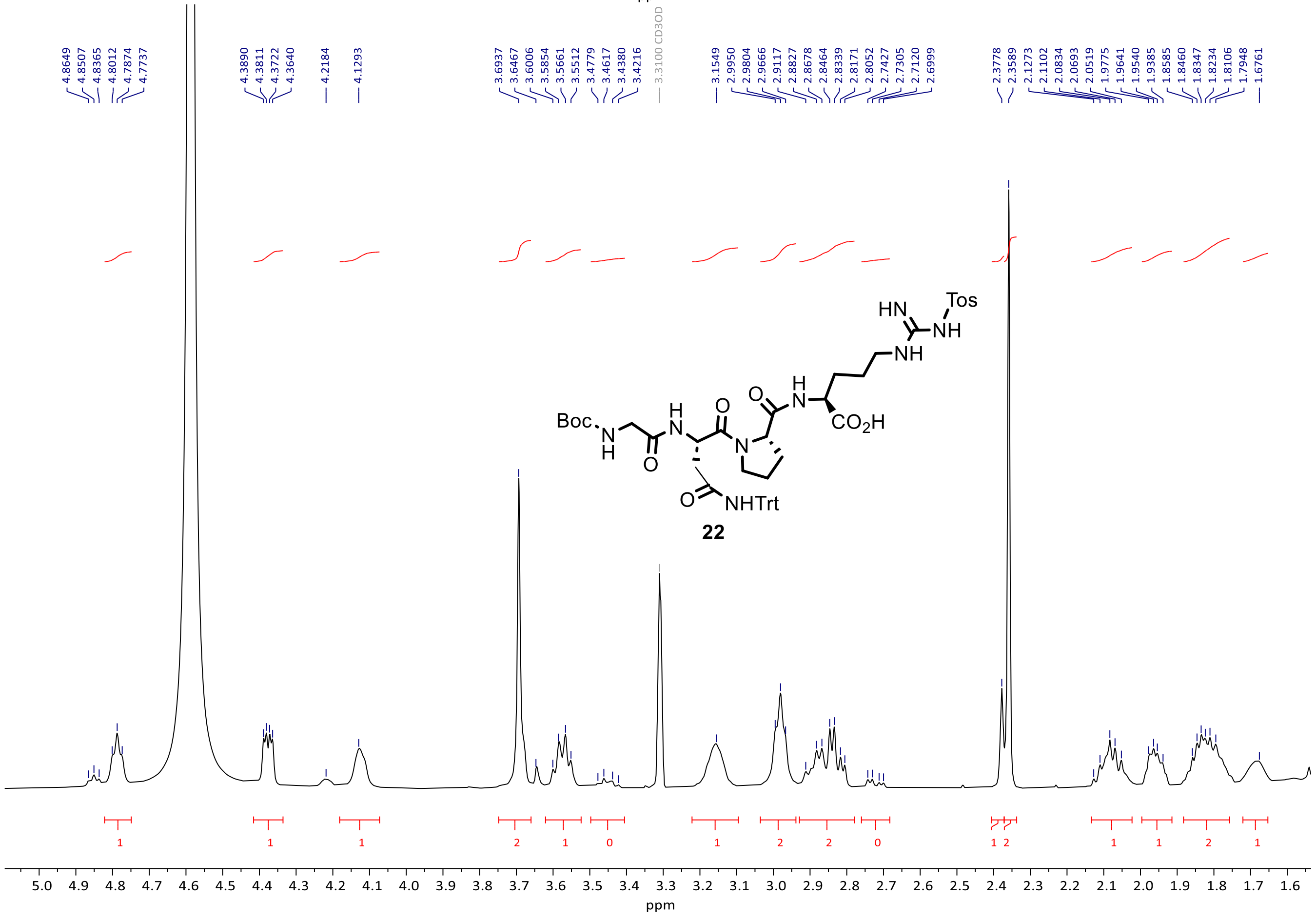

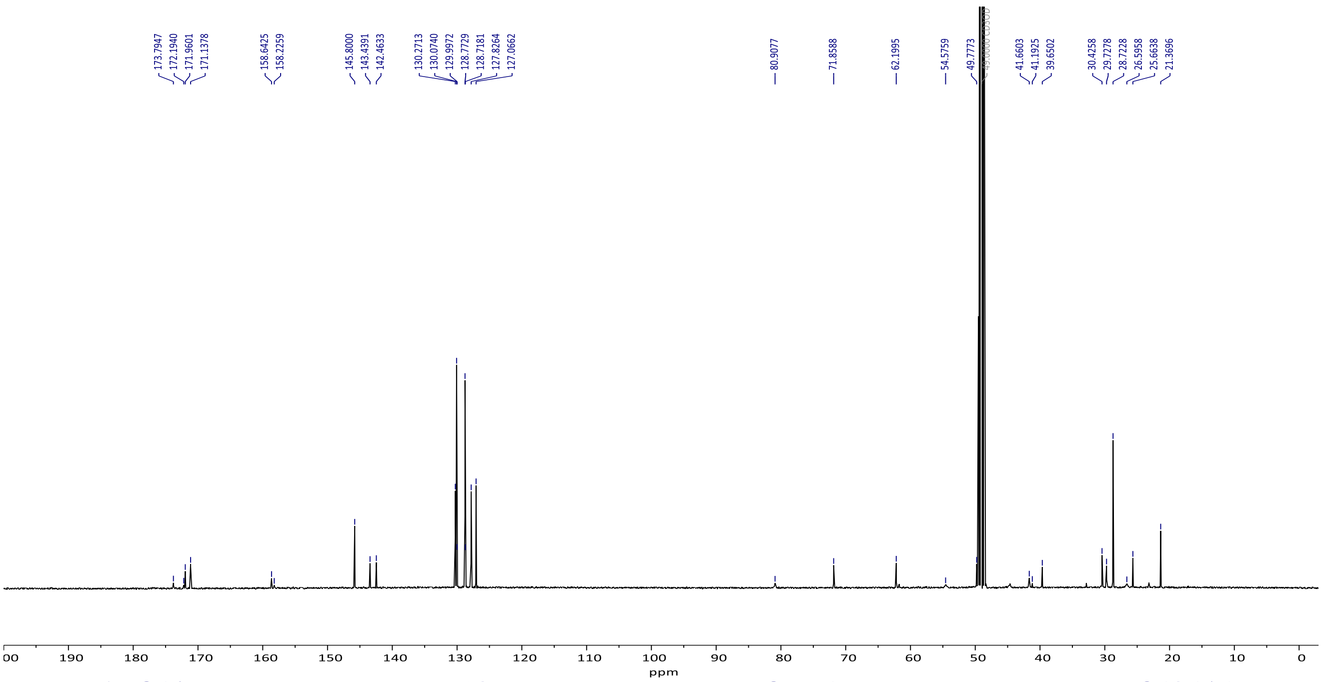
173.7947
172.1940
171.9601
171.1378
158.6425
158.2259
145.8000
143.4391
142.4633
130.2713
130.0740
129.9972
128.7729
128.7181
127.8264
127.0662
80.9077
71.8588
62.1995
54.5759
49.7773
41.6603
41.1925
39.6502
30.4258
29.7278
28.7228
26.5958
25.6638
21.3696
ppm

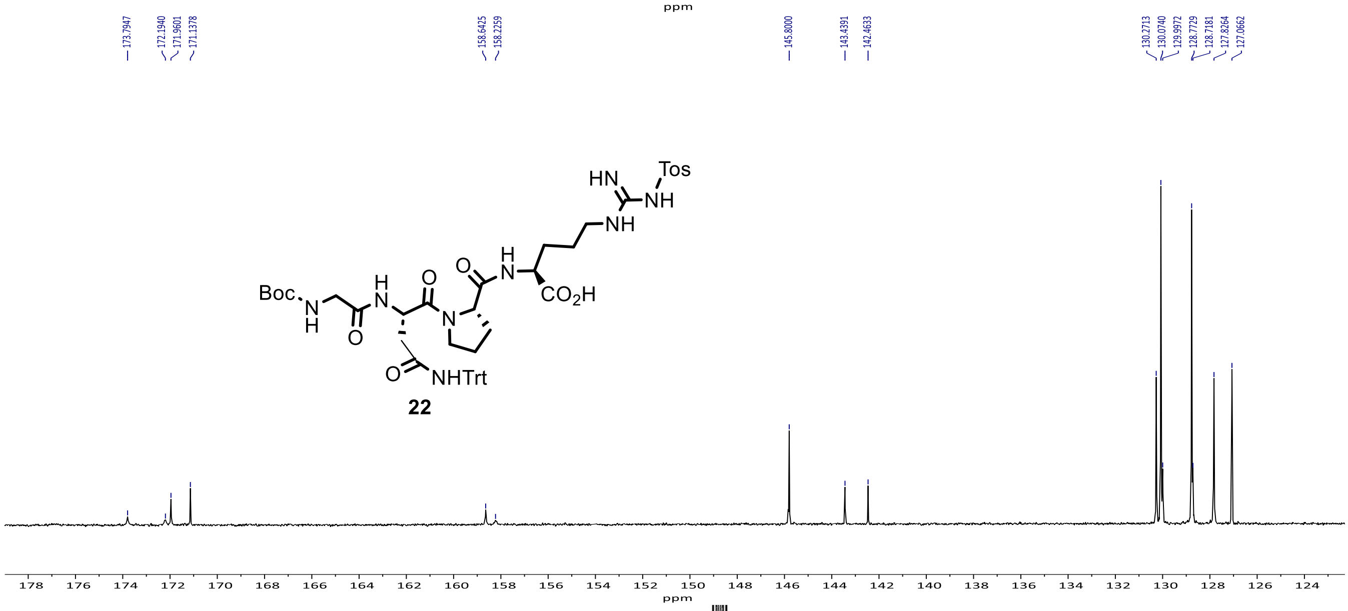
173.7947
172.1940
171.9601
171.1378
158.6425
158.2259
145.8000
143.4391
142.4633
130.2713
130.0740
129.9972
128.7729
128.7181
127.8264
127.0662
ppm
HN
Tos
NH
NH
H
N
O
CO2H
Boc
N
H
O
H
N
O
N
O
NHTrt

22

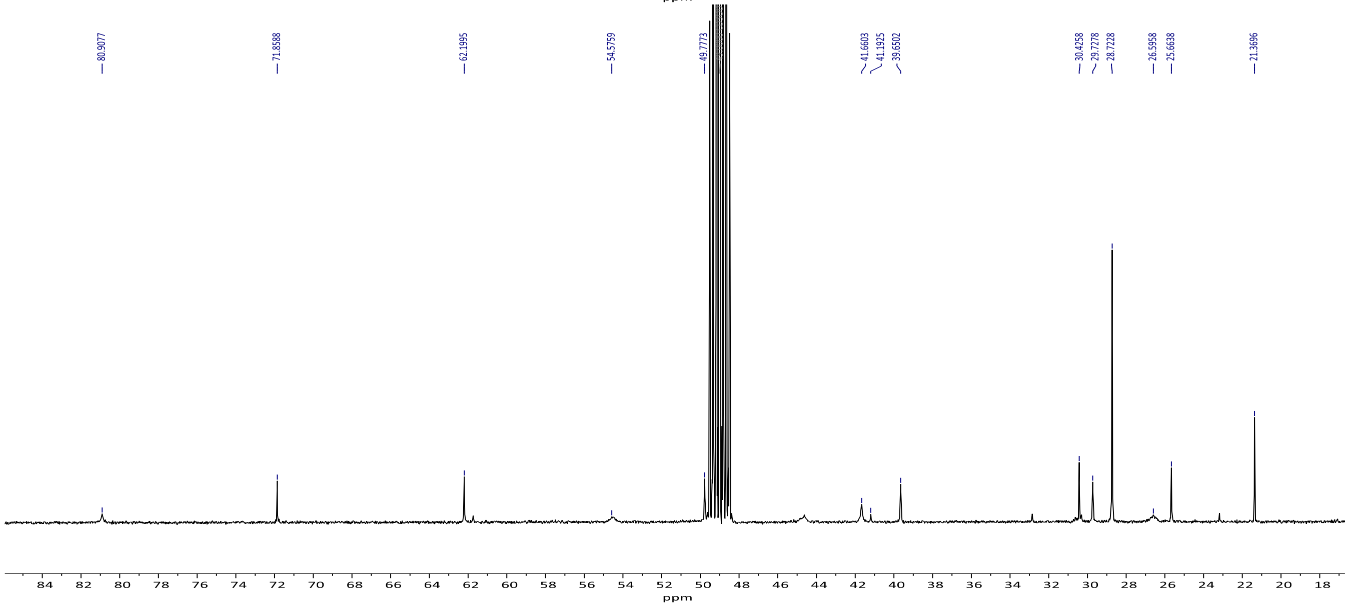
80.9077
71.8588
62.1995
54.5759
49.7773
41.6603
41.1925
39.6502
30.4258
29.7278
28.7228
26.5958
25.6638
21.3696
ppm

Compound **23**, ^{1}H and ^{13}C NMR at 50°C in CD_3OD

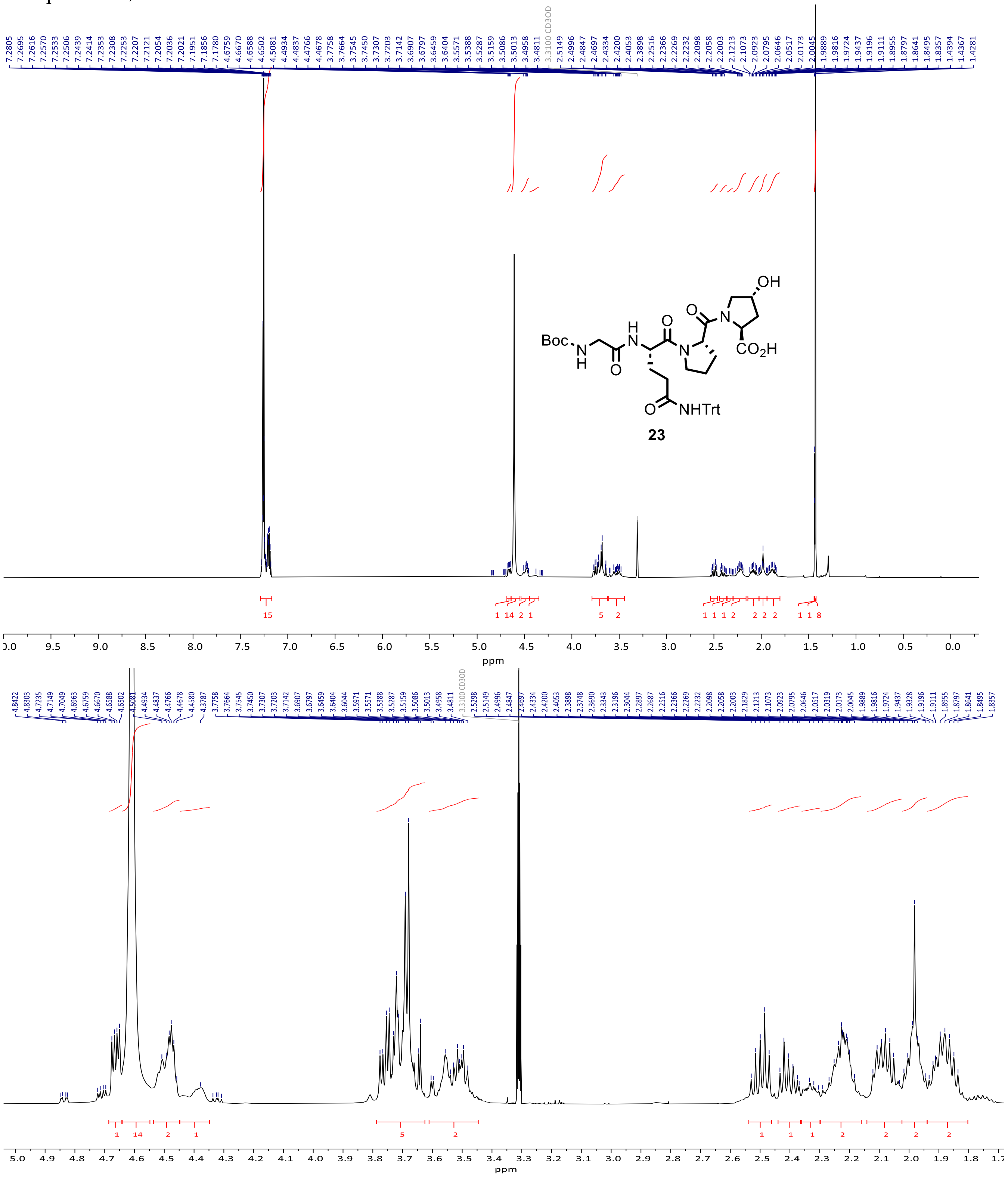

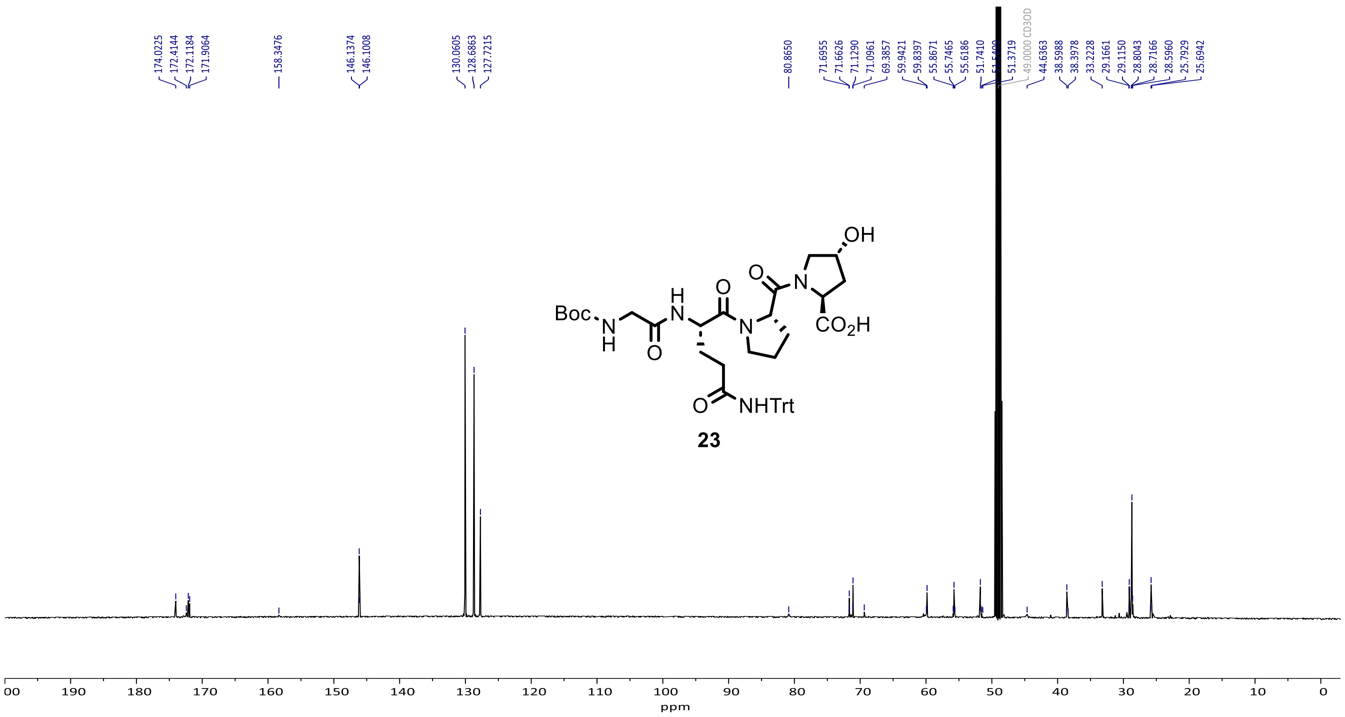
174.0225
172.4144
172.1184
171.9064
158.3476
146.1374
146.1008
130.0605
128.6863
127.7215
80.8650
71.6955
71.6626
71.1290
71.0961
69.3857
59.9421
59.8397
55.8671
55.7465
55.6186
51.7410
51.3719
49.0000 CD3OD
44.6363
38.5988
38.3978
33.2228
29.1661
29.1150
28.8043
28.7166
28.5960
25.7929
25.6942
Boc
N
H
O
OH
CO2H
NHTrt
23
ppm

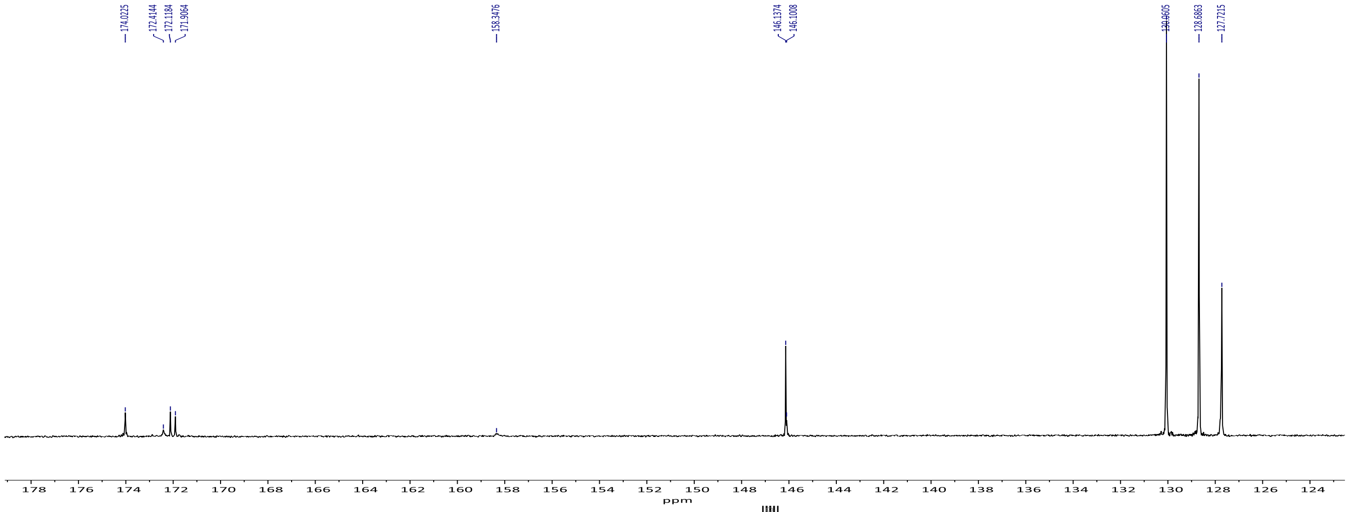
174.0225
172.4144
172.1184
171.9064
158.3476
146.1374
146.1008
128.6863
127.7215
ppm

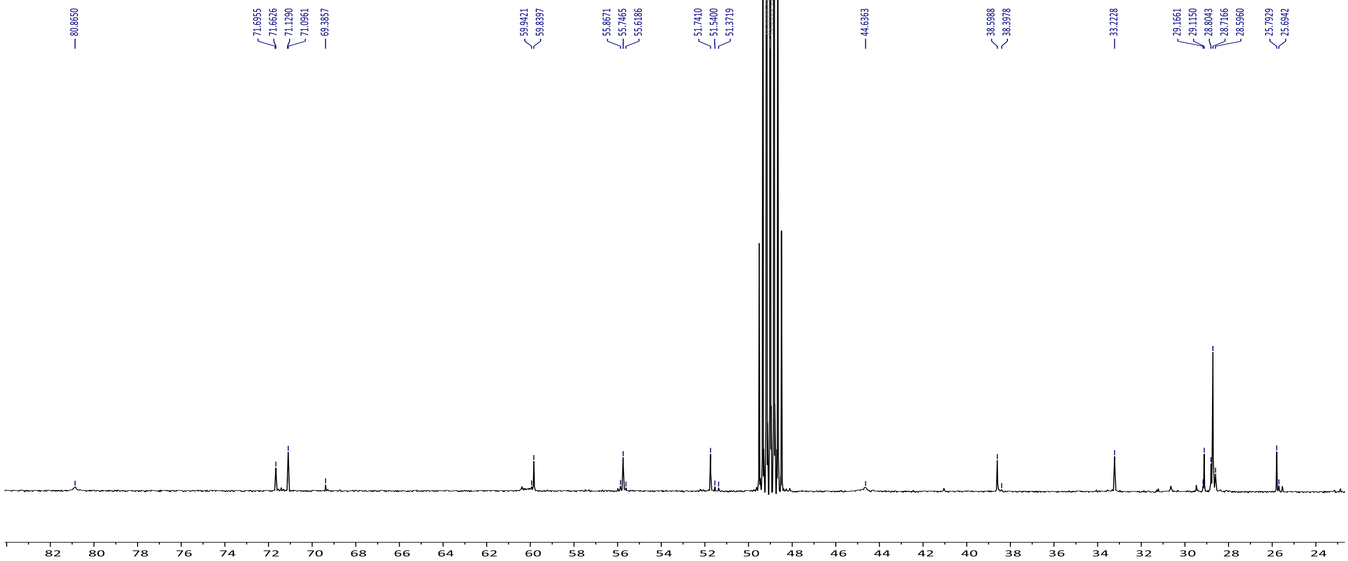
80.8650
71.6955
71.6626
71.1290
71.0961
69.3857
59.9421
59.8397
55.8671
55.7465
55.6186
51.7410
51.5400
51.3719
44.6363
38.5988
38.3978
33.2228
29.1661
29.1150
28.8043
28.7166
28.5960
25.7929
25.6942
ppm

Compound **24**, ^{1}H and ^{13}C NMR at 50°C in CD_3OD

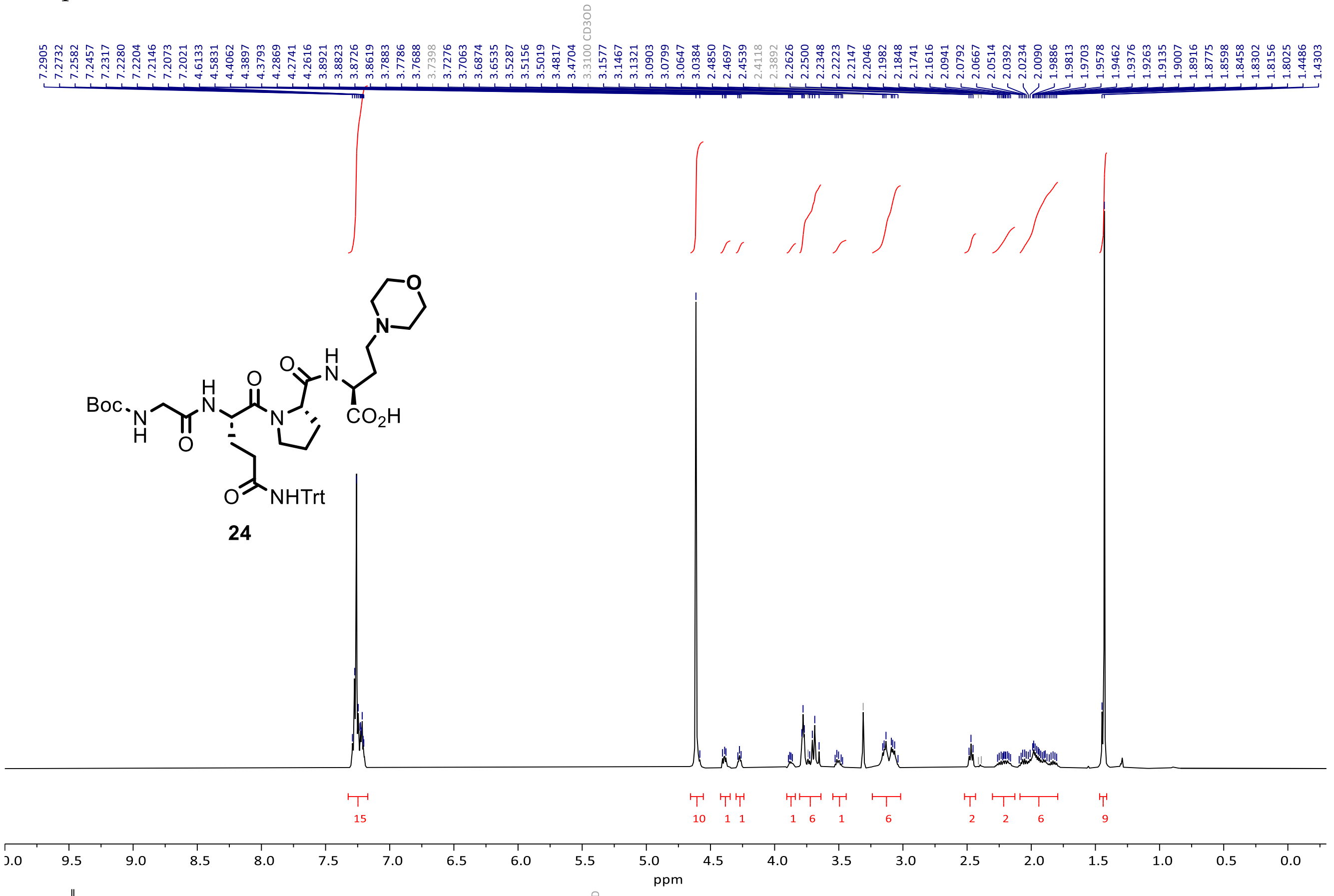

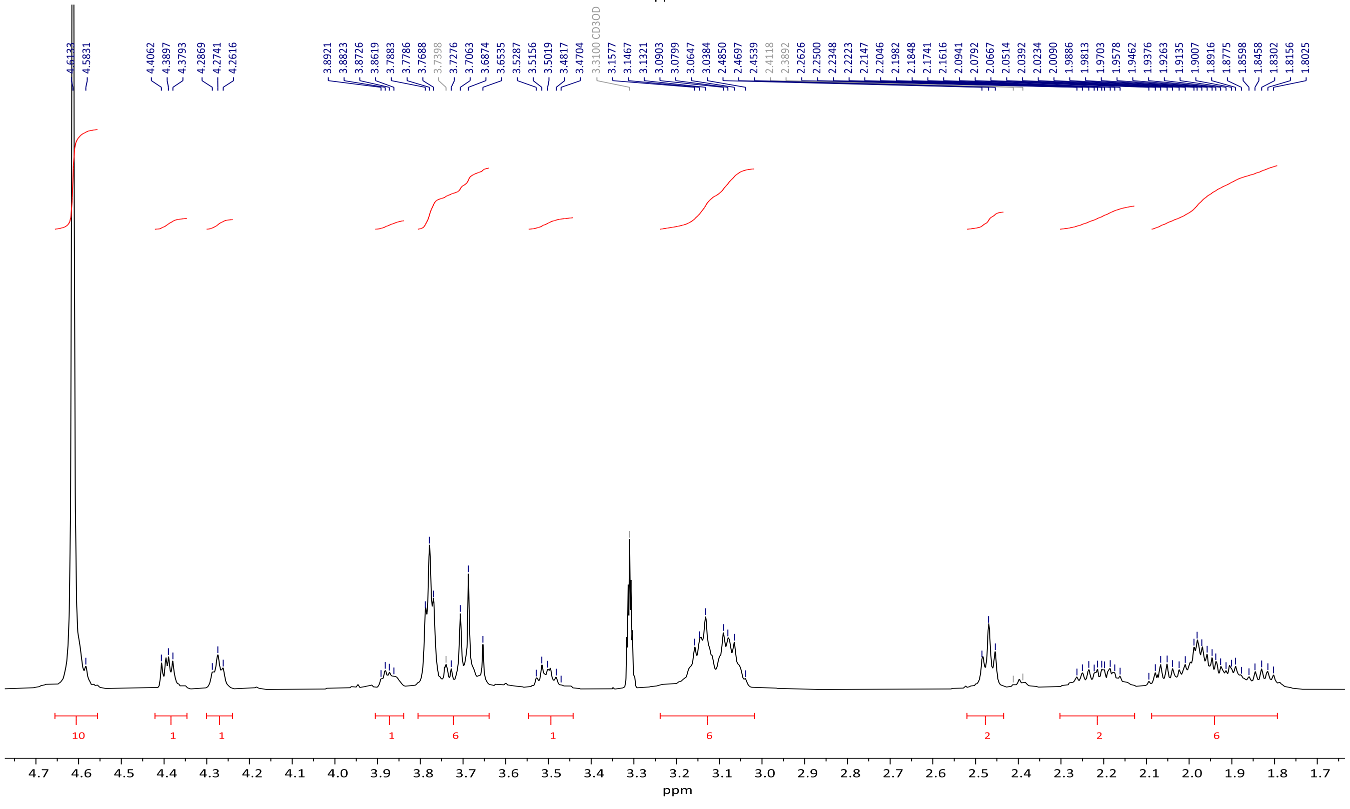

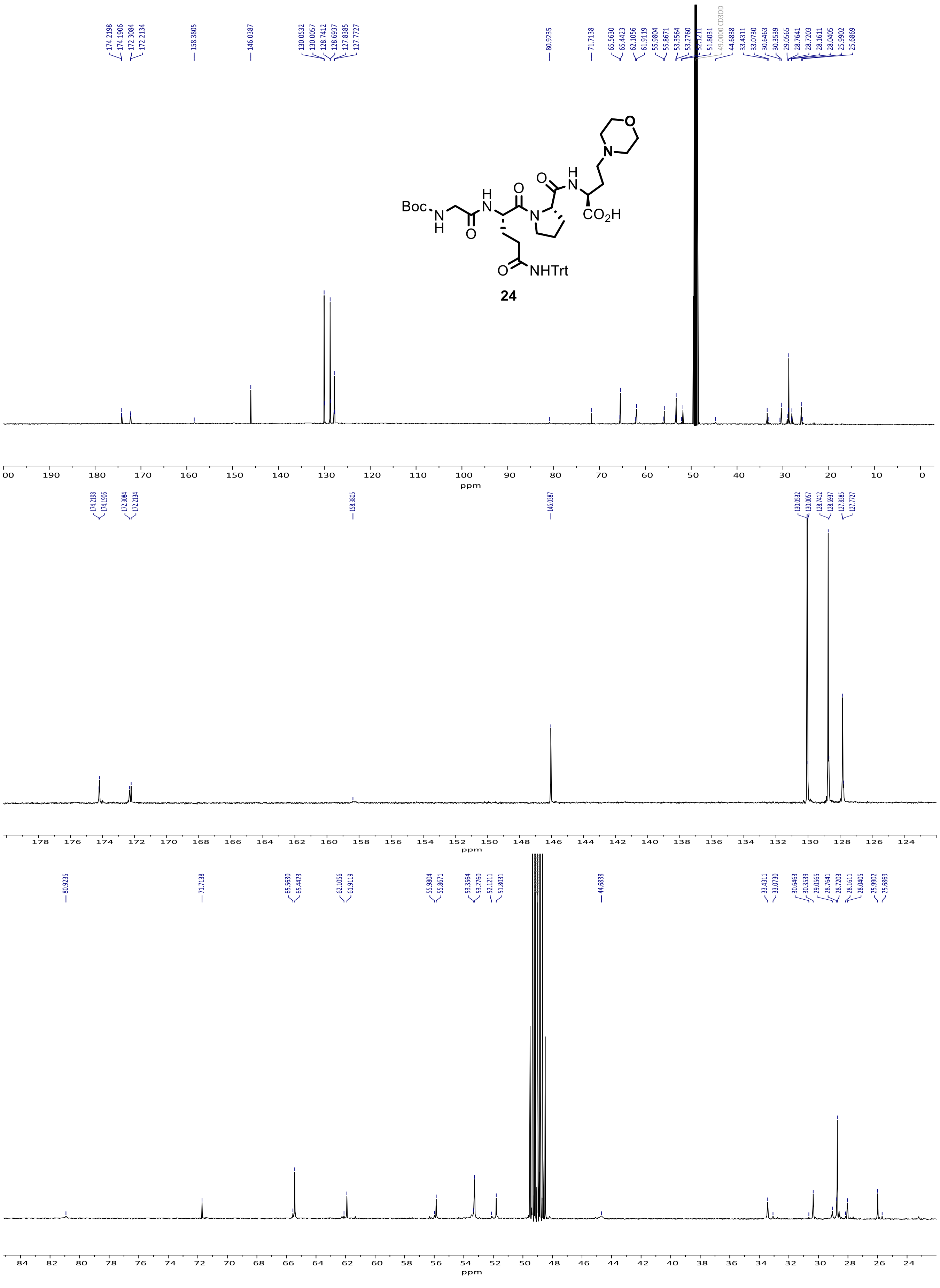
Boc
NHTrt
CO2H
24
CD3OD
ppm

Compound **24**, HSQC and COSY at 50°C in CD3OD

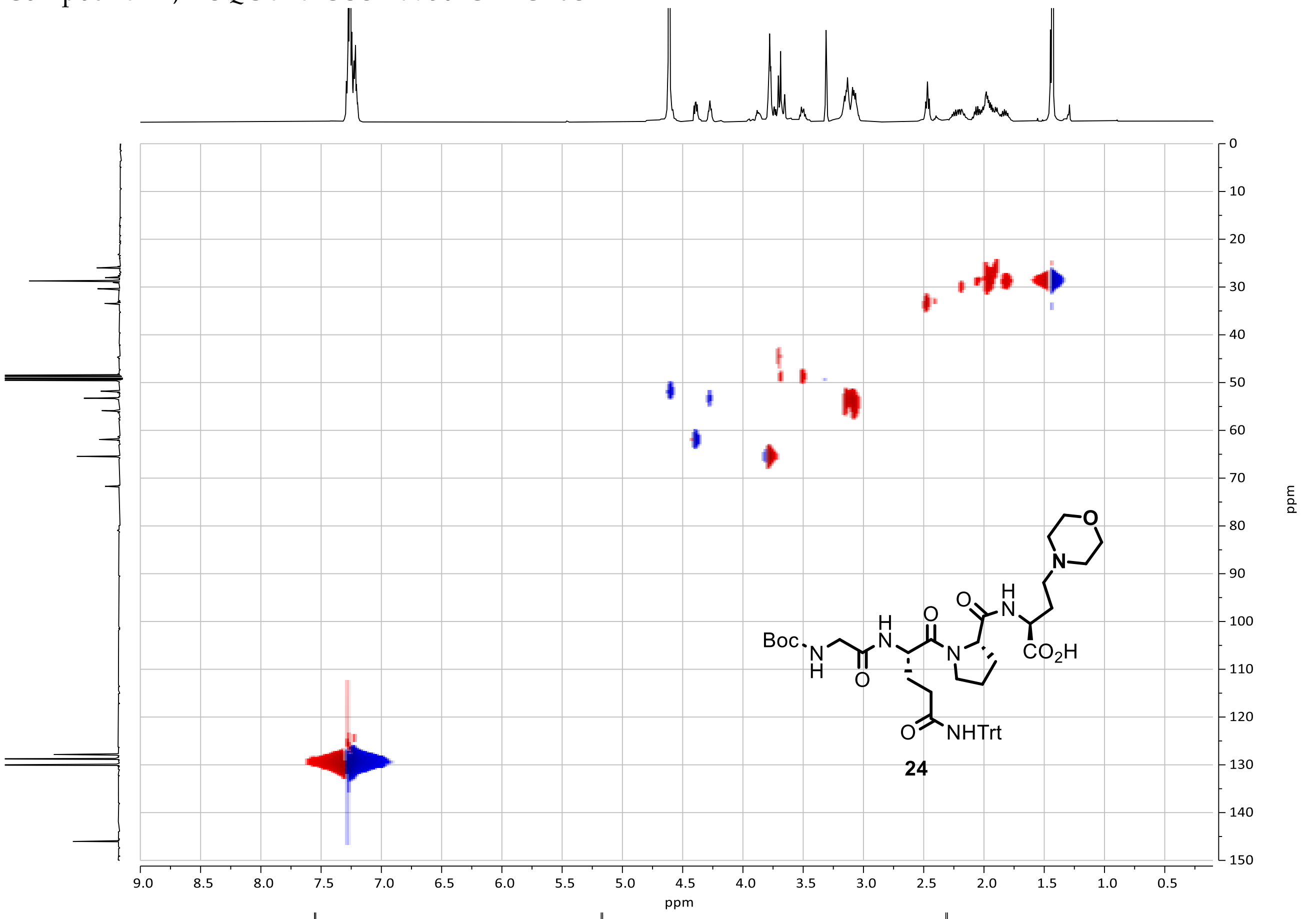

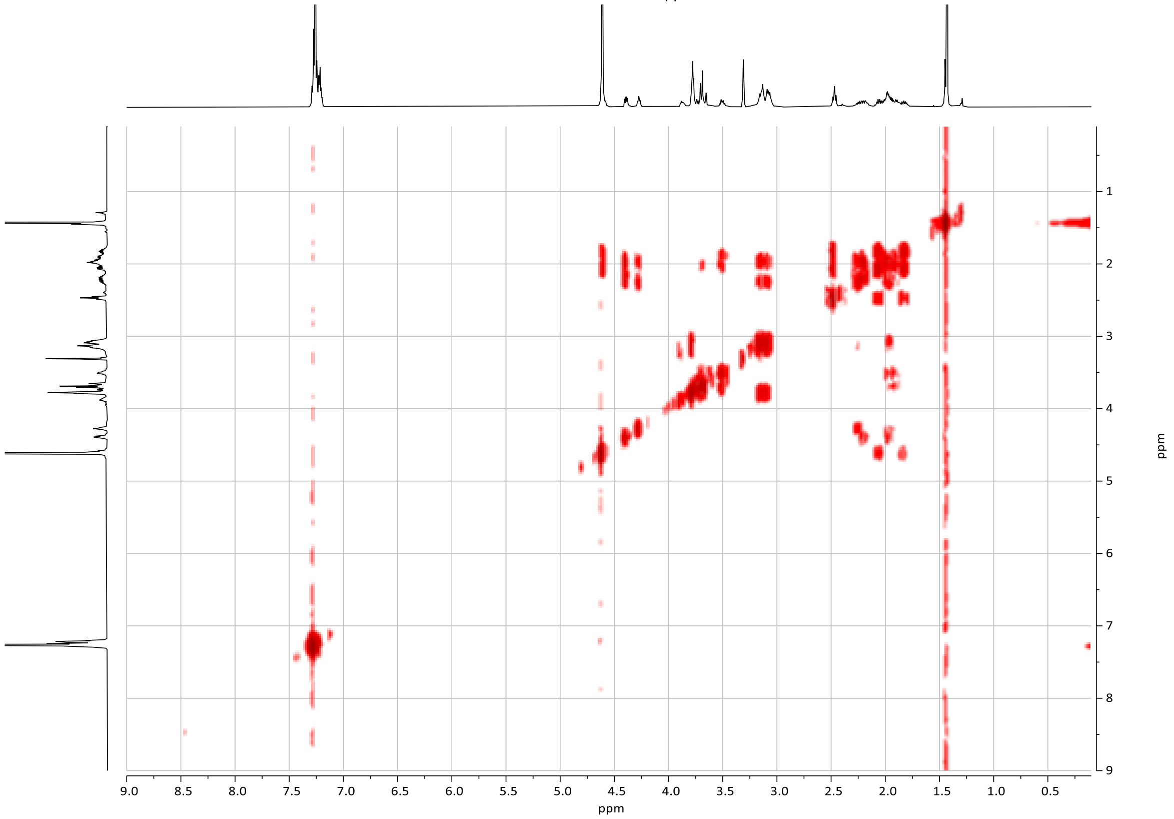

Compound **25**, ^{1}H and ^{13}C NMR at 50°C in CD_3OD

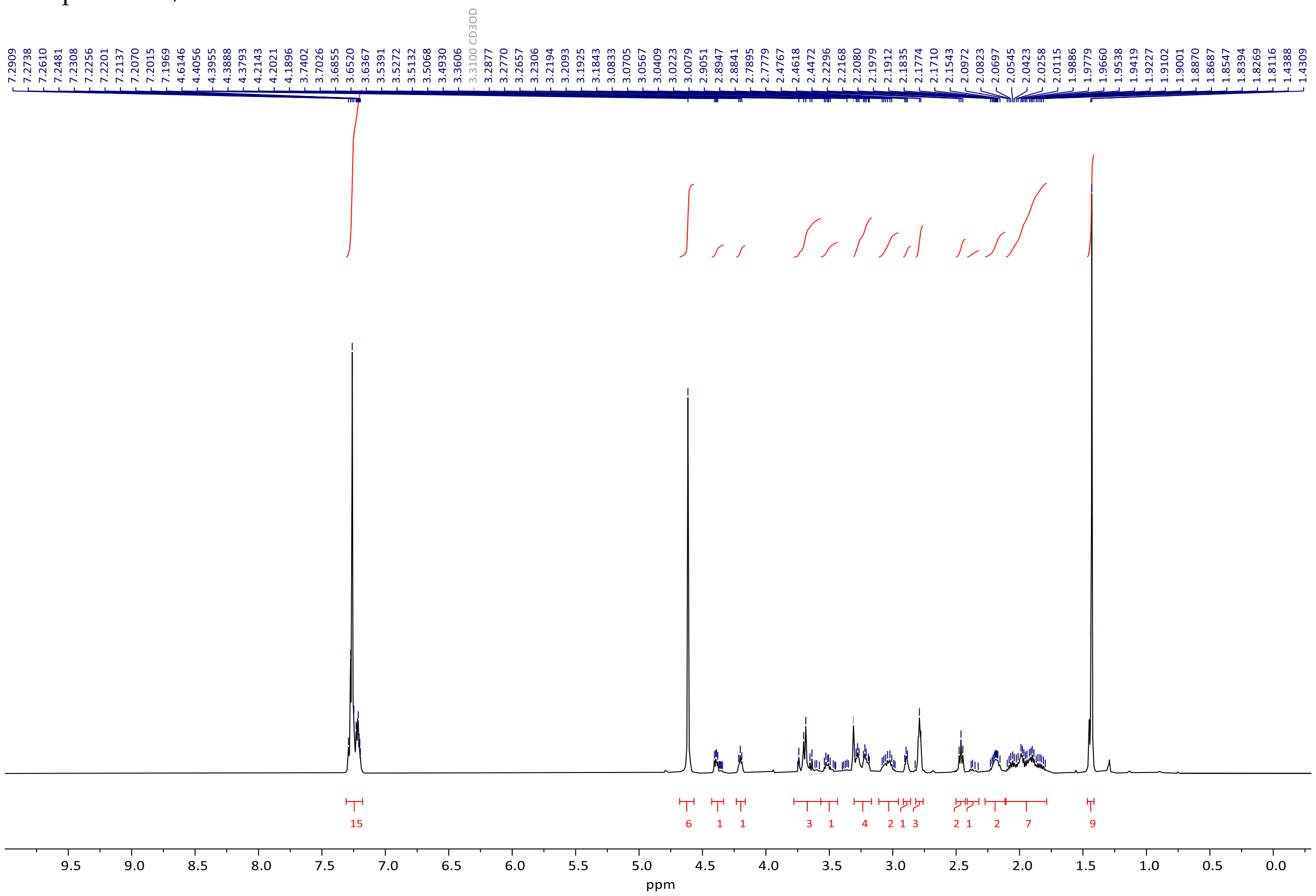

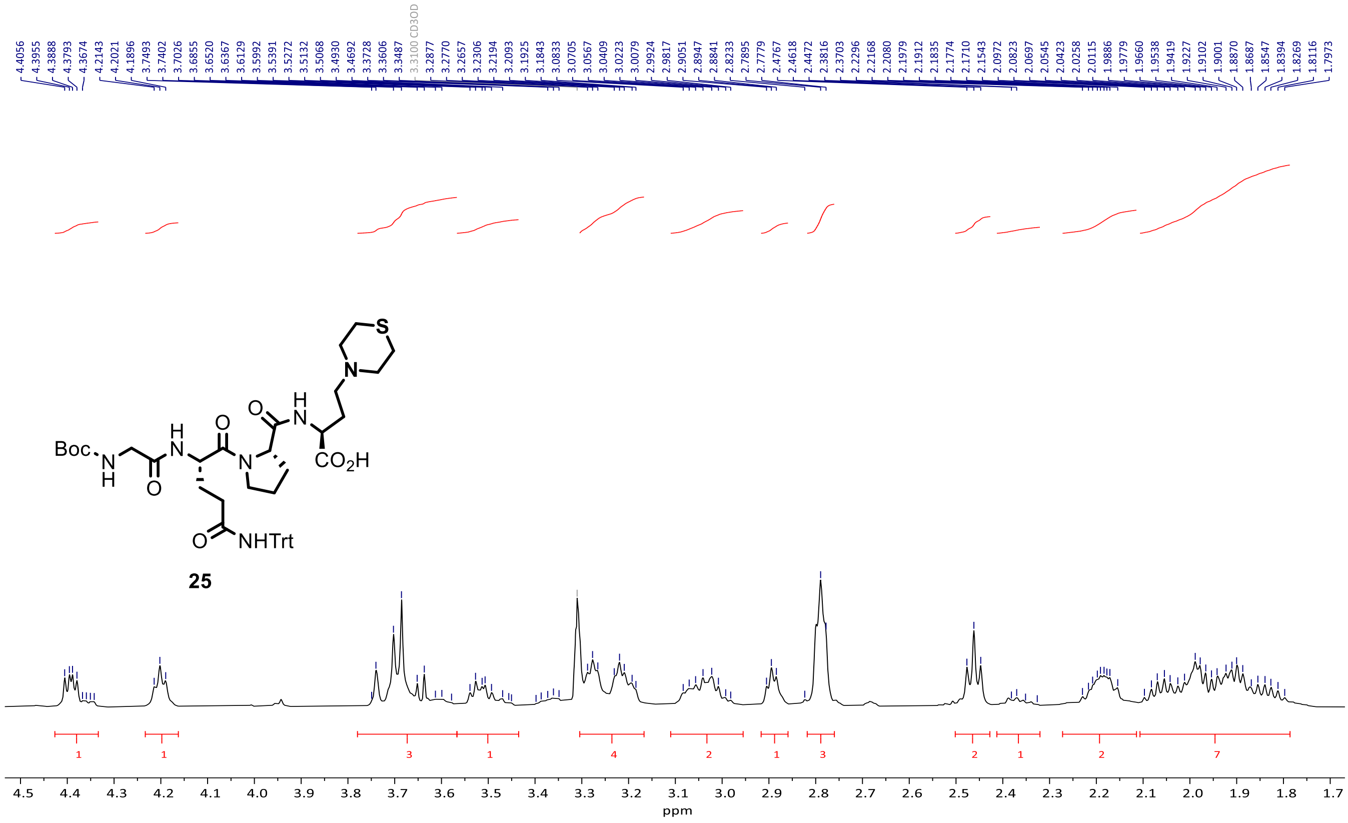

25

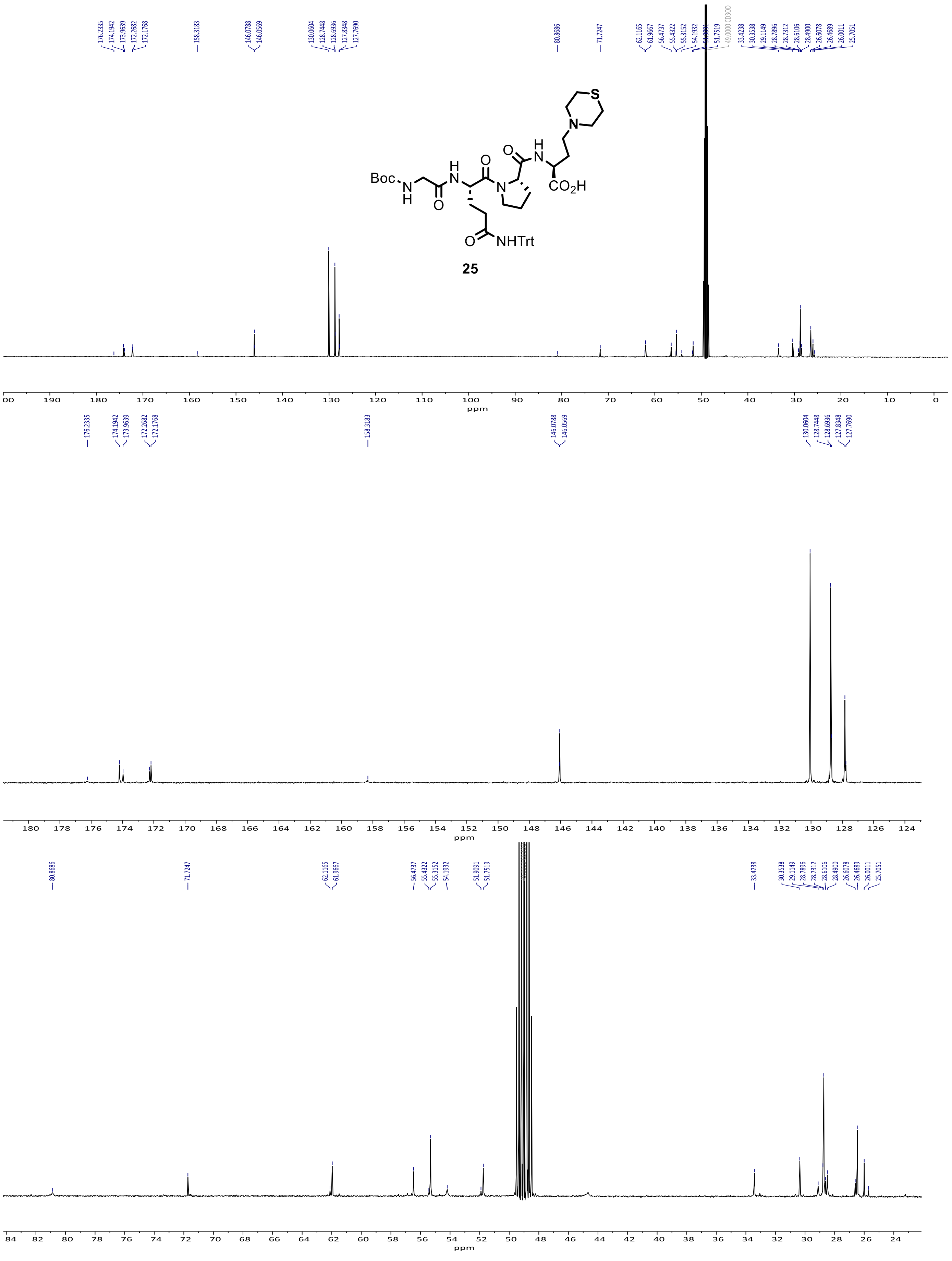

Boc
NHTrt
CO2H
S
N
25
ppm

Compound **26**, ^{1}H and ^{13}C NMR at 80°C in D_2O

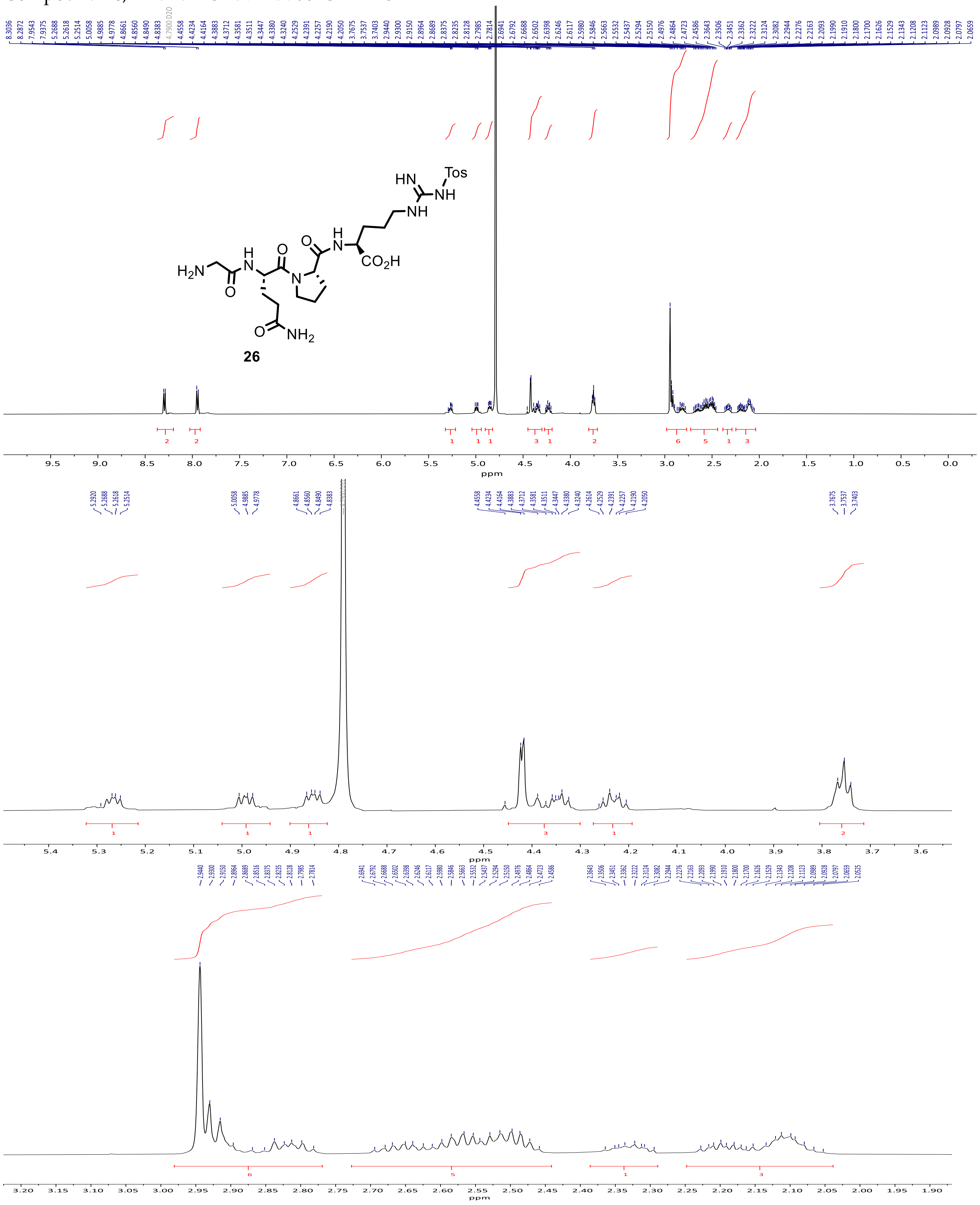

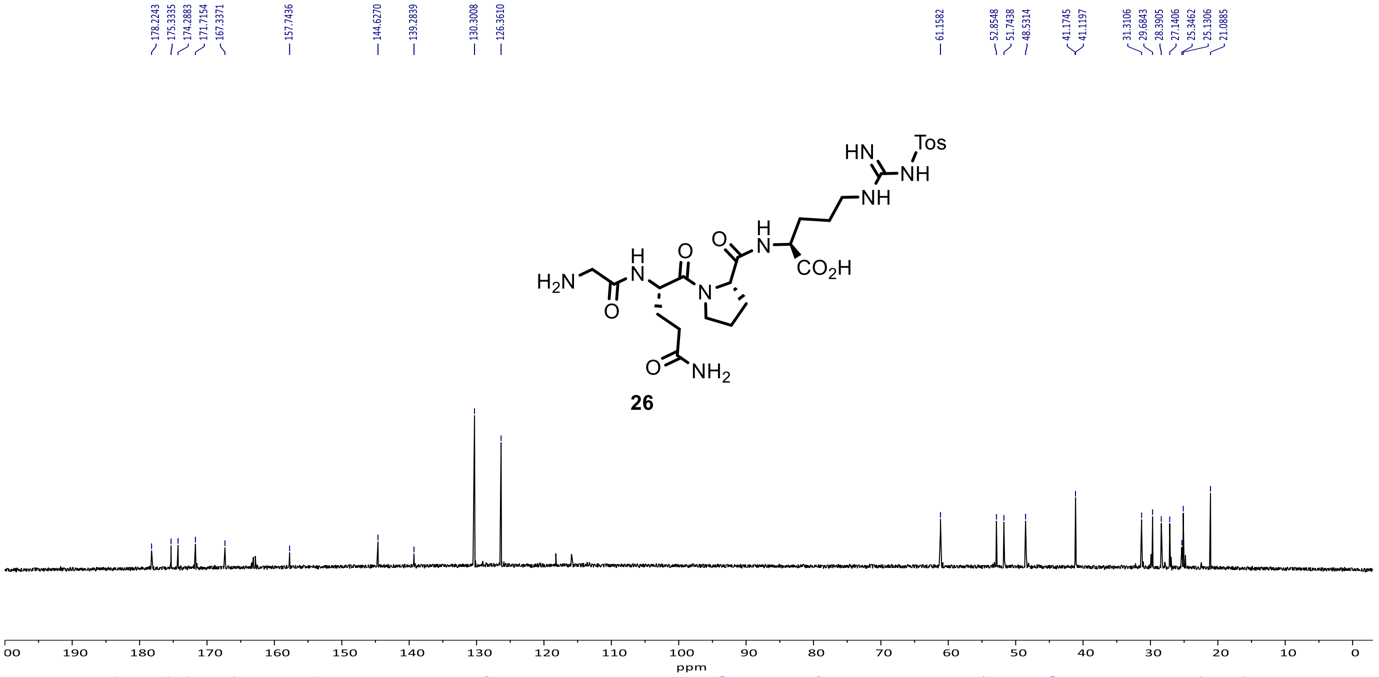
178.2243
175.3335
174.2883
171.7154
167.3371
157.7436
144.6270
139.2839
130.3008
126.3610
61.1582
52.8548
51.7438
48.5314
41.1745
41.1197
31.3106
29.6843
28.3905
27.1406
25.3462
25.1306
21.0885
ppm
HN
Tos
NH
NH
H
N
O
CO2H
H2N
H
N
O
N
O
O
NH2
26

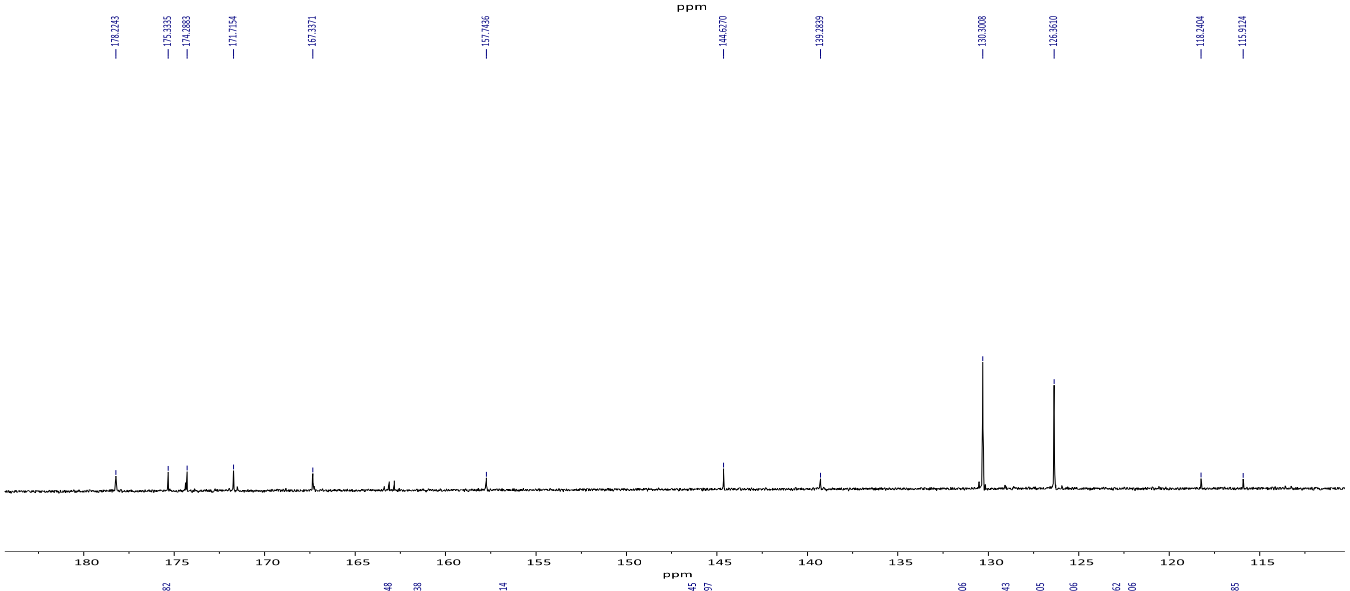
178.2243
175.3335
174.2883
171.7154
167.3371
157.7436
144.6270
139.2839
130.3008
126.3610
118.2404
115.9124
ppm

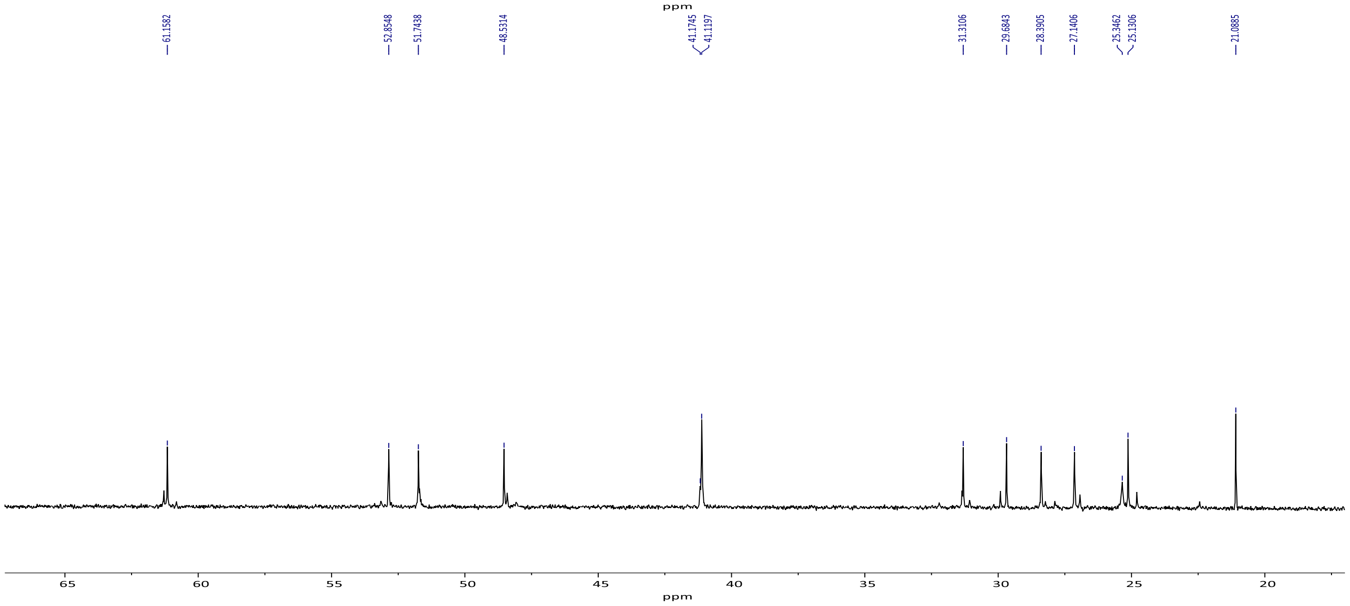
61.1582
52.8548
51.7438
48.5314
41.1745
41.1197
31.3106
29.6843
28.3905
27.1406
25.3462
25.1306
21.0885
ppm

Compound **27**, ^{1}H and ^{13}C NMR at 80°C in D_2O

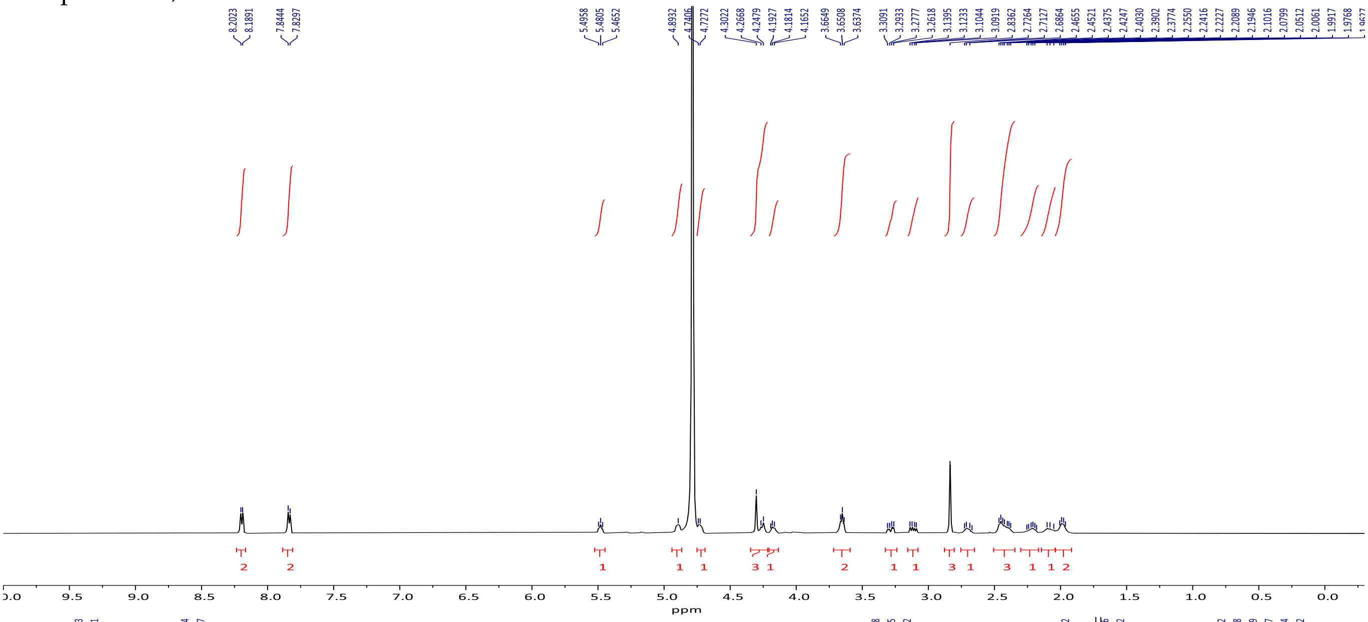

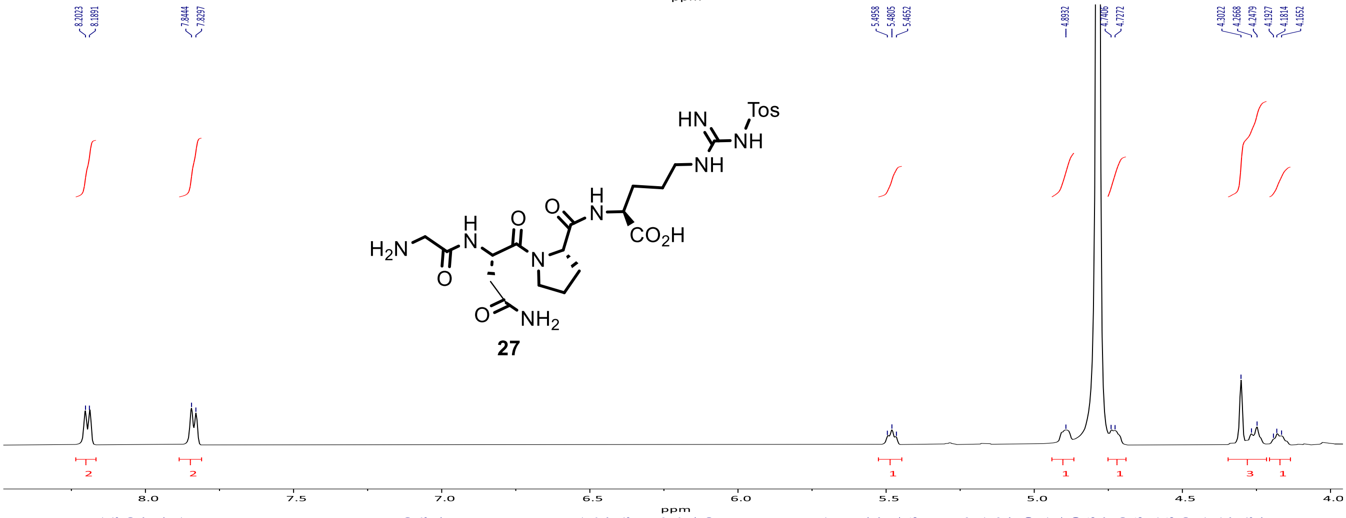

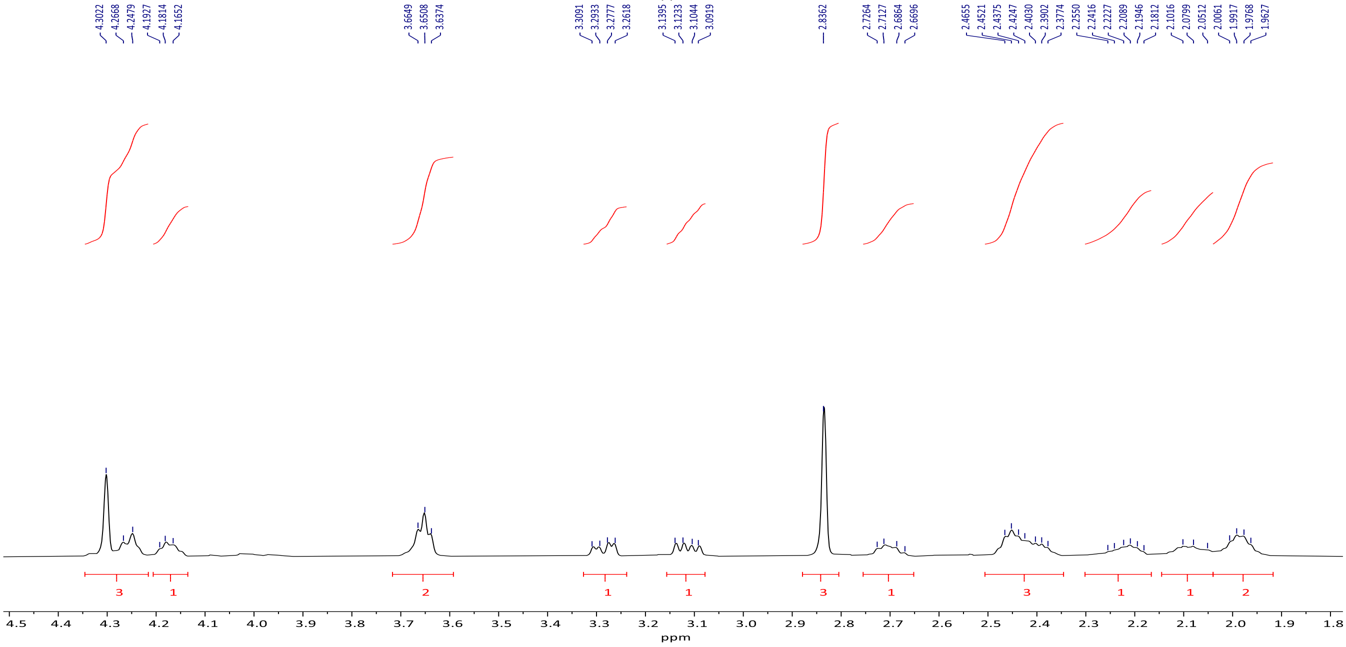

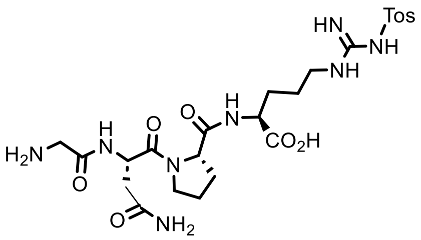
HN
Tos
NH
NH
H
N
O
O
H
N
H2N
N
O
CO2H
O
NH2

27

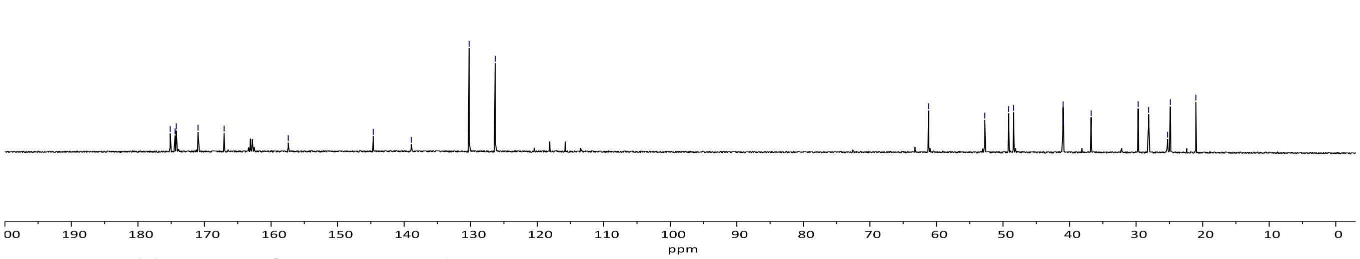
175.1471
174.4198
174.2334
170.9735
167.0520
157.4147
144.6307
138.9111
130.2313
126.3135
61.1838
52.7379
49.1673
48.4290
41.0028
40.9662
36.7488
29.6879
28.1274
25.2731
24.8638
21.0154
ppm

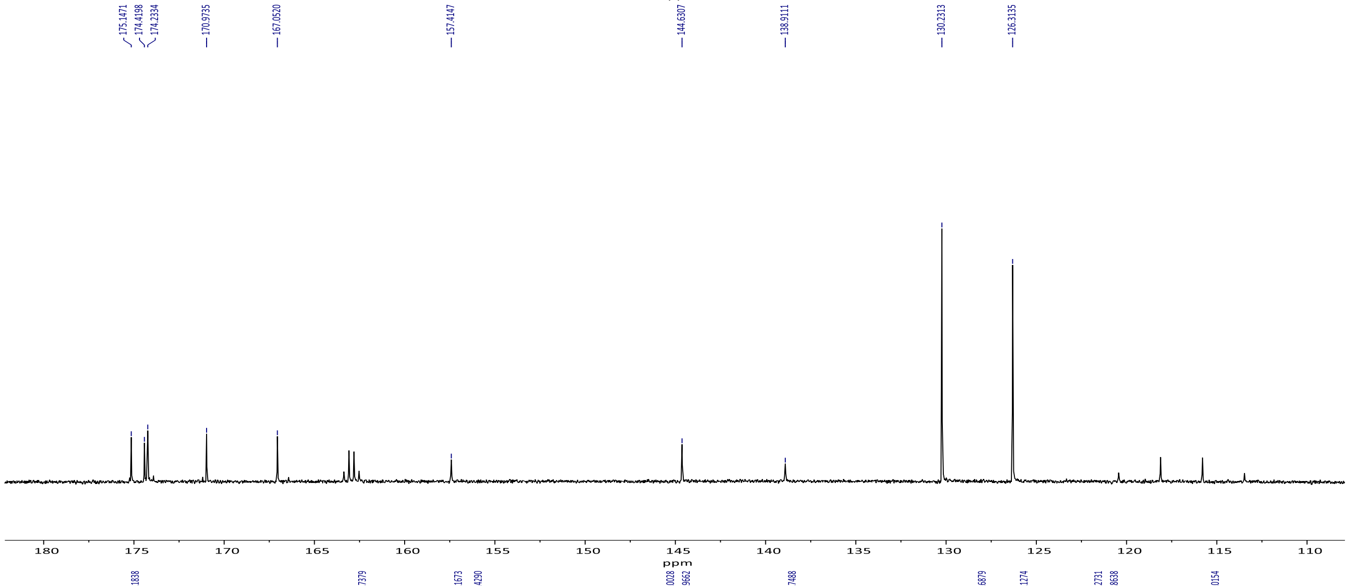
175.1471
174.4198
174.2334
170.9735
167.0520
157.4147
144.6307
138.9111
130.2313
126.3135
ppm

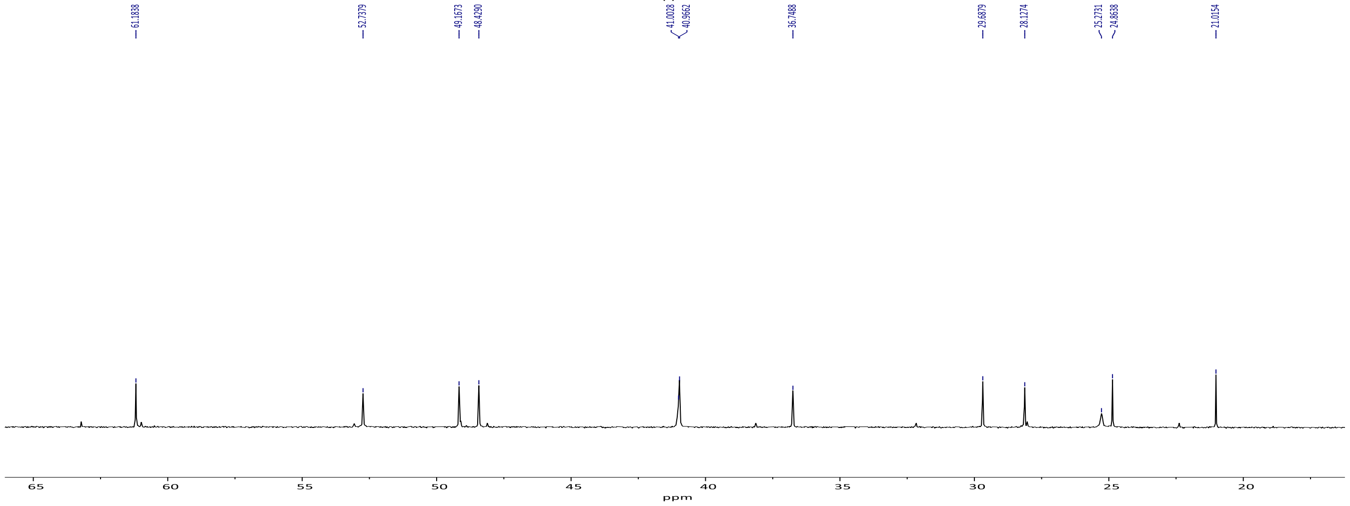
61.1838
52.7379
49.1673
48.4290
41.0028
40.9662
36.7488
29.6879
28.1274
25.2731
24.8638
21.0154
ppm

Compound **28**, ^{1}H and ^{13}C NMR at 80°C in D_2O

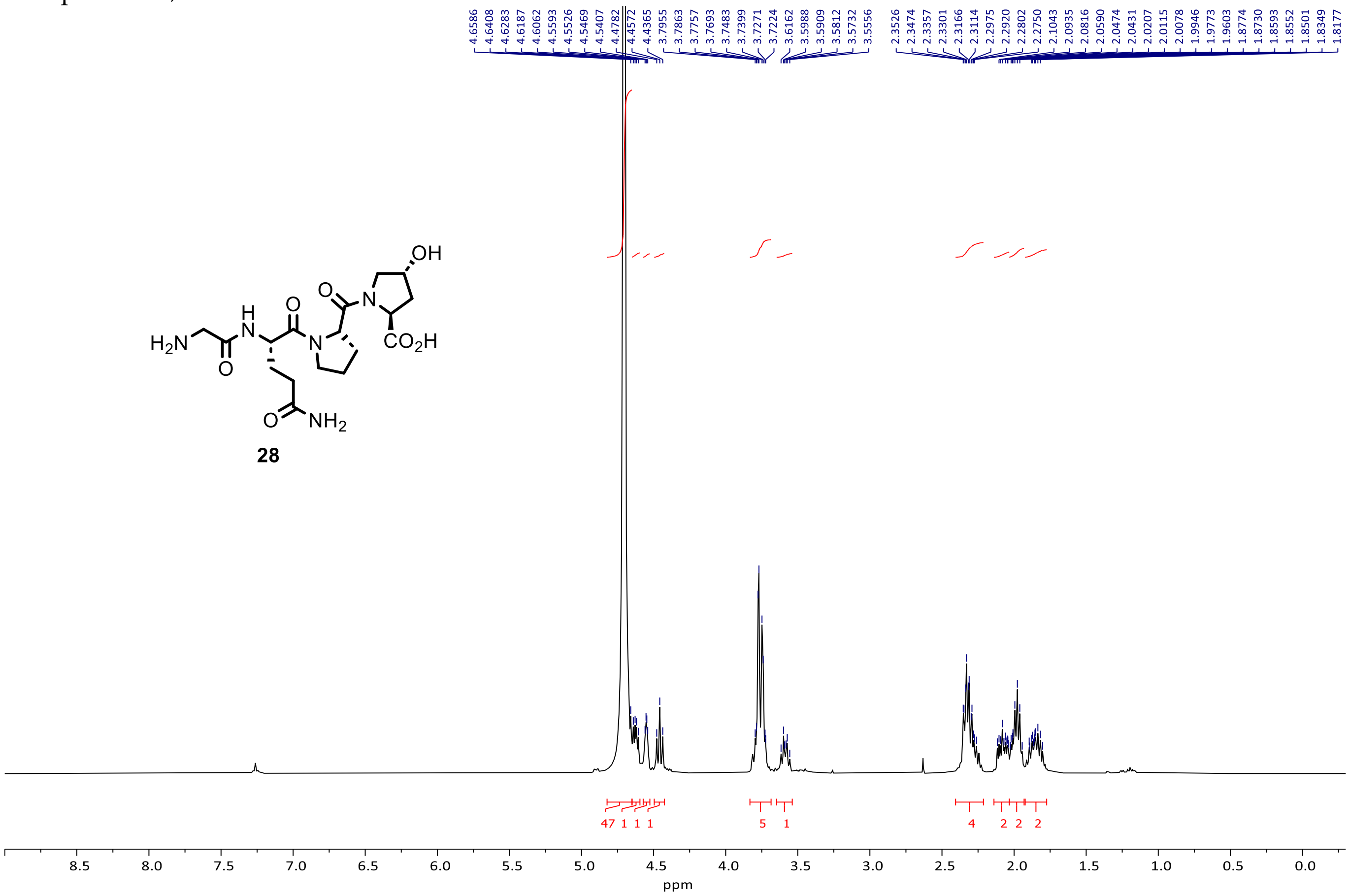

28

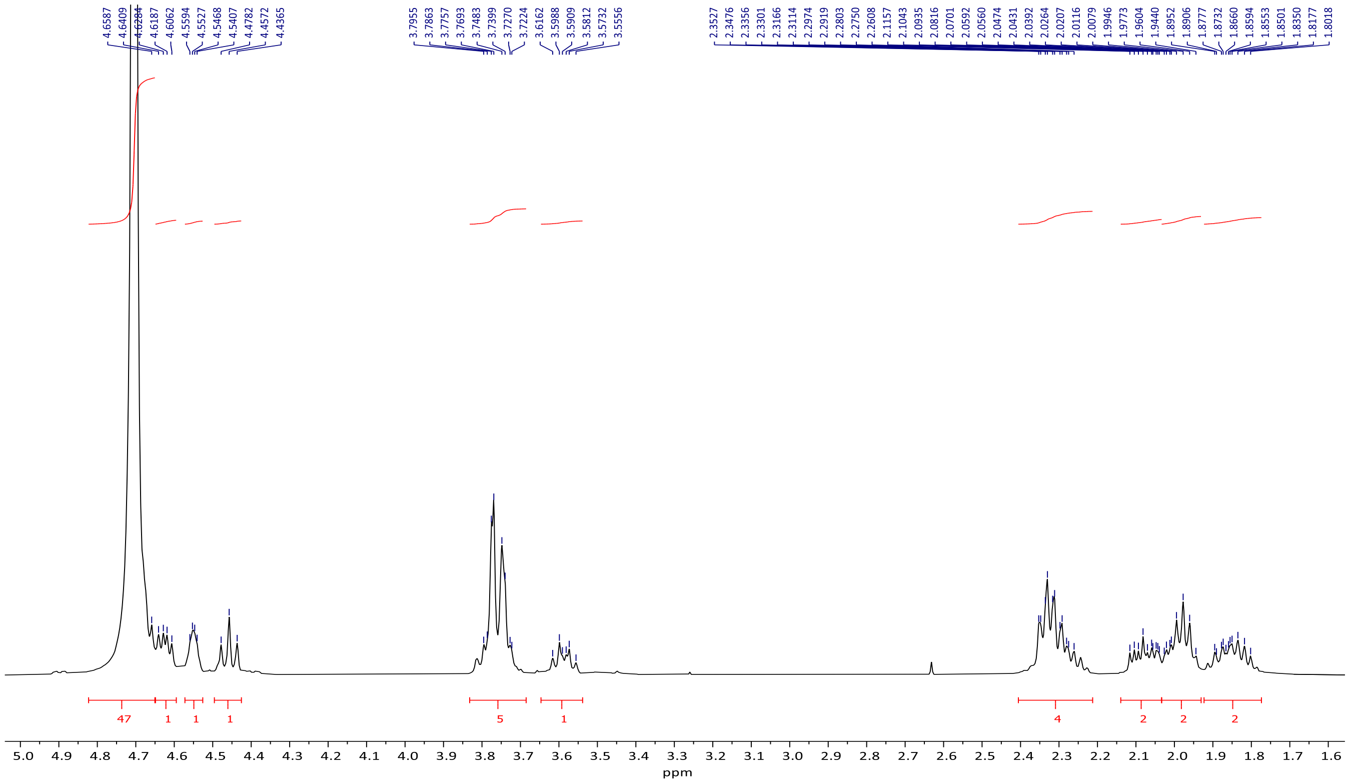

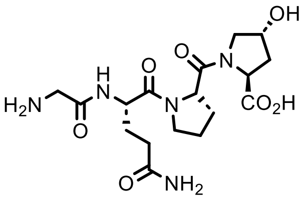
OH
O
N
H
O
H2N
N
CO2H
O
O
NH2

28

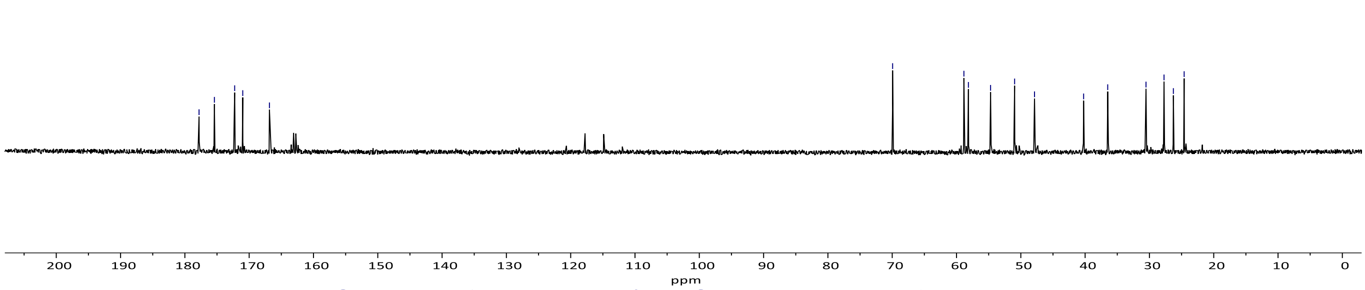
177.80
175.41
172.26
171.00
166.85
69.94
58.85
58.15
54.70
50.97
47.87
40.23
36.49
30.53
27.73
26.28
24.60
200 190 180 170 160 150 140 130 120 110 100 90 80 70 60 50 40 30 20 10 0
ppm

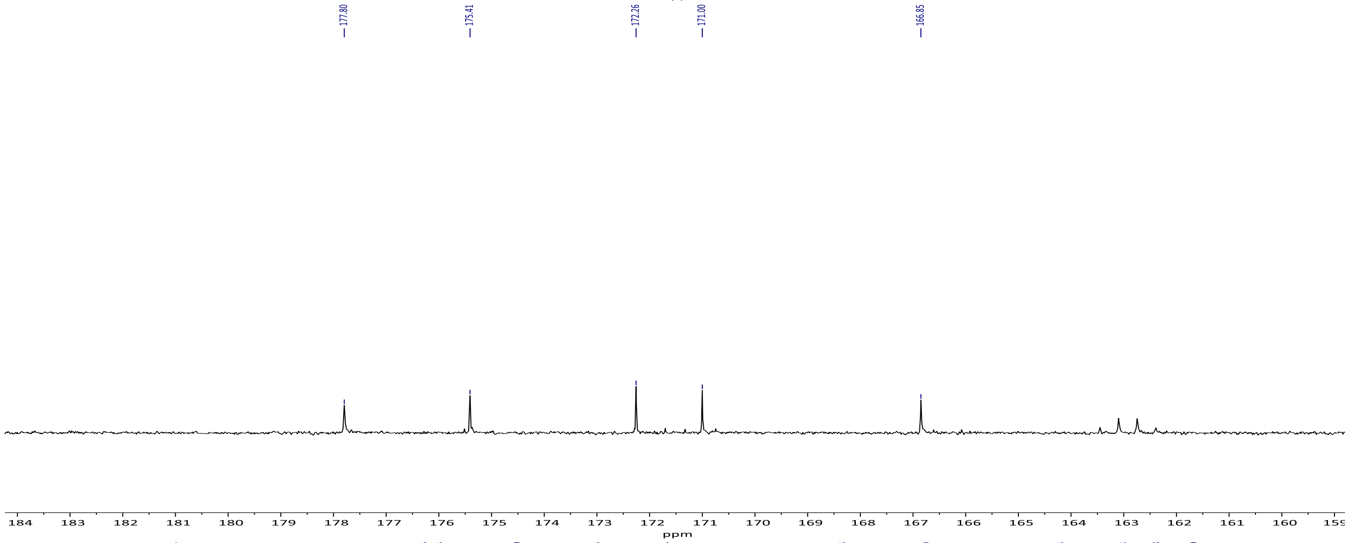
177.80
175.41
172.26
171.00
166.85
184 183 182 181 180 179 178 177 176 175 174 173 172 171 170 169 168 167 166 165 164 163 162 161 160
ppm

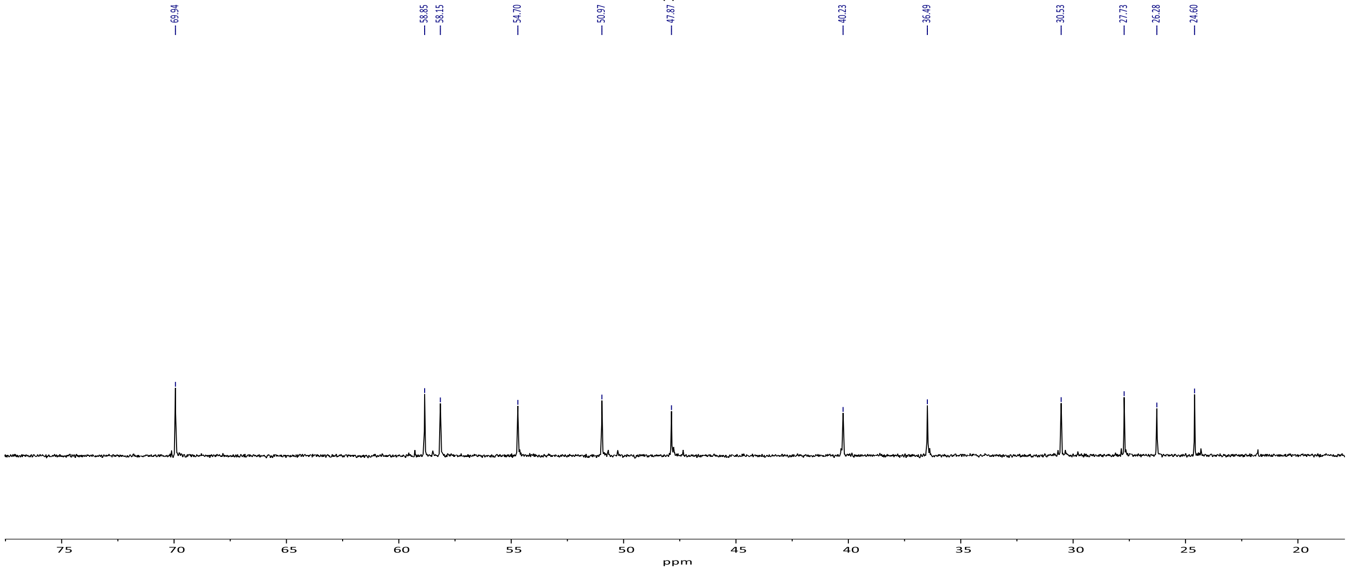
69.94
58.85
58.15
54.70
50.97
47.87
40.23
36.49
30.53
27.73
26.28
24.60
75 70 65 60 55 50 45 40 35 30 25 20
ppm

Compound **29**, ^{1}H and ^{13}C NMR at 80°C in D_2O

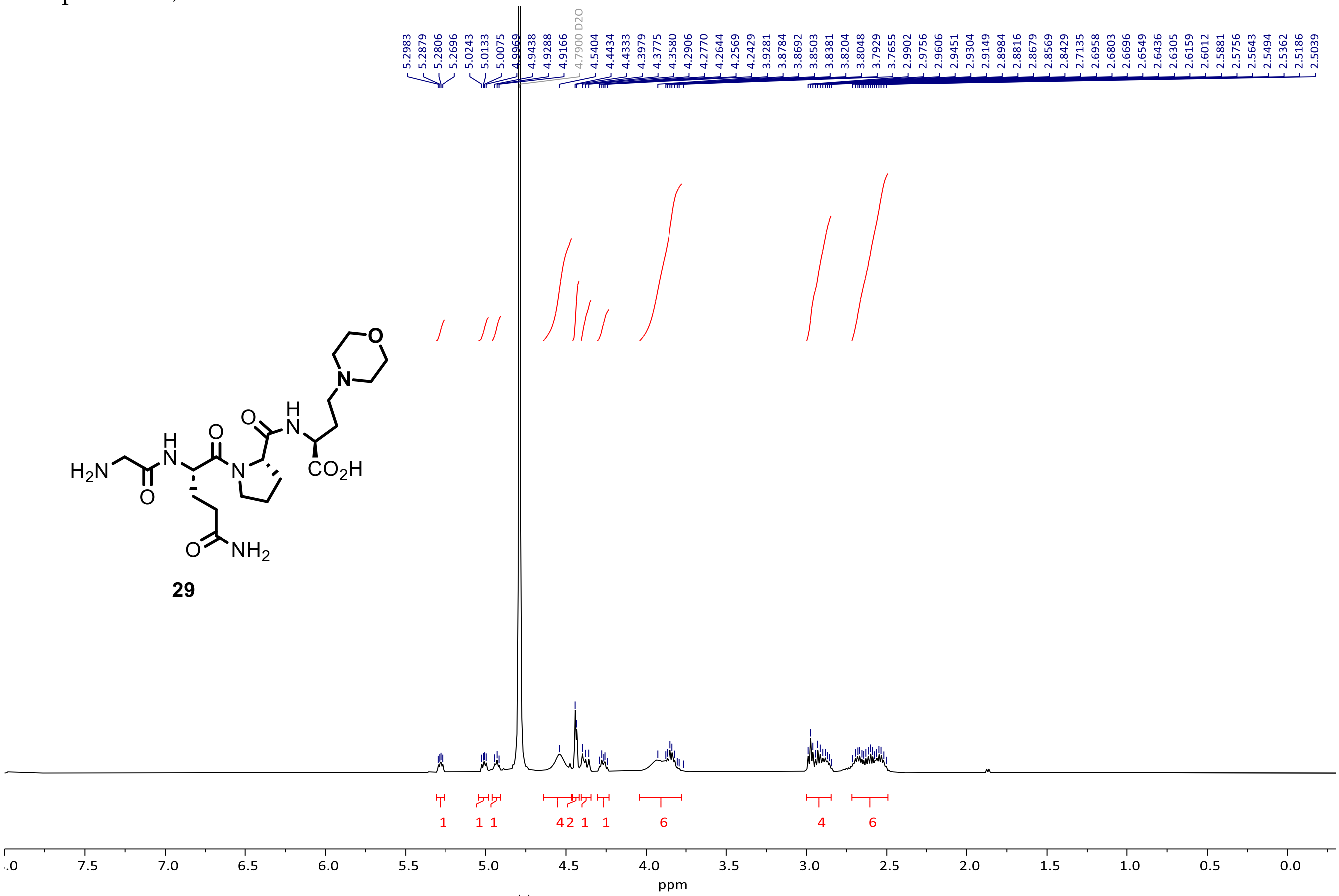

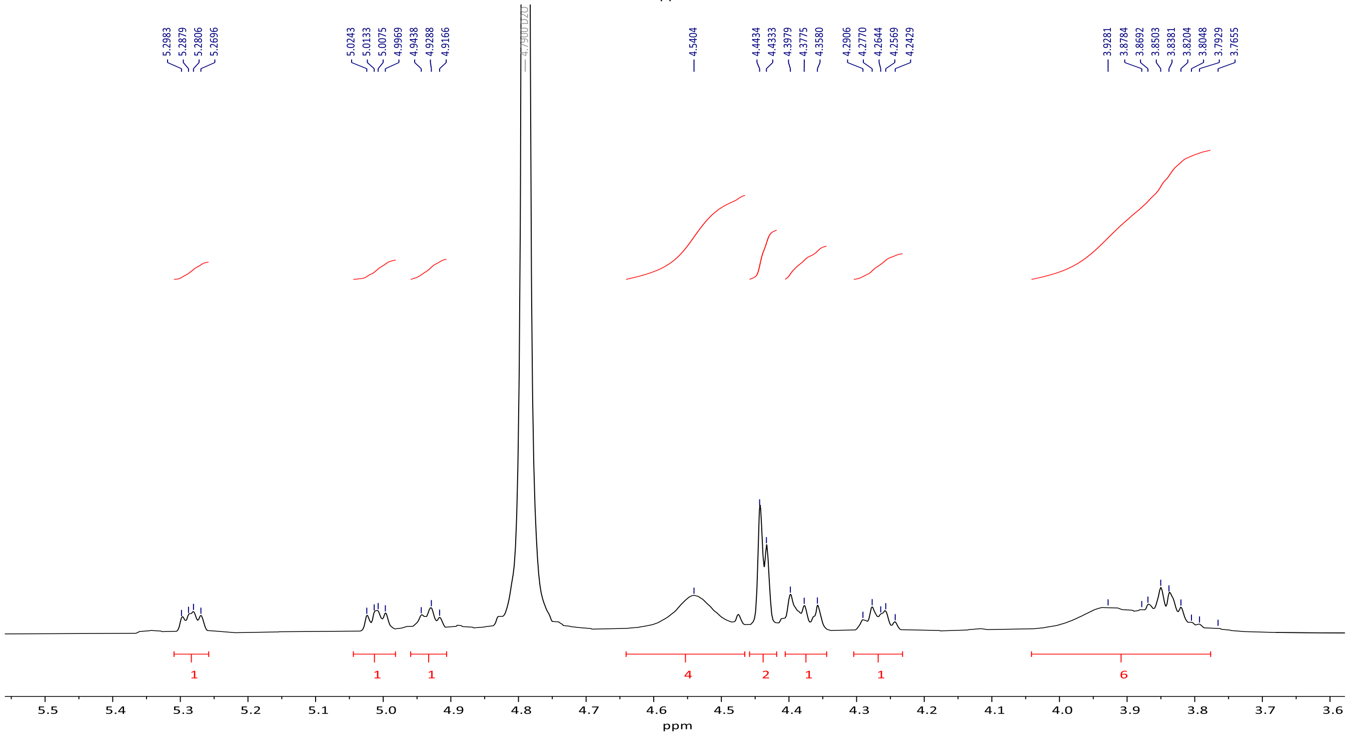

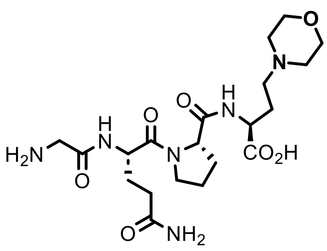

H_2N
O
H
N
O
N
O
H
N
N
O
CO_2H
O
NH_2

29

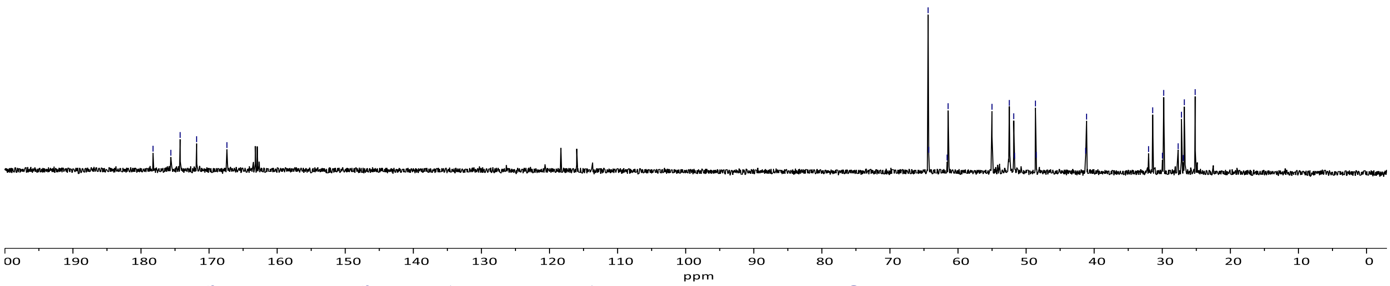

178.2316
175.6186
174.2481
171.8323
167.3810
64.3926
64.3012
61.6041
61.4543
55.0111
52.4674
51.8206
51.6890
48.6301
48.5131
41.2549
41.1197
32.0123
31.4093
29.9803
29.7939
27.6669
27.1845
26.9141
26.7642
25.1671
00 190 180 170 160 150 140 130 120 110 100 90 80 70 60 50 40 30 20 10 0
ppm

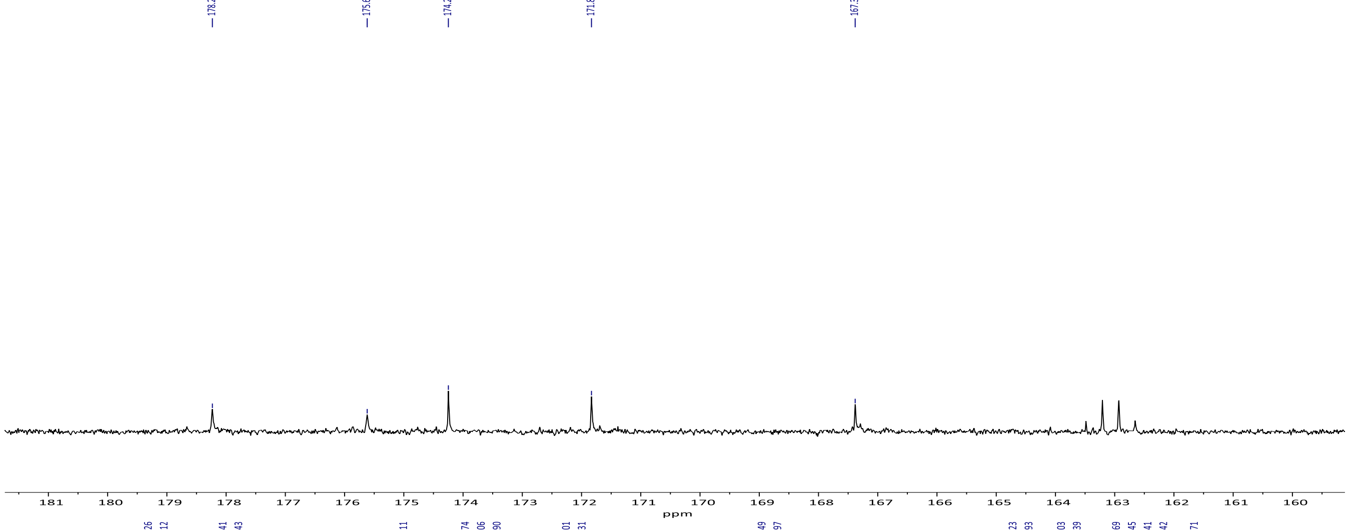

178.2316
175.6186
174.2481
171.8323
167.3810
181 180 179 178 177 176 175 174 173 172 171 170 169 168 167 166 165 164 163 162 161 160
ppm

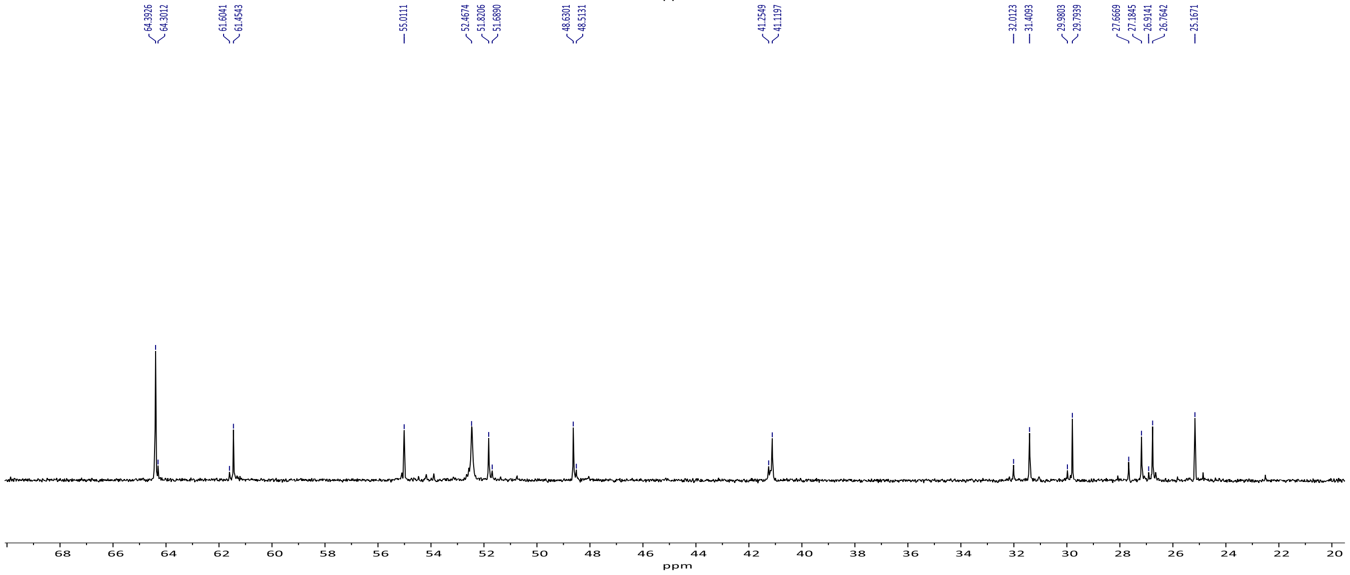

64.3926
64.3012
61.6041
61.4543
55.0111
52.4674
51.8206
51.6890
48.6301
48.5131
41.2549
41.1197
32.0123
31.4093
29.9803
29.7939
27.6669
27.1845
26.9141
26.7642
25.1671
68 66 64 62 60 58 56 54 52 50 48 46 44 42 40 38 36 34 32 30 28 26 24 22 20
ppm

Compound **29**, HSQC at 80°C in D_2O

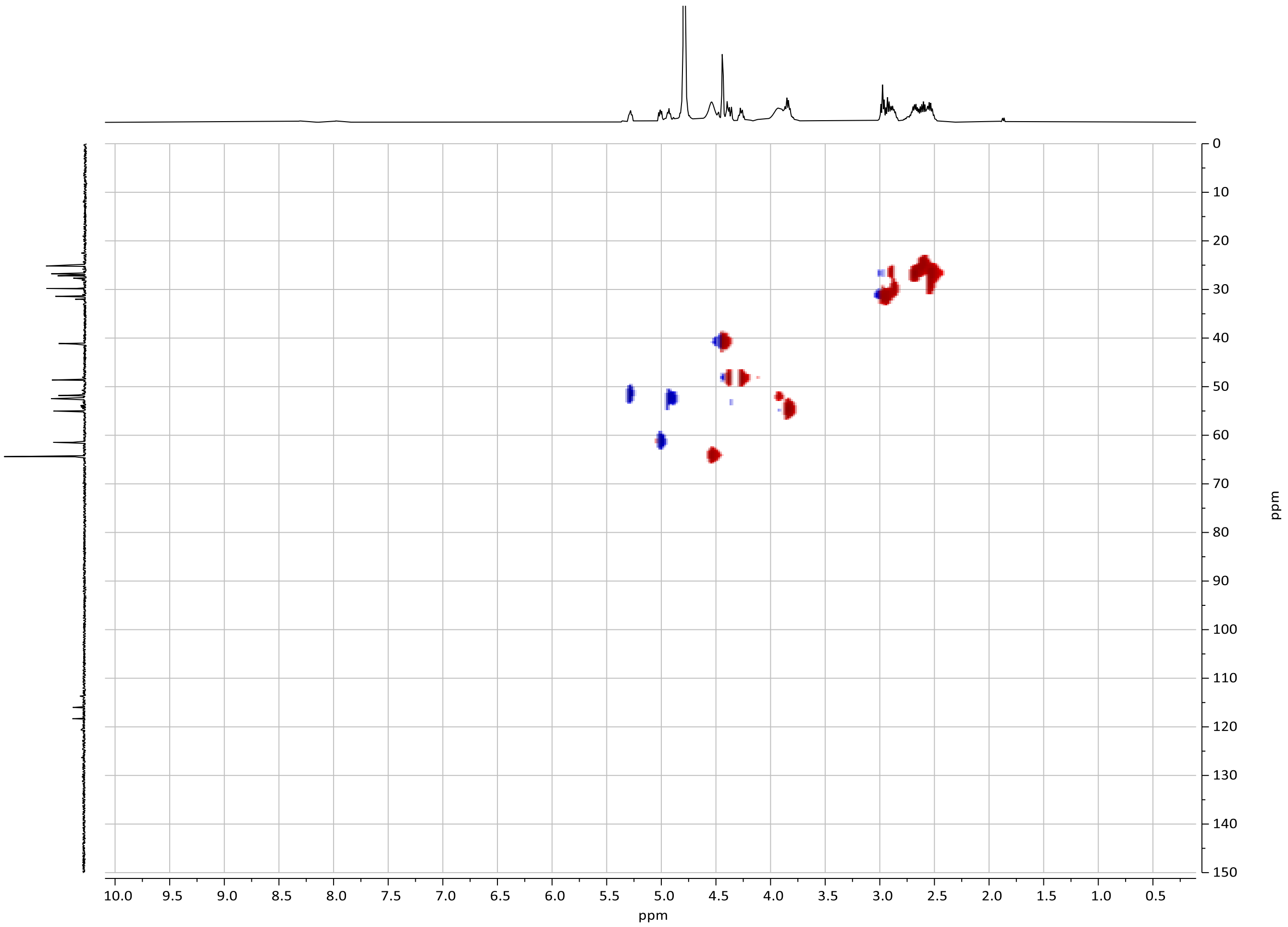

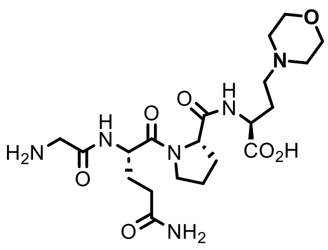

29

Compound **30**, ^{1}H and ^{13}C NMR at 80°C in D_2O

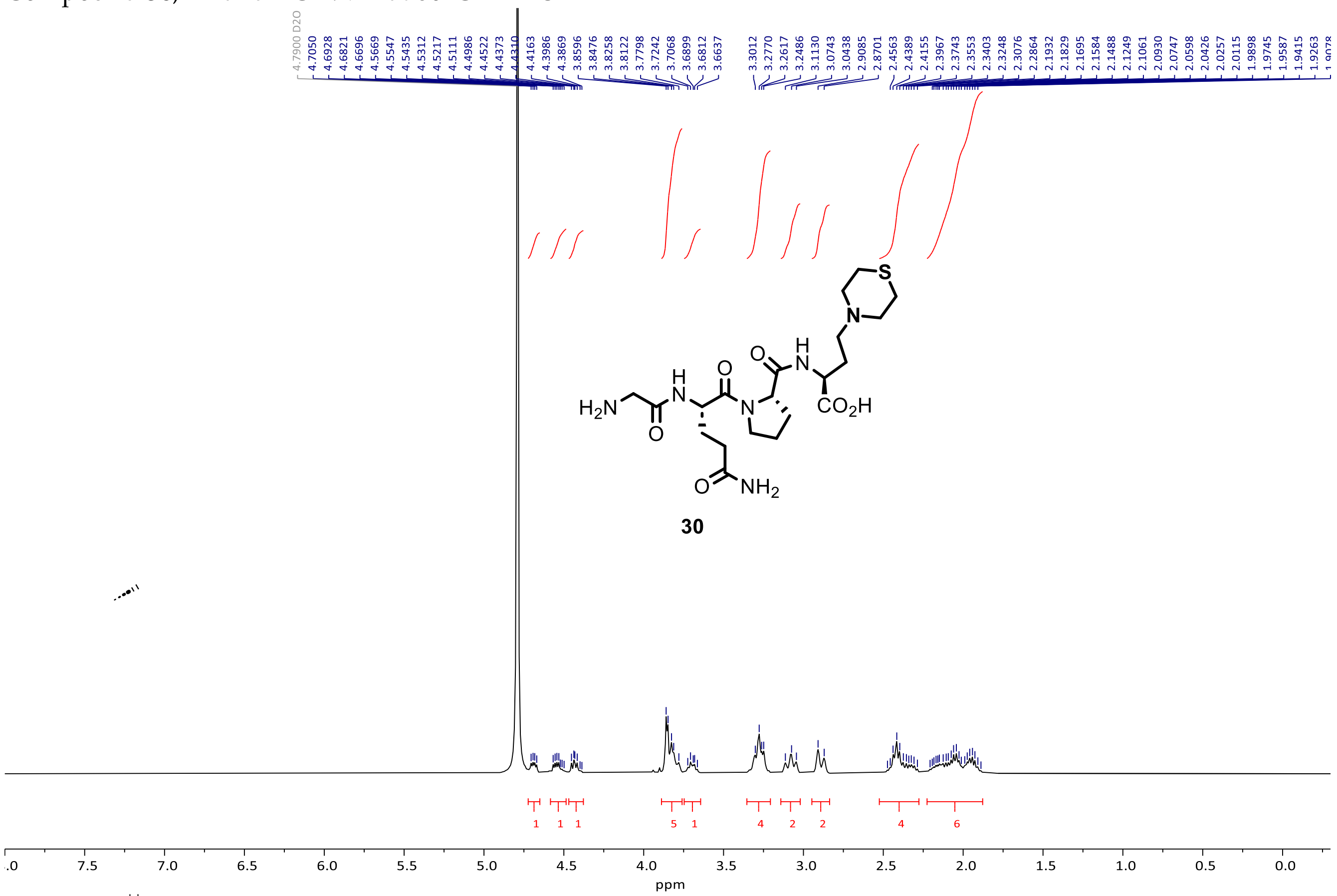

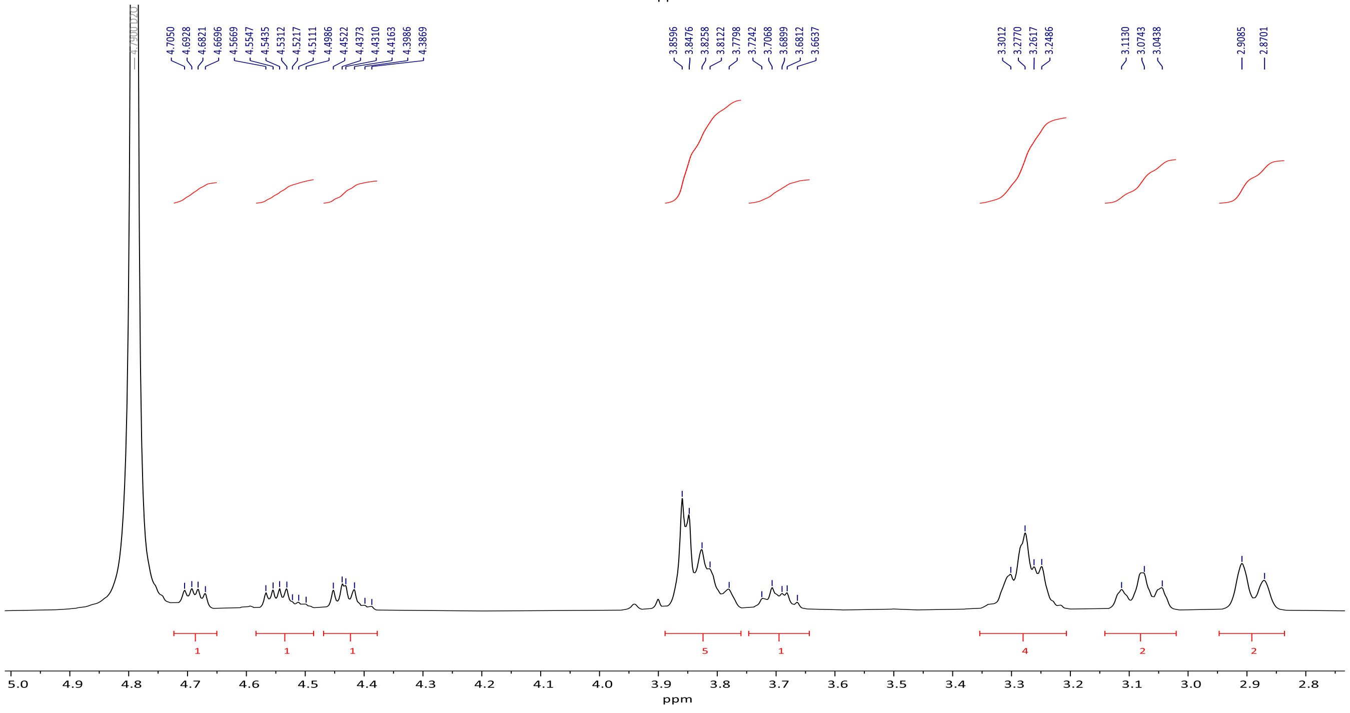

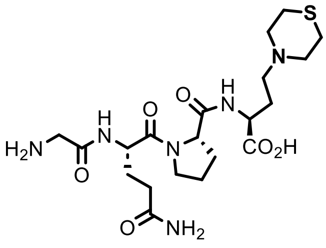

30

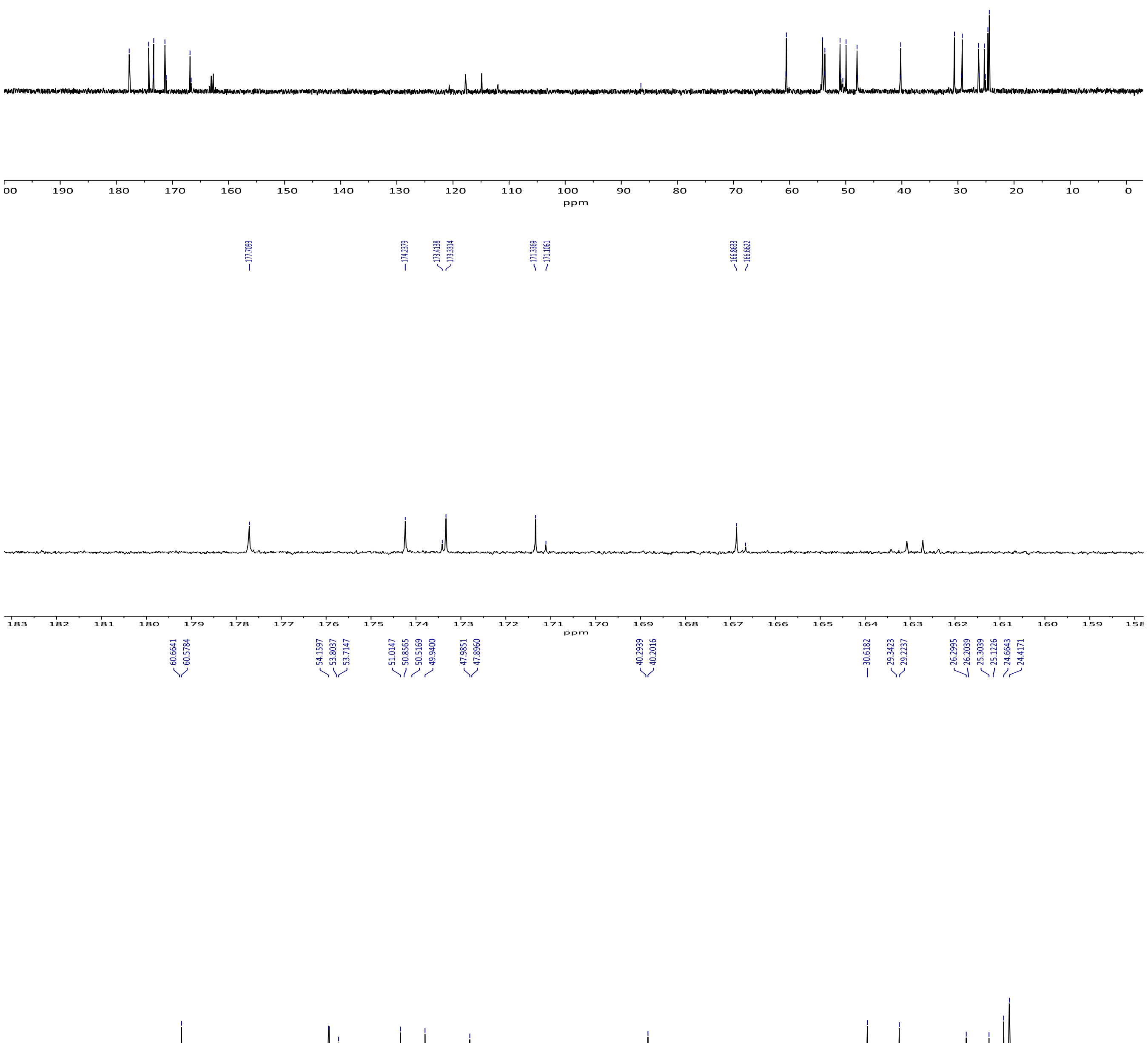

177.7093
174.2379
173.4138
173.3314
171.3369
171.1061
166.8633
166.6622
86.5100
60.6641
60.5784
54.1597
53.8037
53.7147
51.0147
50.8565
50.5169
49.9400
47.9851
47.8960
40.2939
40.2016
30.6182
29.3423
29.2237
26.2995
26.2039
25.3039
25.1226
24.6643
24.4171
ppm
ppm
ppm

1
